# A revision of the Chinese Trigonalyidae (Hymenoptera, Trigonalyoidea)

**DOI:** 10.3897/zookeys.385.6560

**Published:** 2014-02-28

**Authors:** Hua-yan Chen, Cornelis van Achterberg, Jun-hua He, Zai-fu Xu

**Affiliations:** 1 Department of Entomology, College of Natural Resources and Environment, South China Agricultural University, Guangzhou 510640, P. R. China; 2 Department of Entomology, The Ohio State University, 1315 Kinnear Road, Columbus, Ohio 43212, U.S.A.; 3 Department of Terrestrial Zoology, Naturalis Biodiversity Center, Postbus 9517, 2300 RA Leiden, Netherlands; 4 Institute of Insect Sciences, Zhejiang University, Hangzhou 310029, China

**Keywords:** Revision, Trigonalyidae, *Bakeronymus*, *Bareogonalos*, *Jezonogonalos*, *Lycogaster*, *Orthogonalys*, *Pseudogonalos*, *Taeniogonalos*, *Teranishia*, keys, new species, new synonyms, new combinations, China

## Abstract

The Chinese fauna of the family Trigonalyidae Cresson, 1887, is revised, keyed and fully illustrated for the first time. Eight genera of this family (*Bakeronymus* Rohwer, 1922, *Bareogonalos* Schulz, 1907, *Jezonogonalos* Tsuneki, 1991, **re-instated**, *Lycogaster* Shuckard, 1841, *Orthogonalys* Schulz, 1905, *Pseudogonalos* Schulz, 1906, *Taeniogonalos* Schulz, 1906 and *Teranishia* Tsuneki, 1991) are recorded from China. The genus *Ischnogonalos* Schulz, 1907, is synonymized with *Taeniogonalos* Schulz, 1906. In total 40 valid species are recognized. Twenty species are new for science: *Jezonogonalos elliptifera* **sp. n.**, *J. jiangliae* **sp. n.**, *J. luteata* **sp. n.**, *J. nigrata* **sp. n.**, *Lycogaster angustula* **sp. n.**, *L. flavonigrata* **sp. n.**, *L. nigralva* **sp. n.**, *Orthogonalys cheni* **sp. n.**, *O. clypeata* **sp. n.**, *O. robusta* **sp. n.**, *Pseudogonalos angusta* **sp. n.**, *Taeniogonalos bucarinata* **sp. n.**, *T. cordata* **sp. n.**, *T. geminata* **sp. n.**, *T. sculpturata* **sp. n.**, *T. triangulata* **sp. n.**, *T. tricolorisoma* **sp. n.**, *T. uncifera* **sp. n.**, *Teranishia crenulata* **sp. n.** and *T. glabrata* **sp. n.** Two species are reported new for China: *Orthogonalys elongata* Teranishi, 1929 and *Nanogonalos flavocincta* Teranishi, 1929 (renamed to *Taeniogonalos subtruncata* **nom. n.**). Seven new synonyms are proposed: *Poecilogonalos yuasai* Teranishi, 1938, and *P. maga taiwana* Tsuneki, 1991, of *Taeniogonalos taihorina* (Bischoff, 1914); *Taiwanogonalos minima* Tsuneki, 1991, and *T. similis* Tsuneki, 1991, of *Taeniogonalos alticola* (Tsuneki, 1991); *P. intermedia* Chen, 1949, and *P. unifasciata* Chen, 1949, of *Taeniogonalos formosana* (Bischoff, 1913). Six taxa are recognised as valid species: *Bakeronymus seidakka* Yamane & Terayama, 1983, *Jezonogonalos laeviceps* (Tsuneki, 1991), *J. satoi* (Tsuneki, 1991), *Taeniogonalos alticola* (Tsuneki, 1991), *T. flavoscutellata* (Chen, 1949) and *T. gestroi* (Schulz, 1908). Five new combinations are made: *Jezonogonalos laeviceps* (Tsuneki, 1991), **comb. n.**, *J. satoi* (Tsuneki, 1991), **comb. n.**, *Taeniogonalos flavoscutellata* (Chen, 1949), **comb. n.**, *T. gestroi* (Schulz, 1908), **comb. n.** and *T. lachrymosa* (Westwood, 1874), **comb. n.** Lectotypes are designated for *Lycogaster violaceipennis* Chen, 1949, *Poecilogonalos flavoscutellata* Chen, 1949, *P. rufofasciata* Chen, 1949, and *P. tricolor* Chen, 1949.

## Introduction

Trigonalyidae (Hymenoptera) is a worldwide small family in its own superfamily Trigonalyiodea, with 93 recognized species (Carmean and Kimsey 1998; Smith and Stocks 2005; Santos et al. 2012; Smith and Tripotin 2012; Smith et al. 2012). Most species of this family occur in tropical and subtropical regions, but only one species occurs in Europe and the family is absent in arctic and alpine habitats (Carmean and Kimsey 1998). Carmean and Kimsey (1998) recognized two subfamilies: Orthogonalinae (corrected by Lelej (2003) to Orthogonalyinae) and Trigonalinae (correctly: Trigonalyinae) from their morphological phylogenetic analysis. The subfamily Trigonalyinae is subdivided in two tribes: Nomadinini and Trigonalini (correctly: Trigonalyini). According to their analysis the Orthogonalyinae are without derived characters and the recognition of the tribe Nomadinini makes the Trigonalyini paraphyletic. Therefore, we do not recognise these higher categories untill there is a better understanding of the evolution within this family. The distribution of the genera of the Trigonalyidae varies, but most genera known from China are wide spread. *Bakeronymus* occurs only in the Oriental Region and *Pseudogonalos* is only known from the Palaearctic Region. *Jezonogonalos* and *Teranishia* occur in the Oriental and East Palaearctic Regions, *Bareogonalos* and *Lycogaster* occur in the Oriental, East Palaearctic, Nearctic and Central American Regions, and *Orthogonalys* occurs in the Afrotropical, Oriental, East Palaearctic, Nearctic and Neotropical Regions. *Taeniogonalos* has a similar distribution but *Taeniogonalos* is also known from the Australian Region. The genus *Trigonalys* Westwood is most likely restricted to the Neotropical and Afrotropical Regions; the two Oriental species (Carmean and Kimsey 1998) are removed in this paper.

**Figure 1.**
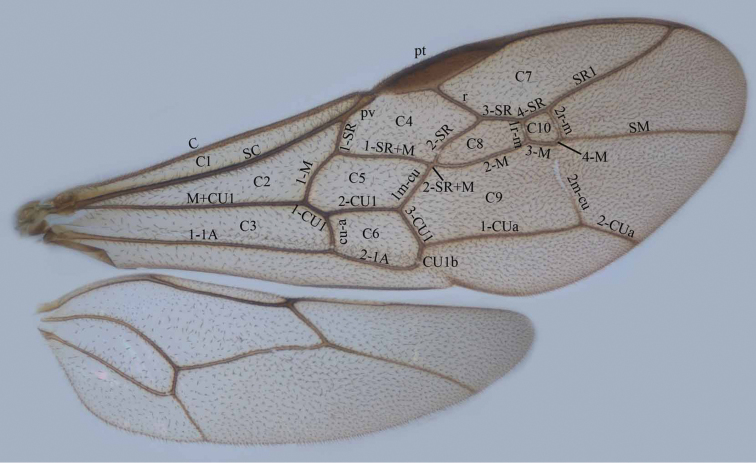
Wings of Trigonalyidae. **C1** costal cell **C2** medial cell **C3** submedial cell **C4** first submarginal cell **C5** discal cell **C6** subdiscal cell **C7** marginal cell **C8** second submarginal cell **C9** second discal cell **C10** third submarginal cell **pt** pterostigma **pv** parastigmal vein.

Trigonalyidae are often misidentified; slender specimens (especially of *Orthogonalys*) with white banded antennae may be mistaken for Ichneumonidae and robust specimens with black antennae for aculeate wasps (e.g. of the family Crabronidae). They can be identified by the combination of the well-developed costal cell of the fore wing (C1 in Fig. 1), the presence of mandibles with 3–5 large teeth (Figs 7, 19, 39, 145, 156, 243, 561, 594) and the presence of plantar lobes on the tarsal segments (Fig. 5). In addition, the tarsal claws are cleft (bifurcate with the inner tooth larger than the outer one: Fig. 5) and as pointed out in Carmean and Kimsey (1998) the females have sparse white scales or specialized setae on the outside of the middle antennal segments.

**Figures 2–5.**
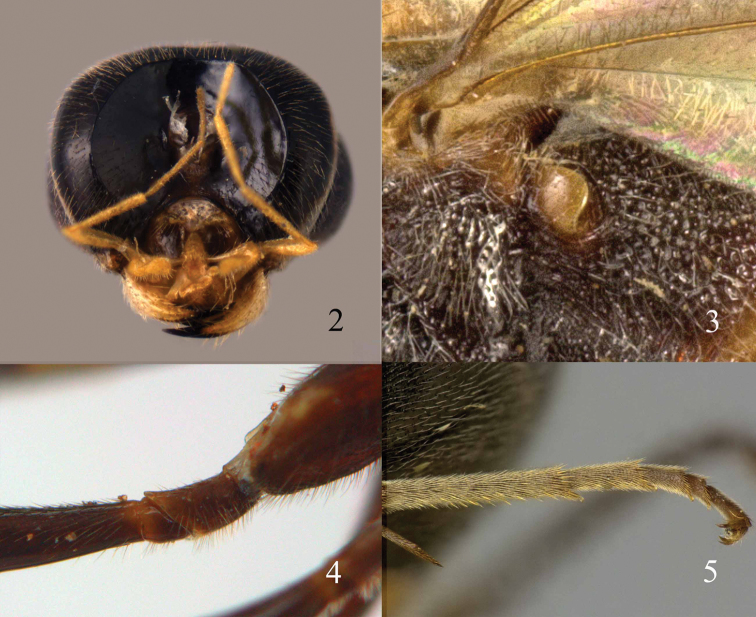
Trigonalyidae **2** *Orthogonalys cheni* sp. n., head posterior **3** *Taeniogonalos rufofasciata* (Chen, 1949), propodeal spiracle **4** *Bakeronymus seidakka* Yamane & Terayama, 1983, hind trochanter and trochantellus **5** *Taeniogonalos fasciata* (Strand, 1913), hind tarsus.

Biologically, species of Trigonalyidae seem not to care about laying the eggs at or in the future food source as is normal for parasitoids. They deposit up to more than 2000 eggs on leaves, which are eaten eventually by caterpillars and sawfly larvae. In the digestive tract the eggs hatch, the mobile larva bores through the intestine wall to search for an eventually present larva of a parasitoid wasp (Ichneumonidae or Braconidae) or of a parasitoid fly (Tachinidae) to develop on it as a hyperparasitoid. The third larval instar has huge mandibles to kill possibly present other parasitoid larvae. Other species are brought into the nests of social Vespidae because they are inside the caterpillars used as prey by the wasps. In the wasp larvae they develop as primary endoparasitoids. Primary endoparasitism of sawflies occurs, but the parasitoid still acts facultatively as a hyperparasitoid (Yamane and Terayama 1983; He and Chen 1986; Weinstein and Austin 1991; Carmean and Kimsey 1998).

The Chinese fauna of Trigonalyidae has never been revised and fully illustrated. Chen (1949) is the only author to exclusively deal with the Chinese fauna, and he listed 16 species belonging to 6 genera from China (including Taiwan) and included two elementary keys, one to the genera and the other to the species of *Poecilogonalos* (now in *Taeniogonalos*). Yamane and Yamane (1975) and Yamane and Terayama (1983) described one new species and one new subspecies from Taiwan in two additional genera. Two species were synonymized (*Poecilogonalos flavoscutellata* Chen, 1949, and *Taeniogonalos pictipennis* Strand, 1914, with *Taeniogonalos sauteri* Bischoff, 1913) by Tsuneki (1991). In addition, he added seven new species from Taiwan in his new genus *Taiwanogonalos*, but without examination of the types (except of *Taeniogonalos alishana*). Carmean and Kimsey (1998) synonymized all with *Taeniogonalos maga* (Teranishi, 1929). *Poecilogonalos unifasciata* Chen, 1949, was synonymized with *Poecilogonalos maga* (Teranishi, 1929) by Marshakov (1981). *Lycogaster celebesiensis* (Szépligeti, 1902) was reported from China, the genera *Nanogonalos* and *Poecilogonalos* were synonymized with *Taeniogonalos* and *Poecilogonalos magnifica* Teranishi, 1929, was synonymized with *Taeniogonalos fasciata* (Strand, 1913) by Carmean and Kimsey (1998). As a result, currently 16 species in 6 genera are known from China (Table 1), of which 15 species are considered valid; one is a synonym and two species are misidentified. In total, 40 valid species occurring in China and belonging to eight genera have been examined during this study. Twenty are new species and described in this paper and two species are new for China (one of which is renamed; Table 2). The actual number will be higher because several species from neighbouring countries are expected to occur also in China (e.g., in the northern provinces of China species known from Japan); in conclusion, the Chinese fauna is still clearly undersampled.

**Table 1.** List of the Chinese species of the family Trigonalyidae before this study with distribution in China.

Species	Distribution
*Bakeronymus typicus seidakka* Yamane & Terayama, 1983	Taiwan (Oriental)
*Bareogonalos huisuni* Yamane & Yamane, 1975	Taiwan (Oriental)
*Lycogaster celebesiensis* (Szépligeti, 1902)	Jiangxi (Oriental) [Misidentification; *Lycogaster flavonigrata*]
*Lycogaster violaceipennis* Chen, 1949	Zhejiang (Oriental)
*Orthogonalys formosana* Teranishi, 1931	Taiwan (Oriental)
*Pseudogonalos hahnii* (Spinola, 1840)	Hebei (Palaearctic)
*Taeniogonalos fasciata* (Strand, 1913)	Zhejiang, Taiwan (Oriental)
*Taeniogonalos formosana* (Bischoff, 1913)	Taiwan (Oriental)
*Taeniogonalos intermedia* (Chen, 1949)	Zhejiang (Oriental)
*Taeniogonalos maga* (Teranishi, 1929) = *Poecilogonalos unifasciata* Chen, 1949 = *Poecilogonalos maga taiwana* Tsuneki, 1991 = *Taiwanogonalos alticola* Tsuneki, 1991 = *Taiwanogonalos laeviceps* Tsuneki, 1991 = *Taiwanogonalos minima* Tsuneki, 1991 = *Taiwanogonalos satoi* Tsuneki, 1991 = *Taiwanogonalos similis* Tsuneki, 1991	Zhejiang, Taiwan (Oriental)
*Taeniogonalos mongolica* (Popov, 1945)	Inner Mongolia (Palaearctic)
*Taeniogonalos rufofasciata* (Chen, 1949)	Jiangxi (Oriental)
*Taeniogonalos sauteri* Bischoff, 1913	Taiwan (Oriental)
*Taeniogonalos taihorina* (Bischoff, 1914)	Taiwan (Oriental)
*Taeniogonalos thwaitesii* (Westwood, 1874)	Taiwan (Oriental) [Misidentification; *Taeniogonalos gestroi*]
*Taeniogonalos tricolor* (Chen, 1949)	Zhejiang (Oriental)

**Table 2.** List of the Chinese species of the family Trigonalyidae Cresson after this study with distribution in China.

Species	Distribution in China
*Bakeronymus seidakka* Yamane & Terayama, 1983, re-instated	Taiwan (Oriental)
*Bareogonalos huisuni* Yamane & Yamane, 1975	Taiwan (Oriental)
*Jezonogonalos elliptifera* sp. n.	Sichuan (Oriental)
*Jezonogonalos jiangliae* sp. n.	Tibet (Oriental)
*Jezonogonalos laeviceps* (Tsuneki, 1991), comb. n., re-instated	Taiwan (Oriental)
*Jezonogonalos luteata* sp. n.	Sichuan (Oriental)
*Jezonogonalos nigrata* sp. n.	Sichuan (Oriental)
*Jezonogonalos satoi* (Tsuneki, 1991), comb. n., re-instated	Taiwan (Oriental)
*Lycogaster angustula* sp. n.	Zhejiang (Oriental)
*Lycogaster flavonigrata* sp. n.	Fujian, Jiangxi, Yunnan (Oriental)
*Lycogaster nigralva* sp. n.	Sichuan (Oriental)
*Lycogaster violaceipennis* Chen, 1949	Zhejiang, Sichuan (Oriental)
*Orthogonalys cheni* sp. n.	Zhejiang, Hubei, Sichuan (Oriental)
*Orthogonalys clypeata* sp. n.	Ningxia, Shaanxi (Palaearctic), Guizhou, Sichuan, Yunnan (Oriental)
*Orthogonalys elongata* Teranishi, 1929	Henan (Palaearctic), Sichuan, Tibet (Oriental)
*Orthogonalys formosana* Teranishi, 1931	Taiwan (Oriental)
*Orthogonalys robusta* sp. n.	Shaanxi (Palaearctic), Guangxi (Oriental)
*Pseudogonalos hahnii* (Spinola, 1840)	Liaoning, Inner Mongolia, Beijing, Hebei, Henan (Palaearctic)
*Pseudogonalos angusta* sp. n.	Inner Mongolia (Palaearctic)
*Taeniogonalos alticola* (Tsuneki, 1991), re-instated = *Taiwanogonalos minima* Tsuneki, 1991, syn. n. = *Taiwanogonalos similis* Tsuneki, 1991, syn. n.	Taiwan (Oriental)
*Taeniogonalos bucarinata* sp. n.	Gansu, Ningxia, Shaanxi, Henan (Palaearctic), Zhejiang, Fujian, Sichuan, Yunnan (Oriental)
*Taeniogonalos cordata* sp. n.	Yunnan (Oriental)
*Taeniogonalos fasciata* (Strand, 1913)	Jilin, Liaoning, Shaanxi, Henan, Anhui (Palaearctic), Zhejiang, Taiwan, Fujian, Hunan, Guangdong, Guangxi, Hainan, Guizhou (Oriental)
*Taeniogonalos flavoscutellata* (Chen, 1949), comb. n., re-instated	Beijing, Shangdong (Palaearctic), Zhejiang, Fujian, Hunan (Oriental)
*Taeniogonalos formosana* (Bischoff, 1913) = *Poecilogonalos intermedia* Chen, 1949, syn. n. = *Poecilogonalos unifasciata* Chen, 1949, syn. n.	Jilin, Shanxi, Ningxia, Henan (Palaearctic), Zhejiang, Fujian, Taiwan, Guangdong, Sichuan, Yunnan, Guizhou, Tibet (Oriental)
*Taeniogonalos geminata* sp. n.	Zhejiang (Oriental)
*Taeniogonalos gestroi* (Schulz, 1908), comb. n., re-instated	Jiangsu (Palaearctic), Hainan, Taiwan, Yunnan (Oriental)
*Taeniogonalos maga* (Teranishi, 1929)	Taiwan, Zhejiang (Oriental)
*Taeniogonalos mongolica* (Popov, 1945)	Inner Mongolia (Palaearctic)
*Taeniogonalos rufofasciata* (Chen, 1949)	Fujian, Jiangxi (Oriental)
*Taeniogonalos sauteri* Bischoff, 1913	Shandong (Palaearctic), Zhejiang, Fujian, Taiwan, Hunan, Guangxi (Oriental)
*Taeniogonalos sculpturata* sp. n.	Guizhou (Oriental)
*Taeniogonalos subtruncata* nom. n. = *Nanogonalos flavocincta* Teranishi, 1929	Shaanxi (Palaearctic)
*Taeniogonalos taihorina* (Bischoff, 1914) = *Poecilogonalos yuasai* Teranishi, 1938, syn. n. = *Poecilogonalos maga taiwana* Tsuneki, 1991, syn. n.	Heilongjiang, Gansu, Ningxia (Palaearctic), Zhejiang, Fujian, Taiwan, Hubei, Guangxi, Sichuan, Yunnan, Tibet (Oriental)
*Taeniogonalos triangulata* sp. n.	Yunnan (Oriental)
*Taeniogonalos tricolor* (Chen, 1949)	Shaanxi, Henan (Palaearctic), Zhejiang, Hubei, Fujian, Jiangxi, Hainan, Guangxi, Sichuan, Guizhou, Yunnan (Oriental)
*Taeniogonalos tricolorisoma* sp. n.	Guizhou, Yunnan (Oriental)
*Taeniogonalos uncifera* sp. n.	Sichuan (Oriental)
*Teranishia crenulata* sp. n.	Ningxia, Gansu (Palaearctic), Sichuan (Oriental)
*Teranishia glabrata* sp. n.	Ningxia, Henan (Palaearctic), Zhejiang, Sichuan (Oriental)

All known species of Trigonalyidae from China are revised, illustrated and keyed. Unfortunately, several of the new and described species have males as holotype and the associated female is unknown. Collection at the type localities and the use of COI (“barcoding”) will recover the conspecific female.

## Material and methods

Descriptions of the species are a combination of comparative illustrations and a short diagnosis and description, followed by a summary of the distribution (only based on examined specimens unless otherwise indicated) and biology (as far as known, at least the phenology is given). Descriptions were made under either an Olympus SZ61 or SZ40 stereoscope, in combination with a 40 W LED lamp or a 27 W fluorescent lamp. Photographic images were made with an Olympus motorized stereomicroscope SZX12 and Stemi 2000-CS; the images were processed with both Image-Pro Plus and AnalySIS Extended Focal Imaging software, and plates were finished with ACDSee 10.0 and Photoshop CS, mostly to adjust the size and background.

Morphology. For other terminology used in this paper, see van Achterberg (1988, 1993) and Hong et al. (2011). Measurements are taken as indicated by van Achterberg (1988); width of the propodeal foramen is measured dorsally and its medial height in lateral view. Additional non-exclusive characters in the key are between brackets.

Material. Types and other specimens have been examined from the following institutions:

BMNH Natural History Museum, London, U. K. (Mr. D. Notton).

CASSF California Academy of Sciences, San Francisco, California, USA (Dr. W. Pulawski).

ECUS Entomology Collection of University of Sapporo, Sapporo, Japan (Dr. M. Ohara).

HUS Entomological Institute, Hokkaido University, Sapporo, Japan (Dr. Masahiro Ohara).

IZCAS Institute of Zoology, Chinese Academy of Sciences, Beijing, China (Dr. Jun Chen, Mr. Jian Yao, Mrs. Hong Liu).

KBIN Koninklijk Belgisch Instituut voor Natuurwetenschappen, Brussels (Mr. Y. Gerard).

NHMS Natural History Museum, Stockholm, Sweden (Dr. H. Vårdal, Dr. S. Klopfstein).

OMNH Osaka Museum of Natural History, Osaka, Japan (Dr. Rikio Matsumoto).

OPU Entomological Laboratory, Osaka Prefecture University, Sakai, Osaka, Japan (Dr. Norio Hirai, Dr. Shigeki Kobayashi).

RMNH Naturalis Biodiversity Center, Leiden, Netherlands (including collections from former National Museum of Natural History, Leiden, the Entomological Institute, Wageningen and the Zoological Museum, Amsterdam).

SCAU Hymenoptera Collection, South China Agricultural University, Guangzhou, China.

SDEI Senckenberg Deutsche Entomologische Institut, Müncheberg, Germany (Dr A. Taeger, Mr A. Liston).

SEMC Shanghai Entomological Museum, Shanghai, China (Dr. Hai-sheng Ying).

USNM National Museum of Natural History, Smithsonian Institution, Washington D. C., USA (Dr. D. Smith).

ZISP Zoological Institute, Academia NAUK, St. Petersburg, Russia (Dr. S.A. Belokobylskij).

ZJUH Institute of Insect Sciences, University of Zhejiang, Hangzhou, China (Prof. Dr. Xue-xin Chen and Prof. Jun-hua He).

ZMB Zoologisches Museum, Humboldt Universität, Berlin, Germany (Dr. F. Koch, Mrs. V. Richter).

## Systematics

### Trigonalyidae

Cresson, 1887

http://species-id.net/wiki/Trigonalyidae

Trigonalyidae Cresson, 1887: 183; Carmean and Kimsey 1998: 54 (as Trigonalidae, corrected by Krieger (1894) to Trigonalyidae).
Trigonalydae Magretti, 1897: 311.
Trigonalydidae Schaufuss, 1905: 34.
Trigonaloidae Schulz, 1907: 1.
Bareogonaloinae Schulz, 1907: 4, 18.
Disceneinae Benoit, 1951: 143.
Lycogastrinae Schulz, 1907: 10.
Nippogonaloinae Uchida, 1929: 79.
Nomadininae Cameron, 1899: 2 (as Nomadinae, corrected by Schulz (1907) to Nomadininae).
Orthogonalyinae Carmean & Kimsey, 1998: 52 (as Orthogonalinae, corrected by Lelej (2003) to Orthogonalyinae).
Phytogonalinae Schulz, 1905: 102, 104.
Seminotinae Schulz, 1907: 4.

#### Diagnosis.

Body length between 3–15 mm; antenna of both sexes with 13–32 segments, males may have smooth more or less protruding sensillae (tyloids; Figs 51, 56, 77, 100, 266, 271, 283, 295, 315, 575) usually on 11^th^–14^th^ antennal segments; females have sparse white scales or specialized seta on the outside of the middle antennal segments (Carmean and Kimsey 1998); eye relatively long, extending almost to level of mandible; mandibles large and usually asymmetrical (with 3–4 large teeth on left mandible and 4–5 teeth on right mandible), and distinctly overlapping when closed; maxillary palp 6-segmented and labial palp 3-segmented, but palpi reduced in *Nomadina*, *Bakeronymus* and *Pseudonomadina* (maxillary palp with 2–4 segments and labial palp 2-segmented); occipital (or genal) carina ending at hypostomal carina near mandibular condylus (Fig. 2) or below mandible; propleuron short in front of mesoscutum; fore wing with wide costal cell (C1 in Fig. 1) and at least 9 closed cells; hind wing with 2 closed cells and no postero-basal lobe; all legs with trochanter and trochantellus, but dorsal triangular part of trochanter of hind leg (not trochantellus as mentioned by Carmean and Kimsey 1998) differentiated by an oblique groove (Fig. 230), except in *Boreogonalos*, *Nomadina*, *Bakeronymus* and *Pseudonomadina* where the hind trochanter is undivided (Fig. 4); plantar lobes of tarsal segments present (Fig. 5); tarsal claws cleft (bifurcate), with inner tooth larger than outer one (Fig. 5); propodeal spiracle covered by a flap (Fig. 3), but minute in *Bakeronymus*; propodeal foramen rounded (“U-shaped”) or arched (“V-shaped”) dorsally and often with distinct dorsal carina or rim (Figs 12, 23, 34, 114, 161, 249, 491, 599); median carina of propodeum often absent or reduced but distinct in *Pseudogonalos* (Fig. 260); metasoma low on propodeum; ovipositor not exposed. Males can be recognised by having a pair of thin flaps (parameres) at apex of metasoma, and 6–7 sternites visible and second sternite flattened or sometimes slightly concave medio-posteriorly. Females have an undivided (usually triangular, but sometimes (*Lycogaster*) truncate or emarginate) sternite (= hypopygium) apically, with 5 additional sternites visible and second sternite often convex or protruding medio-posteriorly.

#### Note.

Although Trigonalidae has been more commonly used (Carmean and Kimsey 1998), we follow Weinstein and Austin (1991) and Lelej (2003) in using the family name as corrected by Krieger (1894) to Trigonalyidae; for the argumentation see Lelej (2003).

#### Key to Oriental and Palaearctic genera of Trigonalyidae

**Table:** 

1	Antenna with 13–15 segments; vertex with medio-longitudinal depression dorsally (Fig. 8); vein 1-SR of fore wing short (Fig. 17); mandibles narrow in anterior view, mandibular condyli far from eye (except in *Bakeronymus typicus*) and submedially attached to head (Fig. 7); apical segment of labial palp slender and tapered; protuberance of third sternite much larger than of second sternite in ♀ (Fig. 15); hind tarsus strongly modified	2
–	Antenna with 17–32 segments; vertex normal, at most with slight median depression dorsally (Fig. 20); vein 1-SR of fore wing medium-sized to long (Figs 28, 33, 112, 172, 246, 410, 597); mandibles wide in anterior view and sublaterally attached to head (Fig. 54), but mandibular condyli distinctly removed from eye in *Bareogonalos*: Fig. 19); apical segment of labial palp widened and obtuse, more or less triangular (Fig. 22); protuberance of third sternite of ♀ absent (Fig. 416), **if** present then much smaller than of second sternite in ♀ or of similar size (Fig. 26; *Bareogonalos*); hind tarsus slightly or not modified	3
2	Maxillary palp 2-segmented and about 0.4 times length of mandible; first metasomal tergite comparatively wide basally; third antennal segment distinctly longer than fourth segment and widened; apical half of antenna of female with distinctly moniliform and asymmetric segments; anterior ocellus much lower situated than posterior ocelli; [antennal sockets beside mandibular condyli; not known from China]	*Pseudonomadina* Yamane & Kojima, 1982
–	Maxillary palp 4-segmented and about 0.7 times as long as mandible (Fig. 11); first tergite subpetiolate and comparatively narrow basally (Fig. 14); third antennal segment 0.8 times as long as fourth segment and slender (Fig. 9); apical half of antenna of female with hardly moniliform and symmetric segments (Fig. 9); anterior ocellus slightly lower situated than posterior ocelli (Fig. 8)	*Bakeronymus* Rohwer, 1922
3	Mandibular condyli remain far removed from eyes (Fig. 22); hind trochanter simple, dorsal triangular part not separated from basal part; metanotum nearly flat and smooth medially (Fig. 23); fore trochanter distinctly widened apically and slightly longer than hind trochanter; [antenna of ♂ without tyloids; head transverse and flattened dorsally (Fig. 20); propodeum strongly rugose; metasoma usually smooth and shiny]	*Bareogonalos* Schulz, 1907
–	Mandibular condyli close to level of eyes (Fig. 54); triangular dorso-apical part of hind trochanter separated by an oblique groove (Fig. 230); metanotum variable, usually convex and sculptured medially (Figs 44, 249); fore trochanter subparallel-sided and distinctly longer than hind trochanter	4
4	Basal half of third metasomal sternite with a posteriorly steep, smooth and complete transverse ledge (Fig. 118; may be partly hidden under second sternite and rather low in *Lycogaster violaceipennis*); second sternite with pair of small triangular teeth on apical protuberance (Fig. 118; but only with pair of lobe-shaped flaps in male of *Lycogaster violaceipennis*); fifth sternite of ♀ distinctly emarginate medio-posteriorly (Fig. 118); metanotum smooth, shiny and weakly convex (Fig. 114); [supra-antennal elevations small; antenna of ♀ widened medially (Fig. 111, but hardly so in *Lycogaster angustula* sp. n.); antenna of ♂ without tyloids; epipleura of tergites laterally strongly pigmented; vertex convex and shiny]	*Lycogaster* Shuckard, 1841
–	Basal half of third sternite flat, without a distinct ledge anteriorly (Fig. 264), **if** sternite somewhat protruding or with a ledge (Fig. 592) then only submedially so (some *Taeniogonalos* spp.) or near apex of sternite situated and ledge distinctly sculptured (*Taeniogonalos sauteri*; Fig. 473); second sternite without pair of small teeth medio-apically (Fig. 264); fifth sternite of ♀ straight or slightly emarginate medio-posteriorly (Fig. 416); metanotum often sculptured, matt and distinctly convex (Figs 44, 249); [hypopygium of ♀ triangular; vertex comparatively flat and rectangular]	5
5	Anterior propodeal sulcus smooth laterally and medially narrow (Figs 323, 491); outer orbita punctate (Fig. 587); posterior propodeal carina (above foramen) arched dorsally and often slightly protruding anteriorly (Fig. 578); 11^th^ –14th antennal segments of ♂ with linear tyloids (Figs 315, 575); [second sternite sometimes with a medial elevation posteriorly (Fig. 592); third sternite at most 0.7 times as long as second sternite; hypopygium of ♀ pointing anteriorly toward second sternite or straight down or pointing posteriad]	*Taeniogonalos* Schulz, 1906
–	Anterior propodeal sulcus crenulate laterally (Figs 59, 599) and medially often widened (Figs 161, 260); outer orbita smooth or largely so (Fig. 598); posterior propodeal carina (above foramen) curved dorsally (Old World genera) and often distinctly protruding dorsally (Fig. 59); antenna of ♂ without tyloids (Fig. 199) or tyloids (as shiny elevated patches) circular or elliptical (Figs 51, 266)	6
6	Supra-antennal elevations hardly protruding and united medially, forming a horizontal “shelf” between antennal bases; posterior propodeal carina arched dorsally and connected to foramen medio-anteriorly; occipital carina ending at lateral edge of mandibular base and protruding beyond level of mandibular base and malar space in anterior view nearly rectangularly narrowed ventrally; posterior margin of first metasomal tergite more or less curved; [sixth tergite of ♀ rectangular posteriorly or nearly so; veins m-cu and 1-M of fore wing subparallel; second tergite and sternite of ♀ convex; unknown from China and probably only in the New World. Both Old World species included by Carmean and Kimsey (1998) (*Trigonalys lachrymosa* Westwood, 1874, from Philippines and *Trigonalys rufiventris* Magretti, 1897, from Myanmar) were included in *Lycogaster* by Weinstein and Austin (1991). We agree with this placement; see also note under *Lycogaster*]	*Trigonalys* Westwood, 1835
–	Supra-antennal elevations conspicuously protruding and remain far separated from each other medially, without horizontal “shelf” between antennal bases (Figs 75, 157, 244, 595); posterior propodeal carina (above propodeal foramen) curved medio-dorsally (Fig. 161) and foramen often somewhat separated from carina medio-anteriorly; occipital carina ending at hypostomal carina at level of mandibular base and malar space gradually narrowed in anterior view (Fig. 54); posterior margin of first metasomal tergite straight (Fig. 262)	7
7	Supra-antennal elevations nearly parallel-sided in dorsal view (Figs 244, 256); area above supra-antennal elevations distinctly depressed and smooth (Fig. 244); scutellum more or less coarsely reticulate-punctate (Fig. 249); hind trochanter black; [occipital carina widened medio-dorsally; body black (no pale patches, at most malar space and margins of basal metasomal sternites and tergites narrowly ivory); fore wing with large dark patch below pterostigma]	*Pseudogonalos* Schulz, 1906
–	Supra-antennal elevations triangular in dorsal view (Figs 75, 157, 595); area above supra-antennal elevations flattened or at most moderately depressed (Figs 75, 157, 595); scutellum largely smooth, except for some punctures (Figs 59, 174, 599), punctulate or densely rugulose-punctate; hind trochanter usually pale yellow or ivory	8
8	Area above supra-antennal elevations shallowly depressed anteriorly (Figs 595, 606); inner side of supra-antennal elevations flat and smooth; area between elevations with small protuberance (Figs 595, 606); posteriorly medio-longitudinal carina of propodeum coarsely developed (Figs 599, 610); [male antenna without tyloids]	*Teranishia* Tsuneki, 1991
–	Area above supra-antennal elevations flat anteriorly (Figs 40, 157); inner side of supra-antennal elevations weakly convex and with some striae, narrow grooves and/or punctures; area between elevations evenly concave or with small pit (Figs 54, 169); posteriorly medio-longitudinal carina of propodeum usually absent (Figs 80, 174), if present then at most moderately developed	9
9	Tyloids on 11^th^ –14th antennal segments of ♂ absent (Fig. 199); second metasomal tergite flat in lateral view (Fig. 164); apical third of antenna often with a pale band (Fig. 158); occipital carina narrow and without crenulae medio-dorsally (Fig. 157), but widened and finely crenulate in *Orthogonalys hagoromonis*); fore wing subhyaline (Fig. 159), at most somewhat infuscate below pterostigma in female; [body often slender and sometimes ichneumonid-like (Fig. 231); posterior propodeal carina reduced in *Orthogonalys formosana* and *Orthogonalys hagoromonis*]	*Orthogonalys* Schulz, 1905
–	Male antenna with nearly circular or elliptical tyloids (visible as shiny elevated elongate patches) on 11^th^–14^th^ segments (Figs 51, 56, 77); second tergite slightly convex in lateral view (Fig. 47); antenna entirely black or dark brown (Figs 41, 76); occipital carina widened, lamelliform and with at least one short carina medio-dorsally (Fig. 75); subapical dark patch of fore wing more or less developed (Figs 42, 57, 68, 78, 89, 101)	*Jezonogonalos* Tsuneki, 1991

### Bakeronymus

Rohwer, 1922

http://species-id.net/wiki/Bakeronymus

Figs 6 –17

Bakeronymus Rohwer, 1922: 417; Weinstein and Austin 1991: 417; Carmean and Kimsey 1998: 59. Type species (by original designation): *Bakeronymus typicus* Rohwer, 1922 (examined).

#### Diagnosis.

Body length 7.7–10.1 mm; antenna with 13–15 segments, of female third antennal segment 0.8 times as long as fourth segment and slender (Fig. 9); apical half of antenna of female and of male with hardly moniliform and symmetric segments (Fig. 9); male antenna without tyloids; antennal sockets closer to eyes than to each other; vertex with medio-longitudinal depression dorsally, widened anteriorly and posteriorly (Fig. 8); anterior ocellus slightly lower situated than posterior ocelli in depression (Fig. 8); clypeus not differentiated from face; mandibles narrow in anterior view, mandibular condyli far from eye and submedially attached to head (Fig. 10); maxillary and labial palpi with 4 and 2 segments, respectively and apical segment of labial palp slender and tapered; maxillary palp about 0.7 times as long as mandible and with distinct segments (Fig. 11); vein 1-SR of fore wing short (Fig. 17); hind basitarsus enlarged; hind trochanter without oblique groove; propodeal foramen rounded dorsally and with lamelliform rim (Fig. 12); first tergite subpetiolate and comparatively narrow basally (Fig. 14); protuberance of second sternite of ♀ small and forked, and of third sternite large and obtuse.

#### Biology.

Reared from the nest of Polistinae (Vespidae) (Yamane and Terayama 1983).

### Bakeronymus seidakka

Yamane & Terayama, 1983 re-instated

http://species-id.net/wiki/Bakeronymus_seidakka

Figs 6 –17

Bakeronymus typicus seidakka Yamane & Terayama, 1983: 169; Weinstein and Austin 1991: 418; Carmean and Kimsey 1998: 59.

#### Type material.

Paratype, ♀ (RMNH), “[China:] Taiwan, Nantou county, Renai town, Nanfeng village”, “[collected] 12.VIII.1980, r[eared] from *Parapolybia varia*, emer[ged] 14–16.VIII.[1980], M. Terayama”, “Paratype ♀ *Bakeronymus typicus seidakka* ssp. n. Yamane et Terayama, 1983”. The holotype ♀ (HUS) and 2 ♀ paratypes (Yamane Collecton, Kagoshima University) are not examined.

#### Diagnosis.

Eyes remain distinctly separated from mandibular condyli; fourth antennal segment slightly widened apically; middle lobe of mesoscutum with large yellow patch; truncate apical part of scutellum 0.7 times as wide as scutellum anteriorly; fore wing with three submarginal cells; apical half of hind basitarsus ivory; propodeum reticulate-rugose, with short rugae interconnecting transverse rugae.

#### Description.

Female paratype, length of body 10.1 mm (of fore wing 9.3 mm).

*Head*. Antenna with 14 segments; frons and vertex smooth and strongly shiny (Fig. 7), glabrous; head gradually narrowed behind eyes (Fig. 8); temple smooth and strongly shiny; occipital carina narrow medio-dorsally; supra-antennal elevations hardly developed as a thin rim and smooth; clypeus slightly protruding and thin medio-ventrally.

**Figures 6–9.**
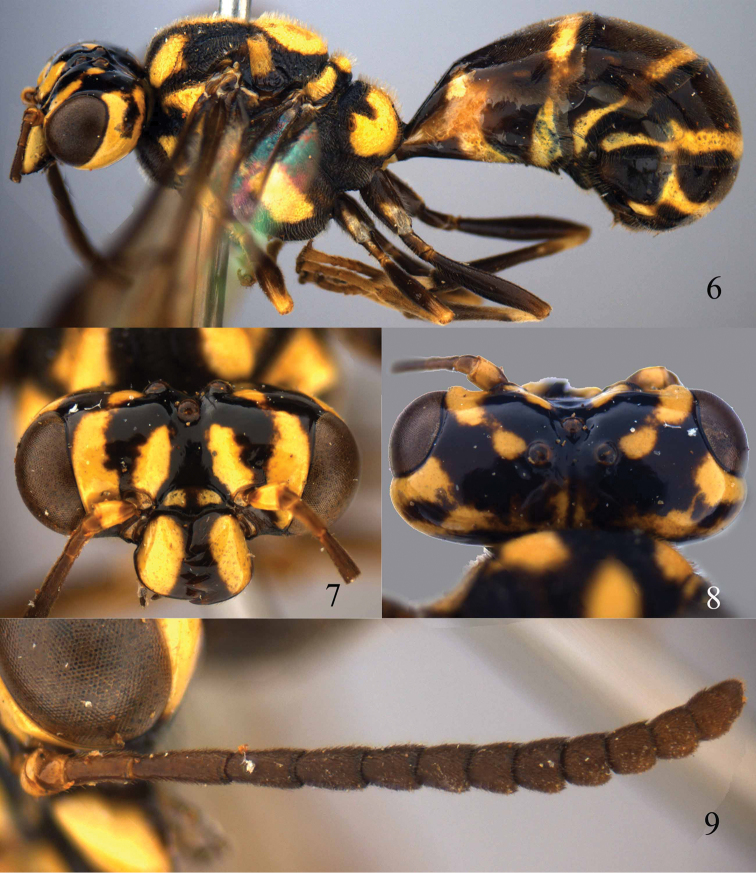
*Bakeronymus seidakka* Yamane & Terayama, 1983, paratype, female. **6** Habitus lateral **7** head anterior **8** head dorsal **9** antenna.

*Mesosoma*. Length of mesosoma 1.2 times its height (Fig. 13); mesopleuron densely and finely rugulose, anteriorly mixed with distinct rugae, dull; transverse mesopleural groove narrow and finely crenulate; notauli medium-sized and distinctly crenulate; mesoscutum largely reticulate-rugose and matt, contrasting with shiny head (Fig. 12); scutellar sulcus absent medially, narrow and crenulate laterally; scutellum reticulate-rugulose, convex and somewhat above level of mesoscutum; metanotum medially protruding, obtuse and medially smooth (Fig. 12); propodeum largely reticulate-rugose (Fig. 12).

**Figures 10–17.**
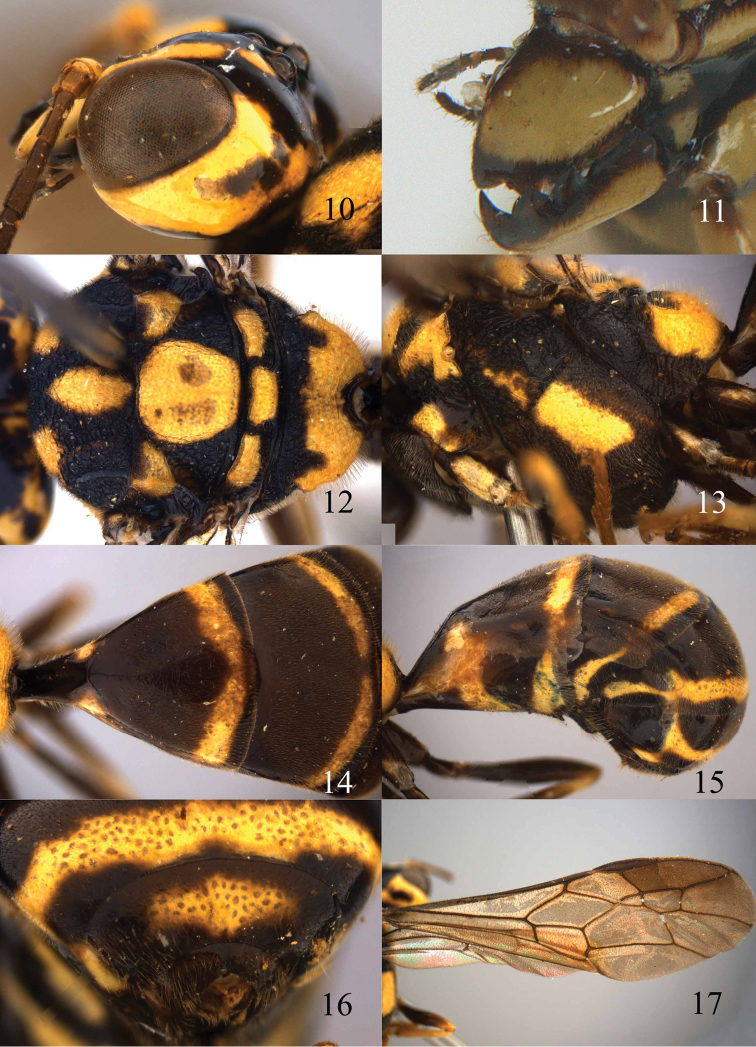
*Bakeronymus seidakka* Yamane & Terayama, 1983, paratype, female. **10** Head lateral **11** maxillary palp and mandible lateral **12** mesosoma dorsal **13** mesosoma lateral **14** first two tergites dorsal **15** metasoma lateral **16** apex of metasoma posterior **17** fore wing.

*Metasoma*. First tergite 0.9 times as long as apically wide, gradually narrowed basally, with large elliptical depression medially and concave apically; second tergite and sternite smooth; hypopygium incised apically.

*Colour*. Blackish brown or dark brown with rich yellow pattern (including mesopleuron; Figs 6, 9, 12, 13, 15); palpi and antenna (except largely yellow scapus) rather dark brown; patch on coxae, fore and middle trochanters partly, apex of fore and middle femora, fore tibia, base of middle and hind tibiae, all tarsi more or less pale yellow; pterostigma and marginal cell of fore wing and surroundings dark brown; remainder of wing membrane slightly infuscate.

*Male*. Unknown.

#### Biology.

Reared from nest of *Parapolybia varia* (Fabricius) (Polistinae: Vespidae) in Taiwan (Yamane and Terayama 1983). Collected in August.

#### Distribution.

China (Taiwan).

#### Notes.

Only one species is known from China: *Bakeronymus seidakka* Yamane & Terayama, 1983, re-instated. It was described as a subspecies of *Bakeronymus typicus* Rohwer, 1922, from the Philippines, but comparison with specimens of this species with a paratype of *Bakeronymus seidakka* shows considerable differences. For instance, the middle lobe of mesoscutum is entirely dark brown; the truncate apical part of the scutellum half as wide as the scutellum anteriorly; the fore wing has only two submarginal cells; the apical half of hind basitarsus is dark brown; the eyes are close to the mandibular condyli; the fourth antennal segment is distinctly widened apically and the propodeum has transverse rugae, without short interconnecting rugae. Therefore, we treat the taxon from Taiwan as an independent species, despite that some of the differences may be sex-related (e.g. the distance between the eye and the mandibular condylus).

### Bareogonalos

Schulz, 1907

http://species-id.net/wiki/Bareogonalos

Figs 18 –28

Bareogonalos Schulz, 1907: 18; Marshakov 1981: 104; Tsuneki 1991: 9; Weinstein and Austin 1991: 412; Carmean and Kimsey 1998: 60. Type species (by original designation): *Trigonalys canadensis* Harrington, 1896.
Nippogonalos Uchida, 1929: 79; Tsuneki 1991: 4; Lelej 1995: 12; Weinstein and Austin 1991: 412. Type species (by original designation): *Nippogonalos jezoensis* Uchida, 1929. Synonymized by Bischoff 1938.

#### Diagnosis.

Body length 8–13 mm; antenna with 18–23 segments; antenna of ♂ without tyloids; area above supra-antennal elevations flat, superficially finely rugulose and elevations small or nearly absent (Fig. 19); apical segment of labial palp widened and obtuse, more or less triangular (Fig. 22); mandibular condyli remain far removed from eyes (Fig. 22); vertex normal, at most with slight median depression dorsally (Fig. 20); head with long setae, transverse and flattened dorsally (Fig. 20); metanotum weakly convex and spaced rugose medially (in *Bareogonalos huisuni*: Fig. 23) or strongly lamelliform protuberant and densely sculptured; propodeum strongly areolate-rugose; hind trochanter simple, its dorsal triangular part not separated from basal part, but rarely partly differentiated (Tsuneki 1991); hind tarsus slightly or not modified; metasoma smooth and usually shiny or finely and rather densely punctate and rather dull; vein 1-SR of fore wing medium-sized to long (Fig. 28); protuberance of third sternite of ♀ similarly sized as protuberance of second sternite in lateral view or somewhat smaller (Fig. 26).

#### Biology.

Reared from *Vespa*, *Vespula*, *Dolichovespula* and *Provespa* spp. (Vespinae: Vespidae); the larva of at least one species has a final ectoparasitoid phase (Carmean 1991; Carmean and Kimsey 1998). Collected in August–October.

#### Notes.

As pointed out by Tsuneki (1991) *Nippogonalos* differs by having the head much narrower than the mesoscutum (nearly as wide as mesoscutum in typical *Bareogonalos*). The name *Nippogonalos* could be used as subgeneric name in *Bareogonalos* for the species with an enlarged mesosoma resulting in a comparatively small head.

#### Key to Old World species of *Bareogonalos* Schulz, 1907

**Table:** 

1	Metanotum with a thick and medially depressed protuberance and its setae long; head distinctly narrower than mesoscutum; anterior half of scutellum flat and at same level as mesoscutum; fourth and fifth tergites largely smooth and shiny; Japan and Far East Russia; [may occur in North China]	*Bareogonalos jezoensis* (Uchida, 1929)
–	Metanotum weakly convex, without protuberance and its setae medium-sized (Fig. 23); head nearly as wide as mesoscutum (Fig. 22); anterior half of scutellum distinctly convex and above level of mesoscutum; fourth and fifth tergites largely finely and rather densely punctate and rather dull (Fig. 27); Taiwan	*Bareogonalos huisuni* Yamane & Yamane, 1975

### Bareogonalos huisuni

Yamane & Yamane, 1975

http://species-id.net/wiki/Bareogonalos_huisuni

Figs 18 –28

Bareogonalos huisuni Yamane & Yamane, 1975: 456; Weinstein and Austin 1991: 412; Carmean and Kimsey 1998: 61.

#### Type material.

Paratypes, ♀ (RMNH) “[China:] Taiwan, Nantou, Kwantau Shih, 15.III.1973, S. Yamane”, or “6.VI.1973, S. Yamane”, “Paratype *Bareogonalos huisuni* Sk. et S. Yamane”. Holotype ♀ (HUS) and 3 ♀ topotypic paratypes (HUS) are not examined.

#### Diagnosis.

Head nearly as wide as mesoscutum (Fig. 23); anterior half of scutellum distinctly convex and above level of mesoscutum; metanotum weakly convex and without protuberance, its setae medium-sized (Fig. 23); protuberance of second sternite of ♀ densely setose and widely truncate medially (Fig. 26); metasoma largely finely and rather densely punctate and rather dull (Figs 25, 27).

#### Description.

Female paratype, length of body 11.0 mm (of fore wing 10.7 mm).

*Head*. Antenna with 20 segments, segments of apical half about as long as wide (Fig. 21); frons and vertex smooth and strongly shiny (Figs 19, 20), with long yellowish setae; head gradually narrowed behind eyes and nearly as wide as mesoscutum (Fig. 23); dorsal length of eye 1.7 times length of temple (Fig. 20); temple smooth and shiny; occipital carina narrow lamelliform medio-dorsally and with a short crenula; supra-antennal elevations hardly developed as a thin rim and smooth; clypeus moderately emarginate and thick medio-ventrally.

*Mesosoma*. Length of mesosoma 1.3 times its height (Fig. 24); mesopleuron below transverse mesopleural groove largely smooth and weakly shiny, above groove coarsely irregularly reticulate; transverse mesopleural groove wide, deep and coarsely crenulate; notauli medium-sized and distinctly crenulate; mesoscutum largely irregularly rugose and matt, contrasting with shiny head (Fig. 23); scutellar sulcus present medially and narrowed, laterally wide and coarsely crenulate; scutellum reticulate-rugose, convex and anteriorly above level of mesoscutum, posteriorly flattened and finely sculptured; metanotum flattened and medially with some rugae (Fig. 23); propodeum largely irregularly reticulate-rugose (Fig. 23).

**Figures 18–21.**
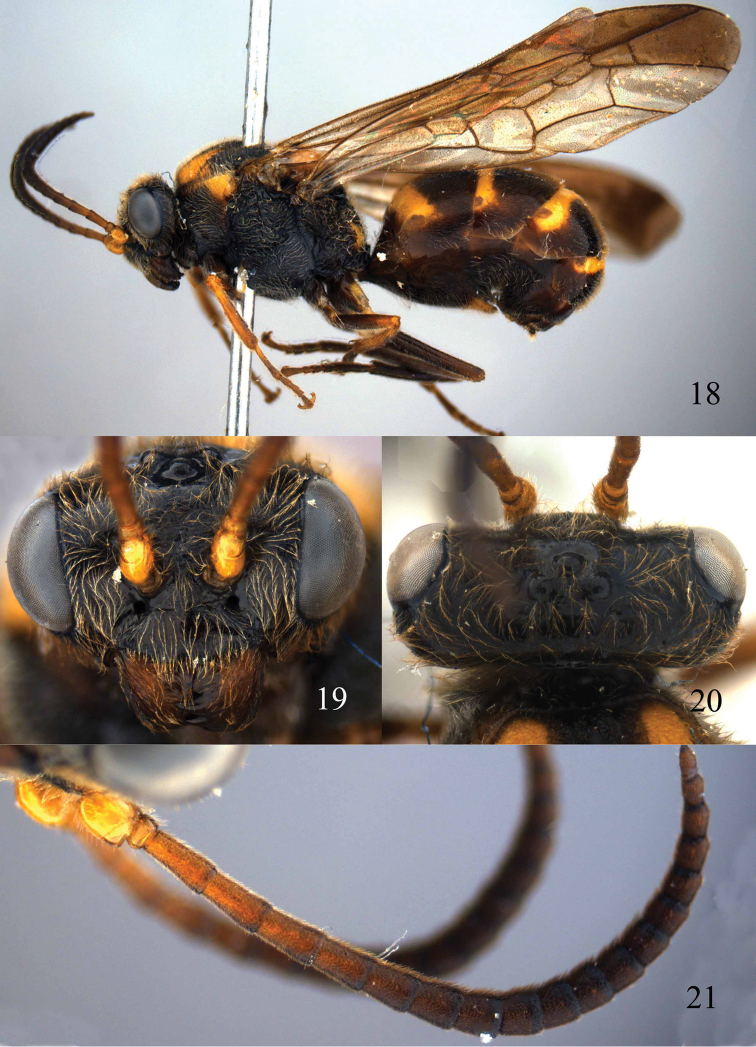
*Bareogonalos huisuni* Yamane & Yamane, 1975, paratype, female. **18** Habitus lateral **19** head anterior **20** head dorsal **21** antennae.

**Figures 22–28.**
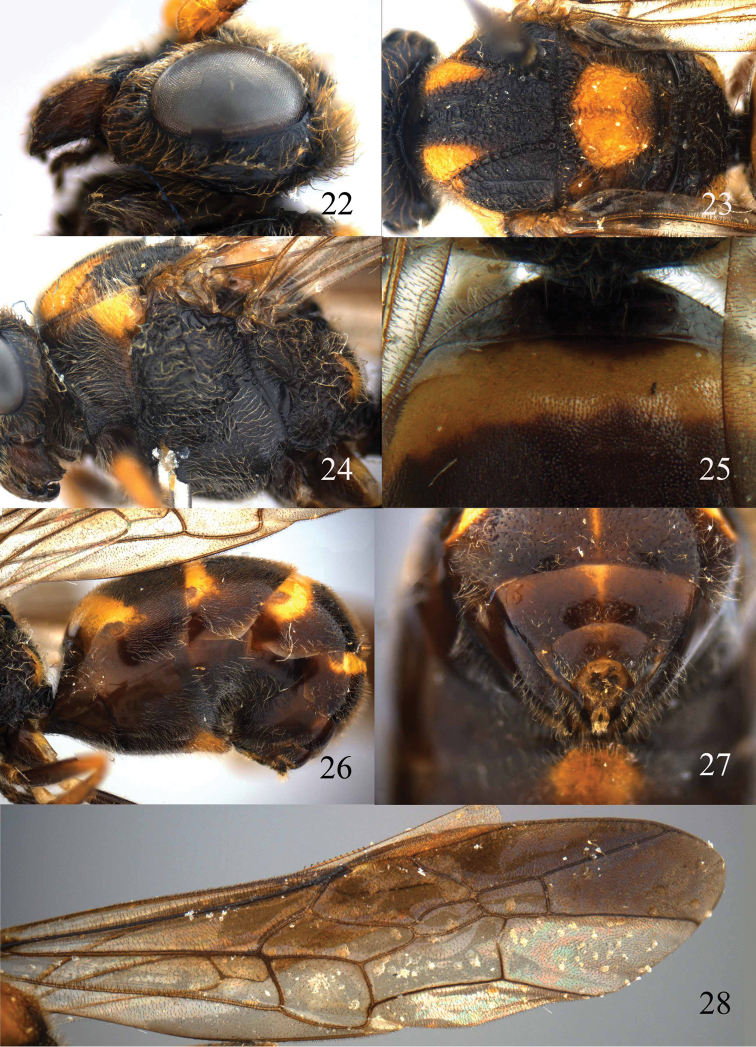
*Bareogonalos huisuni* Yamane & Yamane, 1975, paratype, female. **22** Head lateral **23** mesosoma dorsal **24** mesosoma lateral **25** first tergite dorsal **26** metasoma lateral **27** apex of metasoma posterior **28** fore wing.

*Metasoma*. First tergite 0.3 times as long as apically wide, gradually narrowed basally, with wide oval depression medially and straight apically; second tergite densely finely punctulate and slightly shiny; second sternite smooth and shiny, its medio-apical protuberance densely setose and widely truncate medially (Fig. 26), protuberance of third sternite similar to it but smaller (Fig. 26); hypopygium truncate apically.

*Colour*. Black or dark brown (including palpi) with yellow pattern (including mesopleuron; Figs 18, 23, 24, 26); mandible largely chestnut brown; scapus and pedicellus yellow, third and following segments brown, but apical half of antenna dark brown; pronotum dorso-apically and dorsally, pair of wide patches on mesoscutum anteriorly, scutellum (except posteriorly), axilla partly, lateral patch of propodeum, basal 0.4 of second tergite (except small anterior patch and laterally), anterior quarter of 3^rd^–5^th^ tergites (except laterally) and narrow median stripe of 4^th^–7^th^ tergites, apex of fore and middle femora, base of fore and middle tibiae, yellow; hind coxa dorso-apically, inner side of middle trochanter and hind trochanter and trochantellus pale yellow; pterostigma and veins 1-SR, 1-M and more distal veins of fore wing light brown; membrane of anterior half of fore wing rather dark brown, remainder of wing membrane slightly infuscate.

*Male*. Unknown (“males” reported by Yamane and Yamane (1975) concerns females).

#### Biology.

Reared from *Vespula flaviceps karenkona* Sonan (Vespinae: Vespidae). Collected in March.

#### Distribution.

China (Taiwan).

#### Notes.

The illustrated paratype is a female and not a male as reported by Yamane and Yamane (1975).

### Jezonogonalos

Tsuneki, 1991 re-instated

http://species-id.net/wiki/Jezonogonalos

Figs 29 [Fig F8] [Fig F9] [Fig F10] [Fig F11] [Fig F12] [Fig F13] [Fig F14] [Fig F15] [Fig F16] [Fig F17] [Fig F18] [Fig F19] [Fig F20] –107

Jezonogonalos Tsuneki, 1991: 32, 2003: 4; Carmean and Kimsey 1998: 70. Type species (by original designation): *Jezonogonalos marujamanae* Tsuneki, 1991 [= *Jezonogonalos marujamae* Tsuneki, 1991]. Synonymized with *Pseudogonalos* Schulz, 1906, by Lelej 1995.

#### Diagnosis.

Length of body 6.6–12.0 mm; antenna black and with 23–27 segments; area above supra-antennal elevations flat, more or less punctate, without protuberance between elevations and inner side of supra-antennal elevations flat, smooth and black (Figs 40, 55, 66, 75, 87, 98); tyloids on 11^th^–14^th^ antennal segments of male short and nearly circular or elliptical (Figs 51, 56, 77, 100; unknown of *Jezonogonalos laeviceps* and *Jezonogonalos nigrata* sp. n.); occipital carina widened medio-dorsally; apical segment of labial palp widened and obtuse, more or less triangular; vertex normal, at most with slight median depression dorsally (Fig. 75); mandibles wide in anterior view and sublaterally attached to head (Fig. 39); metanotum strongly convex and finely sculptured medially (Fig. 59); anterior propodeal sulcus crenulate and medially widened (Fig. 59); posterior propodeal carina curved and distinctly protruding and more or less separated from foramen medio-dorsally (Figs 44, 59, 69, 80, 93, 103); fore wing with large dark patch below pterostigma; vein 1-SR of fore wing long (Figs 42, 57, 68, 78, 89, 101); hind trochanter black or ivory; hind tarsus slightly or not modified; second and third sternites of ♀ flat and moderately sclerotized and no protuberances (Figs 48, 72, 95, 107); body without pale pattern, at most malar space and margins of basal metasomal sternites and tergites narrowly ivory, remainder black.

**Figures 29–33.**
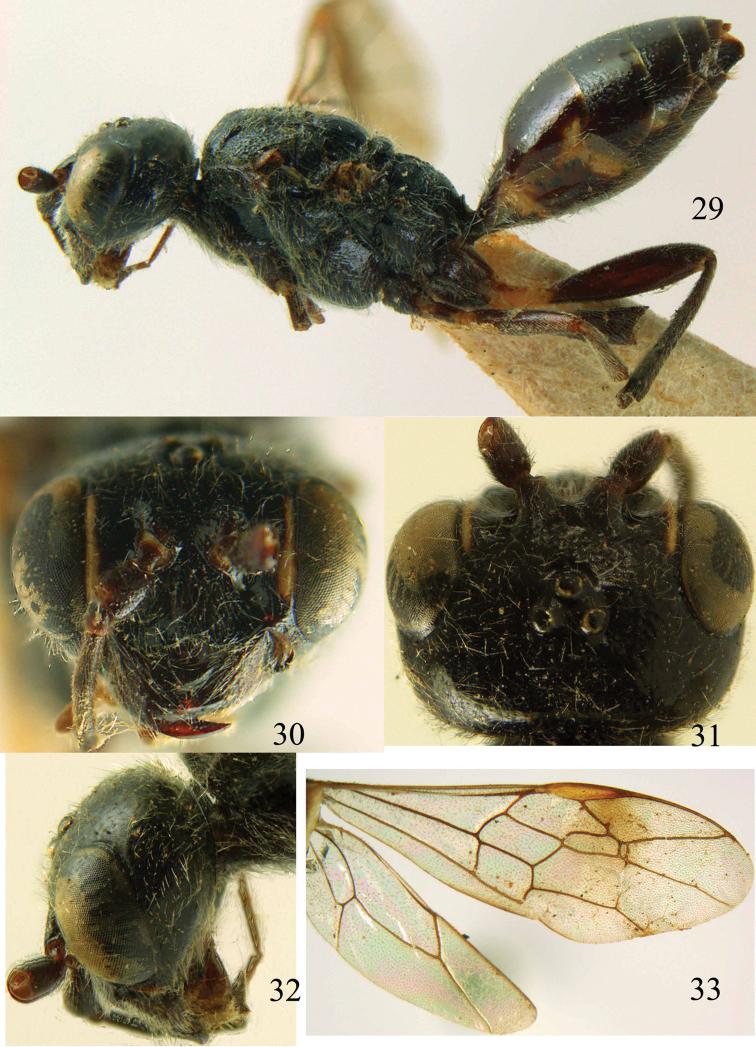
*Jezonogonalos marujamae* Tsuneki, 1991, holotype, female. **29** Habitus lateral **30** head anterior **31** head dorsal **32** head lateral **33** fore and hind wings.

**Figures 34–37. F8:**
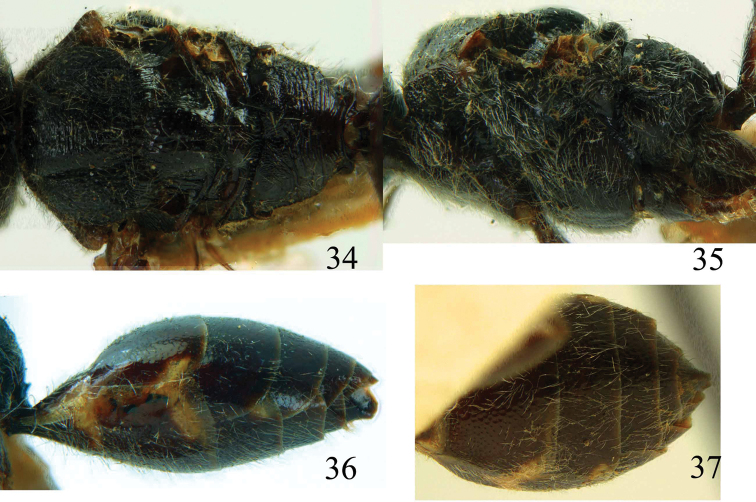
*Jezonogonalos marujamae* Tsuneki, 1991, holotype, female. **34** Mesosoma dorsal **35** mesosoma lateral **36** metasoma lateral **37** metasoma ventral.

#### Biology.

Unknown.

#### Notes.

*Jezonogonalos* Tsuneki was treated as valid genus in the revision by Carmean and Kimsey (1998) and synonymized with *Pseudogonalos* Schulz by Lelej (1995). We agree with Carmean and Kimsey (1998) that the shape of the supra-antennal elevations is different enough to retain both genera as valid.

#### Key to species of *Jezonogonalos* Tsuneki, 1991

**Table:** 

1	First metasomal tergite about as long as its apical width (Fig. 61); supra-antennal elevations only apically ivory (Fig. 55); third sternite 0.6–0.7 times as long as second sternite (Fig. 63); [metasoma of ♂ entirely black, slender (Fig. 61); occipital carina extensively crenulate dorsally and widened (Fig. 55); third submarginal cell of fore wing about 0.4 times as long as second submarginal cell]	*Jezonogonalos jiangliae* sp. n.
–	First tergite 0.6–0.8 times as long as its apical width (Figs 46, 71, 82, 93, 105); at least apical half of supra-antennal elevations yellow (Figs 40, 66, 75, 87, 98) or entirely black; third sternite 0.1–0.6 times as long as second sternite (Figs 48, 72, 84, 95, 107)	2
2	Fore wing subhyaline (Fig. 42); metasoma of ♂ with narrow yellowish brown apical band at apex of all tergites (Fig. 52); third sternite about 0.1 times as long as second sternite (Fig. 48); [occipital carina only medio-dorsally with short carina (Fig. 40)]	*Jezonogonalos elliptifera* sp. n.
–	Fore wing with more or less conspicuous dark brown patch below pterostigma (Figs 68, 78, 89, 101); metasoma of ♂ entirely black dorsally or nearly so (Figs 82, 105); third sternite 0.3–0.6 times as long as second sternite (Figs 72, 84, 95, 107)	3
3	Occipital carina extensively crenulate dorsally (Figs 75, 87); third sternite 0.3–0.4 times as long as second sternite (Figs 84, 95)	4
–	Occipital carina only with short carina medio-dorsally (Figs 66, 98); third sternite 0.4–0.6 times as long as second sternite (Figs 36, 72, 107)	5
4	Supra-antennal elevations largely yellow (Fig. 75); frons and vertex sparsely and finely punctate (Figs 74, 75); occipital carina moderately wide dorsally (Fig. 75)	*Jezonogonalos luteata* sp. n.
–	Supra-antennal elevations black (Fig. 87); frons and vertex densely and coarsely punctate (Figs 86, 87); occipital carina very wide dorsally (Fig. 87)	*Jezonogonalos nigrata* sp. n.
5	Propodeum finely transversely striate medially (Fig. 34); second submarginal cell of fore wing small and parallel-sided, laterally about twice higher than wide anteriorly (Fig. 33); Japan, not found yet in China	*Jezonogonalos marujamae* Tsuneki, 1991
–	Propodeum with 1-2 transverse carinae medially (Figs 69, 103); second submarginal cell of fore wing either small and narrowed anteriorly (Fig. 68) or medium-sized, parallel-sided, laterally about as high as wide anteriorly (Fig. 101)	6
6	Middle lobe of mesoscutum densely transversely striate (Fig. 103); second submarginal cell of fore wing medium-sized, parallel-sided and laterally about as high as wide anteriorly (Fig. 101); apical 0.4 of first metasomal tergite nearly completely dark brown (Fig. 105)	*Jezonogonalos satoi* (Tsuneki, 1991), comb. n., re-instated
–	Middle lobe of mesoscutum largely smooth (Fig. 69); second submarginal cell of fore wing small and narrowed anteriorly (Fig. 68); apical 0.4 of first tergite largely ivory except medially (Fig. 71)	*Jezonogonalos laeviceps* (Tsuneki, 1991), comb. n., re-instated

### Jezonogonalos elliptifera sp. n.

http://zoobank.org/B349BD3D-6A0D-449E-BF22-B76DC9D810CE

http://species-id.net/wiki/Jezonogonalos_elliptifera

Figs 38 – 52

#### Type material.

Holotype, ♀ (IZCAS) “[China:] Sichuan, Mt. Emei, Jiulaodong, 1800–1900 m, 10.VIII.1957, Ke-ren Huang, IOZ(E)1495441”. Paratypes: 1 ♀ + 3 ♂ (IZCAS) “[China:] Sichuan, Mt. Emei, Jiulaodong, 1800–1900 m, 26.VII.1957, Ke-ren Huang, IOZ(E)1495240, IOZ(E)1495242; id., but 7.VII.1957, IOZ(E)1495244; id., but 9.VII.1957, IOZ(E)1495243”; 1 ♂ (IZCAS) “[China:] Sichuan, Mt. Emei, Chudian, 1783 m, 20.VI.1957, Fu-xing Zhu, IOZ(E)1495241”; 1 ♂ (ZJUH) “[China:] Sichuan, Hongya County, Mt. Wawu, 6.VII.2009, Jiang-li Tan, 200906392”.

#### Diagnosis.

Occipital carina widened medio-dorsally, with short carinae (Fig. 40); outer side of supra-antennal elevations subvertical and elevations about 0.7 times as long as scapus (Figs 40, 43); head dorsally coarsely reticulate-punctate (Fig. 40); apical half of supra-antennal elevations yellowish brown (Fig. 40); tyloids of male antenna elliptical and short (Fig. 51); basal half of metasoma largely smooth, apical half mainly punctate (Fig. 46); metasoma largely black with narrow orange-brown apical band at apex of all tergites (Figs 46, 47); first tergite about 0.7 times as long as its apical width (Fig. 46); third sternite about 0.2 times as long as second sternite (Fig. 48).

**Figures 38–40. F9:**
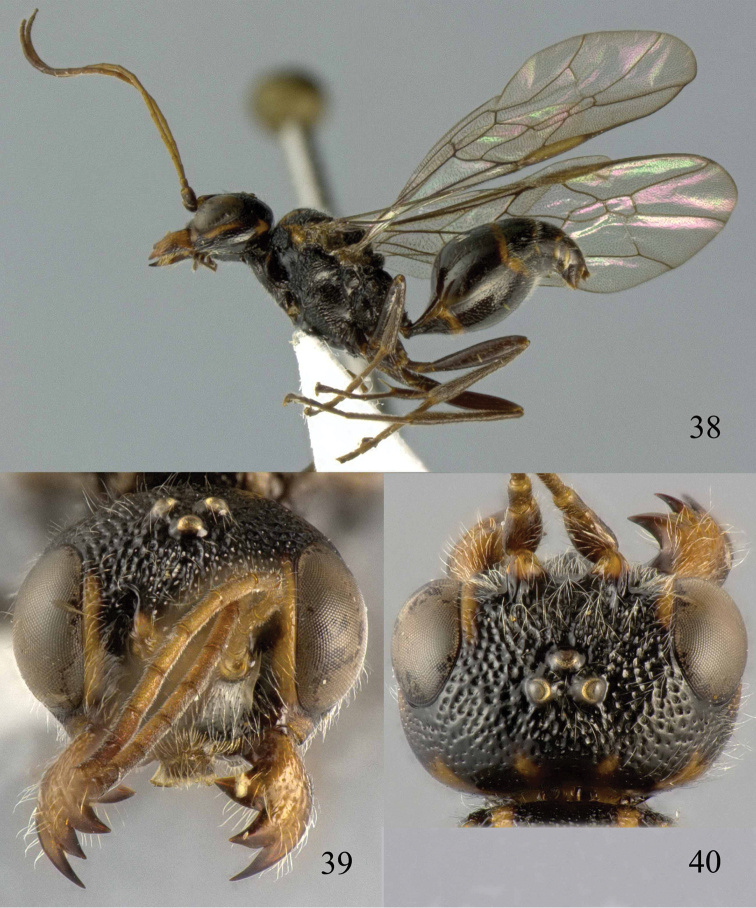
*Jezonogonalos elliptifera* sp. n., holotype, female. **38** Habitus lateral **39** head anterior **40** head dorsal.

**Figures 41–48. F10:**
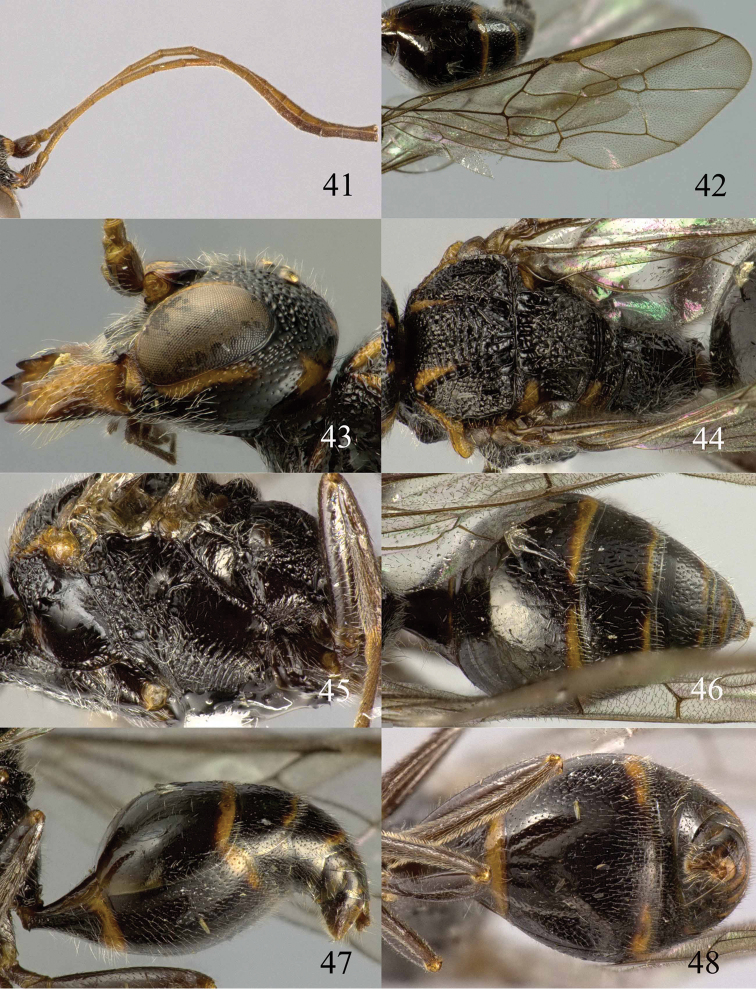
*Jezonogonalos elliptifera* sp. n., holotype, female. **41** Antennae **42** fore wing **43** head lateral **44** mesosoma dorsal **45** mesosoma lateral **46** metasoma dorsal **47** metasoma lateral **48** metasoma ventral.

**Figures 49–52. F11:**
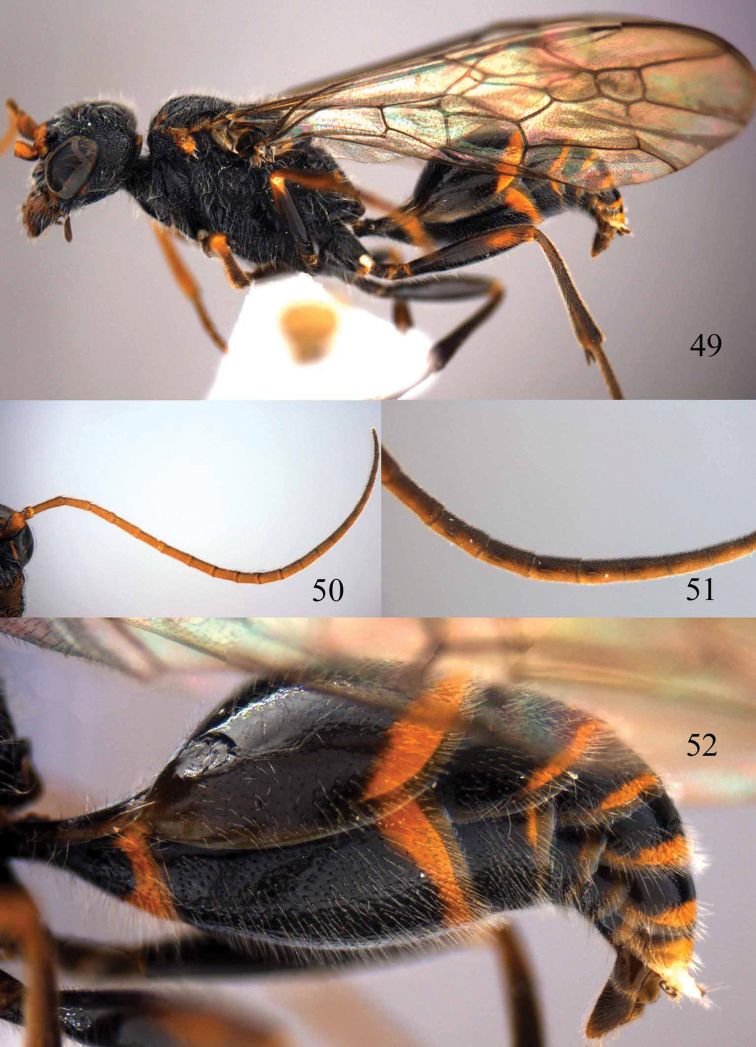
*Jezonogonalos elliptifera* sp. n., paratype, male. **49** Habitus lateral **50** antenna **51** tyloids on 11^th^–15^th^ segments of antenna **52** metasoma lateral.

#### Description.

Holotype, female, length of body 6.6 mm (of fore wing 5.8 mm).

*Head*. Antenna with 23 segments; frons coarsely reticulate-punctate (Fig. 39), with rather long setae; vertex coarsely reticulate-punctate behind posterior ocellus, spaced punctate (interspaces much wider than width of punctures) posteriorly (Fig. 40); temple largely smooth with sparse fine punctures (Fig. 43); head gradually narrowed behind eyes, eye in dorsal view 1.5 times as long as temple (Fig. 40); occipital carina strongly widened and lamelliform medio-dorsally, with many short carinae; supra-antennal elevations strongly enlarged (about 0.7 times as long as scapus), outer side subvertical and largely smooth except for sparse punctures; clypeus slightly concave and thick medio-ventrally.

*Mesosoma*. Length of mesosoma 1.6 times its height (Fig. 45); mesopleuron antero-dorsally irregularly rugose, dorso-posteriorly smooth and shiny and antero-ventrally obliquely rugose; notauli wide, deep and largely crenulate; middle lobe of mesoscutum irregularly rugose, lateral lobes mainly finely rugose (Fig. 44); scutellar sulcus complete, very wide and crenulate; scutellum densely reticulate-rugose, convex posteriorly and flattened anteriorly and medially; metanotum medially strongly lobe-shaped protruding and largely rugose (Fig. 44); propodeum coarsely rugose (Fig. 44); posterior propodeal carina thick lamelliform (foramen about twice as wide as high medially).

*Wings*. Fore wing: length of vein 1-M 1.6 times as long as vein 1-SR (Fig. 42).

*Metasoma*. First tergite 0.7 times as long as apically wide, smooth and with distinct elliptical depression medially (Fig. 46); second tergite largely smooth with sparse fine punctures posteriorly, other tergites superficial coriaceous anteriorly, densely finely punctate posteriorly (Fig. 46); sternites densely finely punctate; second sternite weakly curved in lateral view; third sternite about 0.1 times as long as second sternite (Fig. 48); hypopygium triangular in ventral view (Fig. 48).

*Colour*. Black; inner orbita narrowly orange-brown; apical half of supra-antennal elevations yellowish brown, clypeus with a narrow transverse orange-brown patch; minute patch of outer orbita, vertex posteriorly, occipital carina medially and mandible largely yellowish brown (Figs 39, 40, 43); mesosoma laterally black except for brown dorsal rim of pronotal side; middle lobe of mesoscutum with pair of narrow brown patches anteriorly; axilla with medium-sized brown patch; metanotum with pair of small lateral yellowish brown patches; metasoma dorsally black, with narrow orange-brown apical band at apex of all tergites and first two sternites (Figs 46, 47, 48); palpi dark brown; antenna brown, becoming darker apically; legs dark brown to black; pterostigma brown, remainder of wing membrane subhyaline.

*Variation*. Length of body 6.6–9.8 mm, of fore wing 5.8–8.7 mm; propodeum with pair of small brown spots; vertex coarsely reticulate-punctate; temple rugulose-punctate; second tergite with dense fine punctures posteriorly; length of vein 1-M of fore wing 1.5–1.6 times as long as vein 1-SR.

*Male*. Length of body 8.3–9.1 mm, of fore wing 6.8–7.8 mm; antenna with 23 segments, tyloids elliptical, 0.25 times as long as segment on 11^th^–13^th^ segments and nearly circular on 14^th^ and 15^th^ segments; in general males are darker than females: vertex and outer orbita entirely black; mesosoma with or without brown patches; first and third tergites sometimes without narrow orange-brown apical band; genitalia extruded (Fig. 52).

#### Biology.

Collected in June–August.

#### Distribution.

China (Sichuan).

#### Etymology.

Named after the elliptical tyloids of the male antenna and “*fera*” (Latin suffix for “carry, have”).

### Jezonogonalos jiangliae sp. n.

http://zoobank.org/5A623FD0-2306-4C70-AB7E-4CF76FA7940F

http://species-id.net/wiki/Jezonogonalos_jiangliae

Figs 53 –63

#### Type material.

Holotype, ♂ (ZJUH) “[China:] Tibet, Ranwu–Chayu, 22.VI.2009, Jiang-li Tan, 200906372”.

#### Diagnosis.

Supra-antennal elevations 0.5–0.7 times as long as scapus and outer side of elevations often subvertical, smooth, strongly shiny and only apically ivory (Fig. 55); tyloids of male antenna nearly circular or short elliptical (Fig. 56); occipital carina extensively crenulate dorsally and widened (Fig. 55); third submarginal cell of fore wing about 0.4 times as long as second submarginal cell; first discal cell of fore wing less elongate (Fig. 57); first metasomal tergite about as long as its apical width (Fig. 61); third sternite 0.6–0.7 times as long as second sternite (Fig. 61); metasoma of male largely smooth and entirely black, slender (Fig. 61).

**Figures 53–55. F12:**
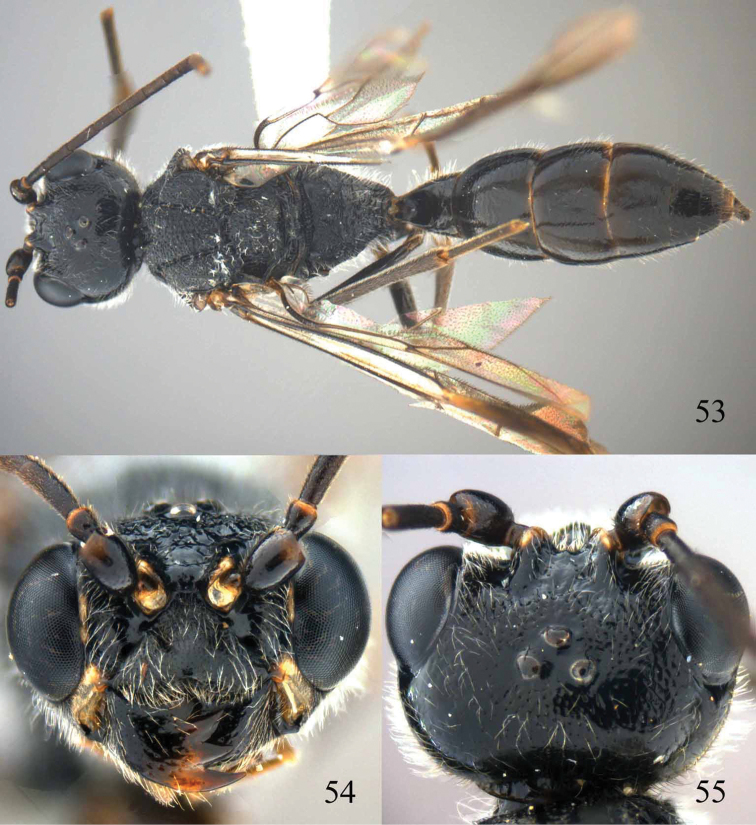
*Jezonogonalos jiangliae* sp. n., holotype, male. **53** Habitus dorsal **54** head anterior **55** head dorsal.

**Figures 56–63. F13:**
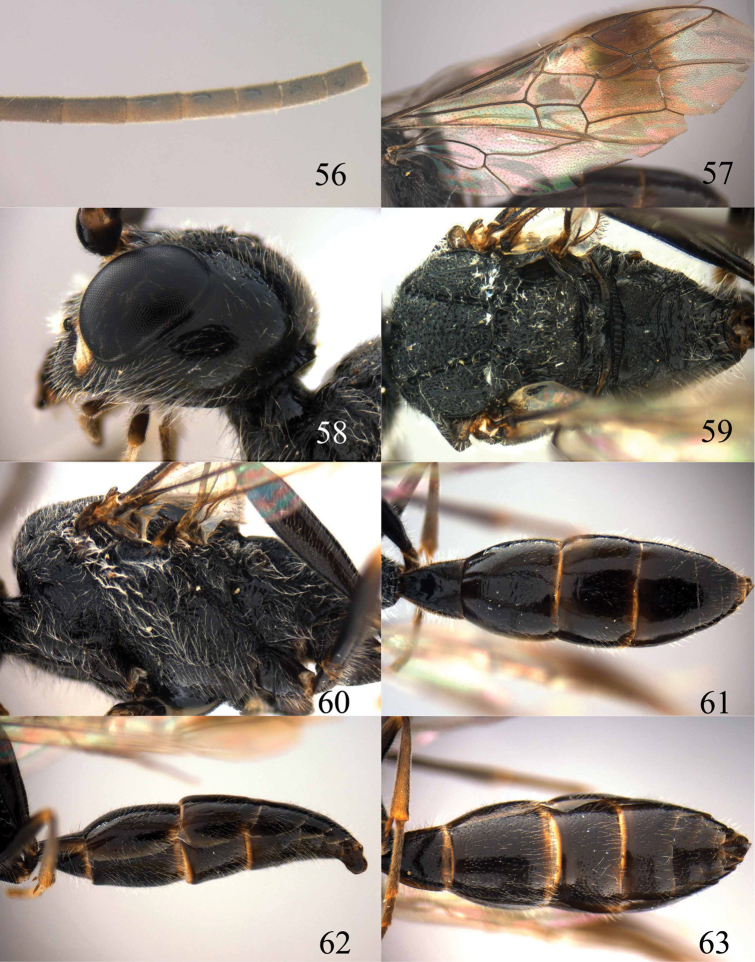
*Jezonogonalos jiangliae* sp. n., holotype, male. **56** Tyloids on 11^th^–15^th^ segments of antenna **57** fore and hind wings **58** head lateral **59** mesosoma dorsal **60** mesosoma lateral **61** metasoma dorsal **62** metasoma lateral **63** metasoma ventral.

#### Description.

Holotype, male, length of body 9.3 mm (of fore wing 7.2 mm).

*Head*. Antenna incomplete, tyloids nearly circular, 0.1 times as long as segment on 10^th^ segment and 0.2 times as long as segments on 11^th^ –15^th^ segments (Fig. 56); frons rugose (Fig. 54); vertex and temple largely smooth and shiny with sparse and fine punctures (Figs 55, 58); head gradually narrowed behind eyes, eye in dorsal view 0.9 times as long as temple (Fig. 55); occipital carina strongly widened and lamelliform, extensively crenulate dorsally; supra-antennal elevations strongly enlarged (about 0.7 times as long as scapus), outer side subvertical and largely smooth except for sparse punctures; clypeus slightly concave and thick medio-ventrally.

*Mesosoma*. Length of mesosoma 1.6 times its height (Fig. 60); mesopleuron transversely reticulate-rugose anteriorly and smooth posteriorly (Fig. 60); transverse mesopleural groove moderately wide, shallow but distinctly crenulate; notauli moderately wide, deep and coarsely crenulate; middle lobe of mesoscutum somewhat transversely rugose, lateral lobes of mesoscutum mainly finely rugose with a shallow furrow medially (Fig. 59); scutellar sulcus wide, both medially and laterally and coarsely crenulate; scutellum densely and coarsely rugose, slightly convex medially and anteriorly near level of mesoscutum; metanotum medially protruding, obtuse and densely and finely punctate (Fig. 59); propodeum obliquely rugulose antero-laterally, transversely striate medially and irregular rugose posteriorly (Fig. 59); posterior propodeal carina thick lamelliform, foramen medially 0.7 times higher than wide basally.

*Wings*. Fore wing: length of vein 1-M 1.6 times as long as vein 1-SR (Fig. 57).

*Metasoma*. First tergite 1.1 times as long as apically wide, smooth and with distinct elliptical depression antero-medially, slightly convex posteriorly (Fig. 61); second–fifth tergites and all sternites largely smooth except for sparse superficial punctures (Fig. 61); second sternite rather flat; third sternite about 0.7 times as long as second sternite (Fig. 63); genitalia extruded (Fig. 62).

*Colour*. Black; inner orbita narrowly, apex of supra-antennal elevation and malar space ivory (Fig. 54); mandibular teeth, palpi and tegulae dark brown; tibiae and tarsi rather brownish; pterostigma and apical half of first submarginal cell to anterior half of marginal cell of fore wing and area below that dark brown, remainder of wing membrane subhyaline.

*Female*. Unknown.

#### Biology.

Unknown. Collected in June.

#### Distribution.

China (Tibet).

#### Etymology.

Named after its collector, Dr Jiang-li Tan from Northwest University at Xi’an.

### Jezonogonalos laeviceps

(Tsuneki, 1991) comb. n., re-instated

http://species-id.net/wiki/Jezonogonalos_laeviceps

Figs 64 –72

Taiwanogonalos laeviceps Tsuneki, 1991: 40. Synonymized by Carmean and Kimsey 1998 with *Taeniogonalos maga* Teranishi, 1929, and is here re-instated as valid species.

#### Type material.

Holotype, ♀ (OMNH) “[China:] Formosa, Arisan, 2-23.X.1918, J Sonan, M. Yoshino”, “*Taiwanogonalos laeviceps* Tsuneki, ♂ [*sic*!], holotype”.

#### Diagnosis.

Occipital carina wide lamelliform and only with a short carina medio-dorsally (Fig. 66); supra-antennal elevations entirely black; middle lobe of mesoscutum largely smooth (Fig. 69); propodeum with 1-2 transverse rugae medially (Fig. 69); second submarginal cell of fore wing small and narrowed anteriorly (Fig. 68); fore wing with more or less conspicuous dark brown patch below pterostigma (Fig. 68); metasoma of ♂ entirely black dorsally or nearly so, but apical 0.4 of first tergite largely ivory except medially (Fig. 71); first tergite 0.6–0.8 times as long as its apical width (Fig. 71); third sternite about 0.5 times as long as second sternite (Fig. 72).

**Figures 64–68. F14:**
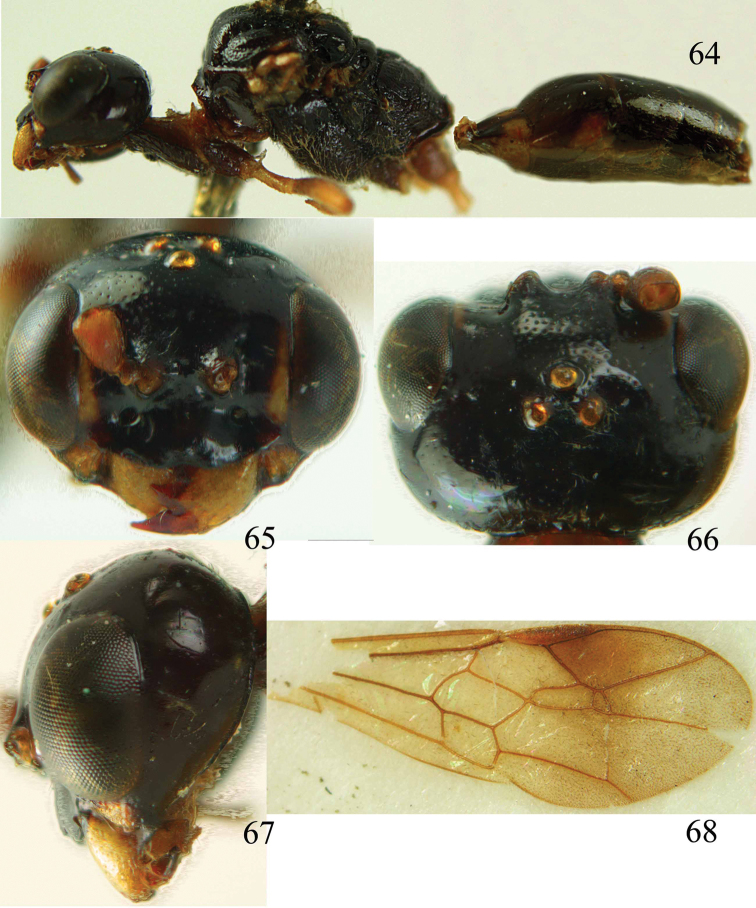
*Jezonogonalos laeviceps* (Tsuneki, 1991), holotype, female. **64** Habitus lateral **65** head anterior **66** head dorsal **67** head lateral **68** fore wing.

**Figures 69–72. F15:**
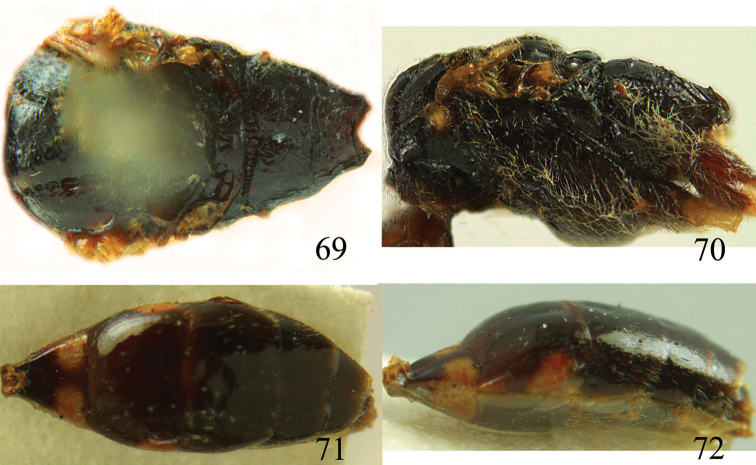
*Jezonogonalos laeviceps* (Tsuneki, 1991), holotype, female. **69** Mesosoma dorsal **70** mesosoma lateral **71** metasoma dorsal **72** metasoma lateral.

#### Description.

Holotype (mutilated as indicated in original description), female (not male as indicated in original description!), length of body 6.5 mm (of fore wing about 5.0 mm).

*Head*. Antenna unknown [male antenna with 21 segments (including scapus) glued on card with longitudinal tyloids on 10^th^–15^th^ segments is most likely of holotype of *Taeniogonalos alticola*]; frons spaced punctulate; vertex largely smooth, sparsely punctulate and strongly shiny (Fig. 66), with rather short setae; temple largely smooth (Fig. 67); head gradually narrowed behind eyes, eye in dorsal view as long as temple (Fig. 66); occipital carina wide lamelliform medio-dorsally with one short carina and few short sublateral carinae; supra-antennal elevations strongly enlarged (about 0.6 times as long as scapus), outer side subvertical and largely smooth except for sparse punctures, latero-basally with pit-shaped depression (Fig. 66); clypeus slightly concave and thick medio-ventrally.

*Mesosoma*. Length of mesosoma 1.6 times its height (Fig. 70); mesopleuron below transverse mesopleural groove largely smooth but superficially rugulose anteriorly, above groove smooth but rugose anteriorly and no vertical groove (Fig. 70); transverse mesopleural groove wide, deep and widely crenulate; notauli narrow anteriorly, wide posteriorly and coarsely crenulate; mesoscutum largely smooth, spaced finely punctate and shiny, with some rugae posteriorly (Fig. 69); scutellar sulcus wide, both medially and laterally and coarsely crenulate; scutellum largely smooth (except some punctulation and medio-posteriorly coarsely crenulate) and shiny, longitudinally depressed medially and anteriorly above level of mesoscutum; metanotum medially hardly protruding, obtuse and smooth (Fig. 69); propodeum rugulose anteriorly (and sulcus medially widely crenulate and depressed), with smooth patches antero-laterally, with few transverse rugae medially (Fig. 69); posterior propodeal carina thick lamelliform and curved, foramen medially 0.6 times higher than wide basally.

*Wings*. Fore wing: length of vein 1-M 2.2 times as long as vein 1-SR and distinctly curved; veins r and 2-SR long (Fig. 68); second submarginal cell of fore wing small and narrowed anteriorly.

*Metasoma*. First tergite 0.7 times as long as apically wide, smooth and with indistinct depression basally (Fig. 71); second and following tergites smooth and strongly shiny (Fig. 71); sternites mainly smooth; second sternite weakly curved in lateral view; third sternite about half as long as second sternite (Fig. 72).

*Colour*. Black or dark brown; mandible (except apically), malar space largely, inner orbita narrowly, pronotum latero-dorsally, minute lateral patch of scutellum, posterior half of first segment largely (except medially), large patch laterally of second tergite, trochanters, trochantelli and base of femora ivory; palpi and tegulae yellowish brown; remainder of legs more or less brown or dark brown; pterostigma (but basally yellowish), veins and marginal cell (except apically) dark brown or brown; remainder of wing membrane subhyaline.

*Male*. Unknown.

#### Biology.

Unknown. Collected in October.

#### Distribution.

China (Taiwan).

### Jezonogonalos luteata sp. n.

http://zoobank.org/64673A1B-9FE3-41E4-8C76-0FCB38193D77

http://species-id.net/wiki/Jezonogonalos_luteata

Figs 73 –84

#### Type material.

Holotype, ♂ (IZCAS) “[China:] Sichuan, Mt. Emei, Xixiangchi, 1800–2000 m, 17.VIII.1957, Ke-ren Huang, IOZ(E)1495440”.

#### Diagnosis.

Occipital carina widened medio-dorsally, with short carinae (Fig. 75); outer side of supra-antennal elevations subvertical and elevations about 0.7 times as long as scapus (Figs 75, 79); head dorsally coarsely reticulate-punctate (Fig. 75); apical half of supra-antennal elevations yellowish brown (Fig. 75); tyloids of male antenna elliptical and short (Fig. 77); basal half of metasoma largely smooth, apical half mainly punctate (Fig. 82); metasoma largely black pair of small lateral patches on first and second tergites, apical margin of first and second sternites (Figs 83, 84); first tergite about 0.7 times as long as its apical width (Fig. 84); second sternite simple (Fig. 84); third sternite about 0.2 times as long as second sternite (Fig. 84).

**Figures 73–76. F16:**
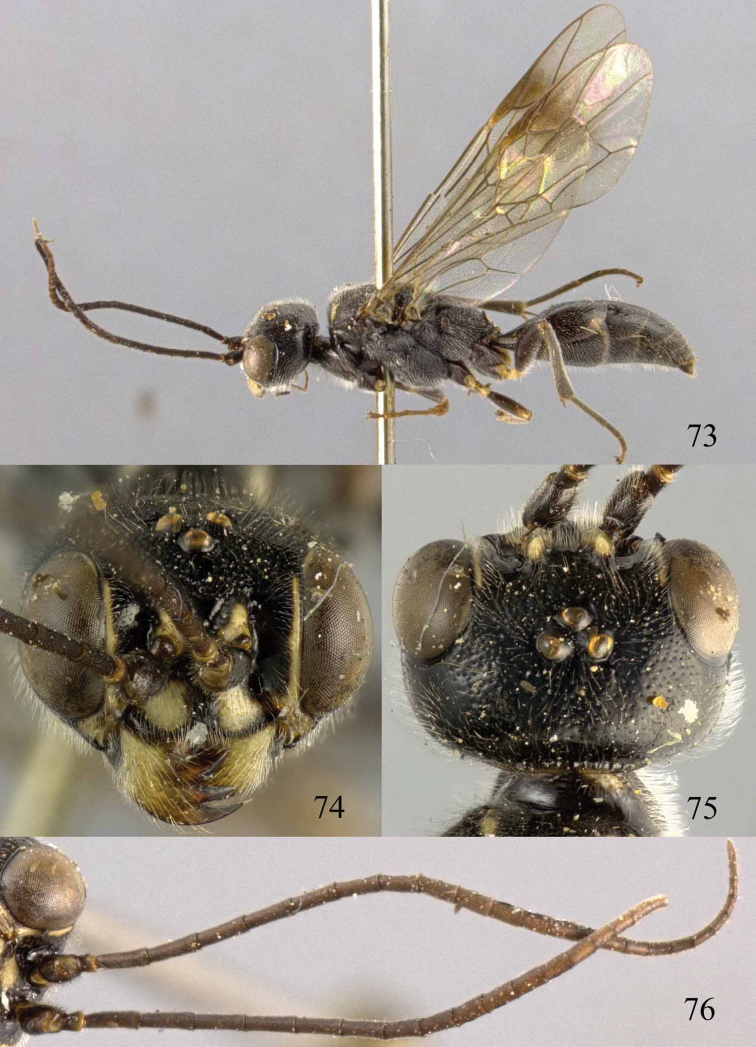
*Jezonogonalos luteata* sp. n., holotype, male. **73** Habitus lateral **74** head anterior **75** head dorsal **76** antennae.

**Figures 77–84. F17:**
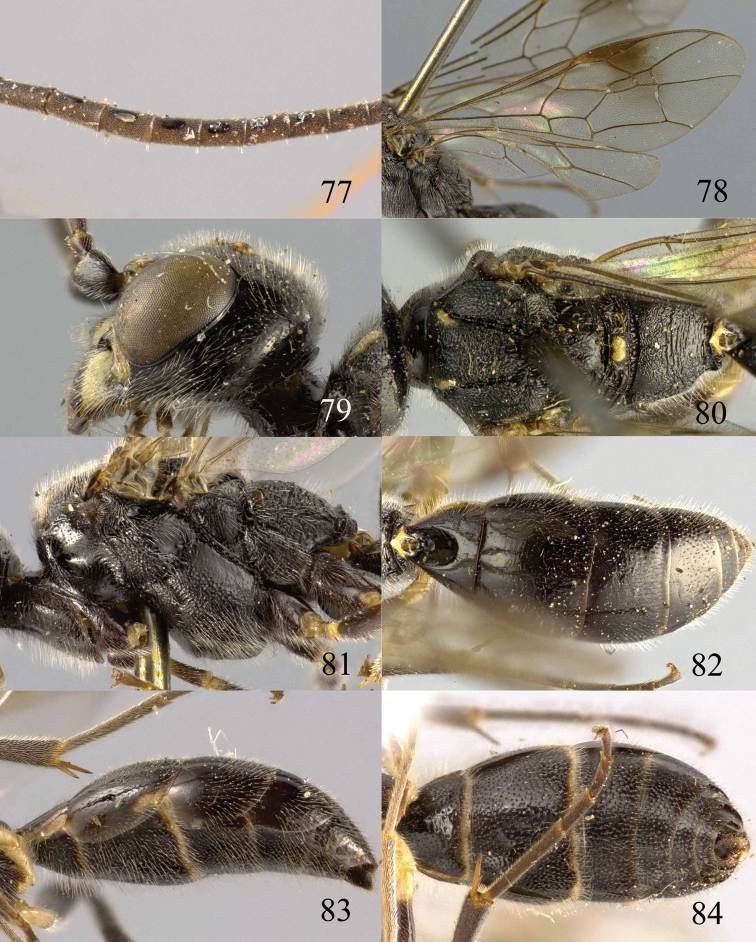
*Jezonogonalos luteata* sp. n., holotype, male. **77** Tyloids on 10^th^–16^th^ segments of antenna **78** fore and hind wings **79** head lateral **80** mesosoma dorsal **81** mesosoma lateral **82** metasoma dorsal **83** metasoma lateral **84** metasoma ventral.

#### Description.

Holotype, male, length of body 9.6 mm (of fore wing 7.7 mm).

*Head*. Antenna with 25 segments, tyloids circular, 0.5 times as long as segment on 11^th^–14^th^ segments and 0.2 times as long as segments on 10^th^ and 16^th^ segments (Fig. 77); frons densely punctate; vertex spaced and finely punctate (interspaces much wider than width of punctures) (Fig. 75), with short setae; temple largely smooth with sparse fine punctures (Fig. 79); head gradually narrowed behind eyes, eye in dorsal view 1.2 times as long as temple (Fig. 75); occipital carina moderately widened and lamelliform medio-dorsally, with many short carinae; supra-antennal elevations strongly enlarged (about 0.7 times as long as scapus), outer side subvertical and largely smooth except for sparse punctures; clypeus slightly concave and thick medio-ventrally.

*Mesosoma*. Length of mesosoma 1.8 times its height (Fig. 81); mesopleuron below transverse mesopleural groove reticulate-rugose anteriorly and largely smooth with sparse fine punctures posteriorly, above groove similar (Fig. 81); transverse mesopleural groove narrow, deep and moderately crenulate; notauli narrow anteriorly, widened posteriorly and coarsely crenulate; mesoscutum densely and coarsely rugose, rugae on lateral lobes of mesoscutum less coarse (Fig. 80); scutellar sulcus wide, both medially and laterally and coarsely crenulate; scutellum densely and coarsely rugose, convex medially and anteriorly near level of mesoscutum; metanotum medially protruding, obtuse and densely and finely punctate (Fig. 81); propodeum rugulose anteriorly with smooth patches antero-laterally, transversely striate medio-posteriorly (Fig. 80); posterior propodeal carina thick lamelliform, foramen medially 0.6 times higher than wide basally.

*Wings*. Fore wing: length of vein 1-M 2.2 times as long as vein 1-SR (Fig. 78).

*Metasoma*. First tergite 0.8 times as long as apically wide, smooth and with distinct elliptical depression medially (Fig. 82); second tergite largely smooth with sparse fine punctures posteriorly, other tergites superficial coriaceous anteriorly, densely finely punctate posteriorly (Fig. 82); sternites densely and rather coarsely punctate; second sternite weakly curved in lateral view; third sternite about 0.4 times as long as second sternite (Fig. 84); genitalia extruded (Fig. 83).

*Colour*. Black; mandible largely except brown teeth, inner orbita narrowly, apical half of supra-antennal elevations, pair of wide patches of clypeus, malar space pair of narrow patches of middle lobe of mesoscutum antero-laterally, small medial patch of metanotum, pair of small lateral patches on first and second tergites, apical margin of first and second sternites, base of fore trochanter and trochantellus, fore and hind trochanters and trochantelli ivory (Figs 74, 75, 80, 83, 84); metasoma ventrally black; palpi and antenna dark brown; ivory, fore tarsi and tibia yellowish brown, remainder of legs dark brown to black; pterostigma and anterior half of marginal cell dark brown, remainder of wing membrane subhyaline.

*Female*. Unknown.

#### Biology.

Unknown. Collected in August at 1800–2000 m.

#### Distribution.

China (Sichuan).

#### Etymology.

Named after the yellow supra-antennal protuberances: from “*luteus*” (Latin for “yellow”).

### Jezonogonalos nigrata sp. n.

http://zoobank.org/4E441D8D-46EE-4F36-8D64-B5CD8DFB9DB5

http://species-id.net/wiki/Jezonogonalos_nigrata

Figs 85 –95

#### Type material.

Holotype, ♀ (IZCAS) “[China:] Sichuan, Mt. Emei, Xixiangchi, 1800–2000 m, 17.VIII.1957, Ke-ren Huang, IOZ(E)1495442”. Paratypes: 2 ♀ (IZCAS) “[China:] Sichuan, Mt. Emei, Jiulaodong, 1800–1900 m, 28.VII.1957, Ke-ren Huang, IOZ(E)1495443; id., but 19.VIII.1957, You-cai Lu, IOZ(E)1495444”.

#### Diagnosis.

Occipital carina very wide and crenulate medio-dorsally (Fig. 87); outer side of supra-antennal elevations subvertical and elevations about 0.7 times as long as scapus (Figs 87, 90); apical half of supra-antennal elevations black (Fig. 87); head dorsally coarsely reticulate-punctate (Fig. 87); frons and vertex densely and coarsely punctate (Figs 86, 87); basal half of metasoma largely smooth, apical half mainly punctate (Fig. 93); metasoma largely black, second tergite with pair of small pale yellow spots postero-laterally (Fig. 94); first tergite about 0.7 times as long as its apical width (Fig. 95); second sternite simple (Fig. 95); third sternite about 0.2 times as long as second sternite (Fig. 95).

**Figures 85–87. F18:**
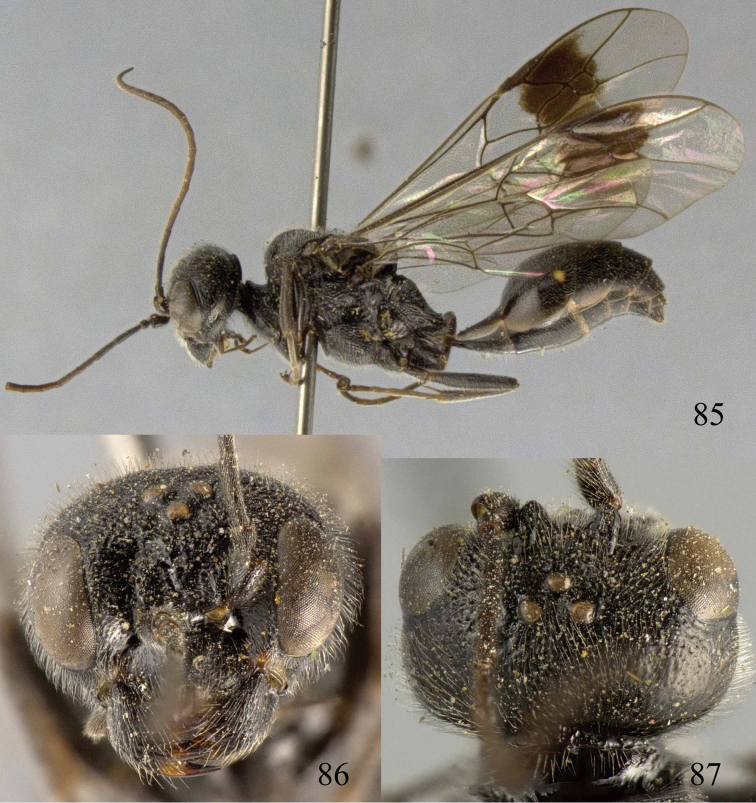
*Jezonogonalos nigrata* sp. n., holotype, female. **85** Habitus lateral **86** head anterior **87** head dorsal.

**Figures 88–95. F19:**
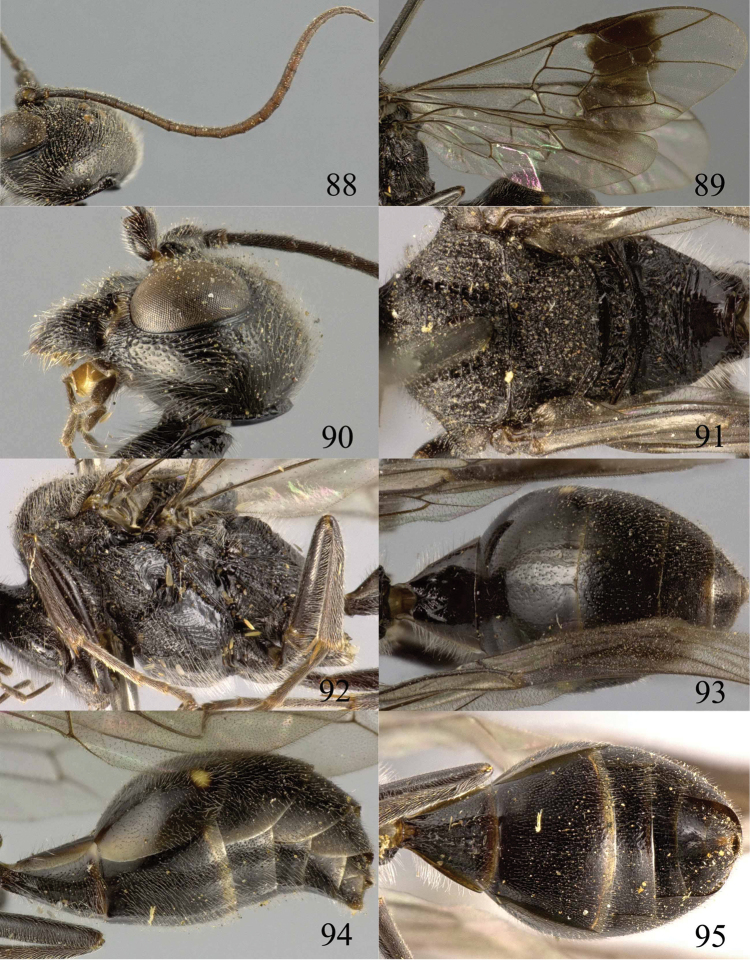
*Jezonogonalos nigrata* sp. n., holotype, female. **88** Antenna **89** fore and hind wings **90** head lateral **91** mesosoma dorsal **92** mesosoma lateral **93** metasoma dorsal **94** metasoma lateral **95** metasoma ventral.

#### Description.

Holotype, female, length of body 10.5 mm (of fore wing 8.6 mm).

*Head*. Antenna with 23 segments; frons densely and coarsely punctate; vertex densely punctate behind stemmaticum, becoming spaced punctate posteriorly (Fig. 87); temple largely smooth with sparse fine punctures (Fig. 90); head gradually narrowed behind eyes, eye in dorsal view 0.7 times as long as temple (Fig. 87); occipital carina strongly widened and lamelliform medio-dorsally, with many short carinae; supra-antennal elevations strongly enlarged (about 0.7 times as long as scapus), outer side subvertical and densely punctate; clypeus slightly concave and thick medio-ventrally.

*Mesosoma*. Length of mesosoma 1.6 times its height (Fig. 92); mesopleuron below transverse mesopleural groove obliquely rugulose anteriorly and largely smooth with sparse fine punctures posteriorly, above groove densely and coarsely rugose (Fig. 92); transverse mesopleural groove narrow, deep and coarsely crenulate; notauli wide and coarsely crenulate; mesoscutum densely and coarsely rugose (Fig. 93); scutellar sulcus wide, both medially and laterally and coarsely crenulate; scutellum coarsely reticulate-rugose, convex medially and anteriorly near level of mesoscutum; metanotum medially protruding, lamelliform and bluntly bifurcating apically and coarsely rugose (Fig. 93); propodeum obliquely rugulose anteriorly, transversely striate medially, smooth and shiny posteriorly (Fig. 93); posterior propodeal carina thick lamelliform, foramen medially 0.4 times higher than wide basally.

*Wings*. Fore wing: length of vein 1-M 1.1 times as long as vein 1-SR (Fig. 89).

*Metasoma*. First tergite 0.6 times as long as apically wide, smooth and with shallow elliptical depression medially (Fig. 93); second to sixth tergites largely smooth with sparse and fine punctures; sternites densely and finely punctate; second sternite slightly convex in lateral view; third sternite about 0.3 times as long as second sternite (Fig. 95); hypopygium triangular in ventral view (Fig. 95).

*Colour*. Black; malar space, lateral margin of all tergites and posterior margin of second sternite ivory; teeth of mandible, antenna and tegulae dark brown; second tergite with pair of small pale yellow spots postero-laterally; legs dark brown to black; pterostigma and posterior half of first submarginal cell to anterior half of marginal cell and area below that dark brown, remainder of wing membrane subhyaline.

*Variation*. Length of body 8.6–11.0 mm, of fore wing 6.8–8.7 mm; length of vein 1-M of fore wing 1.1–1.6 times as long as vein 1-SR; second to sixth tergites largely superficially coriaceous with sparse and fine punctures.

*Male*. Unknown.

#### Biology.

Unknown. Collected in July–August at 1800–2000 m.

#### Distribution.

China (Sichuan).

#### Etymology.

Named after the black supra-antennal protuberances: from “*niger*” (Latin for “black”).

### Jezonogonalos satoi

(Tsuneki, 1991) comb. n., re-instated

http://species-id.net/wiki/Jezonogonalos_satoi

Figs 96 –107

Taiwanogonalos satoi Tsuneki, 1991: 39. Synonymized by Carmean and Kimsey 1998 with *Taeniogonalos maga* Teranishi, 1929, and here re-instated as valid species.

#### Type material.

Holotype, ♂ (OMNH) “[China:] Taiwan, Habon, 1.V.1929, K. Sato”, “*Taiwanogonalos satoi* Tsuneki, ♂, holotype”.

#### Diagnosis.

Occipital carina wide lamelliform and with short carina medio-dorsally and some small carinae near it (Fig. 98); supra-antennal elevations entirely black; middle lobe of mesoscutum densely transversely striate (Fig. 103); propodeum with an irregular transverse ruga medially (Fig. 103); second submarginal cell of fore wing medium-sized, parallel-sided and laterally about as high as wide anteriorly (Fig. 101); fore wing with more or less conspicuous dark brown patch below pterostigma (Fig. 101); metasoma of ♂ black or dark brown dorsally or nearly so (Fig. 105); first tergite 0.8 times as long as its apical width (Fig. 105); third sternite about 0.4 times as long as second sternite (Fig. 107).

**Figures 96–99. F20:**
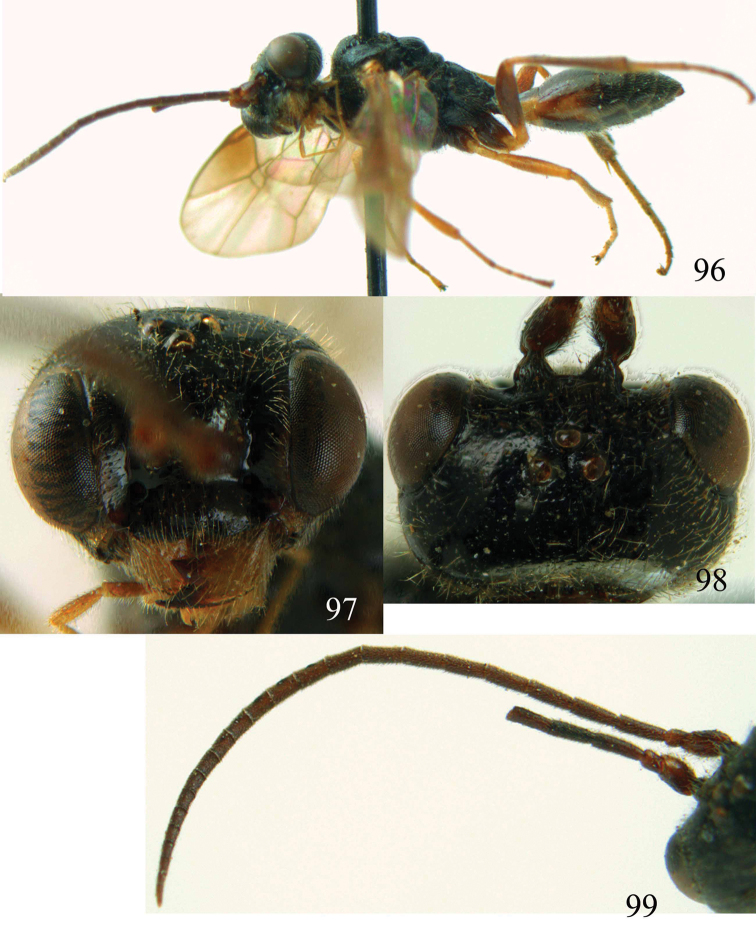
*Jezonogonalos satoi* (Tsuneki, 1991), holotype, male. **96** Habitus lateral **97** head anterior **98** head dorsal **99** antennae.

**Figures 100–107.**
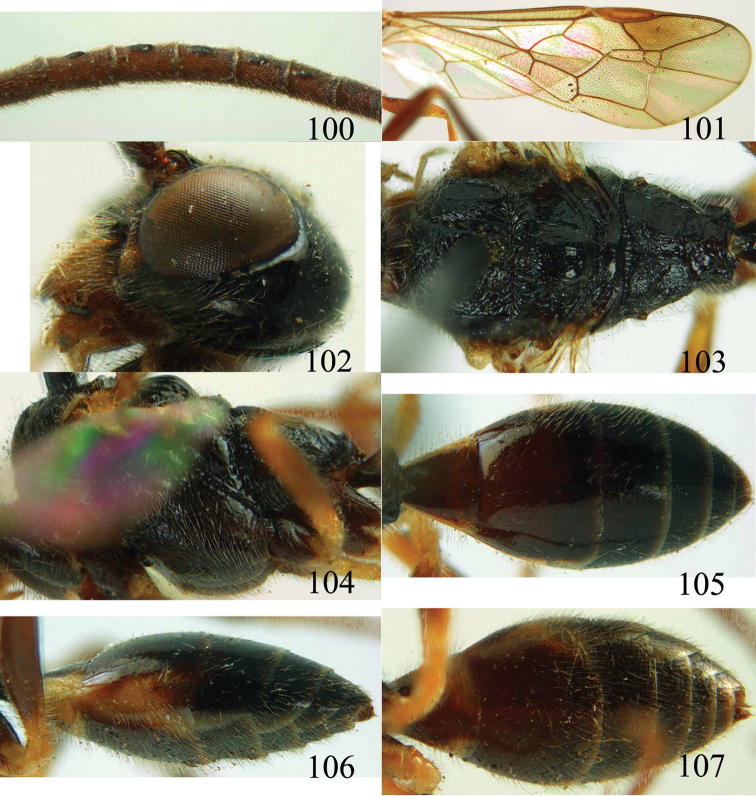
*Jezonogonalos satoi* (Tsuneki, 1991), holotype, male. **100** Tyloids on 10^th^–16^th^ segments of antenna **101** fore and hind wings **102** head lateral **103** mesosoma dorsal **104** mesosoma lateral **105** metasoma dorsal **106** metasoma lateral **107** metasoma ventral.

#### Description.

Holotype, male, length of body 6.9 mm (of fore wing 6.4 mm).

*Head*. Antenna with 24 segments, elliptical tyloids on 11^th^–15^th^ segments (Fig. 100); frons spaced punctulate; vertex largely smooth, sparsely punctulate and strongly shiny (Fig. 98), with medium-sized setae; temple largely smooth (Fig. 102); head gradually narrowed behind eyes, eye in dorsal view 1.7 times as long as temple (Fig. 98); occipital carina wide lamelliform medio-dorsally with one short carina and few short sublateral carinae; supra-antennal elevations strongly enlarged (about 0.6 times as long as scapus), outer side subvertical and largely smooth except for a longitudinal groove and sparse punctures, latero-basally with depression (Fig. 98); clypeus rather concave and thick medio-ventrally.

*Mesosoma*. Length of mesosoma 1.6 times its height (Fig. 104); mesopleuron below transverse mesopleural groove largely smooth but superficially rugulose anteriorly, above groove smooth but rugulose anteriorly and with distinct crenulate vertical groove (Fig. 104); transverse mesopleural groove wide, deep and widely crenulate; notauli moderately wide and coarsely crenulate; middle lobe of mesoscutum densely transversely striate (Fig. 103), mesoscutum partly smooth and shiny, spaced punctate, with some striae posteriorly (Fig. 103); scutellar sulcus wide, both medially and laterally and coarsely crenulate; scutellum largely smooth (except some punctulation and rugae and medio-posteriorly narrowly crenulate) and shiny, longitudinally slightly depressed medially and anteriorly above level of mesoscutum; metanotum medially hardly protruding, evenly convex and smooth (Fig. 103); propodeum rugulose anteriorly (and sulcus medially widely crenulate and depressed), with irregular transverse ruga medially (Fig. 103); posterior propodeal carina thick, wide lamelliform and curved, foramen medially 0.7 times higher than wide basally.

*Wings*. Fore wing: length of vein 1-M 2.2 times as long as vein 1-SR and distinctly curved; veins r and 2-SR long (Fig. 101); second submarginal cell of fore wing medium-sized, parallel-sided and laterally about as high as wide anteriorly.

*Metasoma*. First tergite 0.8 times as long as apically wide, smooth and with wide depression basally (Fig. 105); second and following tergites largely smooth and strongly shiny (Fig. 105); sternites smooth, except for superficial punctures; second sternite weakly curved in lateral view; third sternite 0.4 times as long as second sternite (Fig. 107).

*Colour*. Black or dark brown; mandible (except apically), palpi (except basally), malar space largely, inner orbita very narrowly, pronotum latero-dorsally, tegulae, first segment laterally, large patch of second segment laterally, trochanters, trochantelli and base of femora ivory (Fig. 96); remainder of legs more or less brown (but coxae dark brown); pterostigma (but medially pale brown), veins and marginal cell (except apically) brown; remainder of wing membrane subhyaline.

*Male*. Unknown.

#### Biology.

Unknown. Collected in May.

#### Distribution.

China (Taiwan).

### Lycogaster

Shuckard, 1841

http://species-id.net/wiki/Lycogaster

Figs 108 [Fig F23] [Fig F24] [Fig F25] [Fig F26] [Fig F27] [Fig F28] [Fig F29] –154

Lycogaster Shuckard, 1841: 121; Weinstein and Austin 1991: 414; Carmean and Kimsey 1998: 61. Type species (by original designation): *Lycogaster pullatus* Shuckard, 1841.

#### Diagnosis.

Body length 5.5–15.0 mm; antenna with 22–24 segments, of ♀ widened medially (Fig. 111, but hardly so in *Lycogaster angustula* sp. n.); antenna of ♂ without tyloids; supra-antennal elevations small, without depression dorsally (Figs 110, 121, 132, 146); vertex convex and shiny; mandibular condyli close to level of eyes (Figs 113, 122, 135, 149); apical segment of labial palp widened and obtuse, more or less triangular (Fig. 122); metanotum smooth, shiny and weakly convex (Figs 114, 123, 136, 150); triangular dorso-apical part of hind trochanter separated by an oblique groove; fore trochanter subparallel-sided and distinctly longer than hind trochanter; hind tarsus slightly or not modified; basal half of third metasomal sternite with a posteriorly steep, smooth and complete transverse ledge (Figs 118, 129, 140; may be partly hidden under second sternite and rather low in *Lycogaster violaceipennis*); second sternite with pair of small triangular teeth on apical protuberance (Fig. 118; but only with pair of lobe-shaped flaps in male of *Lycogaster violaceipennis*) and sometimes absent; epipleura of tergites laterally strongly pigmented; fifth sternite of ♀ distinctly emarginate medio-posteriorly (Fig. 118).

#### Biology.

In the New World reared as hyperparasitoid of Ichneumonidae in caterpillars of the families Saturniidae and Notodontidae (Carmean and Kimsey 1998). The record from Eumeninae nests probably concerns prey caterpillars that have been infested with both Ichneumonidae and Trigonalyidae.

#### Notes.

It is clear from the original description (Magretti 1897) and from the redescription of Schulz (1908) that *Lycogaster rufiventris* (Magretti, 1897) from Myanmar was correctly assigned to the genus *Lycogaster* by Schulz (1908), Bischoff (1938) and Weinstein and Austin (1991). It has the medial third of the fore wing dark brown as *Lycogaster flavonigrata* and *Lycogaster nigralva*, but differs by the entire reddish metasoma, the absence of distinct separate teeth besides finely dentate lamelliform protuberance of second sternite and the distinctly convex vertex. The interpretation by Carmean and Kimsey (1998; based on specimens from central and southern India and retaining this species in the genus *Trigonalys*) seems to be incorrect considering the redescription of the holotype female by Schulz (1908) and the distribution of the genus *Trigonalys*.

#### Key to Chinese species of *Lycogaster* Shuckard, 1841

**Table:** 

1	Mesosoma largely reddish brown dorsally; second metasomal sternite with pair of small sublateral flaps, flat medio-posteriorly and sternite weakly convex medially in lateral view; [fore wing largely dark brown; mandible largely ivory (except dark brown teeth)]	*Lycogaster violaceipennis* Chen, 1949
–	Mesosoma black dorsally, at most with vertical row of yellow patches; second sternite with pair of small submedial acute teeth on elevation (Fig. 118) or without sublateral teeth and sternite strongly convex medio-posteriorly (Figs 129, 140)	2
2	Submedial acute teeth of second sternite close to each other (Fig. 118); antenna of ♀ elongate and hardly widened medially (Fig. 111); second tergite smooth and strongly shiny (Fig. 116); medial third of fore wing slightly darkened (Fig. 112)	*Lycogaster angustula* sp. n.
–	Submedial acute teeth of second sternite comparatively far removed from each other (Figs 129, 140); antenna of ♀ less elongate and distinctly widened medially (Figs 124, 133); second tergite punctate and moderately shiny (Figs 127, 138); medial third of fore wing largely dark brown (Figs 125, 134)	3
3	Second metasomal sternite largely yellow posteriorly (Fig. 118); vertex comparatively flat in lateral view (Fig. 113); medio-posterior lamella of second sternite subtruncate to weakly convex (Fig. 129)	*Lycogaster flavonigrata* sp. n.
–	Second sternite black posteriorly (Fig. 140), at most somewhat brownish; vertex less flattened in lateral view (Fig. 135); medio-posterior lamella of second sternite distinctly convex (Fig. 140)	*Lycogaster nigralva* sp. n.

### Lycogaster angustula sp. n.

http://zoobank.org/327268E0-5B21-41D9-B2ED-DD7B7F358490

http://species-id.net/wiki/Lycogaster_angustula

Figs 108 –118

#### Type material.

Holotype, ♀ (ZJUH) “[China:] Zhejiang, Mt. Tianmu, 29.VII.2011, Sheng-nan Son, 201101299”. Paratypes: 1 ♀ (ZJUH) “[China:] Zhejiang, Mt. Tianmu, Xianrending, 1520 m, 1.VII.2001, Mei-hua Piao, 200106404”; 1 ♂ (ZJUH), “[China:] Zhejiang, Qingyuan, Mt. Baishanzu, 1856 m, 13.VIII.2003, Xiao-xia Yu, 20058591”.

#### Diagnosis.

Antenna of female elongate and hardly widened medially (Fig. 111); pair of small submedial acute teeth on elevation of second sternite close to each other (Fig. 118); second tergite smooth and strongly shiny (Fig. 116); mesosoma black dorsally.

**Figures 108–110.**
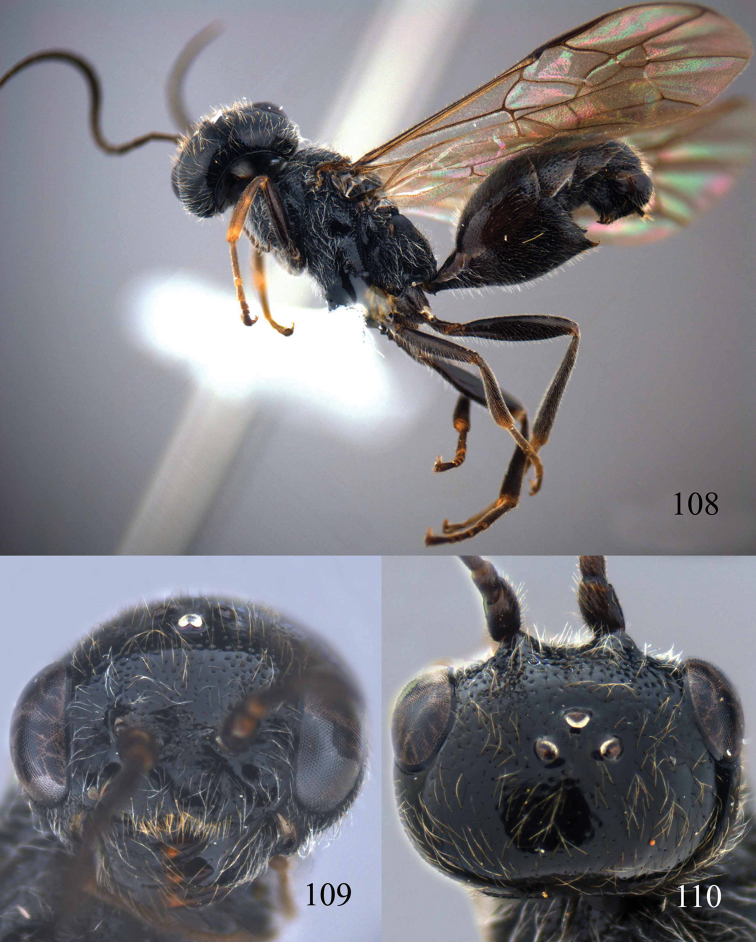
*Lycogaster angustula* sp. n., holotype, female. **108** Habitus lateral **109** head anterior **110** head dorsal.

**Figures 111–118. F23:**
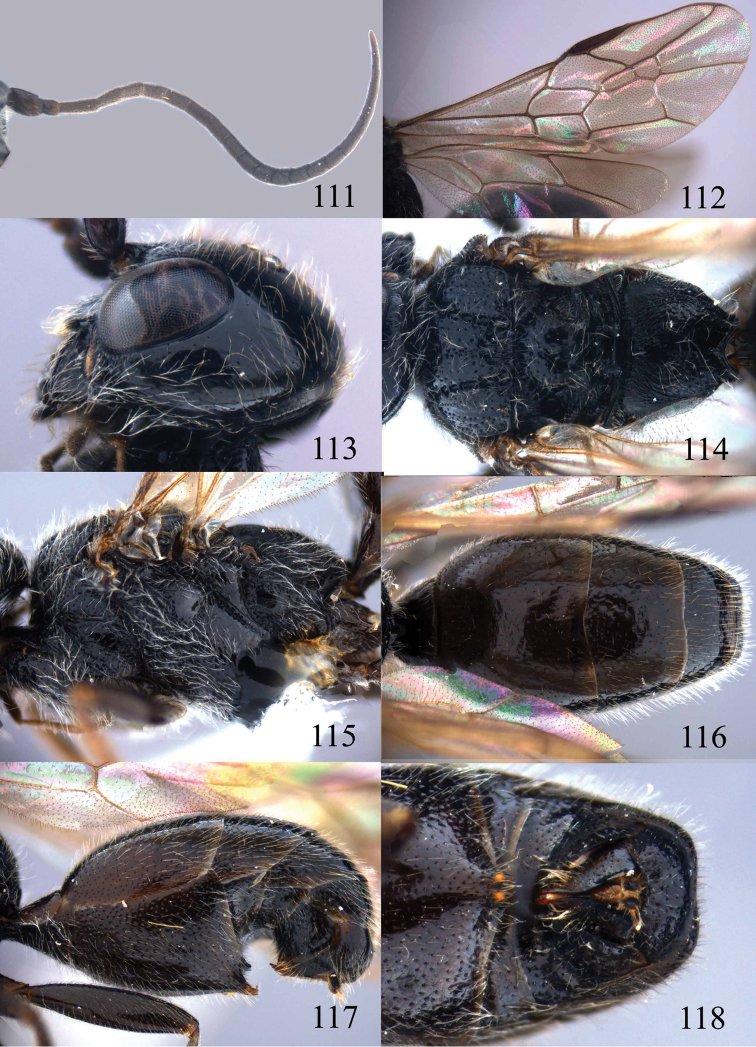
*Lycogaster angustula* sp. n., holotype, female. **111** Antenna **112** fore and hind wings **113** head lateral **114** mesosoma dorsal **115** mesosoma lateral **116** metasoma dorsal **117** metasoma lateral **118** metasoma ventral.

#### Description.

Holotype, female, length of body 6.7 mm (of fore wing 5.4 mm).

*Head*. Antenna with 23 segments, elongate and hardly widened medially (Fig. 111); frons spaced punctate (interspaces much wider than width of punctures) and shiny (Fig. 109); vertex and temple largely smooth with sparse and fine punctures (Figs 110, 113); head subparallel-sided behind eyes, distinctly narrowed posteriorly and 1.1 times wider than mesoscutum (Fig. 110); dorsal length of eye 0.9 times length of temple (Fig. 110); occipital carina narrow lamelliform medio-dorsally and without crenulae; supra-antennal elevations hardly developed as a thin rim and smooth; clypeus weakly emarginate and comparatively thin medio-ventrally.

*Mesosoma*. Length of mesosoma 1.4 times its height (Fig. 115); mesopleuron below transverse mesopleural groove rugose to punctate anteriorly, largely smooth and shiny posteriorly, above groove similar but rugose anteriorly; transverse mesopleural groove wide, deep and coarsely crenulate; notauli narrow anteriorly, widened posteriorly and coarsely crenulate; middle lobe of mesoscutum sparsely and coarsely punctate anteriorly, rugose at posterior end, lateral lobes sparsely and rather finely punctate (Fig. 114); scutellar sulcus wide, both medially and laterally and coarsely crenulate (Fig. 114); scutellum largely smooth (except some punctures), distinctly shiny, rather flat and anteriorly near level of mesoscutum; metanotum flat, shiny and smooth medially (Fig. 114); propodeum largely obliquely rugulose to rugose with smooth interspaces medially and posteriorly (Fig. 114); posterior propodeal carina thick lamelliform and slightly arched, foramen medially 0.5 times higher than wide basally.

*Wings*. Fore wing: length of vein 1-M 1.7 times as long as vein 1-SR (Fig. 112).

*Metasoma*. First tergite 0.4 times as long as apically wide, smooth and with rather distinct elliptical depression medially (Fig. 116); second-fifth tergites largely smooth with sparse fine punctures; sternites sparsely and finely punctate; second sternite strongly convex in lateral view, its medio-apical protuberance with pair of small submedial acute teeth close to each other (Fig. 118); third sternite about 0.1 times as long as second sternite, with distinct ledge (Fig. 117); hypopygium truncate apically (Fig. 118).

*Colour*. Black; metasoma somewhat brownish; mandibular teeth, palpi and tegulae dark brown; legs dark brown to black with tibiae and tarsi paler; pterostigma dark brown; wing membrane subhyaline.

*Variation*. Length of body 7.9 mm, of fore wing 6.1 mm; second tergite with pair of small ivory spots medio-laterally; second–fifth tergites rather densely and coarsely punctate.

*Male*. Length of body 8.7 mm, of fore wing 6.7 mm; antenna with 23 segments and without tyloids; vertex sparsely and rather coarsely punctate; sculpture of mesosoma coarser than that in female; second–fifth tergites and sternites densely and coarsely punctate; genitalia extruded.

#### Biology.

Unknown. Collected in July–August at 1520–1856 m.

#### Distribution.

China (Zhejiang).

#### Etymology.

Named after the elongate and hardly widened antenna of the female: from “*elongatus*” (Latin for “prolonged”).

### Lycogaster flavonigrata sp. n.

http://zoobank.org/DE38F6CD-DC14-4F1A-AE5C-DF1ED01B67AC

http://species-id.net/wiki/Lycogaster_flavonigrata

Figs 119 –129

Lycogaster celebesiensis ; Carmean and Kimsey 1998: 62 (misidentification of *Lycogaster celebesiensis* (Szépligeti, 1902)).

#### Type material.

Holotype, ♀ (ZJUH) “[China:] Fujian, Shaxian County, 14.X.1980”. Paratypes: 1 ♀ (IZCAS) “[China:] Yunnan, Xishuangbanna, Damenglong, 650 m, 11.IV.1958, Shu-yong Wang, IOZ(E)1495238”; 1 ♀ (IZCAS) “[China:] Yunnan, Xishuangbanna, 850 m, 22.VIII.1958, Le-yi Zheng, IOZ(E)1495239”; 1 ♀ (CASSF) “China, S. Kiangsi, Sunwu, 12.vii.1936”, “L. Gressitt collector”, “*Lycogaster celebesiensis* (Szépligeti, 1902). Det. Carmean, 1993”.

#### Diagnosis.

Antenna of female distinctly widened medially (Fig. 124); outer side of supra-antennal elevations strongly oblique and elevations 0.2 times as long as scapus (Fig. 121); vertex comparatively flat in lateral view (Fig. 122); area between ocelli and eye distinctly punctate (Fig. 121); mandible and mesosoma entirely black (Figs 120, 123); lateral lobes of mesoscutum sparsely punctate laterally (Fig. 123); medial third of fore wing largely dark brown (Fig. 125); vein m-cu of fore wing subinterstitial (Fig. 125); second metasomal sternite largely yellow posteriorly (Fig. 129); second sternite with pair of comparatively far separated small triangular teeth sublaterally and in between sternite convexly protruding medio-posteriorly (Fig. 129); second tergite punctate and moderately shiny (Fig. 127).

**Figures 119–123. F24:**
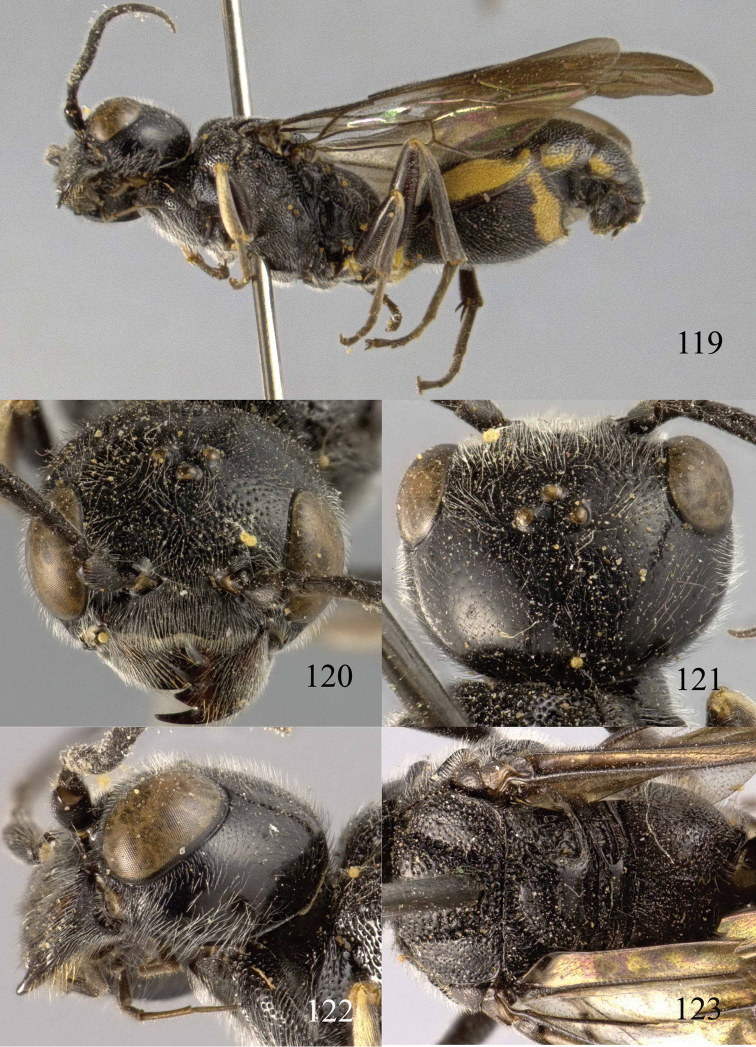
*Lycogaster flavonigrata* sp. n., holotype, female. **119** Habitus lateral **120** head anterior **121** head dorsal **122** head lateral **123** mesosoma dorsal.

**Figures 124–129. F25:**
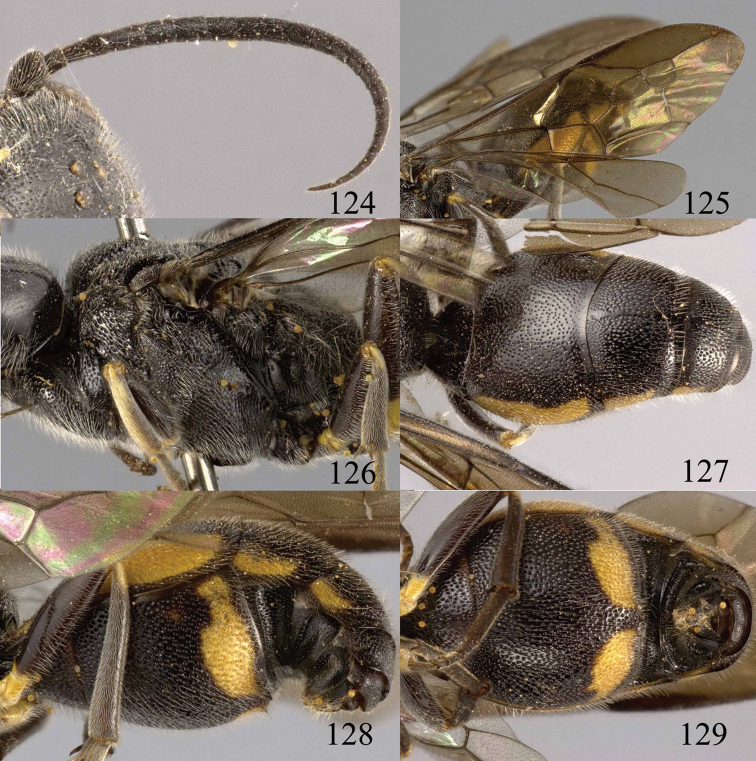
*Lycogaster flavonigrata* sp. n., holotype, female. **124** Antenna **125** fore and hind wings **126** mesosoma lateral **127** metasoma dorsal **128** metasoma lateral **129** metasoma ventral.

#### Description.

Holotype, female, length of body 13.4 mm (of fore wing 10.1 mm).

*Head*. Antenna with 23 segments, segments after third segment distinctly widened and after 14^th^ segments becoming gradually slenderer (Fig. 124); frons coarsely and rather densely punctate and vertex spaced punctate (interspaces often much wider than width of punctures) and shiny (Figs 120, 121), with short greyish setae; head subparallel-sided behind eyes, distinctly narrowed posteriorly and 1.3 times wider than mesoscutum (Fig. 121); dorsal length of eye 0.9 times length of temple (Fig. 121); temple smooth, shiny and with medium-sized whitish setae; occipital carina narrow lamelliform medio-dorsally and without crenula; supra-antennal elevations hardly developed as a thin rim and smooth; clypeus weakly emarginate and comparatively thin medio-ventrally.

*Mesosoma*. Length of mesosoma 1.3 times its height (Fig. 126); mesopleuron below transverse mesopleural groove densely rugose anteriorly and spaced punctate and weakly shiny posteriorly, above groove similar but rugae finer posteriorly denser punctate; transverse mesopleural groove narrow, deep and moderately crenulate; notauli narrow anteriorly, widened posteriorly and coarsely crenulate; middle lobe of mesoscutum coarsely vermiculate rugose and weakly shiny (Fig. 126), its lateral lobes mainly coarsely punctate and medially with smooth interspaces; scutellar sulcus wide, both medially and laterally and coarsely crenulate; scutellum largely smooth (except some punctures), distinctly shiny, rather flat and anteriorly near level of mesoscutum; metanotum flat, shiny and smooth medially (Fig. 126); propodeum largely densely finely rugose and carina of foramen thick and arched (Fig. 126); foramen medially 0.6 times higher than wide basally.

*Wings*. Fore wing: length of vein 1-M 2.3 times as long as vein 1-SR (Fig. 125).

*Metasoma*. First tergite 0.4 times as long as apically wide, gradually narrowed basally, flattened medially and weakly convex apically; second–fifth tergites largely densely coarsely punctate and rather moderately shiny; second sternite similarly sculptured as tergite, its medio-apical protuberance lamelliform and convex medio-apically (Fig. 129) and with pair of triangular submedial teeth, third sternite only with distinct ledge (Fig. 129); hypopygium truncate apically.

*Colour*. Black (including mandible); palpi, fore tibia and tarsus, tegulae, pterostigma and veins dark brown; apex of middle trochantellus, hind trochanter and trochantellus, and narrowly base of hind femur ivory; pair of large lateral patches on second and third tergites and apically on second sternite yellow; fore wing largely dark brown but paler subbasally (Fig. 125).

*Variation*. Length of body 11.5–13.1 mm, of fore wing 8.9–10.0 mm; length of vein 1-M 1.9–2.1 times as long as vein 1-SR; outer side of fore tibia ivory; vein m-cu of fore wing subinterstitial or postfurcal by 0.2 times length of vein m-cu; third tergite with a pair of small to medium-sized yellow or ivory patches laterally.

*Male*. Unknown.

#### Biology.

Unknown. Collected in April, July, August and October at 650–850 m.

#### Distribution.

China (Fujian, Jiangxi, Yunnan).

#### Etymology.

Name derived from “*flavus*” (Latin for “yellow) and “*niger*” (Latin for “black”) because of the yellow-black pattern of the second sternite.

#### Notes.

This species was confused with *Lycogaster celebesiensis* (Szépligeti) by Carmean and Kimsey (1998). *Lycogaster flavonigrata* differs as follows: medial third of fore wing dark brown (largely subhyaline of *Lycogaster celebesiensis*); area between ocelli and eye distinctly punctate (mainly smooth); mandible and mesosoma entirely black (with yellow patches); protuberance of second metasomal sternite convexly protruding medially or truncate (straight or weakly concave medio-apically).

### Lycogaster nigralva sp. n.

http://zoobank.org/B9B283BF-EDB3-466D-A7E7-7DF2EFD628D2

http://species-id.net/wiki/Lycogaster_nigralva

Figs 130 –140

#### Type material.

Holotype, ♀ (IZCAS) “[China], Sichuan, Mt. Emei, Baoguosi, 550–750 m, 23.V.1957, Fu-xing Zhu”.

#### Diagnosis.

Antenna of female distinctly widened medially (Fig. 133); vertex less flattened in lateral view than that of *Lycogaster flavonigrata* (Fig. 135); area between ocelli and eye distinctly punctate (Fig. 132); mandible and mesosoma entirely black (Figs 131, 136); lateral lobes of mesoscutum densely punctate laterally (Fig. 136); vein m-cu of fore wing comparatively far postfurcal (Fig. 134); medial third of fore wing largely dark brown (Fig. 134); second sternite black posteriorly (Fig. 140); medio-posterior lamella of second sternite convex (Fig. 140); protuberance of second metasomal sternite convexly protruding medially or truncate (Fig. 140), submedial acute teeth of second sternite comparatively far removed from each other (Fig. 140); second tergite punctate and moderately shiny (Fig. 138).

**Figures 130–132. F26:**
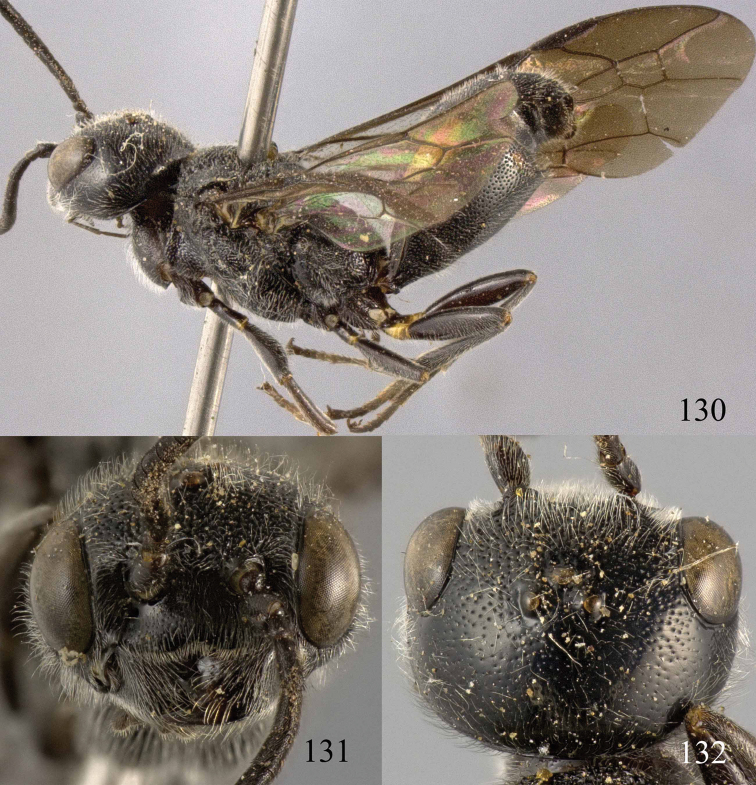
*Lycogaster nigralva* sp. n., holotype, female. **130** Habitus lateral **131** head anterior **132** head dorsal.

**Figures 133–140. F27:**
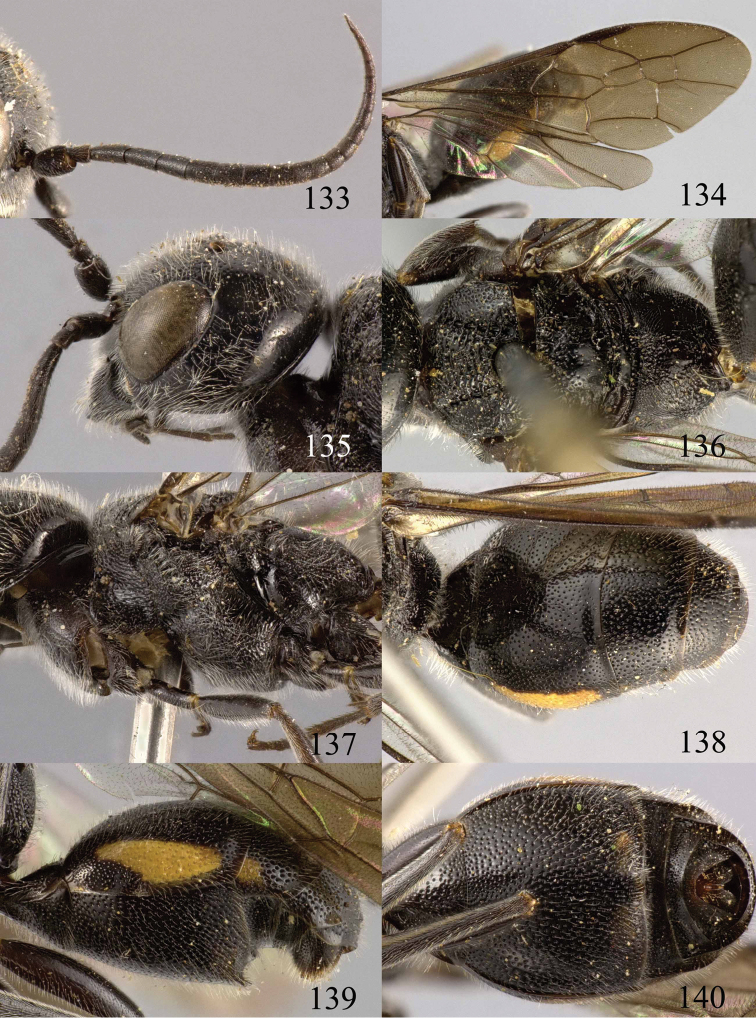
*Lycogaster nigralva* sp. n., holotype, female. **133** Antenna **134** fore and hind wings **135** head lateral **136** mesosoma dorsal **137** mesosoma lateral **138** metasoma dorsal **139** metasoma lateral **140** metasoma ventral.

#### Description.

Holotype, female, length of body 8.9 mm (of fore wing 6.8 mm).

*Head*. Antenna with 23 segments, segments after third segment distinctly widened and after 14^th^ segments becoming gradually slenderer (Fig. 133); frons coarsely and rather densely punctate and vertex spaced punctate (interspaces often much wider than width of punctures) and shiny (Figs 131, 132), with short greyish setae; head subparallel-sided behind eyes, distinctly narrowed posteriorly and 1.1 times wider than mesoscutum (Fig. 132); dorsal length of eye 0.7 times length of temple (Fig. 132); temple smooth, shiny and with medium-sized whitish setae; occipital carina narrow lamelliform medio-dorsally and without crenula; supra-antennal elevations hardly developed as a thin rim and smooth; clypeus weakly emarginate and comparatively thin medio-ventrally.

*Mesosoma*. Length of mesosoma 1.6 times its height (Fig. 137); mesopleuron below transverse mesopleural groove densely rugose anteriorly and spaced punctate and weakly shiny posteriorly, above groove densely rugose; transverse mesopleural groove narrow, deep and moderately crenulate; notauli widened, deep and coarsely crenulate; middle lobe of mesoscutum transversely rugose and weakly shiny (Fig. 138), lateral lobes densely and coarsely punctate; scutellar sulcus wide, both medially and laterally and coarsely crenulate; scutellum largely smooth (except some punctures), distinctly shiny, rather flat and anteriorly near level of mesoscutum; metanotum flat, shiny and smooth medially (Fig. 138); propodeum oblique rugulose anterio-laterally, remainder of propodeum coarsely rugose with small smooth interspaces medially and carina of foramen thick and arched (Fig. 138); foramen medially 0.8 times higher than wide basally.

*Wings*. Fore wing: length of vein 1-M 2.1 times as long as vein 1-SR (Fig. 134).

*Metasoma*. First tergite 0.4 times as long as apically wide, gradually narrowed basally, with shallow elliptical depression medially (Fig. 138) and flattened apically; second–fifth tergites spaced punctate (interspaces much wider than width of punctures) and rather shiny; second sternite coarsely and densely punctate, its medio-apical protuberance lamelliform and convex medio-apically (Fig. 140) and with pair of triangular submedial teeth, third sternite only with distinct ledge (Fig. 140); hypopygium truncate apically.

*Colour*. Black (including mandible); apex of all trochanters ivory, all tarsus, tegulae, pterostigma and veins dark; pair of large lateral patches on second tergite and small patches on basal third tergite and apically on second sternite yellow; fore wing largely brown but paler subbasally (Fig. 134).

*Male*. Unknown.

#### Biology.

Unknown. Collected in May at 550–750 m.

#### Distribution.

China (Sichuan).

#### Etymology.

Name derived from “*niger*” (Latin for “black”) and “*alvus*” (Latin for “belly”) because of the entirely black second sternite.

### Lycogaster violaceipennis

Chen, 1949

http://species-id.net/wiki/Lycogaster_violaceipennis

Figs 141 – 154

Lycogaster violaceipennis Chen, 1949: 10; Weinstein and Austin 1991: 415; Carmean and Kimsey 1998: 62; He 2004: 73.

#### Type material.

Lectotype here designated, ♂ (IZCAS) “[China: Zhejiang], Mokan Shan (= Mt. Mogan), Musée Heude”, “10.VII.[19]37, O. Piel”, “♂ *Lycogaster violaceipennis* Chen”. Paralectotype: 1 ♂ (IZCAS); damaged, mainly head and mesosoma remaining), with same label data.

#### Additional material.

1 ♀ + 1 ♂ (IZCAS) “[China:] Sichuan, Mt. Emei, Baoguosi, 550–750 m, 7.VI.1957, You-cai Lu, IOZ(E)1495236; id., but 30.V.1957, Fu-xing Zhu, IOZ(E)1495237”; 1 ♀ (IZCAS) “[China:] Sichuan, Mt. Emei, 4.VI.1957, Ke-ren Huang, IOZ(E)1495438”.

#### Diagnosis.

Outer side of supra-antennal elevations strongly oblique and elevations 0.2 times as long as scapus (Fig. 146); antenna of ♀ comparatively less elongate and distinctly widened medially; area between ocelli and eye largely coarsely and densely punctate (Fig. 146); mandible black (♀) or largely ivory (except dark brown teeth; ♂) (Fig. 145); mesosoma largely reddish brown dorsally; fore wing largely dark brown (Fig. 148); second tergite punctate and moderately shiny; second sternite of ♀ with pair of widely separated triangular teeth sublateral teeth and in between sternite strongly convexly protruding medio-posteriorly (Fig. 143); second metasomal sternite of ♂ with pair of small sublateral flaps, flat medio-posteriorly and sternite weakly convex medially in lateral view (Fig. 154).

**Figures 141–143. F28:**
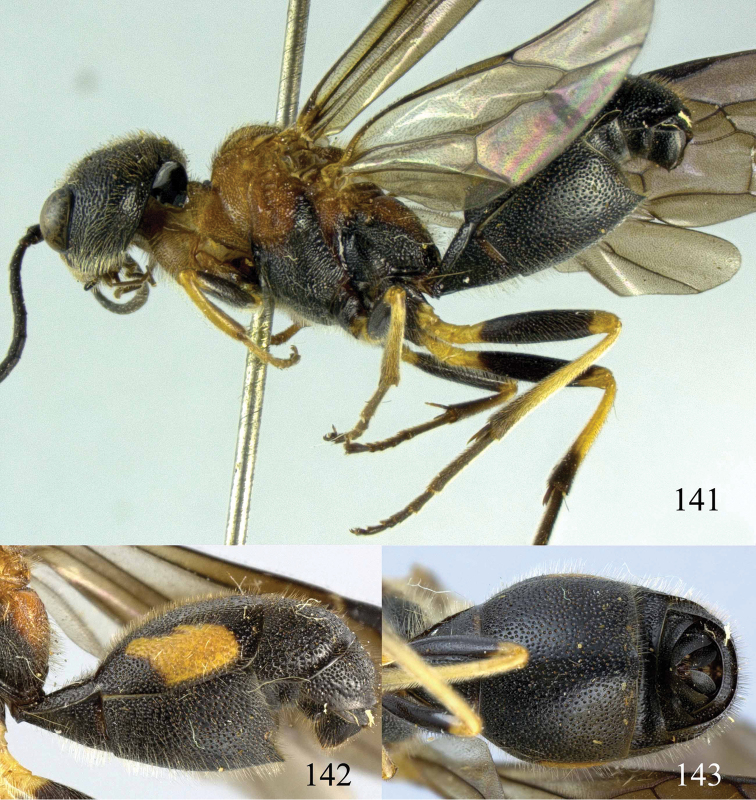
*Lycogaster violaceipennis* Chen, 1949, female from Sichuan. **141** Habitus lateral **142** metasoma lateral **143** metasoma ventral.

**Figures 144–146. F29:**
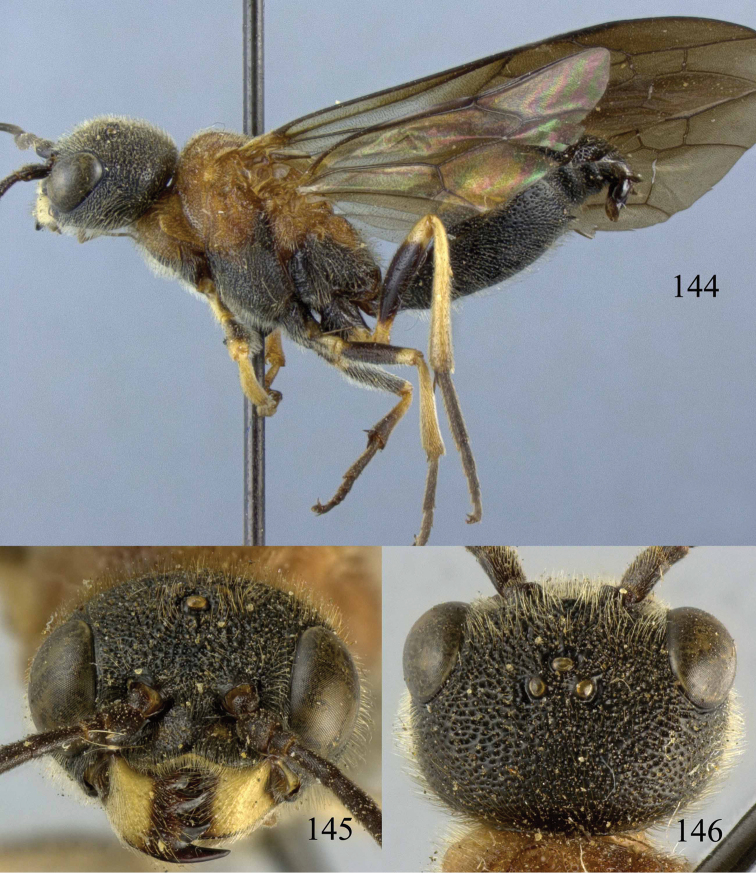
*Lycogaster violaceipennis* Chen, 1949, lectotype, male. **144** Habitus lateral **145** head anterior **146** head dorsal.

**Figures 147–154.**
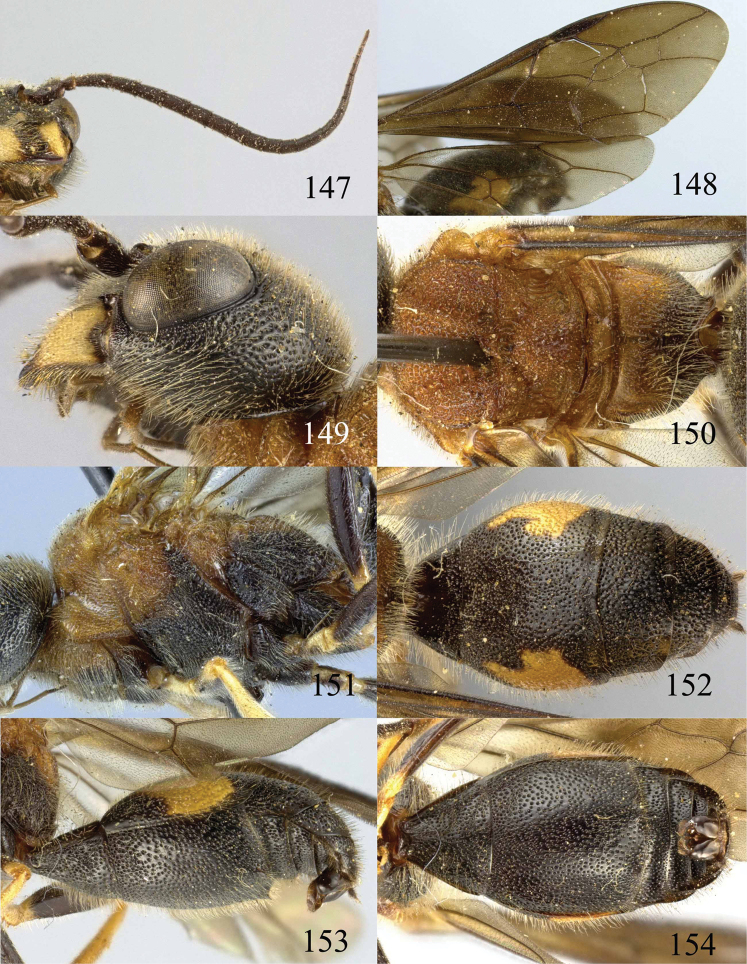
*Lycogaster violaceipennis* Chen, 1949, lectotype, male. **147** Antenna **148** fore and hind wings **149** head lateral **150** mesosoma dorsal **151** mesosoma lateral **152** metasoma dorsal **153** metasoma lateral **154** metasoma ventral.

#### Description.

Lectotype, male, length of body 14.4 mm (of fore wing 11.5 mm).

*Head*. Antenna with 24 segments, without tyloids, segments after third segment distinctly widened and after 14^th^ segments becoming gradually slenderer (Fig. 147); frons and vertex coarsely and densely reticulate-punctate (Figs 145, 146), with medium-sized yellowish setae; head subparallel-sided behind eyes, gradually narrowed posteriorly and about 1.3 times wider than mesoscutum (Fig. 146); dorsal length of eye 0.8 times length of temple (Fig. 146); temple coarsely and densely reticulate-punctate, with rather long yellowish setae; occipital carina narrow lamelliform medio-dorsally, with a few weak carinae; supra-antennal elevations hardly developed as a thin rim and smooth; clypeus weakly emarginate and comparatively thin medio-ventrally.

*Mesosoma*. Length of mesosoma 1.6 times its height (Fig. 151); mesopleuron below transverse mesopleural groove coarsely and densely reticulate-punctate anteriorly and oblique rugose posteriorly, above groove coarsely rugose with several large crenulae medially; transverse mesopleural groove narrow, deep and moderately crenulate; notauli widened, coarsely crenulate; mesoscutum densely reticulate-rugose (Fig. 150); scutellar sulcus wide, both medially and laterally and coarsely crenulate; scutellum densely reticulate-rugose, rather flat and anteriorly near level of mesoscutum; metanotum flat, shiny and smooth medially (Fig. 150); propodeum irregularly rugose and carina of foramen thick and arched (Fig. 150); foramen medially 0.5 times higher than wide basally.

*Wings*. Fore wing: length of vein 1-M 1.4 times as long as vein 1-SR (Fig. 148).

*Metasoma*. First tergite 0.4 times as long as apically wide, gradually narrowed basally, with shallow elliptical depression medially (Fig. 152) and flattened apically; second–fifth tergites largely densely and coarsely punctate and rather moderately shiny; second sternite similarly sculptured as tergite, with pair of small sublateral flaps (Fig. 154), flat medio-posteriorly and sternite weakly convex medially in lateral view; third sternite about 0.2 times as long as second sternite (Fig. 154); genitalia extruded (Fig. 153).

*Colour*. Black; mandible largely ivory except dark brown teeth; palpi, pterostigma and veins dark brown; mesosoma largely reddish brown except for ventral half of mesopleuron and metapleuron and posterior half of propodeum dark brown to black; outer side of fore femur, large part of middle and hind femur, all coxae and tarsus dark brown, remainder of legs ivory; pair of large lateral patches on second tergite yellow; fore wing largely dark brown, but paler subbasally (Fig. 148).

*Variation*. Length of body 12.3 mm, of fore wing 9.7 mm; legs darker; length of vein 1-M of fore wing 2.0 times as long as vein 1-SR.

*Female*. Length of body 14.4–15.0 mm, of fore wing 11.5–12.0 mm; medio-apical protuberance of second sternite lamelliform and convex medio-apically (Fig. 143) and with pair of triangular submedial teeth, third sternite with distinct ledge (Fig. 143); hypopygium incised apically.

#### Biology.

Unknown. Collected in May–July at 550–750 m.

#### Distribution.

China (Zhejiang, Sichuan).

### Orthogonalys

Schulz, 1905

http://species-id.net/wiki/Orthogonalys

Figs 155 [Fig F32] [Fig F33] [Fig F34] [Fig F35] [Fig F36] [Fig F37] [Fig F38] [Fig F39] [Fig F40] [Fig F41] [Fig F42] [Fig F43] [Fig F44] [Fig F45] [Fig F46] [Fig F47] –241

Orthogonalys Schulz, 1905: 76; Weinstein and Austin 1991: 421; Carmean and Kimsey 1998: 52; Smith and Tripotin 2012: 3. Type species (by original designation): *Orthogonalys boliviana* Schulz, 1905.
Orthogonalos Schulz, 1907: 8; Marshakov 1981: 104; Tsuneki 1991: 19; Weinstein and Austin 1991: 421. Unjustified emendation.
Satogonalos Teranishi, 1931: 10; Weinstein and Austin 1991: 424; Lelej 1995: 12. Type species (by original designation): *Satogonalos debilis* (Teranishi, 1929). Synonymized by Tsuneki 1991.

#### Diagnosis.

Body length 3.5–14.1 mm; antenna with 21–32 segments, often with a pale band in apical third of antenna and slender medially (Fig. 158), of male without tyloids (Fig. 199); supra-antennal elevations smooth and shiny, usually comparatively large (Figs 157, 170, 188, 222, 233), without depression dorsally and moderately to widely separated; vertex normal, at most with slight median depression dorsally (Figs 157, 170, 188, 222, 233); apical segment of labial palp widened and obtuse, more or less triangular (Fig. 2); mandibles wide in anterior view and sublaterally attached to head (Fig. 156); occipital carina narrow and smooth but widened and finely crenulate in *Orthogonalys hagoromonis*; mesoscutum and scutellum often smooth or sparsely punctulate (Fig. 174), at most moderately punctate with wide smooth interspaces, but mesoscutum densely sculptured anteriorly in *Orthogonalys hagoromonis*; metanotum concave latero-dorsally and often sculptured, matt and distinctly convex medially (Fig. 161); anterior propodeal sulcus distinctly crenulate (Fig. 174), rarely partly reduced; posterior propodeal carina curved and lamelliform; but reduced in *Orthogonalys formosana* and *Orthogonalys hagoromonis*; vein 1-SR of fore wing medium-sized to long (Figs 159, 172, 211, 224, 235); fore wing subhyaline (Fig. 224), at most slightly infuscate below pterostigma in female; triangular dorso-apical part of hind trochanter separated by an oblique groove (Fig. 230); fore trochanter subparallel-sided and distinctly longer than hind trochanter; hind tarsus slightly or not modified; second metasomal sternite and tergite flat in lateral view, weakly sclerotized and smooth (Fig. 164); second sternite in ventral view flat medially or weakly convex and no medial elevation or teeth posteriorly; basal half of third sternite flat, without a distinct ledge anteriorly (Fig. 241); fifth sternite of female straight or slightly *emarginate* medio-posteriorly (Fig. 241); body often slender (including metasoma) and sometimes ichneumonid-like (Fig. 231).

#### Biology.

In the New World reared as hyperparasitoid of Tachinidae in caterpillars (Carmean and Kimsey 1998; Murphy et al. 2009).

#### Key to Chinese species of *Orthogonalys* Schulz, 1905

**Table:** 

1	Mesosoma with linear ivory pattern dorsally (at least with row of large ivory patch on scutellum and patch on metanotum; Figs 174, 182, 226, 237); first metasomal tergite of ♀ with brownish or ivory apical transverse band (Figs 163, 184, 228, 239), at most with narrow brown median line; vein 1m-cu of fore wing connected to first submarginal cell (Figs 159, 211, 224, 235); [extension of pale colour pattern of head and mesosoma is very variable]	2
–	Mesosoma without pale pattern dorsally, at most with an ivory patch on mesoscutum medially (Fig. 174); first tergite of ♀ black, largely brownish-white or with a reddish-yellow lateral patch apically (Fig. 176); position of vein 1m-cu of fore wing variable (Fig. 172)	5
2	Legs mainly yellowish brown, including apex of hind femur (Figs 155, 218); apical half of first tergite entirely pale yellowish (both sexes; Figs 163, 228)	3
–	Legs mainly black or dark brown (except pale trochanter and trochantellus; Fig. 231), **if** sometimes intermediate (Fig. 207) then apex of hind femur dark brown (Fig. 214); apical half of first tergite partly black (Figs 184, 239)	4
3	Vertex with large yellow patch medio-posteriorly (Fig. 222); head dorsally and mesoscutum coarsely punctate (Figs 222, 226); posterior half of propodeum obliquely rugose (Fig. 226); spiracle of propodeum distinctly protruding (Fig. 227); outer side of hind coxa (except base) yellowish brown; hind tibia (except basal quarter) and tarsus dark brown; first tergite robust and with median groove (Fig. 228); mesosoma laterally with extensive yellow pattern (Fig. 227) [Note. *Orthogonalys centrimaculata* Bischoff, 1951, from Vietnam runs here but has head largely smooth dorsally and mesoscutum very finely and densely rugulose or coriaceous, matt, outer side of hind coxa dark brown, hind femur largely blackish, hind tibia and tarsus yellowish brown; mesopleuron and metapleuron black and body about 15 mm]	*Orthogonalys formosana* Teranishi, 1931
–	Vertex black medio-posteriorly (Fig. 157); head dorsally and mesoscutum largely smooth, punctulate (Fig. 157); posterior half of propodeum largely smooth except some transverse rugae (Fig. 161); spiracle of propodeum slightly protruding (Fig. 162); outer side of hind coxa largely dark brown; hind tibia and tarsus yellowish brown; first tergite less robust and without median groove (Fig. 163); mesosoma laterally with limited yellow pattern (Fig. 162) [Note. Close to *Orthogonalys albomaculata* Bischoff, 1951 from N. India but has propodeum coarsely reticulate, pronotal side crenulate medially, third submarginal cell of fore wing comparatively large and propodeum of ♂ with large ivory patch].	*Orthogonalys cheni* sp. n.
4	Posterior half of propodeum coarsely transversely rugose (Fig. 237); fifth and sixth metasomal tergites more or less ivory medio-apically (Fig. 239); comparatively robust species (Fig. 231); [medially third sternite 0.6–0.7 times as long as second sternite (Fig. 241); mesoscutum very densely to rather sparsely sculptured, as striations of supra-antennal elevations; face and temple usually with small pale patches, but sometimes entirely absent]	*Orthogonalys robusta* sp. n.
–	Posterior half of propodeum largely smooth (Fig. 182); fifth and sixth tergites dark brown or black medio-apically (Figs 181, 193); slender species (Figs 181, 186, 196, 207); medially third sternite 0.8–1.0 times as long as second sternite (Figs 185, 194, 206, 217), rarely less; [first tergite with pair of pale spots (♀) or black (♂); medially third sternite 0.6–1.0 times as long as second sternite and apical half of mandible reddish brown in Japanese specimens, and 0.6–0.7 times and apical half mainly ivory in Chinese specimens]	*Orthogonalys elongata* Teranishi, 1929
5	Third submarginal cell about 0.3 times as long as second submarginal cell; antenna with a pale brown band subapically (13^th^–15^th^ segments); [scutellum shiny and sparsely punctate; vein 1m-cu of fore wing connected to second submarginal cell; only ♂; Japan]	*Orthogonalys fukuiensis* (Tsuneki, 1991)
–	Third submarginal cell 0.4–0.5 times as long as second submarginal cell; antenna without a pale band; [area besides and behind lateral ocellus smooth; mandible largely ivory; anterior 0.7 of first metasomal tergite and anterior 0.2 of second tergite nearly black; fore femur orange-brown or brownish-yellow; pterostigma brown]	*Orthogonalys clypeata* sp. n.

### Orthogonalys cheni sp. n.

http://zoobank.org/7A5EDFC7-83E5-4800-BAD3-28234A469229

http://species-id.net/wiki/Orthogonalys_cheni

Figs 155 – 167

#### Type material.

Holotype, ♀ (ZJUH) [China:] Zhejiang, West Mt. Tianmu, Xianrending, 2–4.VI.1990, Zu-hua Shi, 902316”. Paratypes: 4 ♂ (ZJUH), “[China:] Zhejiang, Mt. Tianmu, 18.VI.1983, Yun Ma, 831073, 831375; id., but 21.VII.1987, Xiao-ming Lou, 874118; id., but 1998, Ming-shui Zhao, 998359”; 3 ♀ + 13 ♂ (ZJUH, RMNH), “[China:] Zhejiang, West Mt. Tianmu, Laodian–Xianrending, 1250–1506 m, 17–18.VI.1988, Xue-xin Chen, 891504, 891770, 891914, 891251, 891771, 891772; id., but Jun-hua He, 890471, 890480, 890612, 890350, 891101, 890492; id., but Xin-geng Wang, 892613, 892754, 892859; id., but Yan-ming Chen, 892384”; 8 ♀ + 40 ♂ (ZJUH, RMNH), “[China:] Zhejiang, West Mt. Tianmu, Xianrending, 2–4.VI.1990, Jun-hua He, 904928, 904973, 904975, 904932, 905254, 905201; id., but Zu-hua Shi, 901514, 902370, 901691, 901700, 902551, 901403, 901855, 902179, 902546, 902178, 901974, 901975, 901434, 902180, 902319, 901856, 902253; id., but Xin-geng Wang, 902978, 903022, 902988, 902871, 902992, 902704, 902705, 902760, 902743, 903882, 903314, 903881; id., but Yong-gen Lou, 900249, 900271, 900316, 900862, 900311; id., but Hai-jun Hu, 901241, 901079, 901090, 901314, 901082, 901238; id., but 14.VII.1998, Ming-shui Zhao, MT, 992457, 200010686; id., but 20.VII.1998, 992654”; 1 ♀ + 1 ♂ (ZJUH, RMNH), “[China:] Zhejiang, West Mt. Tianmu, 16–18.V.1990, Shi-jun Guo, 883038; id., but 31.V.1998, Xue-xin Chen, 980329”; 1 ♂ (ZJUH), “[China:] Hubei, Qianjiaping, 26.VIII.1982, Shang-bo Shi, 870104”; 1 ♂ (SCAU), “[China:] Sichuan, Wanglang National Nature Reserve, 16.VII.2006, Yi-ping Wang, SCAU 253”; 1 ♂ (SCAU), “[China:] Sichuan, Wolong National Nature Reserve, 20–21.VII.2006, Yi-ping Wang, 20115697”.

#### Diagnosis.

Vertex black medio-posteriorly (Fig. 157); head dorsally and mesoscutum largely smooth, punctulate (Figs 157, 161); mesosoma laterally with limited pale yellow or ivory pattern (Fig. 161); posterior half of propodeum largely smooth except some transverse rugae (Fig. 161); spiracle of propodeum slightly protruding (Fig. 162); vein 1m-cu of fore wing connected to first submarginal cell (Fig. 159); legs (including hind tibia and tarsus) mainly yellowish brown, but outer side of hind coxa (except base) largely dark brown (Fig. 162); first tergite less robust and without median groove, its apical half of first tergite entirely pale yellowish (both sexes; Figs 163, 167); medially third sternite half as long as second sternite (Fig. 165).

**Figures 155–157.**
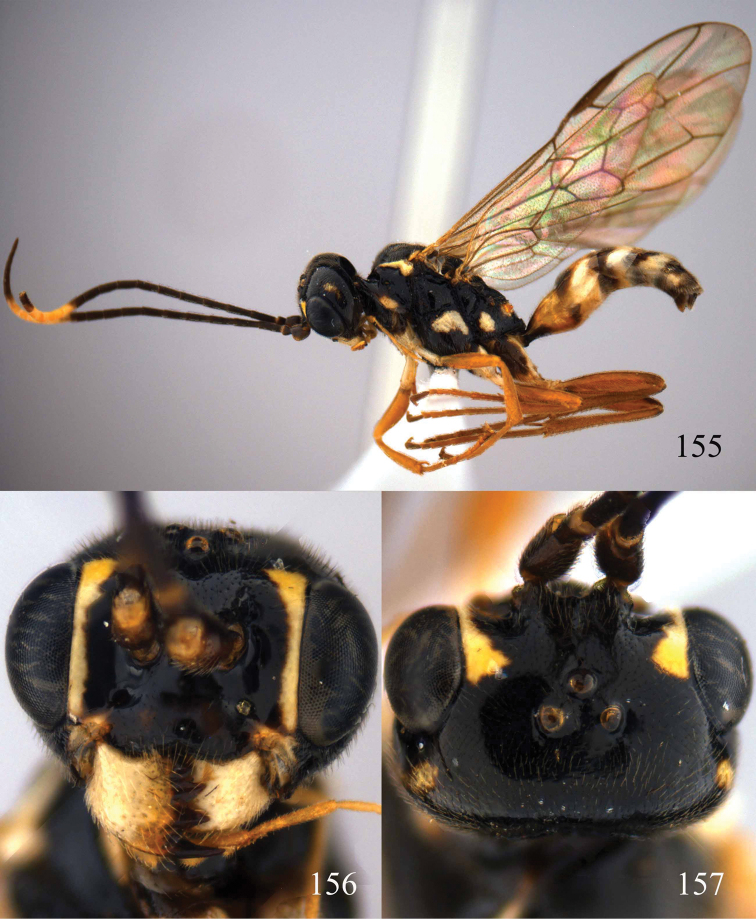
*Orthogonalys cheni* sp. n., holotype, female. **155** Habitus lateral **156** head anterior **157** head dorsal.

**Figures 158–165. F32:**
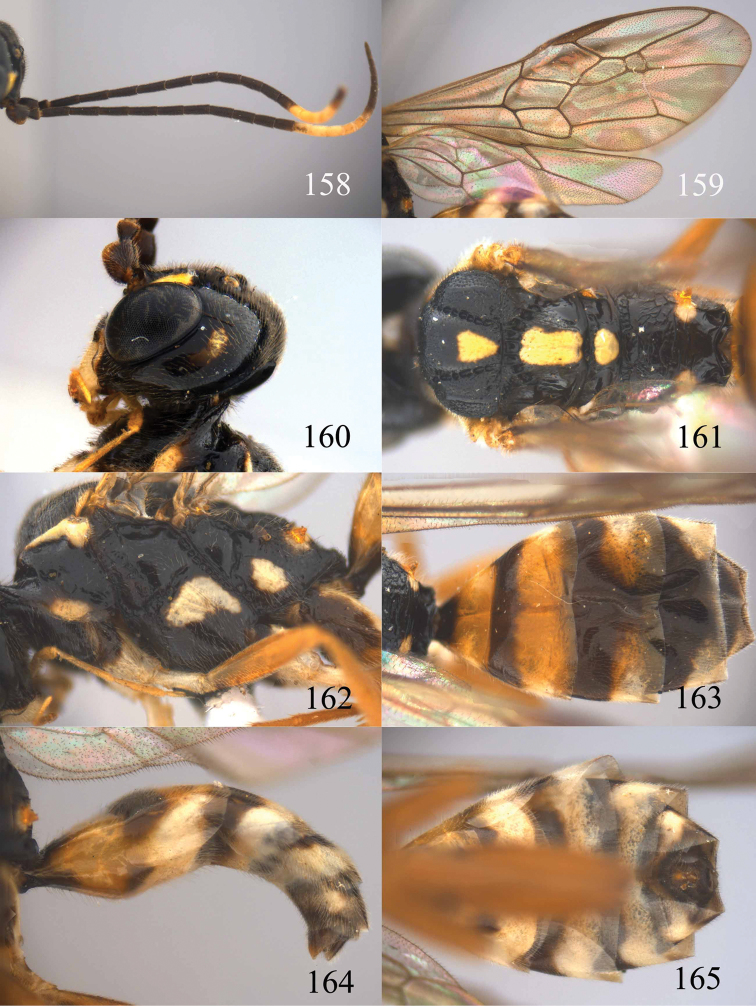
*Orthogonalys cheni* sp. n., holotype, female. **158** Antennae **159** fore and hind wings **160** head lateral **161** mesosoma dorsal **162** mesosoma lateral **163** metasoma dorsal **164** metasoma lateral **165** metasoma ventral.

**Figures 166–167. F33:**
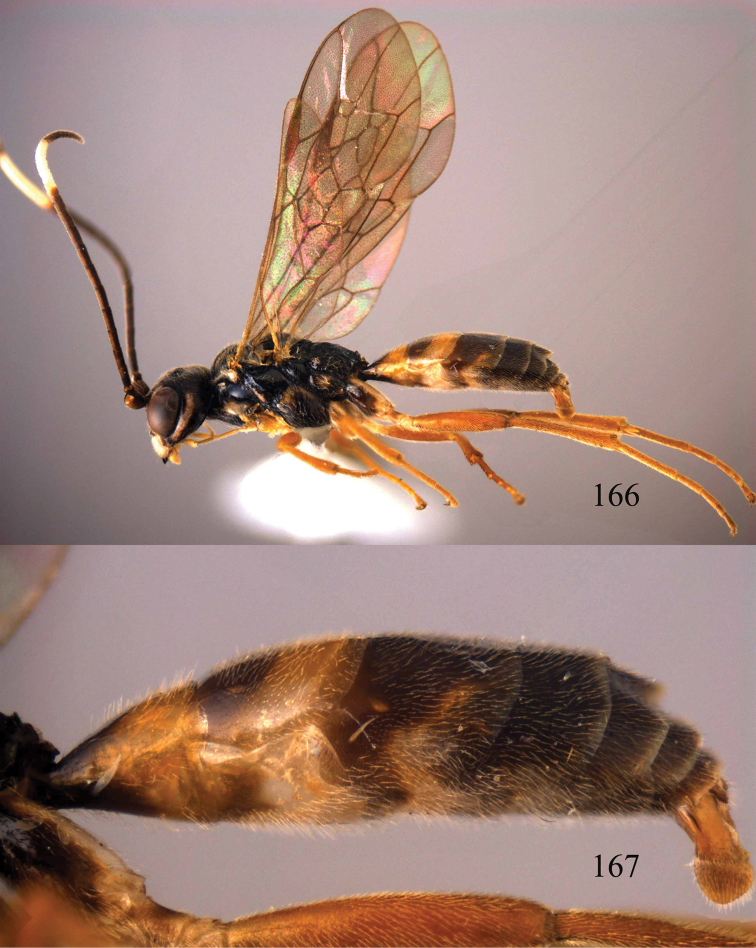
*Orthogonalys cheni* sp. n., paratype, male from Zhejiang. **166** Habitus lateral **167** metasoma lateral.

#### Description.

Holotype, ♀, length of body 6.9 mm (of fore wing 6.1 mm).

*Head*. Antenna with 25 segments; frons, vertex and temple smooth with sparse superficial punctures; head subparallel-sided behind eyes, eye in dorsal view 1.3 times as long as temple (Fig. 157); occipital carina weakly developed and weakly crenulate dorsally (Fig. 157); supra-antennal elevations medium-sized (about 0.4 times as long as scapus), outer side subvertical and largely smooth except for sparse fine punctures (Fig. 157); clypeus slightly concave and thick medio-ventrally.

*Mesosoma*. Length of mesosoma 1.5 times its height (Fig. 162); mesopleuron below transverse mesopleural groove smooth, above groove mainly smooth with longitudinal foveolae medially; transverse mesopleural groove wide, deep and coarsely crenulate; notauli wide, deep and coarsely crenulate; middle lobe of mesoscutum densely punctate anteriorly, largely smooth with sparse fine punctures posteriorly, lateral lobes densely punctate rugose with a narrow mid-longitudinal furrow (Fig. 161); scutellar sulcus wide, both medially and laterally and coarsely crenulate; scutellum mainly smooth medially, rather flat and anteriorly near level of scutellum (Fig. 161); metanotum medially slightly convex, not protruding and smooth (Fig. 161); propodeum irregularly rugulose antero-medially, transversely striate medially, antero-lateral corner and posterior half largely smooth (Fig. 161); posterior propodeal carina thick lamelliform and hardly arched, foramen medially 0.2 times higher than wide basally.

*Wings*. Fore wing: length of vein 1-M 2.2 times as long as vein 1-SR (Fig. 159).

*Metasoma*. First tergite 0.5 times as long as apically wide, smooth and without depression antero-medially (Fig. 163); other tergites and sternites smooth to superficially coriaceous and shiny (Fig. 163); third sternite as long as second sternite (Fig. 165); hypopygium triangular in ventral view (Fig. 165).

*Colour*. Black; mandible largely (but teeth dark brown), entire inner orbita narrowly, narrow stripes behind eyes posteriorly on vertex, large posterior spot on middle lobe of mesoscutum, broad median longitudinal stripe on scutellum, large median spot on metanotum, stripe on upper pronotum and lower pronotum, broad patch on lower mesopleuron, small spot on metapleuron, large lateral spots on propodeum, coxae dorsally, fore and mid trochanters and trochantelli, postero-lateral margin of second–fifth tergites and metasoma ventrally largely, ivory; clypeus black; metasoma dorsally largely dark brown, apical 0.7 of first and second tergite brownish; anterior margin of sternites with narrow transverse dark brown band; palpi yellowish brown; antenna black with a small subapical ivory band; coxae dark brown ventrally; remainder of legs yellowish brown; pterostigma dark brown; wing membrane subhyaline.

*Variation*. Length of body 5.8–7.0 mm, of fore wing 5.1–6.2 mm; ivory stripe on posterior vertex reduced into small spot or absent; lateral ivory spots on propodeum large or reduced; ivory patch on lower mesopleuron small; metasoma largely dark brown to black with first and second tergites and sternites largely yellowish brown; clypeus with pair of ivory spots laterally; legs darker; length of vein 1-M of fore wing 2.0–2.3 times as long as vein 1-SR.

*Male*. Length of body 5.6–8.1 mm, of fore wing 4.5–6.3 mm; antenna with 24–25 segments; ivory stripe along inner orbita narrow or broad; ivory stripe along outer orbita reduced or extending to malar space; clypeus largely dark brown with pair of ivory spots, largely ivory or entirely ivory; upper mesopleuron with ivory spot or absent; propodeum with large lateral spots or absent; legs and metasoma similar to that of female; genitalia extruded (Fig. 167).

#### Biology.

Unknown. Collected in June–August at 1250–1506 m.

#### Distribution.

China (Zhejiang, Hubei, Sichuan).

#### Etymology.

Named after the late Prof. Dr Sicien H. Chen (Beijing), in honour of his great contribution to entomology in China and to biology in general.

### Orthogonalys clypeata sp. n.

http://zoobank.org/981D7436-1224-4B41-9F51-AA57A028F1D8

http://species-id.net/wiki/Orthogonalys_clypeata

Figs 168 – 180

#### Type material.

Holotype, ♀ (ZJUH) “[China:] Guizhou, Suiyang, Kuankuoshui National Nature Reserve, 4.VI.2010, Jie Zeng, 20115981”. Paratypes: 1 ♀ (ZJUH) topotypic and same date, but 20115980; 1 ♂ (ZJUH) “[China:] Ningxia, Mt. Liupan, Sutai, 24.VI.2008, MT, Jing-xian Liu, 200800477”; 1 ♀ (IZCAS) “[China:] Shaanxi, Ningshan, Huoditang, 1580–1650 m, 27.VI.1999, De-cheng Yuan, SCAU 347”; 1 ♂ (SCAU) “[China:] Sichuan, Wanglang National Nature Reserve, 24.VII.2006, Yi-Ping Wang, SCAU 268”; 4 ♂ (ZJUH, RMNH) “[China:] Yunnan, Dali, Yunlong, 3.VI.2009, Jiang-li Tan, 200906671, 200906673, 200906677, 200906678”.

#### Diagnosis.

Antenna without ivory band subapically (Fig. 171); occipital carina weakly developed and smooth dorsally (Fig. 170); frons moderately shiny and partly superficially punctate (Fig. 169); area besides and behind lateral ocellus smooth; supra-antennal elevations comparatively low; clypeus strongly convex ventro-medially; mandible largely ivory; scutellum densely finely punctate and sometimes with some fine longitudinal carinae, without longitudinal depression and moderately shiny (Fig. 174); mesosoma without pale pattern dorsally, mesoscutum black medially (Fig. 174); third submarginal cell 0.4–0.5 times as long as second submarginal cell; pterostigma brown; fore femur orange-brown or brownish-yellow; anterior 0.7 of first metasomal tergite and anterior 0.2 of second tergite nearly black (Fig. 176).

**Figures 168–170. F34:**
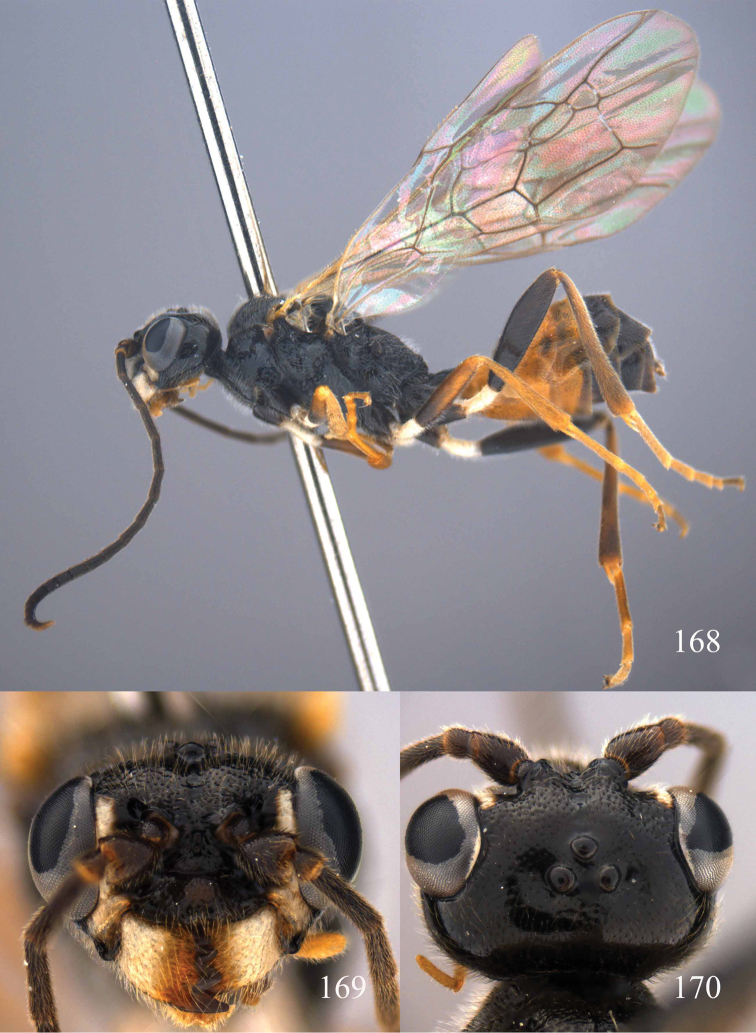
*Orthogonalys clypeata* sp. n., holotype, female. **168** Habitus lateral **169** head anterior **170** head dorsal.

**Figures 171–178. F35:**
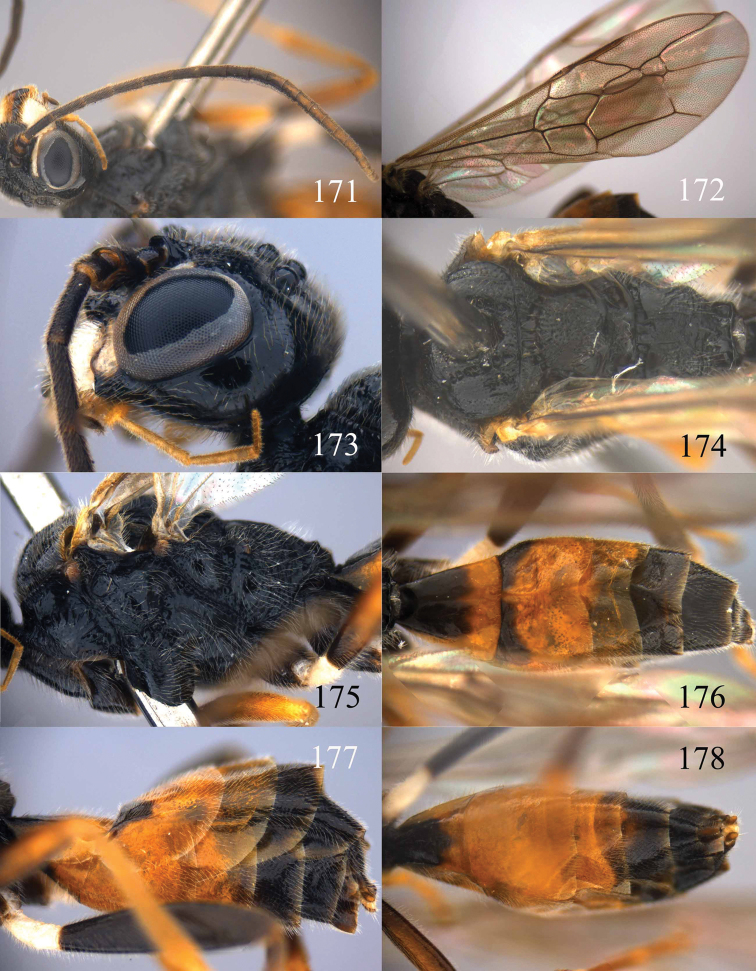
*Orthogonalys clypeata* sp. n., holotype, female. **171** Antenna **172** fore wing **173** head lateral **174** mesosoma dorsal **175** mesosoma lateral **176** metasoma dorsal **177** metasoma lateral **178** metasoma ventral.

**Figures 179–180. F36:**
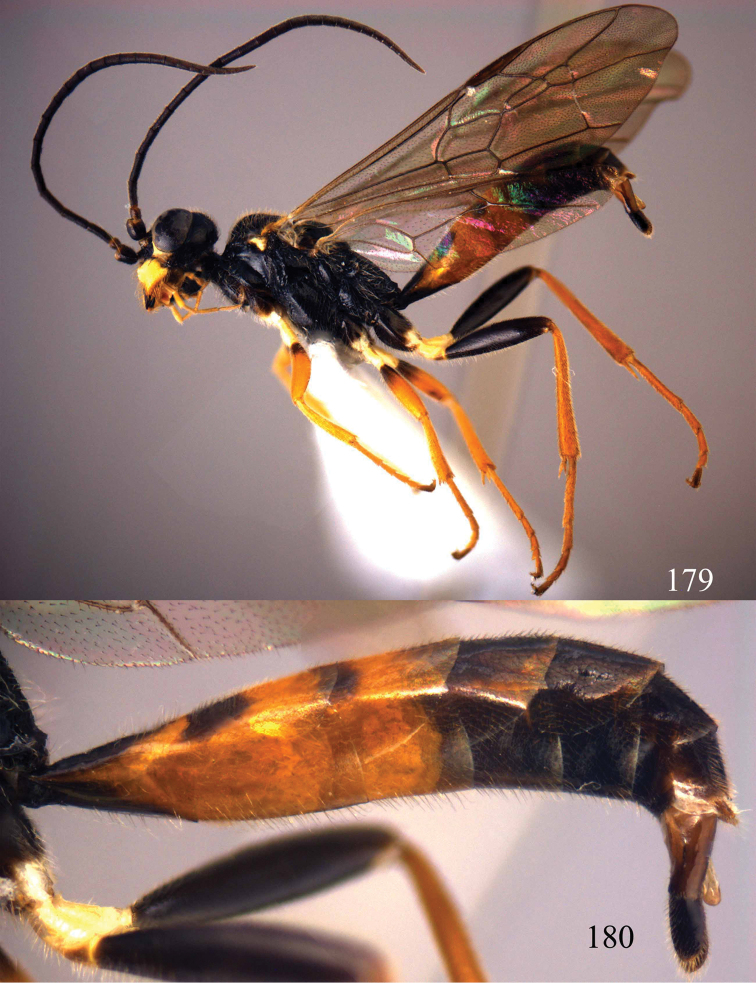
*Orthogonalys clypeata* sp. n., paratype, male. **179** Habitus lateral **180** metasoma lateral.

#### Description.

Holotype, female, length of body 7.6 mm (of fore wing 6.9 mm).

*Head*. Antenna with 24 segments; frons densely and finely punctate; vertex largely smooth with sparse fine punctures behind stemmaticum (Fig. 170); temple smooth (Fig. 173); head gradually narrowed behind eyes, eye in dorsal view 1.3 times as long as temple (Fig. 170); occipital carina weakly developed and smooth dorsally (Fig. 170); supra-antennal elevations medium-sized (about 0.3 times as long as scapus), outer side subvertical and rugose (Fig. 170); clypeus slightly concave and strongly convex medio-ventrally.

*Mesosoma*. Length of mesosoma 1.4 times its height (Fig. 175); mesopleuron below transverse mesopleural groove largely smooth with sparse oblique striations, above groove rugose anteriorly and smooth posteriorly; transverse mesopleural groove wide, deep and coarsely crenulate; notauli wide, deep and coarsely crenulate; mesoscutum densely punctate rugose, lateral lobe with a narrow mid-longitudinal furrow (Fig. 174); scutellar sulcus wide, both medially and laterally and coarsely crenulate; scutellum densely punctate rugose, rather flat and anteriorly near level of scutellum (Fig. 174); metanotum medially slightly protruding, obtuse and rugose (Fig. 174); propodeum antero-laterally smooth, irregularly rugose medially and transversely areolate posteriorly (Fig. 174); posterior propodeal carina thick lamelliform and hardly arched, foramen medially 0.3 times higher than wide basally.

*Wings*. Fore wing: length of vein 1-M 2.3 times as long as vein 1-SR (Fig. 172).

*Metasoma*. First tergite 0.9 times as long as apically wide, smooth and without depression medially (Fig. 176); other tergites and sternites smooth to superficially coriaceous and shiny (Fig. 176); third sternite about 0.5 times as long as second sternite (Fig. 178); hypopygium truncate in ventral view (Fig. 176).

*Colour*. Head black with inner orbita ivory up to malar space; mesosoma black; metasoma largely dark brown to nearly black, posterior 0.3 of first tergite and sternite, posterior 0.8 of second tergite, entirely third tergite and second sternite, anterior half of third tergite reddish-yellow; mandible largely ivory with teeth reddish-yellow to dark brown; palpi yellowish brown; antenna black to dark brown; trochanters and trochantelli ivory, coxae and hind femur black, remainder of legs yellowish brown with tarsi paler; pterostigma brown; wing membrane subhyaline.

*Variation*. Length of body 7.8–9.6 mm, of fore wing 6.9–8.4 mm; frons sparsely and superficially punctate; vertex with small ivory spots posteriorly; clypeus with small yellowish brown spot medially or ivory ventrally; length of vein 1-M of fore wing 2.1–2.3 times as long as vein 1-SR.

*Male*. Length of body 6.1–8.0 mm, of fore wing 5.2–6.9 mm; antenna with 24 segments; frons densely and coarsely or finely punctate; clypeus entirely ivory; colour of metasoma similar to female; genitalia extruded (Fig. 180).

#### Biology.

Unknown. Collected in June–July at 1580–1650 m.

#### Distribution.

China (Ningxia, Shaanxi, Sichuan, Guizhou, Yunnan).

#### Etymology.

Named after the ventro-medially strongly convex clypeus: from “*clypeus*” (Latin for “shield”).

#### Notes.

It is close to *Orthogonalys hagoromonis* Teranishi, 1929, from Japan, but it can be separated from the latter by the yellow or ivory inner orbita and the lower supra-antennal elevations.

### Orthogonalys elongata

Teranishi, 1929

http://species-id.net/wiki/Orthogonalys_elongata

**Figures 181–185. F37:**
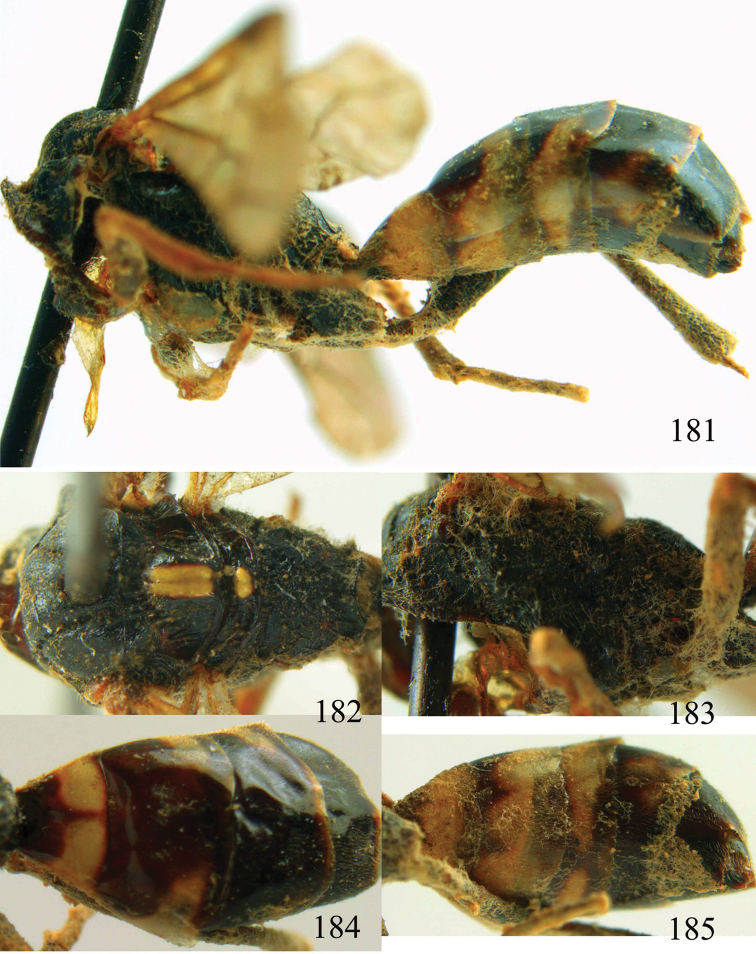
*Orthogonalys elongata* Teranishi, 1929, holotype, female. **181** Habitus lateral (without head) **182** mesosoma dorsal **183** mesosoma lateral **184** metasoma dorsal **185** metasoma ventral.

Figs 181 [Fig F38] [Fig F39] [Fig F40] [Fig F41] [Fig F42] [Fig F43] –219

Orthogonalos elongata Teranishi, 1929: 146; Marshakov 1981: 105; Tsuneki 1991: 20.
Satogonalos elongata ; Weinstein and Austin 1991: 424.
Orthogonalys elongata ; Carmean and Kimsey 1998: 54; Bennett and Lelej 2003: 8.
Orthogonalos debilis Teranishi, 1929: 144; Marshakov 1981: 105. Synonymized by Tsuneki 1991.
Satogonalos debilis ; Weinstein and Austin 1991: 424; Lelej 1995: 14.
Orthogonalos hirasana Teranishi, 1929: 145; Weinstein and Austin 1991: 424. Synonymized by Marshakov 1981.

#### Type material.

Holotype of *Orthogonalys elongata*, ♀ (OMNH, head missing), “[Japan], Sapporo, Maruyama, 24.VII.1923, C. T[eranishi]”, “Allotype, *Orthogonalos elongata*”. Holotype of *Orthogonalos debilis*, ♀ (OMNH), “[Japan], Yamanashi, Mt. Minobu, 12.VI.1928, K. Sato”, “Allotype, *Orthogonalos debilis*”. Holotype of *Orthogonalos hirasana*, ♂ (OMNH), “[Japan], Shiga-ken, Mt. Hira, VII.1927, C. C. T[eranishi]”, “Holotype, *Orthogonalos hirasana*”.

#### Additional material.

1 ♂ (ZJUH), “[China:] Henan, Song County, Mt. Baiyun, 1400 m, 19.VII.2003, Yan-bing Shen, 20047315”; 1 ♀ (RMNH), “[China:] Henan, Neixiang County, Mt. Baotianman, 14.VII.1998, Yun Ma, 986459”; 2 ♀ (ZJUH), “[China:] Sichuan, Tianquan, Laba River, 15.VII.2006, Hong-ying Zhang, 200610725, 200610723”; 1 ♀ (ZJUH), “[China:] Tibet, Lage–Hangmi, 12.VI.2009, Jiang-li Tan, 200908322”.

#### Diagnosis.

**Figures 186–188. F38:**
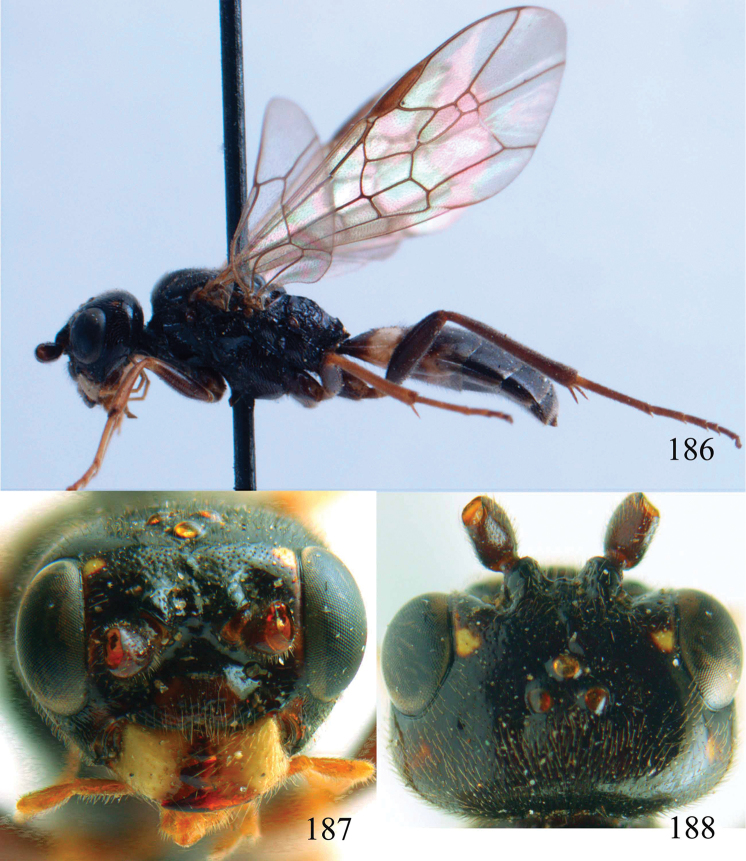
*Orthogonalos debilis* Teranishi, 1929, holotype, female. **186** Habitus lateral **187** head anterior **188** head dorsal.

Face and temple usually with small pale patches, but sometimes entirely absent; mesoscutum very densely to rather sparsely sculptured, as striations of supra-antennal elevations; mesosoma with linear ivory pattern dorsally (at least with large ivory patches on scutellum and metanotum; Figs 182, 190, 202, 213); posterior half of propodeum largely smooth (Figs 182, 190, 202, 213); vein 1m-cu of fore wing connected to first submarginal cell (Figs 195, 200, 211); legs mainly black or dark brown (except pale trochanter and trochantellus); apical half of first tergite with pair of pale spots (♀) or black (♂) (Figs 184, 192, 204, 215, 219); medially third sternite 0.8–1.0 times as long as second sternite (Figs 185, 194, 206, 217), rarely less; fifth and sixth metasomal tergites dark brown or black medio-apically (Figs 181, 192, 202, 215); comparatively slender species (Figs 181, 186, 196, 207).

**Figures 189–195. F39:**
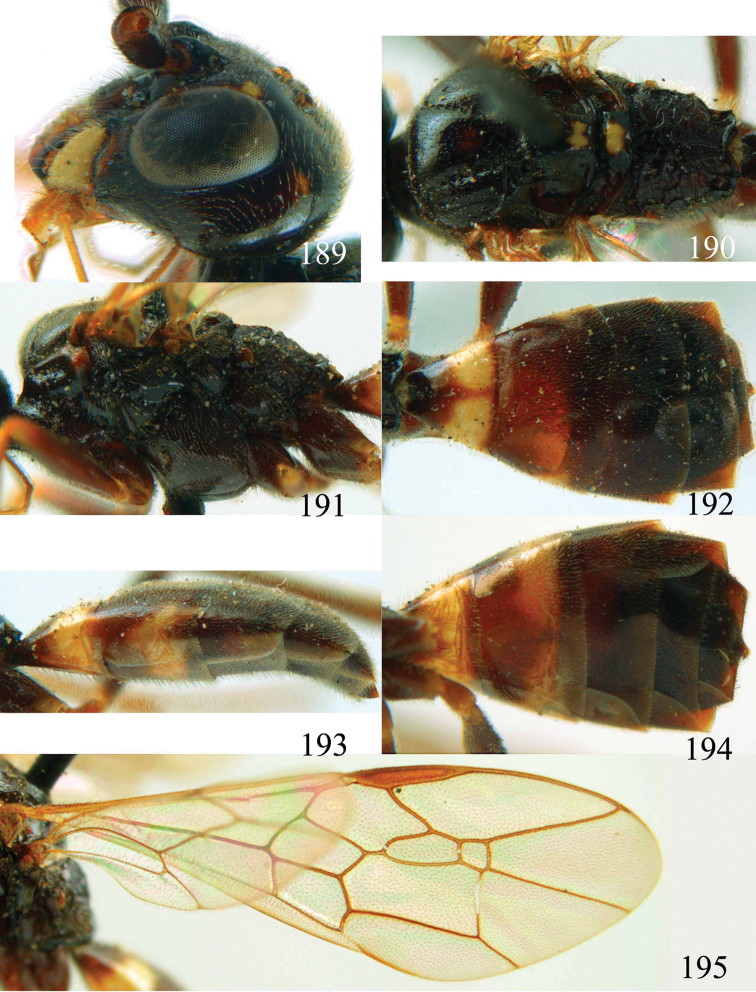
*Orthogonalos debilis* Teranishi, 1929, holotype, female. **189** Head lateral **190** mesosoma dorsal **191** mesosoma lateral **192** metasoma dorsal **193** metasoma lateral **194** metasoma ventral **195** fore wing.

**Figures 196–198. F40:**
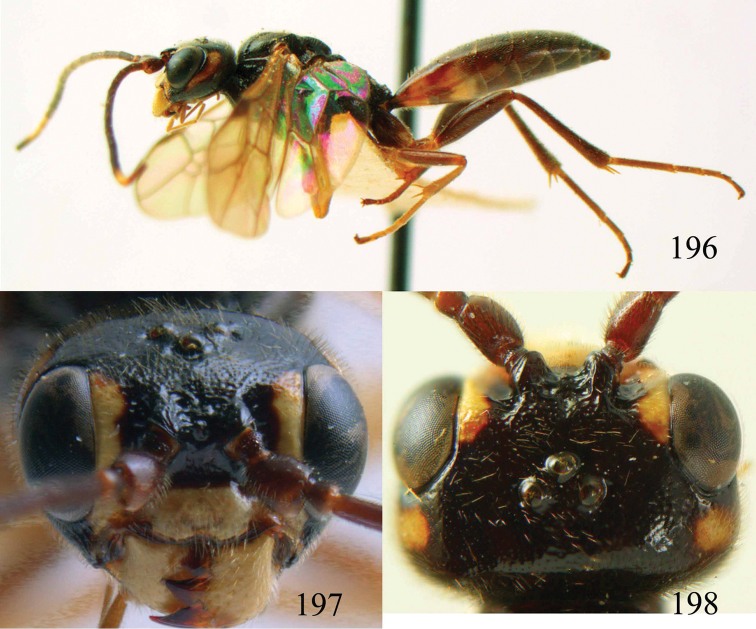
*Orthogonalos hirasana* Teranishi, 1929, holotype, male. **196** Habitus lateral **197** head anterior **198** head dorsal.

**Figures 199–206. F41:**
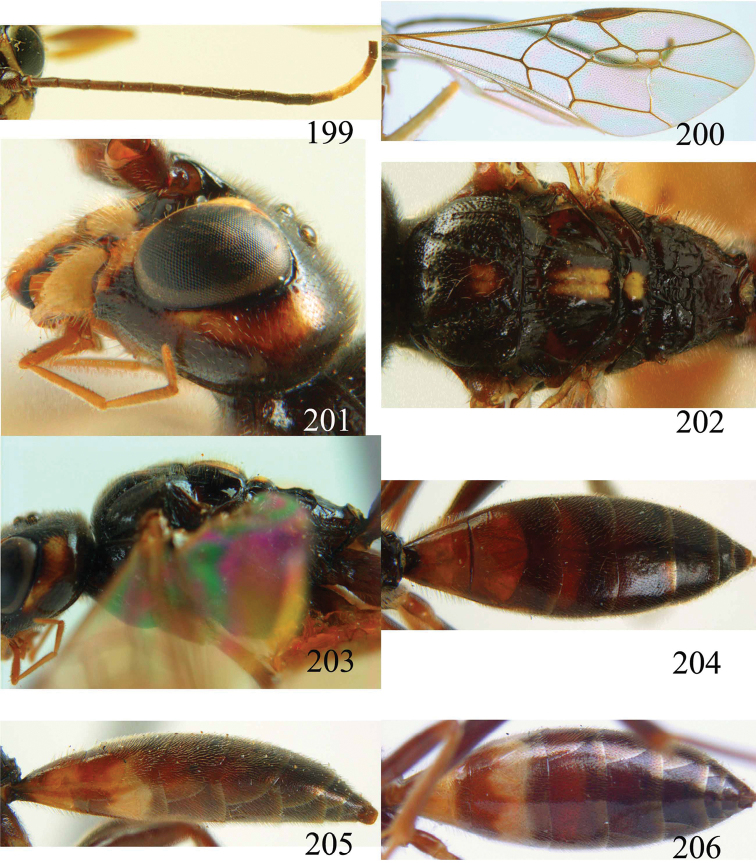
*Orthogonalos hirasana* Teranishi, 1929, holotype, male. **199** Antenna **200** fore wing **201** head lateral **202** mesosoma dorsal **203** mesosoma lateral **204** metasoma dorsal **205** metasoma lateral **206** metasoma ventral.

**Figures 207–209. F42:**
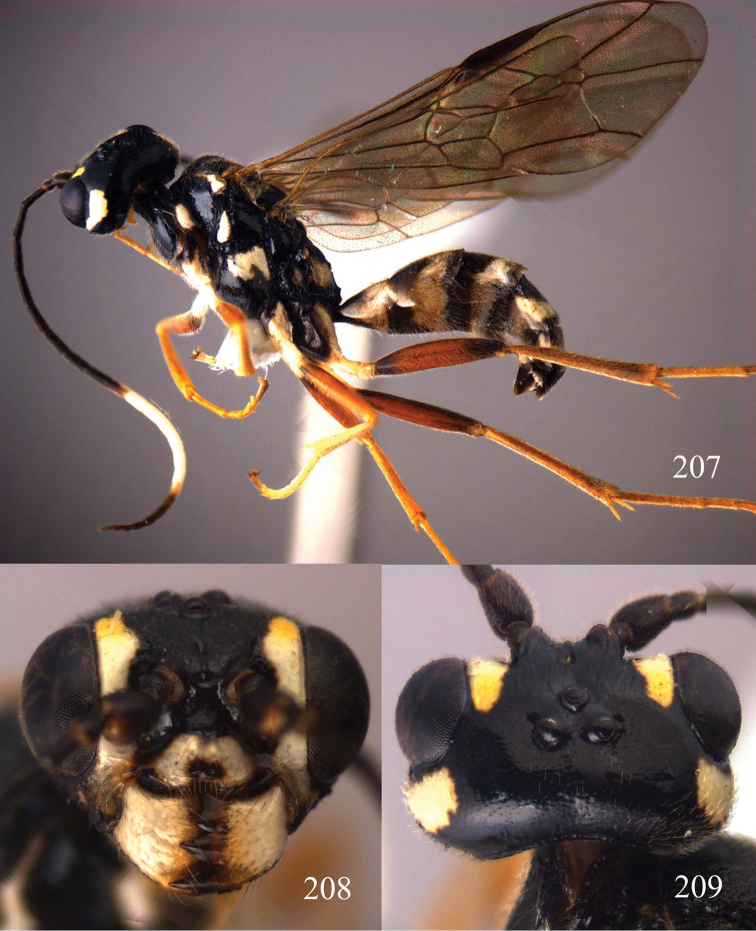
*Orthogonalys elongata* Teranishi, 1929, female from Sichuan. **207** Habitus lateral **208** head anterior **209** head dorsal.

**Figures 210–217. F43:**
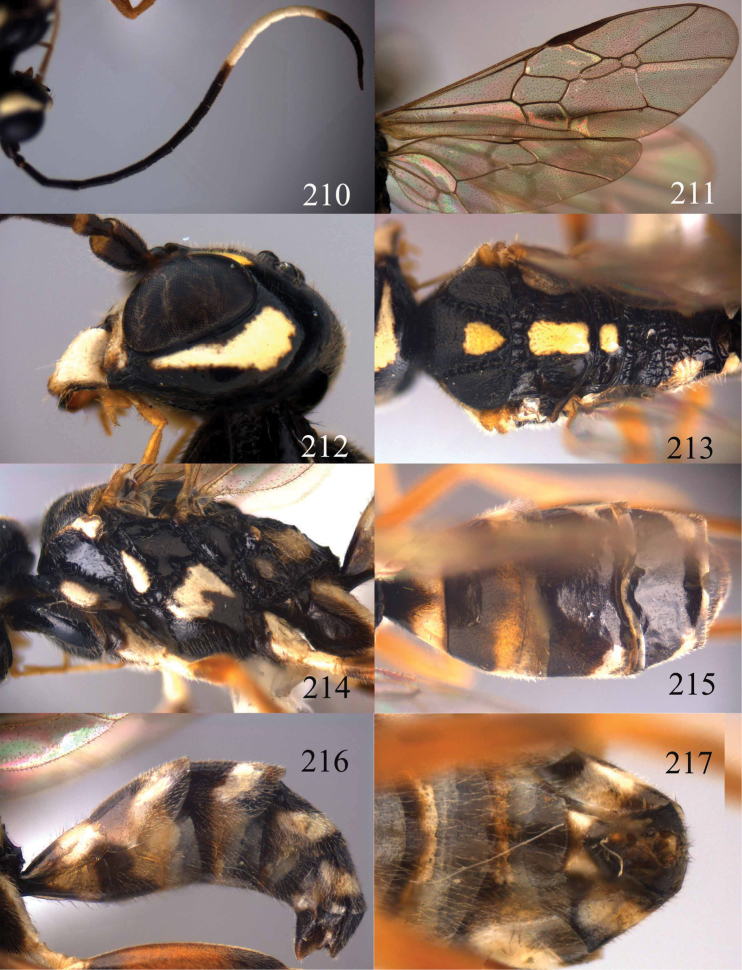
*Orthogonalys elongata* Teranishi, 1929, female from Sichuan. **210** Antenna **211** fore and hind wings **212** head lateral **213** mesosoma dorsal **214** mesosoma lateral **215** metasoma dorsal **216** metasoma lateral **217** metasoma ventral.

**Figures 218–219. F44:**
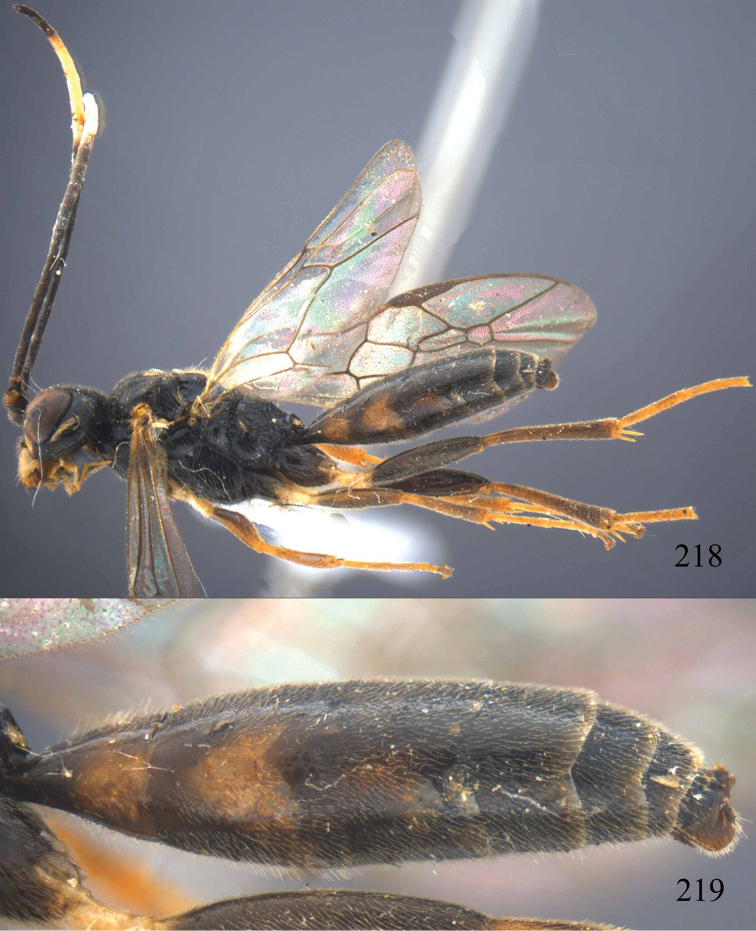
*Orthogonalys elongata* Teranishi, 1929, male from Henan. **218** Habitus lateral **219** metasoma lateral.

#### Description.

Redescribed after a female from Sichuan, length of body 7.4 mm (of fore wing 6.2 mm).

*Head*. Antenna with 25 segments; frons and vertex sparsely and superficially punctate; temple smooth; head subparallel-sided behind eyes, eye in dorsal view 1.2 times as long as temple (Fig. 209); occipital carina weakly developed and weakly crenulate dorsally (Fig. 209); supra-antennal elevations medium-sized (about 0.4 times as long as scapus), outer side subvertical and largely smooth except for sparse fine punctures (Fig. 209); clypeus slightly concave and thick medio-ventrally.

*Mesosoma*. Length of mesosoma 1.6 times its height (Fig. 214); mesopleuron below transverse mesopleural groove smooth, above groove mainly smooth with longitudinal foveolae medially; transverse mesopleural groove wide, deep and coarsely crenulate; notauli wide, deep and coarsely crenulate; middle lobe of mesoscutum densely punctate rugose anteriorly, largely smooth with sparse fine punctures posteriorly, lateral lobe densely punctate rugose with a narrow mid-longitudinal furrow (Fig. 213); scutellar sulcus wide, both medially and laterally and coarsely crenulate; scutellum mainly smooth, rather flat and anteriorly near level of scutellum (Fig. 213); metanotum medially slightly convex, not protruding and smooth (Fig. 213); anterior half of propodeum irregularly areolate or rugose, posterior half largely smooth with a longitudinal carina medially (Fig. 213); posterior propodeal carina thick lamelliform and hardly arched, foramen medially 0.3 times higher than wide basally.

*Wings*. Fore wing: length of vein 1-M 2.5 times as long as vein 1-SR (Fig. 211).

*Metasoma*. First tergite 0.5 times as long as apically wide, smooth and without shallow depression antero-medially (Fig. 215); other tergites and sternites smooth to superficially coriaceous and shiny (Figs 215, 217); third sternite about 0.6 times as long as second sternite (Fig. 217); hypopygium triangular in ventral view (Fig. 217).

*Colour*. Black; mandible largely (but teeth dark brown), broad stripes of inner and outer orbita, clypeus largely (except for median dark brown spot), large posterior spot on middle lobe of mesoscutum, broad median longitudinal stripe on scutellum, large median spot on metanotum, stripe on upper pronotum and lower pronotum, oblique stripe on upper mesopleuron and broad patch on lower mesopleuron, small spot on metapleuron, large lateral spots on propodeum, trochanters and trochantelli and outer side of coxae, posterior and lateral margin of all metasomal tergites and sternites, ivory or pale yellow; remainder of metasoma largely dark brown to nearly black; palpi yellowish brown; antenna black with a large ivory band subapically; fore and mid tibiae and all tarsi yellowish brown, remainder of legs dark brown to nearly black; pterostigma dark brown; wing membrane subhyaline.

*Variation*. Length of body 5.8–8.3 mm, of fore wing 4.7–7.4 mm; upper mesopleuron with large interrupted ivory patch or an oblique stripe or mesosoma laterally entirely (Japan) or largely black; ivory patch of mesoscutum present or absent; ivory patches on posterior and lateral margin of all tergites and sternites comparatively small or large; clypeus entirely ivory or with dark median spot; inner and outer orbita widely ivory but largely black in holotype of *Orthogonalos debilis*; hind coxa entirely dark brown or largely ivory dorsally; length of vein 1-M of fore wing 1.9–2.5 times as long as vein 1-SR.

*Male*. Length of body 7.4 mm, of fore wing 6.0 mm; antenna with 24 segments; ivory stripe along outer orbita reduced or wide (as in holotype of *Orthogonalos hirasana*); lateral mesosoma and propodeum entirely black; metasoma largely black, only with postero-lateral margin of first and second tergites and sternites dark brown (but absent in holotype of *Orthogonalos hirasana*); ventral half of clypeus ivory; genitalia extruded (Fig. 219).

#### Biology.

Unknown. Collected in June–July, up to 1400 m.

#### Distribution.

China (Henan, Sichuan, Tibet); Russia (Far East); Japan (Hokkaido, Honshu) (Lelej 2003).

### Orthogonalys formosana

Teranishi, 1931

http://species-id.net/wiki/Orthogonalys_formosana

Figs 220 –230

Orthogonalos formosana Teranishi, 1931: 9; Tsuneki 1991: 64.
Orthogonalys formosana ; Weinstein and Austin 1991: 421; Carmean and Kimsey 1998: 54.

#### Type material.

Holotype, ♀ (OPU), “[China:] Formosa, Nokosan, V.1922, M. Kato”, “Holotype *Orthogonalos formosana* Ter., ♀”.

#### Diagnosis.

Vertex with large yellow patch medio-posteriorly (Fig. 222); head dorsally and mesoscutum coarsely punctate (Figs 222, 226); mesosoma laterally with extensive pale yellow or ivory pattern (Fig. 227); posterior half of propodeum obliquely rugose (Fig. 227); spiracle of propodeum distinctly protruding (Fig. 227); vein 1m-cu of fore wing connected to first submarginal cell (Fig. 224); legs mainly yellowish brown, including outer side of hind coxa (except base) but hind tibia (except basal quarter) and tarsus dark brown (Fig. 230); first tergite robust and with median groove, its apical half of first tergite entirely pale yellowish (both sexes; Fig. 228); medially third sternite half as long as second sternite.

**Figures 220–222. F45:**
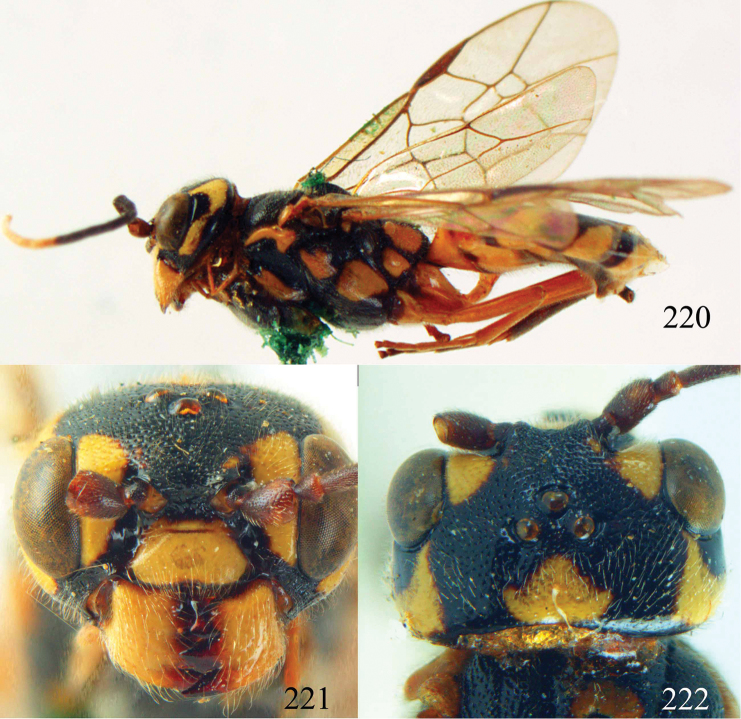
*Orthogonalys formosana* Teranishi, 1931, holotype, female. **220** Habitus lateral **221** head anterior **222** head dorsal.

**Figures 223–230. F46:**
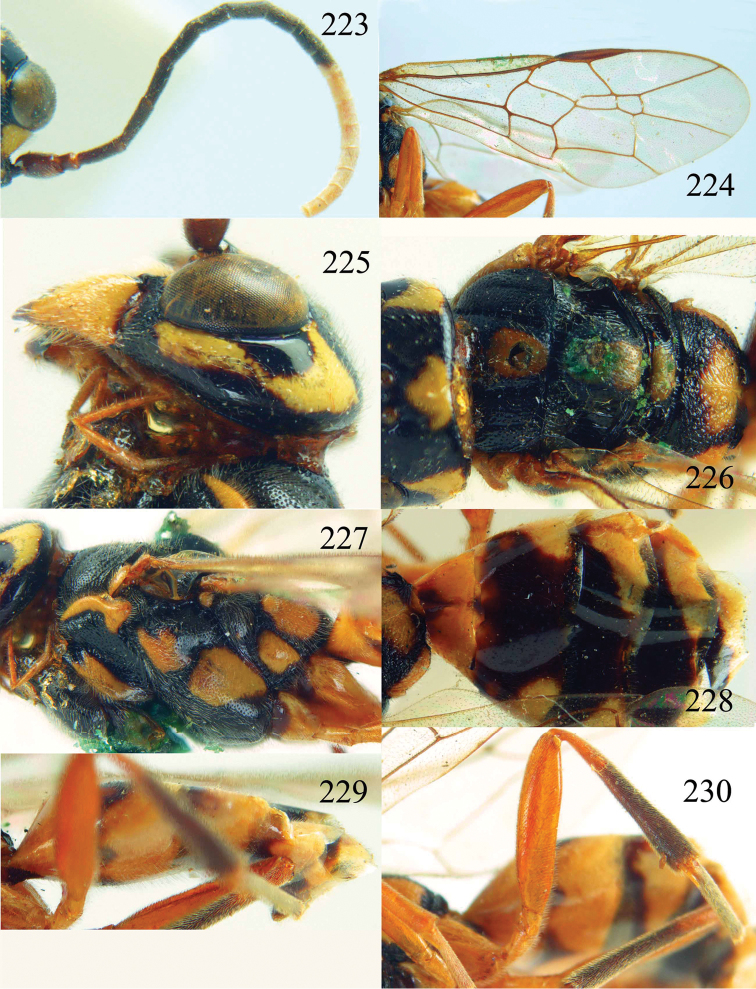
*Orthogonalys formosana* Teranishi, 1931, holotype, female. **223** Antenna **224** fore wing **225** head lateral **226** mesosoma dorsal **227** mesosoma lateral **228** metasoma dorsal **229** metasoma lateral **230** hind leg.

#### Description.

Holotype, ♀, length of body 9.0 mm (of fore wing 8.3 mm).

*Head*. Antenna incomplete, with 26 segments according to original description; frons and vertex coarsely punctate and partly with smooth interspaces equal to width of punctures or wider; temple smooth; head subparallel-sided behind eyes, eye in dorsal view 1.1 times as long as temple (Fig. 222); occipital carina narrow, non-lamelliform and smooth dorsally (Fig. 222); supra-antennal elevations medium-sized (about 0.5 times as long as scapus), outer side oblique and punctate, apically smooth and non-lamelliform (Fig. 222); clypeus nearly straight and thick medio-ventrally.

*Mesosoma*. Length of mesosoma 1.3 times its height (Fig. 227); postero-dorsal corner of pronotal side protruding far posteriorly (Fig. 227); mesopleuron below transverse mesopleural groove distinctly punctate with smooth interspaces about equal to diameter of punctures, above groove densely punctate and partly with rugae; transverse mesopleural groove anteriorly wide, deep and coarsely crenulate, narrow and finely crenulate posteriorly; notauli wide, deep and coarsely crenulate; mesoscutum coarsely punctate with wide smooth interspaces, lateral lobes with a narrow mid-longitudinal furrow (Fig. 226); scutellar sulcus wide and coarsely crenulate; scutellum mainly smooth except some punctures, rather convex and anteriorly above level of scutellum (Fig. 226); metanotum medially moderately convex and protruding, smooth (Fig. 226); propodeum irregularly reticulate-rugose, posteriorly partly smooth and median carina nearly complete (Fig. 226); posterior propodeal carina weakly developed and non-lamelliform and distinctly arched, foramen medially about as high as wide basally.

*Wings*. Fore wing: length of vein 1-M 2.7 times as long as vein 1-SR (Fig. 224); second submarginal cell 1.6 times longer than third cell.

*Metasoma*. First tergite 0.4 times as long as apically wide, smooth and with linear depression medially (Fig. 228); other tergites and sternites smooth to superficially coriaceous and shiny (Fig. 228); third sternite half as long as second sternite (Fig. 229); hypopygium truncate in ventral view.

*Colour*. Black; mandible, clypeus, face, curved patch behind eye dorsally, triangular patch behind stemmaticum, ventral half of outer orbita and inner orbita largely and widely, large posterior patch on middle lobe of mesoscutum and on scutellum, metanotum medially, large patches on propodeum medio-posteriorly and laterally, pronotal side dorsally and ventrally, two large patches on mesopleuron and large patch on metapleuron, first metasomal tergite and sternite largely, second tergite laterally, second sternite largely, second–fourth tergites latero-posteriorly, fifth and sixth tergites posteriorly, seventh tergite, third–fifth sternites laterally, pale yellow or ivory; remainder of metasoma dark brown; antenna black with a wide ivory band (apex of 10^th^ and 12^th^–19^th^ segments) subapically and scapus and pedicellus largely dark brown; fore leg missing; middle and hind legs brownish-yellow, but coxae narrowly basally, hind tibia (except basally) and hind tarsus dark brown; pterostigma and veins dark brown; wing membrane subhyaline.

*Male*. Unknown.

#### Biology.

Unknown. Collected in May.

#### Distribution.

China (Taiwan).

### Orthogonalys robusta sp. n.

http://zoobank.org/451FDA26-0EBB-4325-9350-678A526C19D6

http://species-id.net/wiki/Orthogonalys_robusta

Figs 231 –241

#### Type material.

Holotype, ♀ (SCAU) “[China:] Shaanxi, Mt. Taibai, 107°53.505’E, 34°07.430’N, 1580 m, 30.VI.1999, Xiao-yu Jiang, SCAU 272”. Paratypes: 2 ♀ (IZCAS) “[China:] Shaanxi, Ningshan, Dashuigou, 1500–1760 m, 30.VI.1999, De-cheng Yuan, 200105000, 200105001”; 1 ♀ (ZJUH) “[China:] Guangxi, Longsheng, Huaping, 25–26.VI.1982, Jun-hua He, No. 823513”; 1 ♀ (ZJUH) “[China:] 903839”.

#### Diagnosis.

**Figures 231–233. F47:**
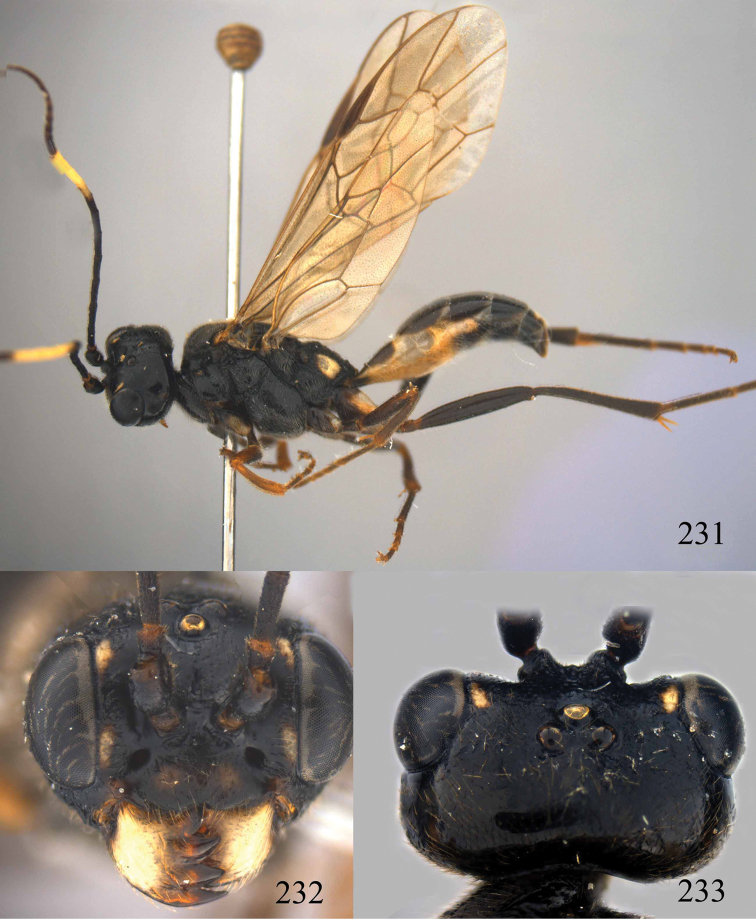
*Orthogonalys robusta* sp. n., holotype, female. **231** Habitus lateral **232** head anterior **233** head dorsal.

Face and temple usually with small pale patches, but sometimes entirely absent; mesoscutum very densely to rather sparsely sculptured, as striations of supra-antennal elevations; mesosoma with linear ivory pattern dorsally (at least with large ivory patches on scutellum and metanotum; Fig. 237); posterior half of propodeum coarsely transversely rugose (Fig. 237); vein 1m-cu of fore wing connected to first submarginal cell (Fig. 235); legs mainly black or dark brown (except pale trochanter and trochantellus; Fig. 231); apical half of first tergite partly black, with brownish or ivory apical transverse band (Fig. 239); medially third sternite 0.6–0.7 times as long as second sternite (Fig. 241); fifth and sixth metasomal tergites more or less ivory medio-apically (Fig. 239); comparatively robust species (Fig. 231).

**Figures 234–241.**
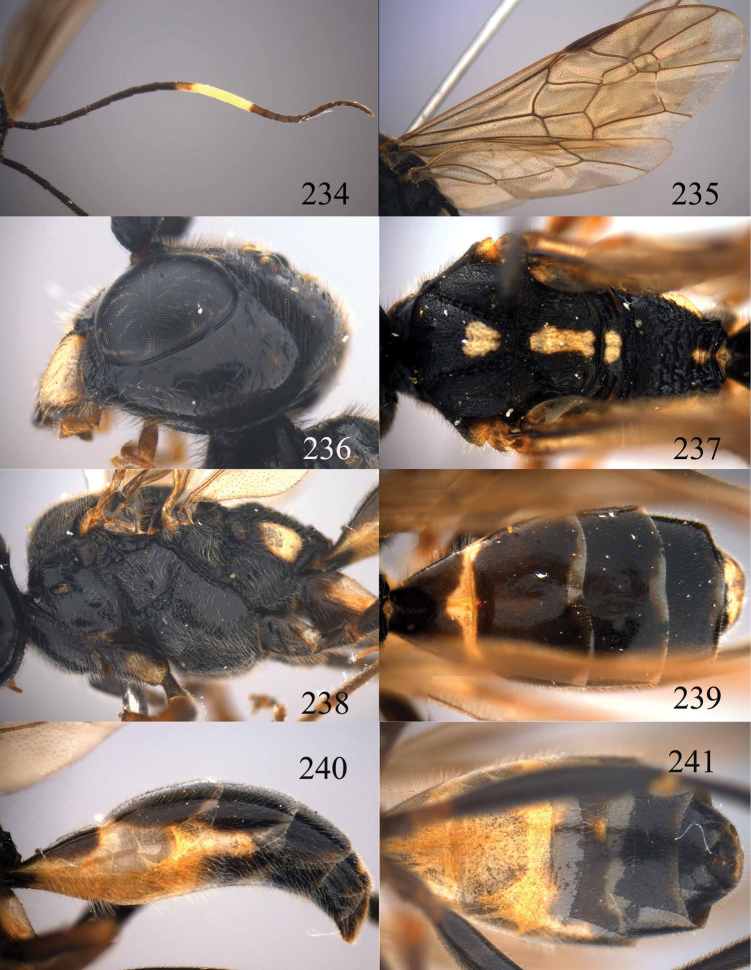
*Orthogonalys robusta* sp. n., holotype, female. **234** Antenna **235** fore and hind wings **236** head lateral **237** mesosoma dorsal **238** mesosoma lateral **239** metasoma dorsal **240** metasoma lateral **241** metasoma ventral.

#### Description.

Holotype, female, length of body 10.2 mm (of fore wing 9.4 mm).

*Head*. Antenna with 27 segments; frons densely and finely punctate (Fig. 232); vertex and temple smooth and shiny (Fig. 236); head subparallel-sided behind eyes, eye in dorsal view 0.9 times as long as temple (Fig. 233); occipital carina narrowly lamelliform with a weak short carina medio-dorsally (Fig. 233); supra-antennal elevations medium-sized (about 0.4 times as long as scapus), outer side subvertical and obliquely and weakly striate (Fig. 233); clypeus slightly concave and thick medio-ventrally.

*Mesosoma*. Length of mesosoma 1.6 times its height (Fig. 238); mesopleuron below transverse mesopleural groove somewhat transversely rugulose antero-ventrally and smooth postero-dorsally, above groove rugose anteriorly and smooth posteriorly; transverse mesopleural groove wide, deep and coarsely crenulate; notauli moderately wide, deep and coarsely crenulate; middle lobe of mesoscutum transversely punctate-rugose, lateral lobes mainly irregularly punctate rugose with narrow, smooth mid-longitudinal line (Fig. 237); scutellar sulcus wide, both medially and laterally and coarsely crenulate; scutellum longitudinally rugose, slightly convex and anteriorly near level of mesoscutum; metanotum medially slightly convex, not protruding and largely smooth with sparse fine punctures (Fig. 237); propodeum irregularly rugose anteriorly, transversely and more coarsely rugose posteriorly (Fig. 237); posterior propodeal carina thick lamelliform and slightly arched, foramen medially 0.2 times higher than wide basally.

*Wings*. Fore wing: length of vein 1-M 2.3 times as long as vein 1-SR (Fig. 235).

*Metasoma*. First tergite 0.7 times as long as apically wide, smooth and with distinct elliptical depression antero-medially (Fig. 239); second–sixth tergites smooth to superficially coriaceous and shiny (Fig. 239); sternites superficially coriaceous; third sternite about 0.4 times as long as second sternite (Fig. 241); hypopygium triangular in ventral view.

*Colour*. Black; mandible largely (but teeth orange-brown or black), two patches at inner orbita, clypeus faintly medially, large posterior spot on middle lobe of mesoscutum, broad median longitudinal stripe on scutellum, large median spot on metanotum, small posterior spot and large lateral spots on propodeum, narrow stripe on posterior margin of first metasomal tergite and anterior margin of second tergite, almost entirely fifth and sixth tergites and first and second sternites and antero-latero margin of third sternite, ivory; palpi yellowish brown; basal half of antenna black, medially ivory and dark brown apically (Fig. 234); femur, tibia and tarsus of fore leg and tarsus of hind leg yellowish brown, remainder of legs dark brown to nearly black; pterostigma of fore wing dark brown, remainder of wing membrane subhyaline.

*Variation*. Length of body 9.0–12.6 mm, of fore wing 7.7–10.8 mm; antenna of ♀ with 27–29 segments; frons without ivory stripes or stripes continuous, clypeus with pair of ivory spots, entirely ivory or black, vertex and outer orbita with ivory spots or stripes; pronotal side with ivory stripe antero-dorsally; propodeum entirely black; antenna medially orange-yellow; legs darker or paler; length of vein 1-M of fore wing 1.2–3.1 times as long as vein 1-SR.

*Male*. Unknown.

#### Biology.

Unknown. Collected in June and August at 1500–1760 m.

#### Distribution.

China (Shaanxi, Guangxi).

#### Etymology.

Named after the comparatively robust body of this species: from “*robustus*” (Latin for “hard and strong like an oak”).

### Pseudogonalos

Schulz, 1906

http://species-id.net/wiki/Pseudogonalos

**Figures 242–244.**
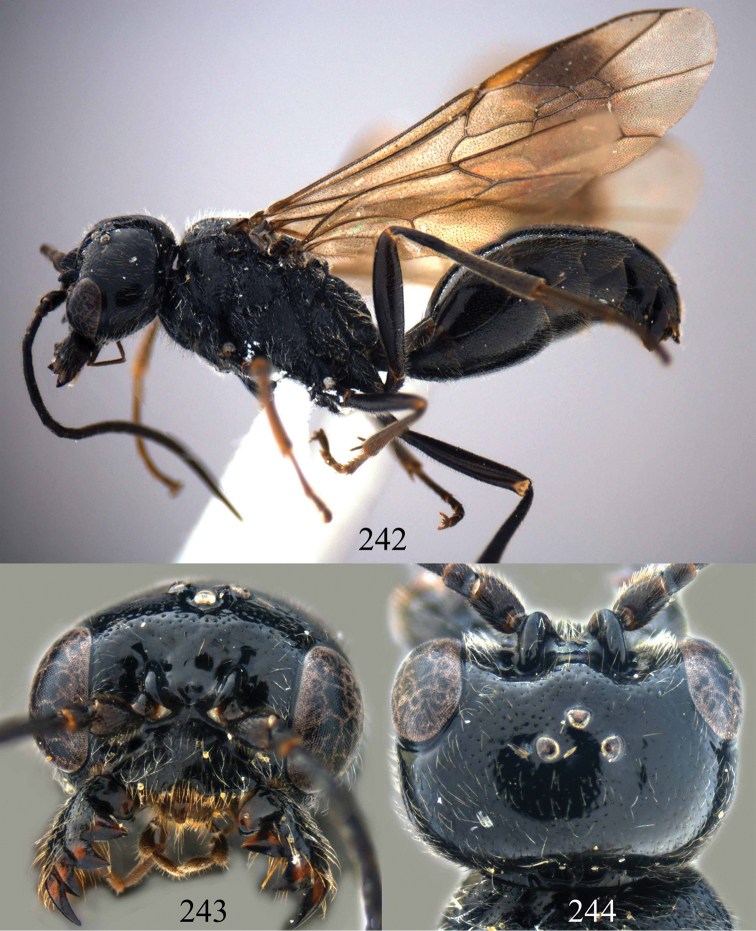
*Pseudogonalos angusta* sp. n., holotype, female. **242** Habitus lateral **243** head anterior **244** head dorsal.

Figs 242 [Fig F50] [Fig F51] [Fig F52] –266

Trigonalis Spinola, 1840: 1 (attributed to Klug and as synonym of the earlier published *Seminota* Spinola, 1840; invalid according to Article 11.6 of the ICZN Code (1999)); Oehlke 1983: 93, 1984: 186 (as valid genus). According to Carmean and Kimsey (1998)*Trigonalis* Spinola is an incorrect spelling of *Trigonalys* Westwood, 1835, but Spinola (1841) indicated that he did not know the latter genus in 1840. The generic name *Trigonalis* was used by Lepeletier (1845) and later authors, and this action could made it available according to Article 11.6.1 of the ICZN Code (1999: “However, if such a name published as a junior synonym had been treated before 1961 as an available name and either adopted as the name of a taxon or treated as a senior homonym, it is made available thereby but dates from its first publication as a synonym”). However, these authors refer to *Trigonalys* Westwood, 1835, what makes *Trigonalis* Lepeletier, 1845, an invalid emendation of *Trigonalys* Westwood and, therefore, both *Trigonalis* Spinola and *Trigonalis* Lepeletier are unavailable names.
Abastus Guérin-Méneville, 1844: 84 (invalid). Type species (by original designation): *Abastus macquartii* Guérin-Méneville, 1844: 84 (nom. nud. and published as synonym of *Trigonalys hahnii*).
Pseudogonalos Schulz, 1906: 209; Weinstein and Austin 1991: 424; Tsuneki 1991: 3; Lelej 1995: 12; Carmean and Kimsey 1998: 72. Type species (by monotypy): *Trigonalis* (!) *hahnii* Spinola, 1840.

#### Diagnosis.

Length of body 5.5–13.9 mm; antenna black and with 23–28 segments; area above supra-antennal elevations distinctly depressed, smooth, with low triangular or transverse protuberance between elevations and inner side of supra-antennal elevations flat, smooth and black (Figs 244, 256); tyloids on 11^th^–14^th^ antennal segments of male short and nearly circular (Fig. 266); occipital carina widened medio-dorsally; apical segment of labial palp widened and obtuse, more or less triangular (Fig. 255); vertex normal, at most with slight median depression dorsally (Fig. 244); mandibles wide in anterior view and sublaterally attached to head (Fig. 243); metanotum strongly convex and finely sculptured medially (Fig. 249); anterior propodeal sulcus finely crenulate laterally (Fig. 249); fore wing with large dark patch below pterostigma; vein 1-SR of fore wing long (Figs 246, 258); hind trochanter black; hind tarsus slightly or not modified; second and third sternites of ♀ flat and moderately sclerotized and no protuberances (Fig. 264); body without pale pattern, at most malar space and margins of basal metasomal sternites and tergites narrowly ivory, remainder black.

#### Biology.

Reared as hyperparasitoid of Ichneumonidae in caterpillars and from Diprionidae (Carmean and Kimsey 1998).

#### Key to species of *Pseudogonalos* Schulz, 1906

**Table:** 

1	Notauli narrow and finely crenulate (Fig. 249); middle mesoscutal lobe convex and shiny, sparsely punctate (Fig. 249); malar space nearly black (Fig. 243); anterior propodeal sulcus without crenulae medially (Fig. 249)	*Pseudogonalos angusta* sp. n.
–	Notauli comparatively wide and coarsely crenulate (Fig. 260); middle mesoscutal lobe less convex and rather matt, largely densely punctate (Fig. 260); malar space ivory (Fig. 255); anterior propodeal sulcus crenulate-rugose medially (Fig. 260)	*Pseudogonalos hahnii* (Spinola, 1840)

### Pseudogonalos angusta sp. n.

http://zoobank.org/ED6936FE-652E-4EC1-B38E-64CF1F448BE2

http://species-id.net/wiki/Pseudogonalos_angusta

Figs 242 –253

#### Type material.

Holotype, ♀ (SCAU) “[China:] Inner Mongolia, Mt. Helan, Halawu–Shatangzi, 3.VIII.2010, sweeping, Rui Guo, SCAU 183”.

#### Diagnosis.

Notauli narrow and finely crenulate; malar space nearly black; middle mesoscutal lobe convex and shiny, sparsely punctate.

#### Description.

Holotype, female, length of body 7.8 mm (of fore wing 6.2 mm).

*Head*. Antenna with 23 segments; frons largely smooth with sparse small punctures (Fig. 243); vertex and temple largely smooth with sparse and fine punctures (Fig. 244); head gradually narrowed behind eyes, eye in dorsal view 0.8 times as long as temple (Fig. 244); occipital carina strongly widened and lamelliform, coarsely crenulate medio-dorsally (Fig. 244); supra-antennal elevations strongly enlarged (about 1.1 times as long as scapus), outer side flat and smooth (Fig. 244); clypeus slightly concave and thick medio-ventrally.

*Mesosoma*. Length of mesosoma 1.4 times its height (Fig. 250); mesopleuron below transverse mesopleural groove rugose anteriorly and rugulose to smooth posteriorly, above groove rugose (Fig. 250); transverse mesopleural groove moderately wide, deep and coarsely crenulate; notauli narrow and finely crenulate; middle lobe of mesoscutum convex and shiny, sparsely punctate anteriorly, rugose at posterior end, lateral lobes sparsely punctate and medially with smooth interspaces (Fig. 249); scutellar sulcus wide, both medially and laterally and coarsely crenulate; scutellum densely reticulate-punctate, rather flat and anteriorly near level of mesoscutum; metanotum medially protruding, obtuse and reticulate-rugose (Fig. 249); anterior propodeal sulcus without crenulae medially (Fig. 249); propodeum obliquely rugulose to rugose medially, smooth and shiny antero-laterally and posteriorly (Fig. 249); posterior propodeal carina thick lamelliform and curved, foramen medially 0.4 times higher than wide basally.

**Figures 245–253. F50:**
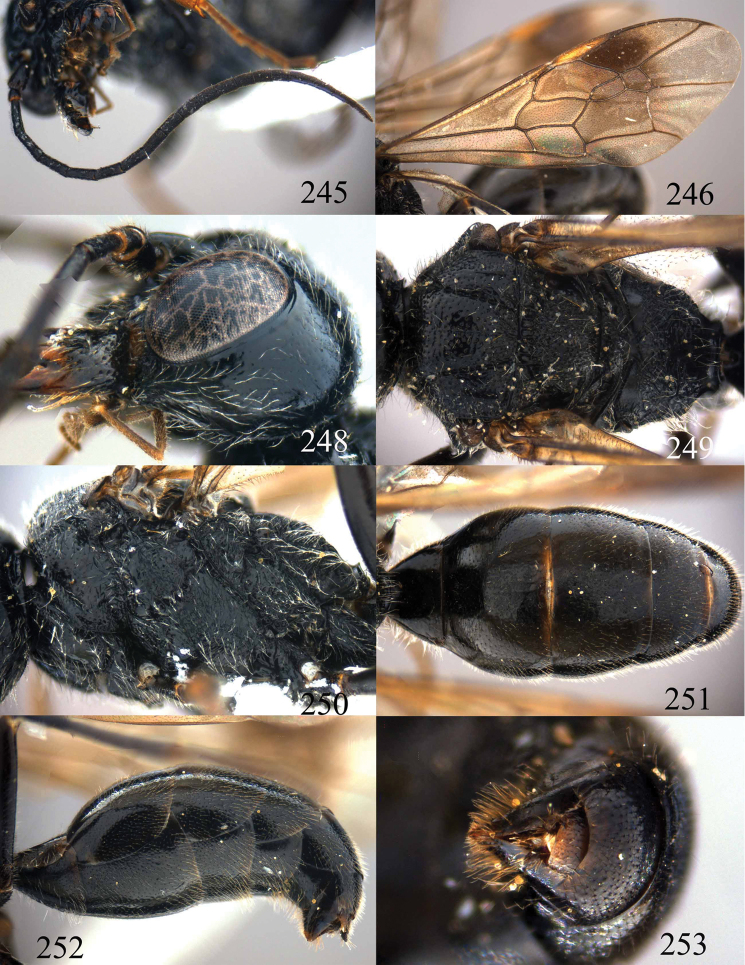
*Pseudogonalos angusta* sp. n., holotype, female. **245** Antenna **246** fore wing **247** head dorsal **248** head lateral **249** mesosoma dorsal **250** mesosoma lateral **251** metasoma dorsal **252** metasoma lateral **253** metasoma posterior dorsal.

*Wings*. Fore wing: length of vein 1-M 1.6 times as long as vein 1-SR (Fig. 246).

*Metasoma*. First tergite 0.5 times as long as apically wide, smooth and with shallow elliptical depression antero-medially (Fig. 251); second–fifth tergites largely smooth except for sparse superficial punctation; sternites largely smooth except for sparse superficial punctation; second sternite slightly convex (Fig. 252); third sternite about 0.4 times as long as second sternite (Fig. 252); hypopygium triangular in ventral view.

*Colour*. Black; malar space nearly black, somewhat brownish; mandibular teeth, palpi, tegulae, fore and mid tibiae and tarsi, pterostigma and anterior half of marginal cell of fore wing dark brown, remainder of wing membrane pale brown.

*Male*. Unknown.

#### Biology.

Unknown. Collected in August.

#### Distribution.

China (Inner Mongolia).

#### Etymology.

From “*angustus*” (Latin for “narrow”) after the narrow notauli.

### Pseudogonalos hahnii

(Spinola, 1840)

http://species-id.net/wiki/Pseudogonalos_hahnii

Figs 254 – 266

Trigonalys hahnii Spinola, 1840: 1; Weinstein and Austin 1991: 425. [type in Dresden (Oehlke) or Turin (Scaramozzino and Pagliano 1989)].
Pseudogonalos hahni (*sic*!); Teranishi 1929: 147; Tsuneki 1991: 14.
Pseudogonalos hahnii ; Marshakov 1981: 104; Lelej 1995: 12; Carmean and Kimsey 1998: 72.
Trigonalys anglicana Shuckard, 1841: 122; Weinstein and Austin 1991: 425.
Abastus macquartii Guérin-Méneville, 1844: 84 (nom. nud.); Weinstein and Austin 1991: 425.
Trigonalys nigra Westwood, 1843: 274; Weinstein and Austin 1991: 425.
Trigonalys aterrima Eversmann, 1849: 384; Weinstein and Austin 1991: 425.
Trigonalys europaea Westwood in Schenck 1861: 167; Weinstein and Austin 1991: 425.
Trigonalys nigra var. *solitaria* Jacobs, 1878: 244; Weinstein and Austin 1991: 425.
Trigonalys hahni var. *phaeognatha* Enderlein, 1905: 200; Weinstein and Austin 1991: 425.
Trigonalys hahni var. *enslini* Torka, 1936: 153; Weinstein and Austin 1991: 425.
Trigonalys prudnicensis Torka, 1936: 151; Weinstein and Austin 1991: 425.

#### Additional material.

1 ♀ (ZJUH) “[China:] Liaoning, Benxi, Laotudingzi Nature Reserve, 15–18.VII.2011, Sheng-nan Song, 201100085”; 1 ♂ (ZJUH) “[China:] Inner Mongolia, Mt. Helang, 30.VII.2010, Hong-fei Cai, 201100273”; 1 ♀ (ZJUH) “[China:] Beijing, Mt. Xi, 28.IV.1986”; 1 ♀ (ZJUH) “[China:] Henan, Jiyuan, 7.VI.2000, Ping Cai, 200102094”.

#### Diagnosis.

Notauli comparatively wide and coarsely crenulate; malar space ivory; middle mesoscutal lobe less convex than of *Pseudogonalos angusta* and rather matt, largely densely punctate.

#### Description.

Redescribed after a female from Liaoning, length of body 10.9 mm (of fore wing 8.2 mm).

*Head*. Antenna with 26 segments; frons densely and finely punctate (Fig. 255); vertex and temple largely smooth with sparse and fine punctures (Figs 256, 259); head gradually narrowed behind eyes, eye in dorsal view 0.7 times as long as temple (Fig. 256); occipital carina strongly widened and lamelliform, coarsely crenulate medio-dorsally (Fig. 256); supra-antennal elevations strongly enlarged (about 1.1 times as long as scapus), outer side flat and smooth (Fig. 256); clypeus slightly concave and thick medio-ventrally.

**Figures 254–256. F51:**
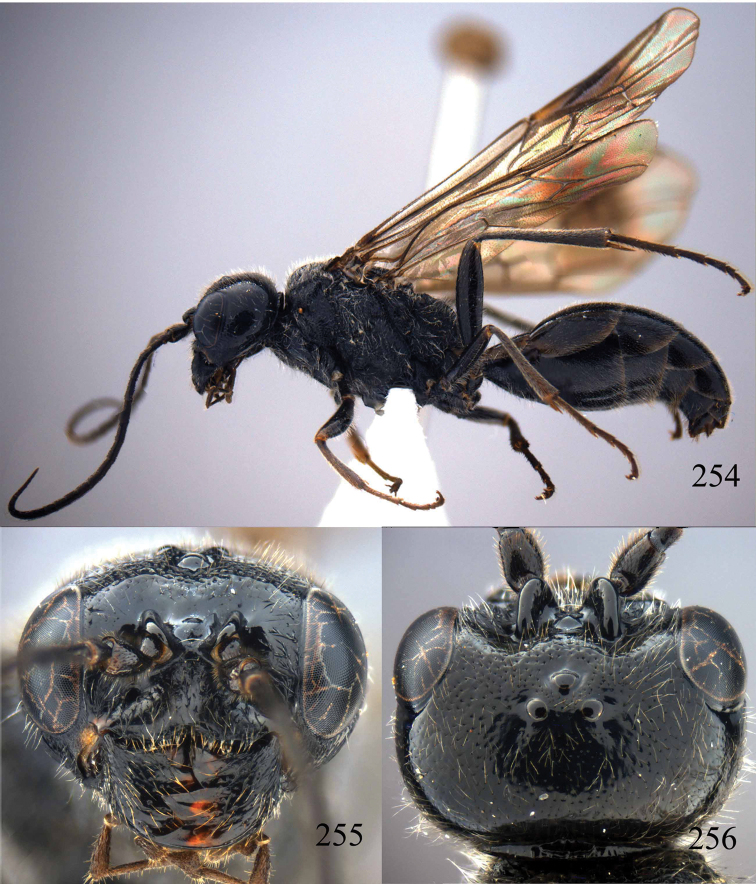
*Pseudogonalos hahnii* (Spinola, 1840), female from Liaoning. **254** Habitus lateral **255** head anterior **256** head dorsal.

**Figures 257–264. F52:**
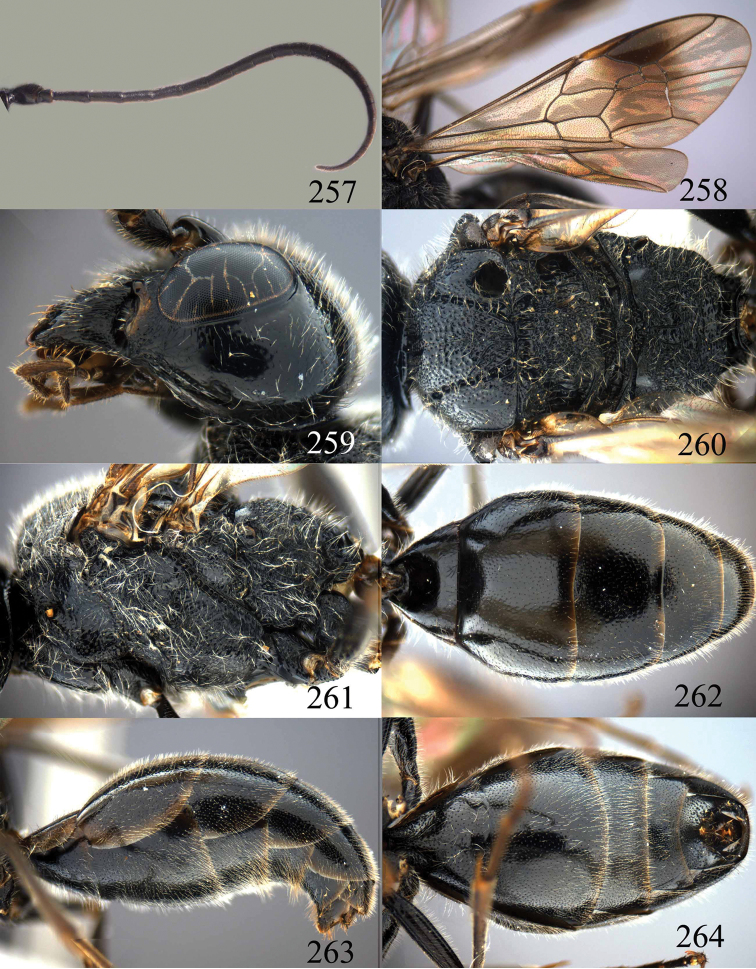
*Pseudogonalos hahnii* (Spinola, 1840), female from Liaoning. **257** Antenna **258** fore and hind wings **259** head lateral **260** mesosoma dorsal **261** mesosoma lateral **262** metasoma dorsal **263** metasoma lateral **264** metasoma ventral.

*Mesosoma*. Length of mesosoma 1.4 times its height (Fig. 261); mesopleuron transversely reticulate-rugose anteriorly, rugulose to smooth posteriorly; transverse mesopleural groove moderately wide, deep and coarsely crenulate; notauli moderately wide, deep and coarsely crenulate; middle lobe of mesoscutum largely densely punctate and rugose at posterior end, lateral lobes mainly finely rugose (Fig. 260); scutellar sulcus wide, both medially and laterally and coarsely crenulate; scutellum densely reticulate-punctate, rather flat and anteriorly near level of mesoscutum; metanotum medially protruding, obtusely bifurcate dorsally and reticulate-rugose (Fig. 260); anterior propodeal sulcus crenulate-rugose and more or less widened medially; propodeum largely coarsely reticulate-rugose except for small smooth area on antero-lateral and posterior margin (Fig. 260); posterior propodeal carina thick lamelliform and curved, foramen medially 0.4 times higher than wide basally.

*Wings*. Fore wing: length of vein 1-M 1.4 times as long as vein 1-SR (Fig. 258).

*Metasoma*. First tergite 0.6 times as long as apically wide, smooth and with distinct elliptical depression antero-medially (Fig. 262); second–fifth tergites largely smooth except for sparse superficial punctures; first and second sternites largely smooth except for sparse superficial punctation, third–fifth sternites densely and superficially punctate (Fig. 264); second sternite slightly convex (Fig. 263); third sternite about 0.3 times as long as second sternite (Fig. 264); hypopygium triangular in ventral view.

*Colour*. Black; malar space ivory; mandibular teeth, palpi, tegulae, fore and mid tibiae and tarsi dark brown; pterostigma and posterior half of first submarginal cell to anterior third of marginal cell of fore wing and area below it dark brown, remainder of wing membrane pale brown.

*Variation*. Length of body 9.4–11.5 mm, of fore wing 7.9–8.9 mm; antenna of ♀ with 25 (1), 26 (2) or 28 (1) segments; propodeum coarsely rugose anteriorly, transversely striate medially and smooth posteriorly; length of vein 1-M of fore wing 1.4–1.9 times as long as vein 1-SR.

*Male*. Length of body 11.3 mm, of fore wing 9.2 mm; antenna with 25 segments, tyloids nearly circular, 0.2 times as long as segment on 11^th^–15^th^ segments (Fig. 266); genitalia extruded; third–seventh tergites densely and superficially punctate.

**Figures 265–266.**
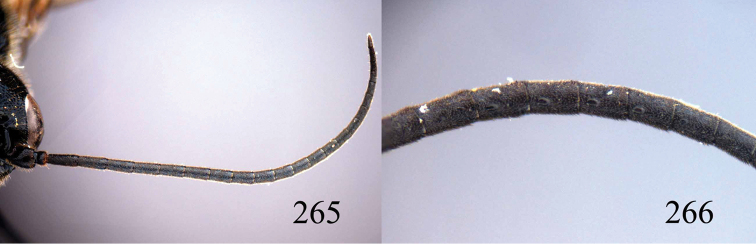
*Pseudogonalos hahnii* (Spinola, 1840), male from Inner Mongolia. **265** Antenna **266** tyloids on 11^th^–15^th^ segments of antenna.

#### Biology.

Hyperparasitoid of Ichneumonidae in caterpillars and reared from Diprionid sawflies (Carmean and Kimsey 1998). Collected in China in April, June and July.

#### Distribution.

China (Liaoning, Inner Mongolia, Beijing, Hebei and Henan); Russia; Ukraine; Kazakhstan; Mongolia; Western Europe (Lelej 2003).

### Taeniogonalos

Schulz, 1906

http://species-id.net/wiki/Taeniogonalos

Figs 267 [Fig F55] [Fig F56] [Fig F57] [Fig F58] [Fig F59] [Fig F60] [Fig F61] [Fig F62] [Fig F63] [Fig F64] [Fig F65] [Fig F66] [Fig F67] [Fig F68] [Fig F69] [Fig F70] [Fig F71] [Fig F72] [Fig F73] [Fig F74] [Fig F75] [Fig F76] [Fig F77] [Fig F78] [Fig F79] [Fig F80] [Fig F81] [Fig F82] [Fig F83] [Fig F84] [Fig F85] [Fig F86] [Fig F87] [Fig F88] [Fig F89] [Fig F90] [Fig F91] [Fig F92] [Fig F93] [Fig F94] [Fig F95] [Fig F96] [Fig F97] [Fig F98] [Fig F99] [Fig F100] [Fig F101] [Fig F102] [Fig F103] [Fig F104] [Fig F105] [Fig F106] [Fig F107] [Fig F108] [Fig F109] [Fig F110] [Fig F111] [Fig F112] –592

Taeniogonalos Schulz, 1906: 212; Weinstein and Austin 1991: 416; Tsuneki 1991: 59; Carmean and Kimsey 1998: 65. Type species (by monotypy): *Trigonalys maculata* Smith, 1851.
Labidogonalos Schulz, 1906: 207; Weinstein and Austin 1991: 414; Carmean and Kimsey 1998: 65. Type species (by monotypy): *Trigonalys ornata* Smith, 1851.
Poecilogonalos Schulz, 1906: 212; Marshakov 1981: 105; Tsuneki 1991: 46; Weinstein and Austin 1991: 422; Tsuneki 1991: 46; Lelej 1995: 14. Type species (by monotypy): *Trigonalys thwaitesii* Westwood, 1874. Synonymized by Carmean and Kimsey 1998.
Nanogonalos Schulz, 1906: 211; Teranishi 1929: 150; Marshakov 1981: 107; Tsuneki 1991: 56; Weinstein and Austin 1991: 421. Synonymized by Carmean and Kimsey 1998. Type species (by monotypy): *Nanogonalos enderleini* De Santis, 1980.
Ischnogonalos Schulz, 1907: 11; 1908: 33; Bischoff 1933: 482, 1938: 11; Weinstein and Austin 1991: 413; Carmean and Kimsey 1998: 65. Type species (by monotypy): *Trigonalys dubia* Magretti, 1997. Syn. n.
Lycogastroides Strand, 1912: 129; Weinstein and Austin 1991: 413. Type species (by original designation): *Lycogastroides gracilicornis* Strand, 1912. Synonymized by Carmean and Kimsey 1998.
Lycogonalos Bischoff, 1913: 155; Weinstein and Austin 1991: 415. Type species (by original designation): *Lycogonalos flavicincta* Bischoff, 1913. Synonymized by Carmean and Kimsey 1998.
Taiwanogonalos Tsuneki, 1991: 35. Type species (by original designation): *Taiwanogonalos alishana* Tsuneki, 1991. Synonymized by Carmean and Kimsey 1998.

#### Diagnosis.

Body length 4.3–13.0 mm; antenna with 21–26 segments, without pale band and slender medially (Fig. 306), of male with linear tyloids (= elevated elongate areas) on 11^th^–16^th^ antennal segments (Fig. 415); supra-antennal elevations smooth or punctate, without depression dorsally, remain far separated from each other medially and without horizontal “shelf” between antennal bases (Figs 269, 319, 330, 363, 374, 408, 419, 430, 454, 465, 487, 509, 520, 551, 562, 573, 584); temple usually punctate or reticulate-punctate and moderately shiny (Fig. 587); occipital carina ending at hypostomal carina at level of mandibular base; vertex flattened, without median depression dorsally (Fig. 584); apical segment of labial palp widened and obtuse, more or less triangular (Fig. 457); mandibles wide in anterior view and sublaterally attached to head (Fig. 453); mesoscutum and scutellum distinctly punctate or rugose (Fig. 458); metanotum at least partly convex latero-dorsally and often sculptured (Fig. 309); vein 1-SR of fore wing medium-sized to long (Figs 272, 489); fore wing often with subapical dark patch (Fig. 410) or large part of fore wing dark brown (Fig. 449); triangular dorso-apical part of hind trochanter separated by an oblique groove; fore trochanter subparallel-sided and distinctly longer than hind trochanter; hind tarsus slightly or not modified; propodeal foramen more or less arched dorsally and often with a lamelliform carina; second sternite convex in lateral view (Fig. 312; but less so in males: Figs 316, 581), strongly sclerotized and frequently densely punctate (Fig. 592), sometimes with a medio-posterior elevation (Fig. 592) but without pair of small teeth; third sternite at most 0.7 times as long as second sternite; basal half of third sternite flat, without a distinct ledge anteriorly (Fig. 592); hypopygium of ♀ pointing anteriorly toward second sternite or straight down or pointing posteriad; body variable, often moderately robust (Figs 317, 328, 452, 485, 507, 582).

#### Biology.

Reared as hyperparasitoid of parasitoid wasps (Ichneumonidae and Braconidae) and parasitoid flies (Tachinidae) in caterpillars, but some species are primary parasitoids of Pergid sawflies in Australia (Raff 1934; Carne 1969; He and Chen 1986; Weinstein and Austin 1995; Carmean and Kimsey 1998).

#### Notes.

The enigmatic genus *Ischnogonalos* Schulz, 1907, is synonymized; it fits well in the genus *Taeniogonalos* as redefined in this paper. Unfortunately, the holotype of the type species could not be found. It is aberrant because of the comparatively slender first metasomal tergite, the posteriorly protruding propodeum, the swollen male antennal segments and the pair of minute teeth medio-posteriorly on the second metasomal sternite.

*Trigonalys lachrymosa* Westwood, 1874, from Mindanao (Philippines) was assigned to the genus *Lycogaster* Shuckard by Bischoff (1938) without giving arguments and the name was changed into *Lycogaster lacrimosa*. The holotype should be in the Semper collection (ZMB?) but could not be traced. Judging from the original description and the figure it does not belong in the genus *Lycogaster*. The comparatively long vein 1-SR of the fore wing, the narrow third submarginal cell of the fore wing and the yellow pattern of the mesosoma (middle lobe of mesoscutum antero-laterally, axilla and metanotum laterally with yellow patches) indicate that it belongs to the genus *Taeniogonalos*, resulting in the new combination *Trigonalys lachrymosa* (Westwood, 1874), comb. n.

#### Key to Chinese species of *Taeniogonalos* Schulz, 1906

**Table:** 

1	Entire fore wing dark brown (Fig. 449); second metasomal sternite only slightly convex (Fig. 453); second tergite and sternite with wide yellow band apically, and remainder of metasoma largely black (Fig. 453); [second and third sternites of ♂ with flattened and shiny posterior patch; occipital carina lamelliform, wide; head with yellow pattern: supra-antennal elevation anteriorly, two patches of clypeus, inner and (narrowly) outer orbita yellow; metanotum with lateral yellow patch]	*Taeniogonalos mongolica* (Popov, 1945)
–	At least posterior half of fore wing subhyaline or faintly yellowish (Fig. 321); second sternite of ♀ distinctly convex (Fig. 277; less of ♂ and in *Taeniogonalos geminata*); colour of metasoma variable (Figs 276, 580), if second tergite and sternite widely yellow apically then also with extensive yellow pattern on other parts of metasoma (Fig. 369); [third sternite of ♂ (and sometimes also second sternite of ♂) without flattened and shiny medio-posterior patch]	2
2	Middle mesoscutal lobe strongly contrasting with both lateral lobes, yellow laterally and orange-brown medially (Fig. 323); anterior half of fore wing largely dark brown (Fig. 321); scutellum largely orange-brown, only medio-posteriorly and narrowly black laterally (Fig. 323); propodeum very finely and regularly densely rugulose (Fig. 323); head without V-shaped pale pattern medio-posteriorly (Fig. 319)	*Taeniogonalos cordata* sp. n.
–	Middle mesoscutal lobe similar to lateral lobes, black or yellow laterally and black medially (Figs 268, 304, 329, 362, 373, 407, 418, 429, 453, 464, 486, 508, 519, 550, 561, 572, 583); anterior half of fore wing and colour of scutellum medially variable, often largely (except subapically) subhyaline (Fig. 410); **if** scutellum largely orange-brown or yellow and anterior half of fore wing largely dark brown (e.g. *Taeniogonalos tricolor*; Fig. 564) then propodeum irregularly rugose (Fig. 566) and head with V-shaped pale pattern medio-posteriorly (Fig. 562)	3
3	Head posteriorly with extensive yellowish or orange-brown pattern, sometimes including a V-shaped yellow or orange pattern behind stemmaticum (Fig. 465); head dorsally often densely reticulate-punctate (Figs 363, 374, 454, 465, 562), with (e.g. *Taeniogonalos gestroi*) or without smooth interspaces and head comparatively long in dorsal view (Fig. 419)	4
–	Head posteriorly entirely black (Fig. 269) or with limited yellowish or orange pattern and black behind stemmaticum (Fig. 374); head dorsally punctate with distinct interspaces and shorter in dorsal view (Figs 269, 305, 330, 374, 408, 430, 487, 509, 520, 551, 573, 584)	9
4	Second metasomal sternite with distinct medio-apical protuberance (Fig. 473), in ♀ resulting in circular or rectangular opening between second and following sternites in lateral view (Fig. 472); third sternite of ♀ with steep ledge medio-apically (Fig. 461; but weaker in small specimens and absent in *Taeniogonalos flavoscutellata*); basal cell and posterior half of first submarginal cells of fore wing subhyaline (but costal cell more or less dark brown), distinctly paler than marginal cell (Fig. 456); [supra-antennal elevations at least partly yellow]	5
–	Second sternite of both sexes without medio-apical protuberance (Fig. 427) and no opening between second and following sternites in lateral view (Fig. 426), at most second sternite of ♀ with minute tubercle (Fig. 381); third sternite of ♀ without apical ledge (Fig. 427); basal cell often largely and posterior half of first submarginal cells of fore wing more or less darkened, similar to colour of marginal cell (Figs 376, 421, 564)	7
5	Protuberance of second metasomal sternite medio-posteriorly concave (Fig. 462); temple dorsally coarsely reticulate-punctate (Fig. 457); scutellum black medio-anteriorly (Fig. 458)	*Taeniogonalos rufofasciata* (Chen, 1949)
–	Protuberance of second sternite triangularly protruding or truncate posteriorly (Figs 371, 473); temple dorsally largely smooth except for some punctures (Fig. 366); scutellum medio-anteriorly yellowish or orange-brown (Fig. 367), at most narrowly darkened	6
6	Protuberance of second metasomal sternite not protruding behind apical margin of sternite (Fig. 371); mesopleuron with extensive yellowish pattern (Fig. 368); second sternite truncate apically in lateral view (Fig. 370)	*Taeniogonalos flavoscutellata* (Chen, 1949), comb. n., re-instated
–	Protuberance of second sternite protruding behind apical margin of sternite (Fig. 473); mesopleuron largely black, without pattern (Fig. 470), at most with orange patch postero-ventrally; second sternite subtruncate apically in lateral view (Fig. 472); [third–fifth tergites with continuous yellow band posteriorly; sixth tergite largely yellow]	*Taeniogonalos sauteri* Bischoff, 1913
7	Mesopleuron and metapleuron with extensive yellow pattern; clypeus narrowly emarginate medio-ventrally (Fig. 418); third submarginal cell of fore wing 0.5–0.7 times as long as second submarginal cell (Fig. 421)	*Taeniogonalos gestroi* (Schulz, 1908), comb. n., re-instated
–	Mesopleuron black or at most with medio-dorsal orange-brown patch and metapleuron black (Fig. 567); clypeus widely emarginate medio-ventrally (Figs 373, 561); third submarginal cell of fore wing 0.7–1.0 times as long as second submarginal cell (Figs 376, 564)	8
8	Basal cell largely and posterior half of first submarginal cells of fore wing more or less darkened, similar to colour of marginal cell (Fig. 564); sixth tergite medially orange-brown or yellow (Fig. 570); most of V-shaped pale area of head dorsally without distinct interspaces between punctures (Fig. 562); second tergite tricoloured: dark brown anteriorly, followed by intermediate chestnut brown area and a medially widened posterior yellow band or bicoloured (Fig. 568); [fourth–sixth tergites without medial dark brown patch; area besides stemmaticum variable, often dark brown]	*Taeniogonalos tricolor* (Chen, 1949)
–	Basal cell and posterior half of first submarginal cells of fore wing subhyaline (but costal cell more or less dark brown), distinctly paler than marginal cell (Fig. 376); sixth tergite with black central patch, surrounded by U-shaped yellow patch (Fig. 382); V-shaped pale area of head dorsally with smooth interspaces between punctures (Fig. 374); second tergite bicoloured (Fig. 380); anteriorly black and posteriorly yellow; [third–fifth metasomal tergites with yellow patches postero-laterally]	*Taeniogonalos formosana* (Bischoff, 1913)
9	Mesosoma mainly or entirely orange-brown dorsally (Fig. 333) or black with extensive yellowish and orange pattern (Fig. 356)	10
–	Mesosoma entirely black dorsally (Figs 274, 434, 524, 555, 588) or with limited yellowish pattern (Figs 309, 378, 412, 513, 578)	11
10	Notauli posteriorly and scutellar sulcus wide and both coarsely crenulate (Fig. 491); occipital carina widened medio-dorsally and crenulate (Fig. 487); head dorso-posteriorly densely punctate and entirely black (♀) (Fig. 487); basal half of antenna (except scapus) yellowish brown (Fig. 488); fore wing without subapical infuscation (Fig. 489)	*Taeniogonalos sculpturata* sp. n.
–	Notauli posteriorly and scutellar sulcus narrow, notauli smooth or finely crenulate and sulcus moderately crenulate (Fig. 333); occipital carina narrow medio-dorsally and smooth (Fig. 330); head dorso-posteriorly comparatively sparsely punctate and black with yellow pattern (♀, sometimes less so in ♂) (Fig. 330); basal half of antenna dark brown or largely so dorsally (Fig. 331); fore wing with subapical dark brown patch or complete marginal cell and surroundings dark brown (Fig. 338); [second sternite of ♂ partly flattened medio-posteriorly, of ♀ distinctly convex; second metasomal tergite more or less with a shallow depression medio-basally; second sternite usually densely punctate, but sometimes sparsely so; head of ♂ darker than of ♀]	*Taeniogonalos fasciata* (Strand, 1913)
11	Third sternite of ♀ with distinct medio-apical protuberance (Figs 517, 559, 592); anterior half of fore wing dark brown (Figs 511, 553, 586); supra-antennal elevations 0.5-0.7 times as long as scapus (Figs 509, 551, 584)	12
–	Third sternite of ♀ without protuberance (Figs 313, 382, 416, 438, 528, 581); anterior half of fore wing only below pterostigma or subapically dark brown (sometimes obsolescent) and remainder subhyaline (Figs 272, 307, 376, 410, 432, 522, 576); supra-antennal elevations 0.1–0.6 times as long as scapus (Figs 269, 305, 374, 408, 430, 520, 573)	14
12	Protuberance of third sternite of ♀ subtruncate medio-apically; metanotum ivory medially (Fig. 513); apex of supra-antennal elevations pale yellowish (Fig. 509); head of ♀ posteriorly with pair of ivory patches in dorsal view (Fig. 509; black in ♂); hind tibia black (Fig. 507); outer side of supra-antennal elevations oblique (Fig. 509)	*Taeniogonalos subtruncata* nom. n.
–	Protuberance of third sternite of ♀ acutely protruding medio-apically (Fig. 591); metanotum, apex of supra-antennal elevations and head posteriorly in dorsal view black (Figs 584, 588); hind tibia brown (Fig. 582); outer side of supra-antennal elevations subvertical (Figs 551, 584)	13
13	Protuberance of third sternite of ♀ in lateral view hook-shaped (Fig. 591); third submarginal cell of fore wing nearly as long as second submarginal cell (Fig. 586)	*Taeniogonalos uncifera* sp. n.
–	Protuberance of third sternite of ♀ in lateral view acute apically (Fig. 558; but of ♂ flat or slightly convex); third submarginal cell of fore wing 0.6–0.7 times as long as second submarginal cell (Fig. 553)	*Taeniogonalos triangulata* sp. n.
14	Scutellum with pair of medium-sized yellow patches medially (Fig. 412) **and** occipital carina strongly widened medio-dorsally and distinctly lamelliform (Fig. 408); mesoscutum yellow latero-posteriorly (Fig. 412)	*Taeniogonalos geminata* sp. n.
–	Scutellum entirely black (Fig. 434) **and/or** occipital carina narrow medio-dorsally and non-lamelliform (Fig. 573); mesoscutum black or orange-brown latero-posteriorly (Fig. 578)	15
15	Occipital carina distinctly lamelliform (also dorso-laterally), distinctly widened medio-dorsally (in ♀ of *Taeniogonalos bucarinata* up to 1.5 times as wide as diameter of ocellus; Fig. 305), in small ♂ often less developed but remaining lamelliform (Fig. 430); mesosoma of ♀ with limited yellowish pattern (Fig. 434) or entirely black (as in ♂); sculpture of mesosoma dorsally comparatively coarsely developed (Fig. 434)	16
–	Occipital carina non-lamelliform, narrow and smooth medio-dorsally (Figs 269, 374, 520, 573); colour pattern of mesosoma variable (Figs 274, 524, 578), if with limited yellowish pattern (*Taeniogonalos formosana*; Fig. 378) then usually sculpture of mesosoma less developed (Fig. 378)	17
16	Occipital carina strongly widened medio-dorsally (in ♀ up to 1.5 times as wide as diameter of ocellus; Fig. 305); anteriorly vertex of ♀ finely punctate and with distinct smooth interspaces (Fig. 305)	*Taeniogonalos bucarinata* sp. n.
–	Occipital carina far less widened medio-dorsally (near weak medio-posterior depression of vertex up to 0.5 times as wide as diameter of ocellus; Fig. 430); anteriorly vertex of ♀ often partly coarsely punctate and smooth interspaces partly narrower than diameter of punctures (Fig. 430)	*Taeniogonalos maga* (Teranishi, 1929)
17	Metasoma dorsally largely orange-brown except first tergite (Fig. 580); second sternite of ♂ distinctly impressed medio-posteriorly and tricoloured (Fig. 581); metanotum of ♂ pale yellow or ivory medially (Fig. 578)	*Taeniogonalos tricolorisoma* sp. n.
–	Metasoma dorsally black with yellowish pattern (Fig. 276) or nearly entirely black; second sternite of ♂ flattened medio-posteriorly and bicoloured or entirely black (Fig. 278); metanotum of ♂ black medially (Fig. 274) or with pair of yellow (often small) patches	18
18	Propodeum comparatively slender (Fig. 274); first metasomal tergite with wide ivory band or patch posteriorly (Fig. 276); vertex largely smooth and strongly shiny (Fig. 269); mesoscutum weakly sculptured (Fig. 274)	*Taeniogonalos alticola* (Tsuneki, 1991), re-instated
–	Propodeum comparatively slender (Fig. 378, 524); first tergite black or with narrow pale band posteriorly (Figs 380, 526); vertex distinctly punctate and moderately shiny (Fig. 374); mesoscutum distinctly sculptured (Fig. 378)	19
19	Outer side of supra-antennal elevations comparatively steep (angle about 45°; Fig. 520); notauli crenulate posteriorly (Fig. 524), rarely indistinctly so; propodeal foramen comparatively narrow and more arched (Fig. 524); area between supra-antennal elevations distinctly concave (Fig. 520); head anteriorly, axilla, scutellum, metanotum and propodeum entirely black (Figs 519, 524)	*Taeniogonalos taihorina* (Bischoff, 1914)
–	Outer side of supra-antennal elevations less steep (angle about 35°; Fig. 374); notauli smooth posteriorly (Fig. 378); propodeal foramen comparatively wide and less arched (Fig. 378); area between elevations comparatively shallowly depressed (Fig. 374); head anteriorly, axilla, scutellum, metanotum and propodeum more or less with a yellowish or brownish pattern (Figs 373, 378)	*Taeniogonalos formosana* (Bischoff, 1913)

### Taeniogonalos alticola

(Tsuneki, 1991) re-instated

http://species-id.net/wiki/Taeniogonalos_alticola

**Figures 267–270.**
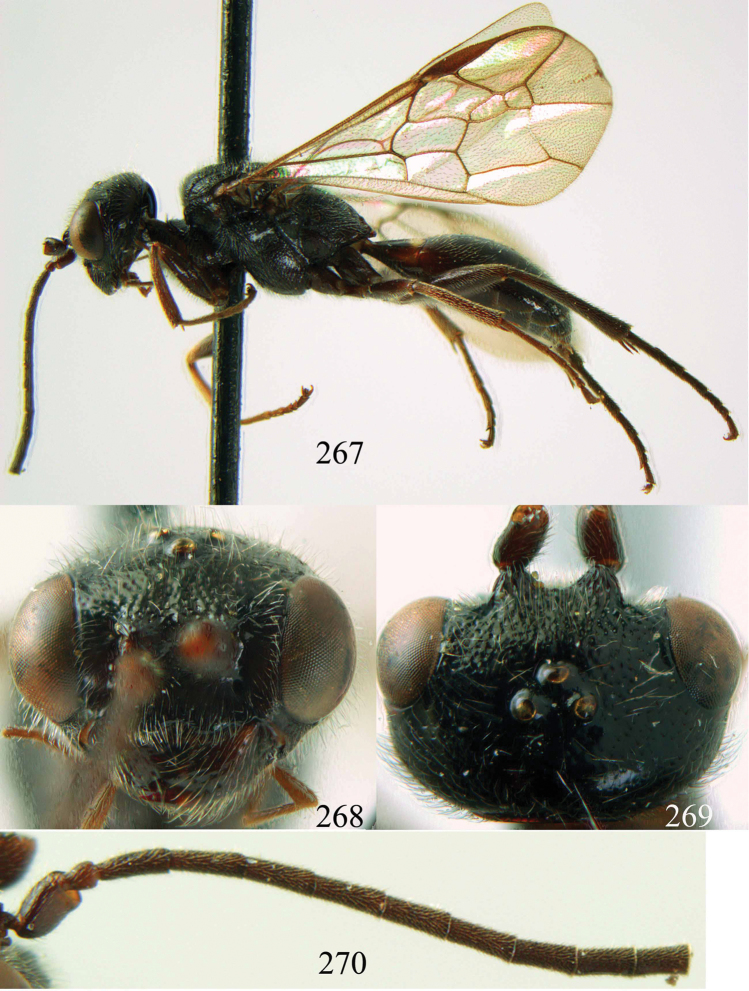
*Taeniogonalos alticola* (Tsuneki, 1991), holotype, male. **267** Habitus lateral **268** head anterior **269** head dorsal **270** antenna.

Figs 267 [Fig F55] [Fig F56] [Fig F57] [Fig F58] –302

Taiwanogonalos alticola Tsuneki, 1991: 42. Synonymized by Carmean and Kimsey 1998 with *Taeniogonalos maga*.
Taiwanogonalos minima Tsuneki, 1991: 43. Synonymized by Carmean and Kimsey 1998 with *Taeniogonalos maga*. Syn. n.
Taiwanogonalos similis Tsuneki, 1991: 45. Synonymized by Carmean and Kimsey 1998 with *Taeniogonalos maga*. Syn. n.

#### Type material.

Holotype of *Taeniogonalos alticola*, ♂ (OMNH), “[China:] Taiwan, 27.V.1929, K. Sato”, “*Taiwanogonalos alticola* Tsuneki, ♂, holotype”. Holotype of *Taeniogonalos minima*, ♂ (OMNH), same label data, but “*Taiwanogonalos minima* Tsuneki, ♂, holotype”. Holotype of *Taeniogonalos similis*, ♂ (OMNH), same label data, but “*Taiwanogonalos similis* Tsuneki, ♂, holotype”.

#### Diagnosis.

Outer side of supra-antennal elevations comparatively steep (angle about 45°) and elevations about 0.5 times as long as scapus and area between elevations distinctly concave (Fig. 269); occipital carina narrow, non-lamelliform and smooth medio-dorsally or nearly so (Fig. 269); head anteriorly and posteriorly, mesosoma dorsally and pronotum laterally entirely black (Figs 268, 269, 274, 275); vertex largely smooth and strongly shiny (Fig. 269); mesoscutum weakly sculptured (Fig. 274); notauli crenulate posteriorly (Fig. 274); scutellum entirely black (Fig. 274); metanotum of male black medially (Fig. 274); fore wing of male subhyaline (Fig. 272; female unknown); propodeum comparatively slender (Fig. 274); propodeal foramen comparatively narrow and posterior propodeal carina distinctly arched (Fig. 274); first metasomal tergite with wide ivory band or pair of patches posteriorly (Fig. 276); second sternite of male slightly convex medio-posteriorly and entirely dark brown (Fig. 278); third sternite of female unknown; metasoma dorsally largely dark brown, only first tergite apically and seventh tergite ivory (Fig. 276).

**Figures 271–278. F55:**
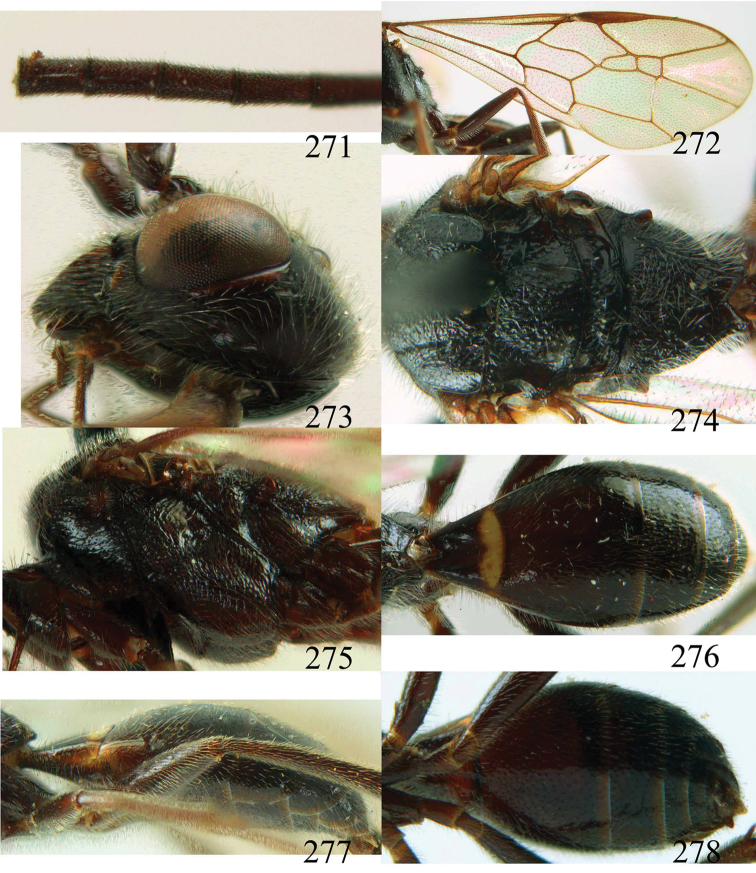
*Taeniogonalos alticola* (Tsuneki, 1991), holotype, male. **271** Tyloids on 10^th^–16^th^ segments of antenna **272** fore wing **273** head lateral **274** mesosoma dorsal **275** mesosoma lateral **276** metasoma dorsal **277** metasoma lateral **278** metasoma ventral.

**Figures 279–282. F56:**
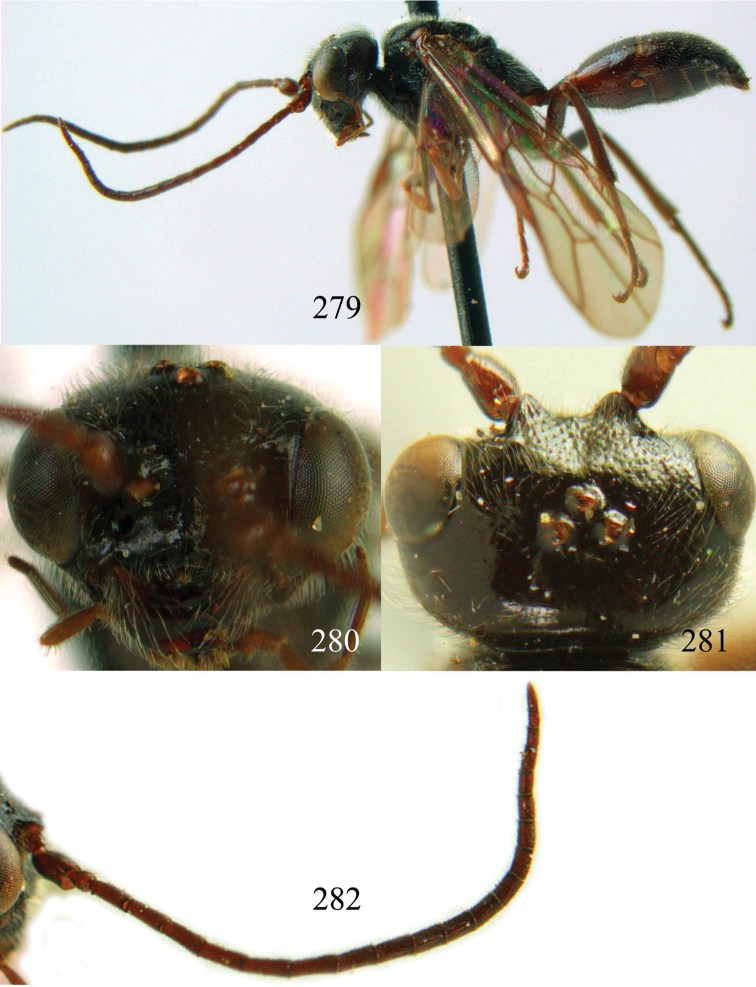
*Taeniogonalos minima* (Tsuneki, 1991), holotype, male. **279** Habitus lateral **280** head anterior **281** head dorsal **282** antenna.

**Figures 283–290. F57:**
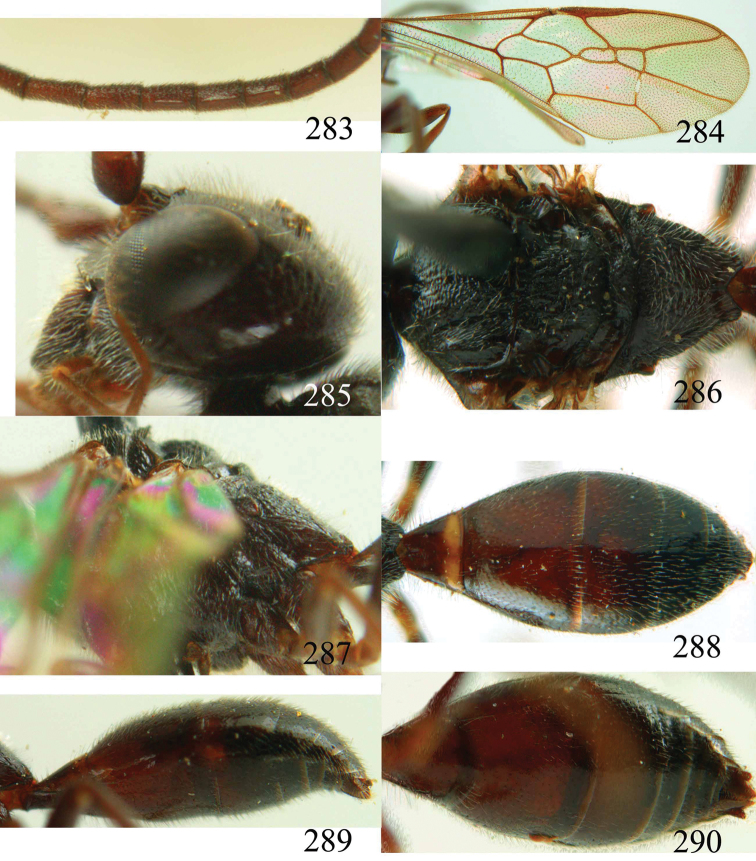
*Taeniogonalos minima* (Tsuneki, 1991), holotype, male. **283** Tyloids on 10^th^–16^th^ segments of antenna **284** fore wing **285** head lateral **286** mesosoma dorsal **287** mesosoma lateral **288** metasoma dorsal **289** metasoma lateral **290** metasoma ventral.

**Figures 291–293. F58:**
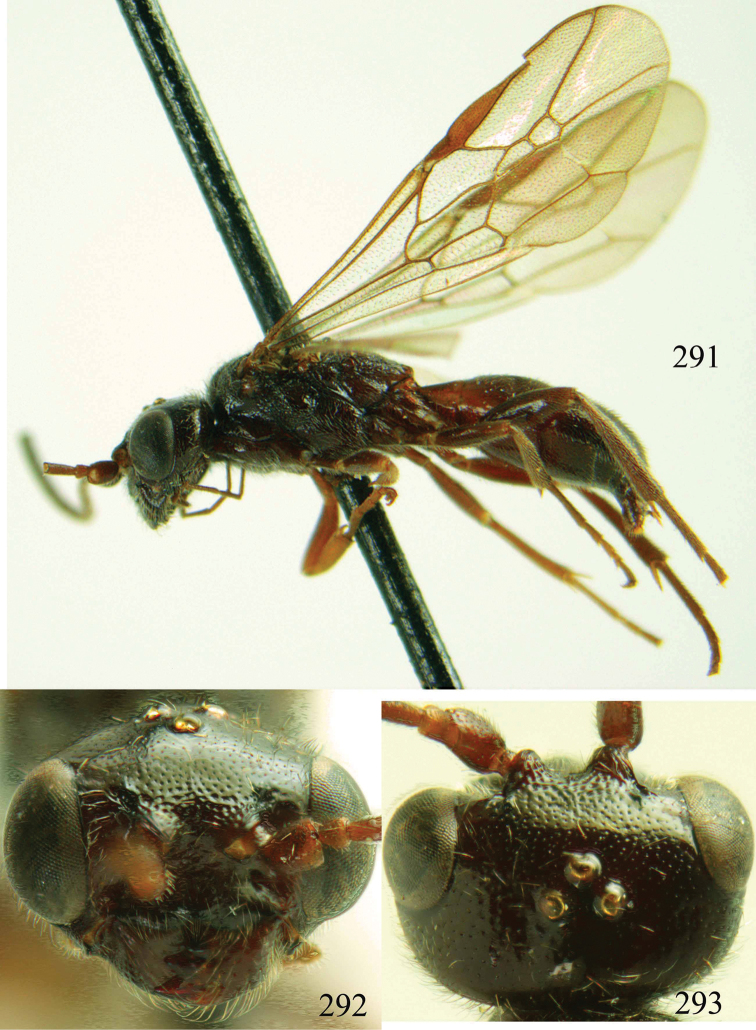
*Taeniogonalos similis* (Tsuneki, 1991), holotype, male. **291** Habitus lateral **292** head anterior **293** head dorsal.

**Figures 294–302. F59:**
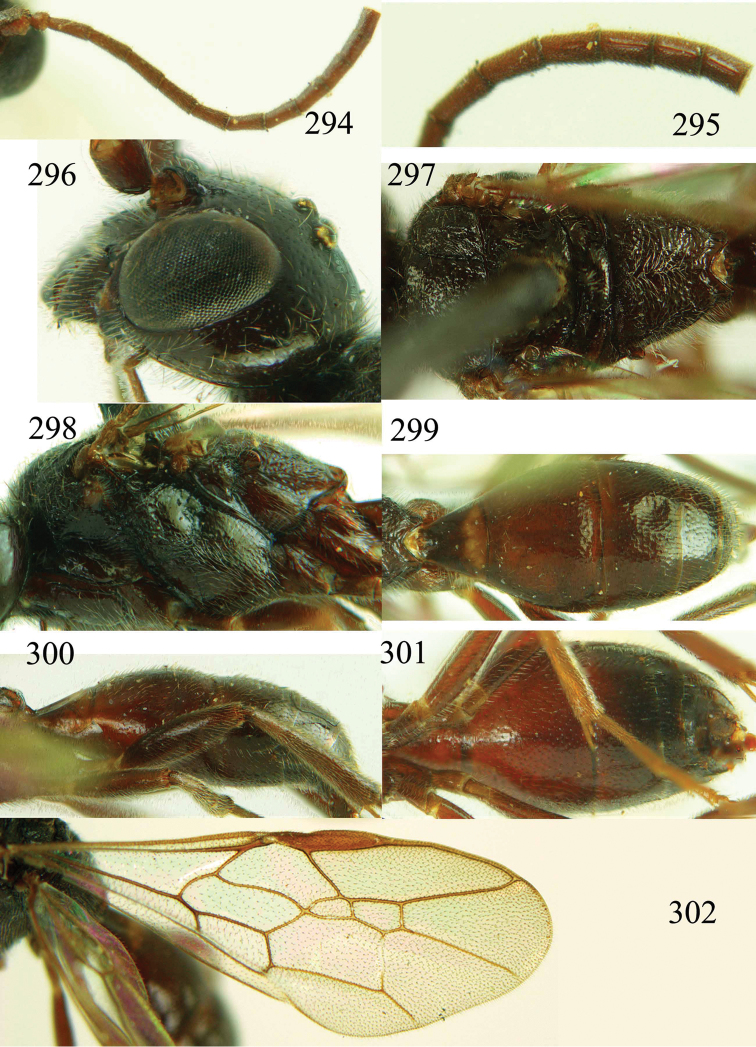
*Taeniogonalos similis* (Tsuneki, 1991), holotype, male. **294** Antenna **295** tyloids on 10^th^–16^th^ segments of antenna **296** head lateral **297** mesosoma dorsal **298** mesosoma lateral **299** metasoma dorsal **300** metasoma lateral **301** metasoma ventral **302** fore wing.

#### Description.

Holotype of *Taeniogonalos alticola*, male, length of body 6.0 mm (of fore wing 5.4 mm).

*Head*. Antenna incomplete, with 11 segments remaining; frons moderately punctate with smooth interspaces much wider than width of punctures; vertex sparsely punctulate and strongly shiny (Fig. 269), with rather long setae; temple largely smooth with few punctures at orbita (Fig. 273); head gradually narrowed behind eyes, eye in dorsal view nearly as long as temple (Fig. 269); occipital carina narrow, non-lamelliform, smooth (Fig. 269); supra-antennal elevations medium-sized (about 0.5 times as long as scapus), outer side distinctly oblique (angle about 45°) carinate and with few punctures and rugae (Fig. 269); area between elevations distinctly concave (Fig. 269); clypeus distinctly concave and thick medio-ventrally.

*Mesosoma*. Length of mesosoma 1.5 times its height (Fig. 275); mesopleuron densely and finely obliquely rugulose, but smooth posteriorly, rather shiny; transverse mesopleural groove narrow, shallow and anteriorly crenulate; notauli narrow, rather deep and finely crenulate posteriorly; middle lobe of mesoscutum irregularly transversely rugose, lateral lobes rugulose laterally, coriaceous medially and with satin sheen (Fig. 274); scutellar sulcus complete, narrow and narrowly crenulate; scutellum rather flat and moderately rugose but smooth and shiny posteriorly, anteriorly near level of mesoscutum; metanotum medially distinctly convex and finely rugose (Fig. 274); propodeum comparatively slender and flat, anteriorly irregularly finely rugose and posteriorly largely smooth (Fig. 274); posterior propodeal carina distinctly arched, narrow lamelliform, foramen comparatively narrow (Fig. 274) and as high as wide basally.

*Wings*. Fore wing: vein 1-M 1.4 times as long as vein 1-SR (Fig. 272), distinctly curved and vein 1-SR narrow anteriorly; second submarginal cell 1.5 times as long as third cell.

*Metasoma*. First tergite 0.8 times as long as apically wide, smooth and with shallow wide depression medially (Fig. 276); second and following tergites superficially punctate and strongly shiny (Fig. 276); second sternite slightly convex, superficially and rather sparsely punctate (Fig. 278); third sternite without depression.

*Colour*. Black or dark brown; outer orbita slightly brownish (Fig. 268); mandibles largely dark brown; palpi, pronotum postero-dorsally and tegulae brown; first tergite widely posteriorly and seventh tergite ivory (Fig. 276); antenna mainly, legs and pterostigma dark brown; fore wing membrane subhyaline (Fig. 272).

*Female*. Unknown.

*Variation*. Holotypes of *Taeniogonalos minima* and of *Taeniogonalos similis* are very similar to the holotype of *Taeniogonalos alticola*. *Taeniogonalos minima* has the antenna with 22 segments and longitudinal tyloids on 10^th^-13^th^ segments (Fig. 283); mesoscutal lobes largely smooth and shiny or rugose; mesopleuron smooth (except antero-dorsally) to obliquely finely rugulose; seventh tergite brown or ivory; length of body 4.8-6.0 mm and of fore wing 4.4–5.4 mm.

#### Biology.

Unknown. Collected in May.

#### Distribution.

China (Taiwan).

### Taeniogonalos bucarinata sp. n.

http://zoobank.org/10456B2F-047E-47B8-B3D8-B0D6B67B0B5D

http://species-id.net/wiki/Taeniogonalos_bucarinata

Figs 303 – 316

#### Type material.

Holotype, ♀ (ZJUH) “[China:] Gansu, Baishui River, Qiujiaba, 2348 m, 26.VII.2004, Qiong Wu, 20046942”. Paratypes: 1 ♀ (ZJUH) “[China:] Gansu, Wen County, Mt. Lilou, 1809 m, 29.VII.2004, Xue-xin Chen, 20046943”; 3 ♀ + 6 ♂ (SCAU, RMNH) “[China:] Ningxia, Mt. Liupan, Qiuqianjia, 8.VII.2008, Jie-Min Yao, 200808549, 200808554, 200808576; id., but Jing-xian Liu, 200801030, 2008013039, 200800991, 200800999, 200800997, 200801038”; 1 ♂ (SCAU) “[China:] Ningxia, Mt. Liupan, Heshangpu, 25.VII.2008, Jing-xian Liu, 200800498”; 6 ♂ (SCAU) “[China:] Ningxia, Mt. Liupan, Hongxia Forestry Farm, 1.VII.2008, Jing-xian Liu, 200800761, 200800760, 200800782, 200800780, 200800786; id., but 1–7.VII.2008, Jie-min Yao, 200808818”; id, but 25.VII.2008, Jing-xian Liu, 200800498”; 1 ♂ (ZJUH) “[China:] Ningxia, Mt. Liupan, Fengtai Forestry Farm, 27.VI.2008, Gang Yao, 200800538”; 2 ♀ (ZJUH) “[China:] Shaanxi, Hu County, Zhuque National Forest Park, 11.VII.2012, Sheng-nan Song, 201200372, 201200382”; 1 ♀ (ZJUH) “[China:] Shaanxi, Liping National Forest Park, 1742 m, 22.VII.2004, Qiong Wu, 20046852”; 1 ♂ (ZJUH) “[China:] Shaanxi, Mt. Taibai, Dianbingchang, 1200 m, 1.VII.1981, SCAU 101”; 1 ♂ (ZJUH) “[China:] Shaanxi, Mt. Taibai, Haopingsi, 1200 m, 1.VII.1981, Lin Xue, SCAU 088”; 1 ♀ (ZJUH) “[China:] Shaanxi, Qingling, Mt. Tiantai, 4.IX.1999, 991703”; 3 ♀ + 1 ♂ (ZJUH) “[China:] Henan, Song County, Mt. Baiyun, 17.VIII.2008, Man-man Wang, 200801258, 200801244; id., but 1400 m, 22.VII.2003, Gui-shou Xue, 20047318; id., but 19.VII.1996, Ping Cai, 973013”; 1 ♀ + 1 ♂ (ZJUH) “[China:] Henan, Neixiang, Mt. Baotianman, 1700 m, 15.VII.1998, Xue-xin Chen, 987964, 989186”; 2 ♀ + 4 ♂ (ZJUH) “[China:] Zhejiang, Mt. Tianmu, 29.VII.1984, Ying Qian, 842985; id., 1.VII.1982, Rui-liang Wang, 825915; id., 18.VI.1983, Jun-hua He, 831555; id., 18.VI.1983, Yun Ma, 831377, 830999; id., VIII.1981, Rong-lan Yuan, 816421”;1 ♀ + 9 ♂ (ZJUH, RMNH) “[China:] Zhejiang, Mt. Tianmu, Xianrending, 1509 m, 14.VII.1998, MT, Ming-shui Zhao, 200010839; id., but 20.VII.1998, 992751, 992755; id., but 23.VII.1998, 200010698; id., but Xue-xin Chen, 20034499; id., but 2–4.VI.1990, Zu-hua Shi, 901555, 902342; id., but Yong-gen Lou, 900390; id., but Xin-geng Wang, 903291; id., but 29.VII.2003, Min Shi 20034552”; 1 ♀ (ZJUH) “[China:] Zhejiang, West Mt. Tianmu, Laodian–Xianrending, 1250–1506 m, 5.VI.1989, Yan-ming Chen, 892251”; 1 ♀ (SCAU) “[China:] Zhejiang, Anji, Mt. Longwang, 31.VIII.1993, Zai-fu Xu, 9310266”; 1 ♂ (ZJUH) “[China:] Fujian, Mt. Wuyi, 14.VII.1994, Xue-xin Chen, 942655”; 1 ♀ (ZJUH) “[China:] Fujian, Mt. Huanggang, 13.VII.1985, Dong-hong Huang, SCAU 089”; 1 ♀ (ZJUH) “[China:] Sichuan, Wanglang National Nature Reserve, 26.VII.2006, Hong-ying Zhang, 200611201”; 1 ♀ (ZJUH) “[China:] Sichuan, Pingwu, Baimazhai, 25.VII.2006, Hong-ying Zhang, 200611107”; 1 ♀ (ZJUH) “[China:] Sichuan, Mt. Emei, 7.VII.2009, Jiang-li Tan, 200906431”; 1 ♀ (ZJUH) “[China:] Sichuan, Wolong National Nature Reserve, 21.VII.2006, Zhi-lei Gao, 200610833”; 2 ♂ (ZJUH) “[China:] Yunnan, Dali, Yunlong, 3.VI.2009, Jiang-li Tan, 200906610, 200906613”.

#### Diagnosis.

Outer side of supra-antennal elevations oblique and elevations 0.1–0.4 times as long as scapus (Fig. 305); occipital carina strongly widened medio-dorsally (in female about 1.5 times as wide as diameter of ocellus), distinctly lamelliform (also dorso-laterally) and coarsely crenulate (Fig. 305), in small males often less developed but remaining lamelliform; head posteriorly entirely black (Fig. 305) or with limited yellowish or orange pattern and black behind stemmaticum, dorsally punctate with distinct interspaces (Fig. 305); mesosoma of female with limited yellowish pattern (Fig. 309) or absent in melanistic specimens (as in male); sculpture of mesosoma dorsally comparatively coarsely developed (Fig. 309); middle mesoscutal lobe similar to lateral lobes, black or yellow laterally and black medially (Fig. 309); scutellum entirely black (Fig. 309); fore wing largely subhyaline with subapical dark brown patch (Fig. 307); second sternite of female distinctly convex (Fig. 312); of male with shallow depressed subposterior area (Fig. 316); third sternite of male slightly convex or flat subposteriorly.

**Figures 303–305. F60:**
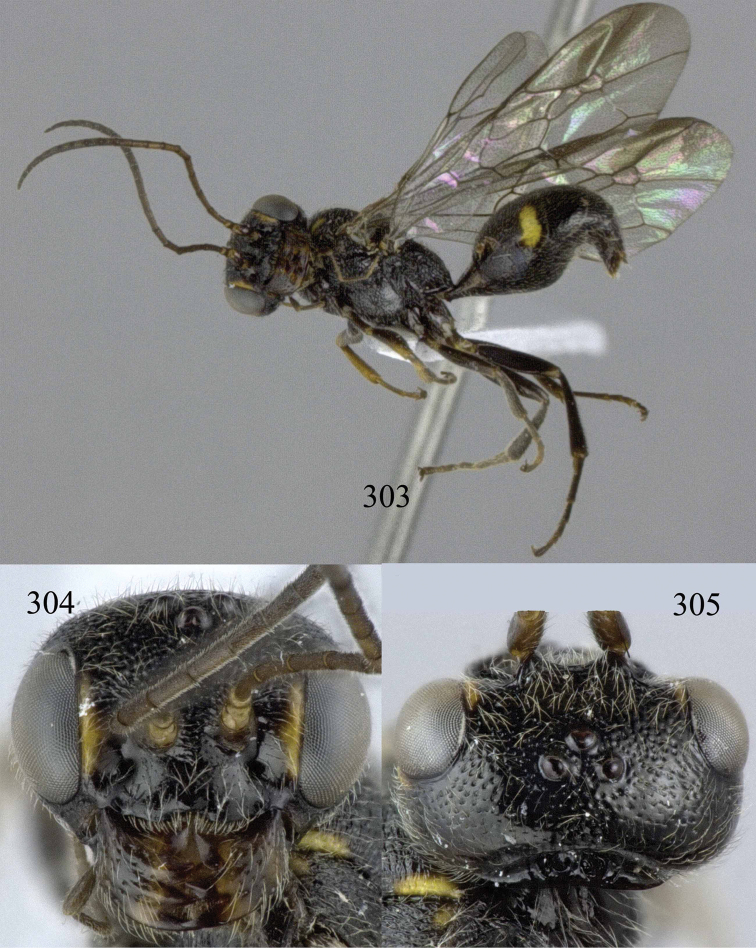
*Taeniogonalos bucarinata* sp. n., holotype, female. **303** Habitus lateral **304** head anterior **305** head dorsal.

**Figures 306–313. F61:**
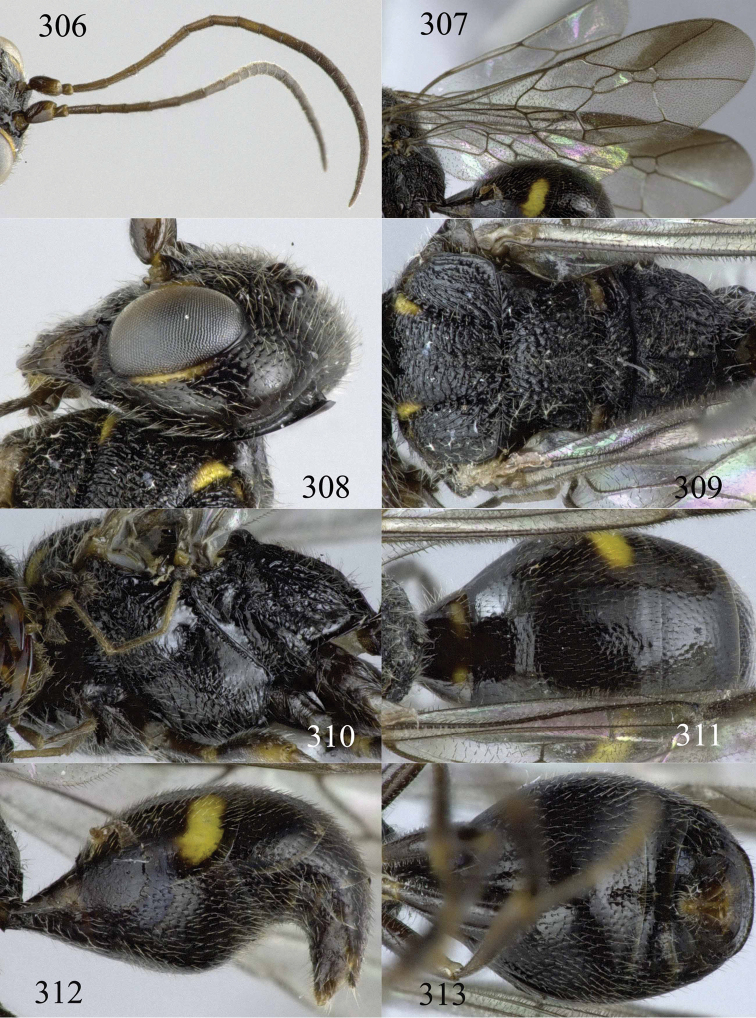
*Taeniogonalos bucarinata* sp. n., holotype, female. **306** Antennae **307** fore and hind wings **308** head lateral **309** mesosoma dorsal **310** mesosoma lateral **311** metasoma dorsal **312** metasoma lateral **313** metasoma ventral.

**Figures 314–316. F62:**
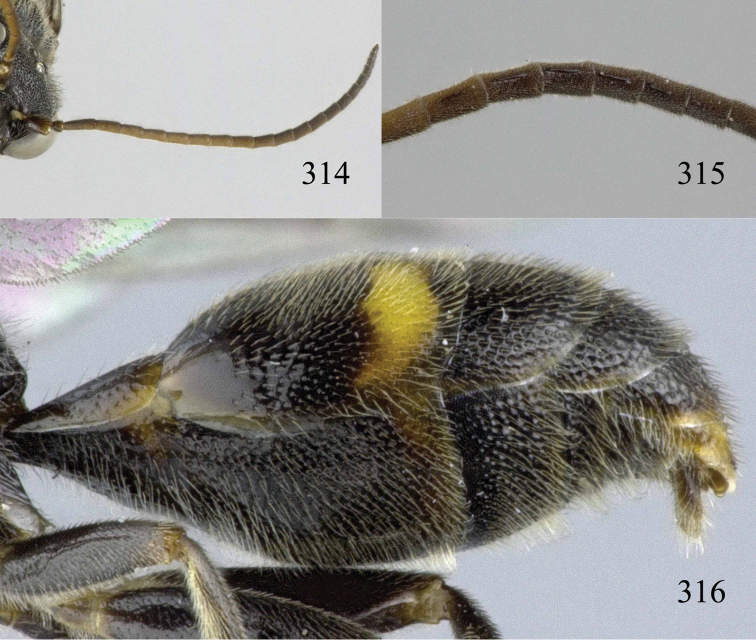
*Taeniogonalos bucarinata* sp. n., paratype, male from Ningxia. **314** Antenna **315** tyloids on 11^th^–16^th^ segments of antenna **316** metasoma lateral.

#### Description.

Holotype, female, length of body 5.9 mm (of fore wing 5.2 mm).

*Head*. Antenna with 22 segments; frons spaced punctate (interspaces about equal to width of punctures or less); vertex largely smooth except some superficial punctures (Fig. 305), with medium-sized setae; temple largely smooth except some sparse punctation (Fig. 308); head gradually narrowed behind eyes, eye in dorsal view 1.5 times as long as temple (Fig. 305); occipital carina strongly widened (medio-dorsally about 1.5 times as wide as posterior ocellus) and lamelliform medio-dorsally, with one strong median carina and few weak carinae laterally; supra-antennal elevations medium-sized (about 0.4 times as long as scapus) and their outer side oblique (Fig. 305); clypeus moderately concave and thick medio-ventrally.

*Mesosoma*. Length of mesosoma 1.4 times its height (Fig. 310); transverse mesopleural groove moderately wide, deep and sparsely crenulate; mesopleuron antero-dorsally irregularly rugose, posteriorly smooth and shiny and antero-ventrally finely irregularly rugose; notauli moderately narrow, deep and largely crenulate; middle lobe of mesoscutum coarsely transversely rugose, lateral lobes mainly finely rugose (Fig. 309); scutellar sulcus complete, medium-sized and crenulate medially and wider laterally; scutellum densely reticulate-rugose, convex anteriorly and above level of mesoscutum, but flattened medially; metanotum medially slightly convex, not protruding and largely obliquely rugose (Fig. 309); propodeum largely longitudinally rugose or nearly so (Fig. 309); posterior propodeal carina rather wide lamelliform and distinctly arched (foramen about 1.1 times as wide as high medially).

*Wings*. Fore wing: length of vein 1-M 1.4 times as long as vein 1-SR (Fig. 307).

*Metasoma*. First tergite 0.6 times as long as apically wide, smooth and with distinct elliptical depression medially (Fig. 311); second tergite largely smooth and shiny, other tergites superficially punctate (Fig. 311); sternites largely smooth except spaced superficial punctures; second sternite distinctly curved in lateral view (Fig. 312); third sternite about 0.2 times as long as second sternite (Fig. 313); hypopygium triangular in ventral view.

*Colour*. Black; apex of scapus, inner and outer orbita narrowly, middle mesoscutal lobe antero-laterally, small anterior spot of lateral lobe, metanotum with small lateral spot, first tergite with faint patch postero-laterally and second tergite with large postero-lateral patch yellow (Fig. 311); apical 0.4 of antenna, tegulae, remainder of scapus and basally palpi dark brown, remainder of antenna and palpi apically brown; mandible dark brown but subbasally and near base of teeth brown; apex of trochanters narrowly and trochantelli largely pale yellow and remainder dark brown; fore femur apically and most of tibia yellowish brown, remainder of fore leg dark brown; apex of metasoma narrowly brown; apical patch of fore wing, pterostigma and veins dark brown, remainder of wing membrane subhyaline.

*Variation*. Length of body 4.9–10.4 mm, of fore wing 4.7–9.0 mm; antenna of ♀ with 21 (1), 22 (10), 23 (6) or 24 (2) segments; yellow patches of inner orbita wide triangular or linear, mandible brown to largely yellow; lateral brownish patch of vertex present or absent; patch lateral mesoscutal lobes postero-laterally and axilla black or yellow; metanotum black or yellow medially and its lateral patch narrow or wide; propodeum black or with pair of large yellow patches; tegulae dark brown, brown or yellow; first and second sternites latero-posteriorly black or orange-brown; only first and second tergites or all tergites yellow or orange-brown latero-posteriorly, patches sometimes connected except for a small brown triangle; second tergite largely smooth to largely moderately densely punctate; vertex largely smooth except some superficial punctures to largely coarsely punctate; length of vein 1-M of fore wing 1.3–1.6 times as long as vein 1-SR; width of depression in front of occipital carina medio-dorsally 1.0–1.5 times width of posterior ocellus and crenulation varies from strong to weak.

*Male*. Length of body 5.0–8.4 mm, of fore wing 4.2–7.7 mm; antenna with 21 (1), 22 (16), 23 (10) or 24 (3) segments, tyloids linear, 0.7 times as long as segment on 11^th^–14^th^ segments and about half as long on 10^th^ and 15^th^ segments with a remnant on 16^th^ segment; genitalia internal, sometimes extruded (Fig. 316); in general males are darker than females: head entirely black or orbita narrowly yellow and mesosoma black or pronotum dorsally slightly yellow; tegulae brown or yellow; metasoma coloured as holotype or darker; sculpture varies as in females, but in general less strongly developed.

#### Biology.

Unknown. Collected in June–September at 1200–2350 m.

#### Distribution.

China (Gansu, Ningxia, Shaanxi, Henan, Zhejiang, Fujian, Sichuan, Yunnan).

#### Etymology.

Named after the strongly enlarged occipital carina: from “*bu*” (Latin for “large”) and “*carina*” (Latin for “keel”).

### Taeniogonalos cordata sp. n.

http://zoobank.org/4998D0C4-BED6-4FB0-8241-2E780901AE84

http://species-id.net/wiki/Taeniogonalos_cordata

Figs 317 –327

#### Type material.

Holotype, ♀ (ZJUH) “[China:] Yunnan, Dali, Yunlong, 3.VI.2009, Jiang-li Tan, 200906614”.

#### Diagnosis.

Outer side of supra-antennal elevations oblique and elevations about 0.2 times as long as scapus (Fig. 319); and anterior half dark brown (Fig. 319); head black dorsally, without V-shaped pale pattern medio-posteriorly (Fig. 319); middle mesoscutal lobe strongly contrasting with both black lateral lobes, yellow laterally and orange-brown medially (Fig. 323); scutellum largely orange-brown, only medio-posteriorly and laterally narrowly black (Fig. 323); anterior half of fore wing largely dark brown and posterior half of fore wing subhyaline (Fig. 321); propodeum very finely and regularly densely rugulose (Fig. 323); second tergite and sternite narrowly yellow apically (Fig. 325); second sternite of ♀ distinctly convex (Fig. 326).

**Figures 317–319. F63:**
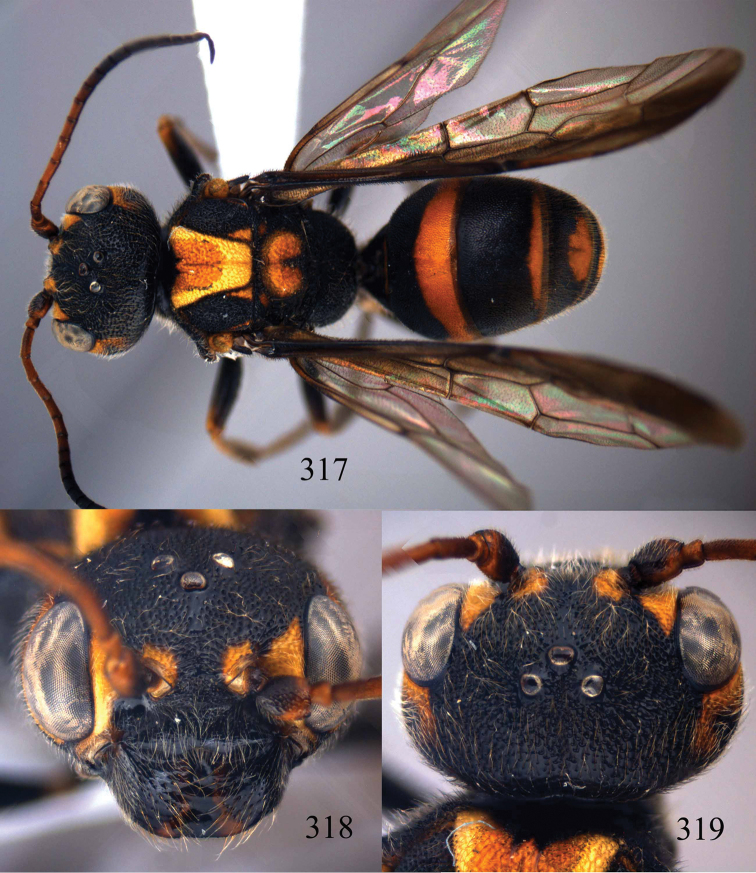
*Taeniogonalos cordata* sp. n., holotype, female. **317** Habitus dorsal **318** head anterior **319** head dorsal.

**Figures 320–327. F64:**
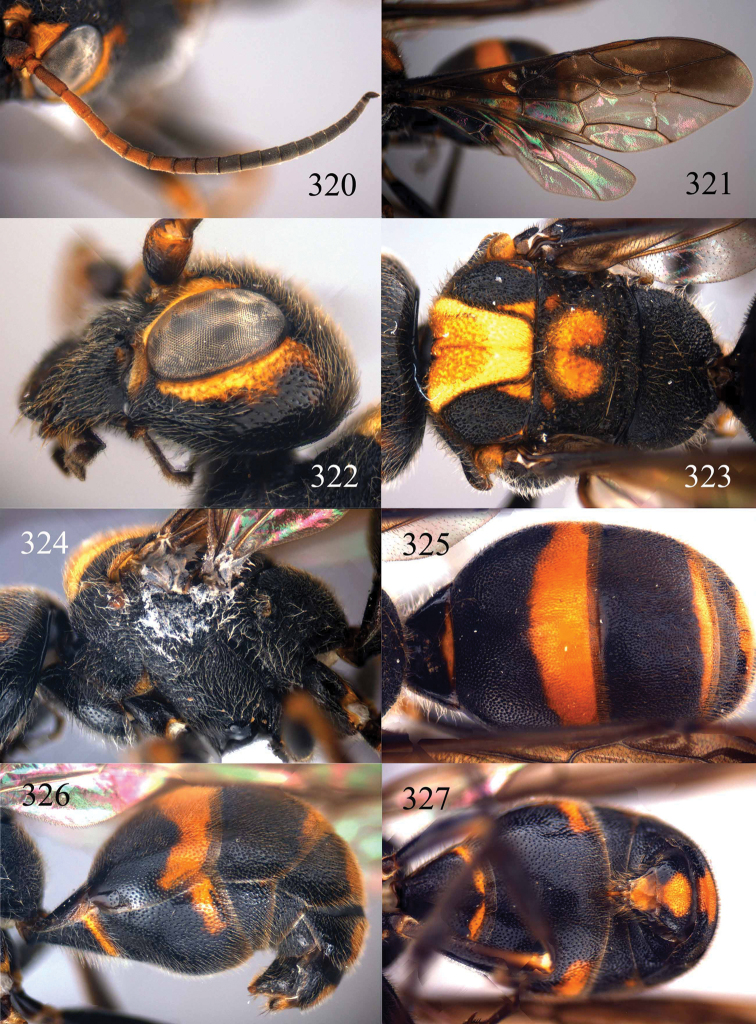
*Taeniogonalos cordata* sp. n., holotype, female. **320** Antenna **321** fore and hind wings **322** head lateral **323** mesosoma dorsal **324** mesosoma lateral **325** metasoma dorsal **326** metasoma lateral **327** metasoma ventral.

#### Description.

Holotype, female, length of body 8.3 mm (of fore wing 7.6 mm).

*Head*. Antenna with 22 segments; frons spaced punctate and vertex densely punctate with no smooth interspaces (Fig. 319), with rather long setae; temple rugulose-punctate (Fig. 322); head gradually narrowed behind eyes, eye in dorsal view 1.3 times as long as temple (Fig. 319); occipital carina narrow medio-dorsally; supra-antennal elevations medium-sized (about 0.2 times as long as scapus), outer side oblique and largely smooth except for sparse punctures; clypeus slightly concave and thick medio-ventrally.

*Mesosoma*. Length of mesosoma 1.4 times its height (Fig. 324); mesopleuron obliquely rugose; notauli narrow, deep and finely crenulate; middle lobe of mesoscutum coarsely irregularly rugose, lateral lobes longitudinally rugose (Fig. 323); scutellar sulcus complete, moderately wide and crenulate; scutellum densely and coarsely reticulate-rugose, convex anteriorly and near level of mesoscutum, but flattened medially; metanotum medially slightly convex, not protruding and densely reticulate-rugose (Fig. 323); propodeum coarsely reticulate-rugose (Fig. 323); posterior propodeal carina thick lamelliform.

*Wings*. Fore wing: length of vein 1-M 1.3 times as long as vein 1-SR (Fig. 321).

*Metasoma*. First tergite 0.4 times as long as apically wide, smooth and with shallow elliptical depression medially (Fig. 325); other tergites and all sternites densely and finely punctate (Figs 325, 327); second sternite distinctly convex medially, rather flattened anteriorly and posteriorly; third sternite about 0.1 times as long as second sternite (Fig. 327).

*Colour*. Black; head anteriorly with moderate orange-brown pattern (Fig. 318); remainder of head black except for yellowish brown outer orbita (Fig. 322); mesosoma laterally black except for ventral and dorsal brown small patches of pronotal side and middle mesoscutal lobe strongly contrasting with both lateral lobes, yellow laterally and orange-brown medially (Fig. 323); scutellum largely orange-brown, only medio-posteriorly and narrowly black laterally (Fig. 323); axilla with small brown patch; metasoma dorsally black, first three tergites with posterior orange-brown stripes, other tergites with large orange-brown patch medially; metasoma ventrally black (Fig. 323), first sternite with orange posterior brown stripes black basally, second and third sternites with pair of small orange-brown patches latero-posteriorly; palpi and antenna (but ventrally brown) dark brown; patch on middle and hind coxae, all trochanters partly, apex of all femora, fore and middle tibia tarsi dark brown, remainder of legs black; pterostigma and anterior half of fore wing largely dark brown (Fig. 321), remainder of wing membrane hyaline.

*Male*. Unknown.

#### Biology.

Unknown. Collected in June.

#### Distribution.

China (Yunnan).

#### Etymology.

From “*cordatus*” (Latin for “heart-shaped”) after the cordate shape of the yellow and orange-brown middle lobe of the mesoscutum.

### Taeniogonalos fasciata

(Strand, 1913)

http://species-id.net/wiki/Taeniogonalos_fasciata

**Figures 328–331. F65:**
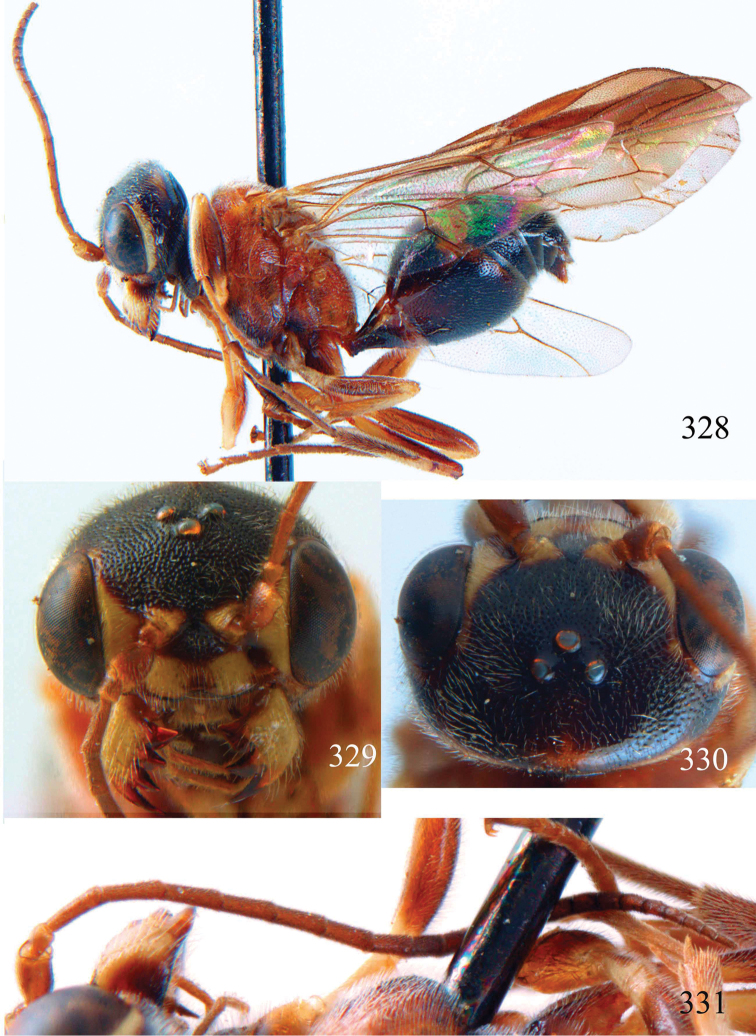
*Taeniogonalos fasciata* (Strand, 1913), holotype, female. **328** Habitus lateral **329** head anterior **330** head dorsal **331** antenna.

Figs 328 [Fig F66] [Fig F67] [Fig F68] [Fig F69] –360

Poecilogonalos fasciata Strand, 1913: 97; Weinstein and Austin 1991: 422; Lelej 1995: 14; He and Lou 2001: 686; He 2004: 73.
Poecilogonalos fasciata rubrothoracica Bischoff, 1913: 153; Weinstein and Austin 1991: 423.
Poecilogonalos magnifica Teranishi, 1929: 147; Chen 1949: 50; Marshakov 1981: 105; Tsuneki 1991: 50; Weinstein and Austin 1991: 423; He and Chen 1992: 1291; Lelej 1995: 14; He and Lou 2001: 687; He 2004: 75. Synonymized by Carmean and Kimsey 1998.
Poecilogonalos fasciata var. *kibunensis* Uchida, 1929: 77; Weinstein and Austin 1991: 422.
Poecilogonalos fasciata var. *interrupta* Chen, 1949: 12; Weinstein and Austin 1991: 422; Lelej 1995: 14. Synonymized by Lelej 1995.
Taeniogonalos fasciata ; Carmean and Kimsey 1998: 67.

#### Type material.

Holotype of *Poecilogonalos fasciata*, ♀ [not ♂ as stated in original description] (SDEI) “[China:] Formosa, Taihorin, IV.[19]10, H. Sauter”, “Holotypus”, “*Poecilogonalos fasciata*, ♂, Strand det.”, “Type”. Paratypes (SDEI): 2 ♀, topotypic, but 7.XI.1911 and 7.XII.1911; 1 ♀, “Formosa, Kosempo, H. Sauter, 1909”, “7.V.”. Holotype of *Poecilogonalos fasciata interrupta*, ♀ (IZCAS) “[China: Anhui] Huangshan, 23.VI.1936”, “*Poecilogonalos fasciata interrupta* Chen”. Holotype of *Poecilogonalos magnifica*, ♀ (OMNH), “Corea, Mt. Kongo, VII.1925, K. Sato”, “Allotype, *Poecilogonalos magnifica*”.

#### Additional material.

1 ♀ topotypic with holotype of *Poecilogonalos fasciata* (ZMB), “[China:] Formosa, Tiahorin, X.[19]10, H. Sauter, S.G.”, “*fasciata* Strand, det. Bischoff”; 1 ♀ (ZJUH), “[China:] Jilin, Zuojia Nature Reserve, 18.VII.1989, Xiao-qiang Du, 948217”; 1 ♂ (ZJUH), “[China:] Liaoning, Benxi, Laotudingzi Nature Reserve, 15–18.VII.2011, Sheng-nan Song, 201100084”; 1 ♂ (ZJUH), “[China:] Shaanxi, Miaotaizi, 16.VII.1981, Jian-hua Wei, 907641”; 1 ♂ (ZJUH), “[China:] Zhejiang, West Mt. Tianmu, Laodian–Xianrending, 1250–1506 m, 6.VI.1989, Jun-Hua He, 890857”; 1 ♂ (ZJUH), “[China:] Zhejiang, Mt. Tianmu, Laodian, 14.VII.1998, Ming-shui Zhao, 200011003”; 1 ♀ + 5 ♂ (ZJUH, RMNH), “[China:] Zhejiang, West Mt. Tianmu, 1–3.VII.2010, Hong-jie Li, SCAU 291; id., but Yun-ling Su, SCAU 293; id., but 18.VI.1983, Jun-hua He, 831809; id., but 4.VI.1994, Fu-liang Hu, 941123”; 1 ♀ (ZJUH), “[China:] Zhejiang, Chunan, Mt. Lao, 28.VI.2004, Tang-xin Wang, SCAU 295”; 1 ♀ (ZJUH), “[China:] Taiwan, Nantou, Riyuetan Pool, 18.VI.2011, Pu Tang, 201101475”; 1 ♂ (ZJUH), “[China:] Fujian, Chongan, Sangang, 8.XI.1989, Jia-she Wang, 20008056”; 1 ♀ (SCAU), “[China:] Fujian, 17.VI.1999, Wen-bin Yao, SCAU 290”; 1 ♀ (SCAU), “[China:] Fujian, Tongmu, 7.VII.1982, Liu-na Kong, SCAU 275”; 1 ♀ (ZJUH), “[China:] Fujian, Fuzhou, 25.IV.1991, Chang-ming Liu, 966573”; 1 ♀ (ZJUH), “[China:] Hunan, Changsha, 8.X.1984, 846510”; 1 ♀ (ZJUH), “[China:] Guangdong, Shaoguan, 12.V.1992, 921107”; 1 ♀ (ZJUH), “[China:] Guangdong, Fengkai, 16.V.1992, Xue-xin Chen, 921576”; 1 ♀ (ZJUH), “[China:] Guizhou, Mt. Leigong, 2–3.VI.2005, Jing-xian Liu, 20059340”; 2 ♀ (ZJUH, RMNH), “[China:] Guizhou, Guiyang, 16.X.1983, Jun-hua He, 834634, 834635”; 1 ♀ (IZCAS), “[China:] Guangxi, Jinxiu, Mt. Shengtang, 900 m, 18.V.1999, Xin-ke Yang, SCAU 288”; 1 ♀ (ZJUH), “[China:] Guangxi, Longsheng, Huaping, 25–26.VI.1982, Jun-hua He, 823652”; 1 ♀ (SCAU), “[China:] Hainan, Mt. Diaoluo, 1.VI.2006, Jing-xian Liu, 200702336, 200805852”; 1 ♀ (ZJUH), “[China:] Hainan, Mt. Yinggeling, 16.XI.2008, Jiang-li Tan, 200805852”.

#### Diagnosis.

Supra-antennal elevations 0.1–0.4 times as long as scapus and outer side of elevations oblique (Figs 330, 341, 352) occipital carina narrow medio-dorsally and smooth (Figs 330, 341, 352); head dorso-posteriorly comparatively sparsely punctate and black with yellow pattern (female, usually less so in male); basal half of antenna dark brown or largely so dorsally (Fig. 331); mesosoma mainly or entirely orange-brown dorsally (Figs 333, 345) or black with extensive yellowish and orange pattern with middle mesoscutal lobe similar to lateral lobes (Fig. 356); notauli posteriorly and scutellar sulcus narrow and both finely crenulate (Figs 333, 345, 356); fore wing with subapical dark brown patch or complete marginal cell and surroundings dark brown (Figs 338, 343, 354); second metasomal tergite more or less with a shallow depression medio-basally; second sternite of female distinctly convex in lateral view (Figs 336, 348, 359; of male partly flattened medio-posteriorly).

**Figures 332–338. F66:**
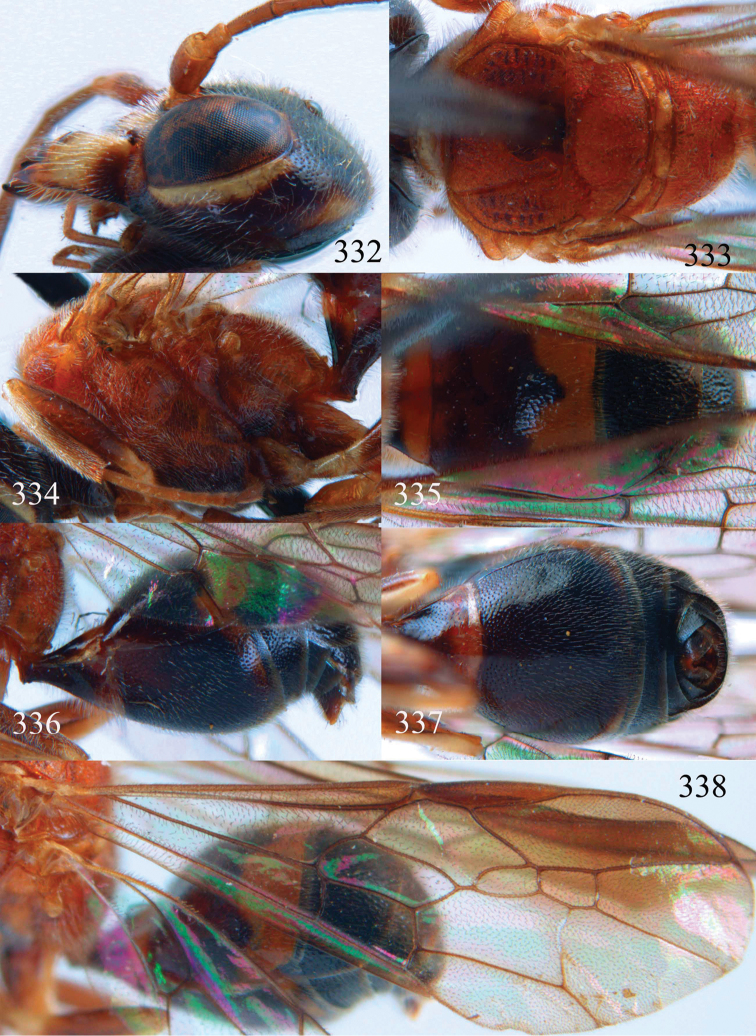
*Taeniogonalos fasciata* (Strand, 1913), holotype, female. **332** Head lateral **333** mesosoma dorsal **334** mesosoma lateral **335** metasoma dorsal **336** metasoma lateral **337** metasoma ventral **338** fore and hind wings.

**Figures 339–341. F67:**
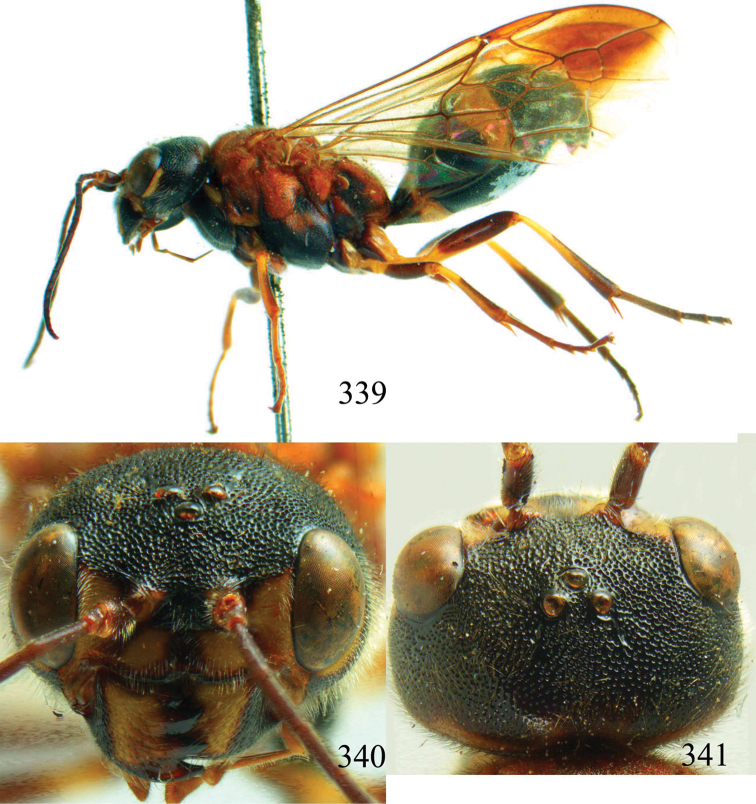
*Taeniogonalos magnifica* (Teranishi, 1929), holotype, female. **339** Habitus lateral **340** head anterior **341** head dorsal.

**Figures 342–349. F68:**
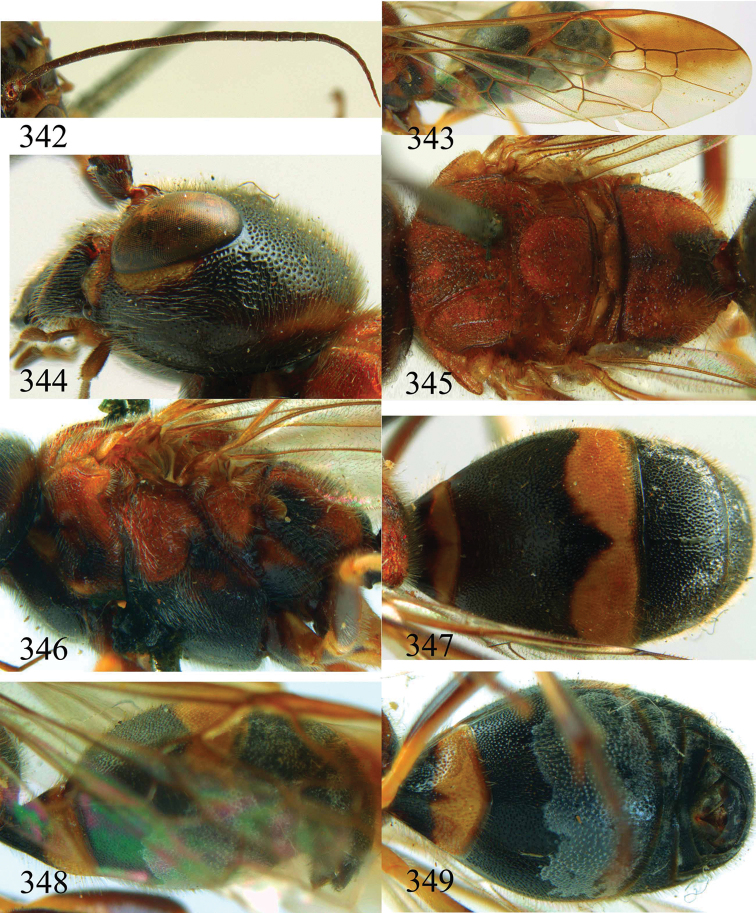
*Taeniogonalos magnifica* (Teranishi, 1929), holotype, female. **342** Antenna **343** fore and hind wings **344** head lateral **345** mesosoma dorsal **346** mesosoma lateral **347** metasoma dorsal **348** metasoma lateral **349** metasoma ventral.

**Figures 350–352. F69:**
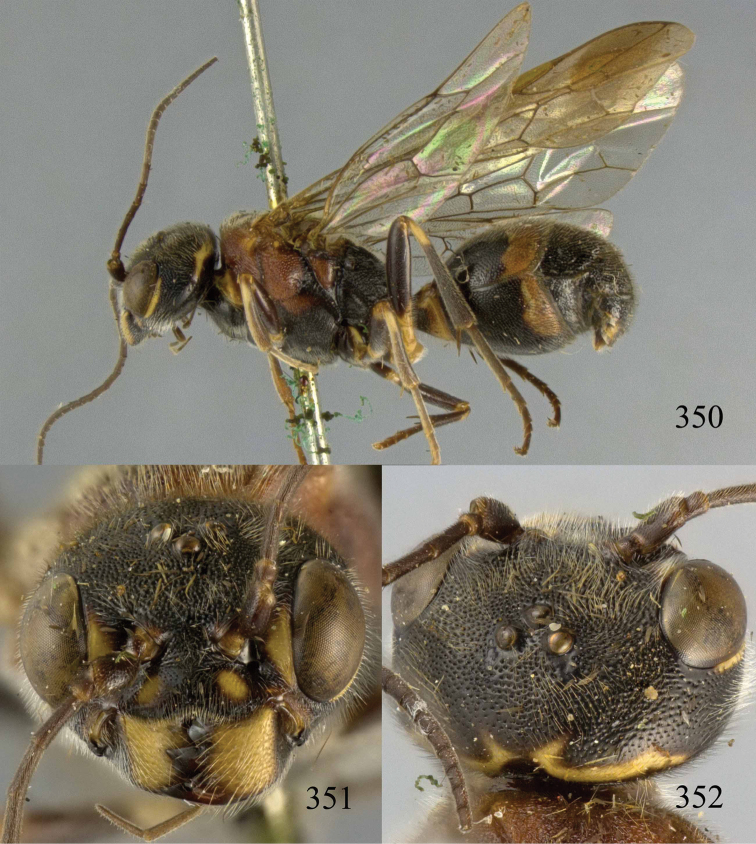
*Taeniogonalos fasciata* f. *interrupta* (Chen, 1949), holotype, female. **350** Habitus lateral **351** head anterior **352** head dorsal.

**Figures 353–360. F70:**
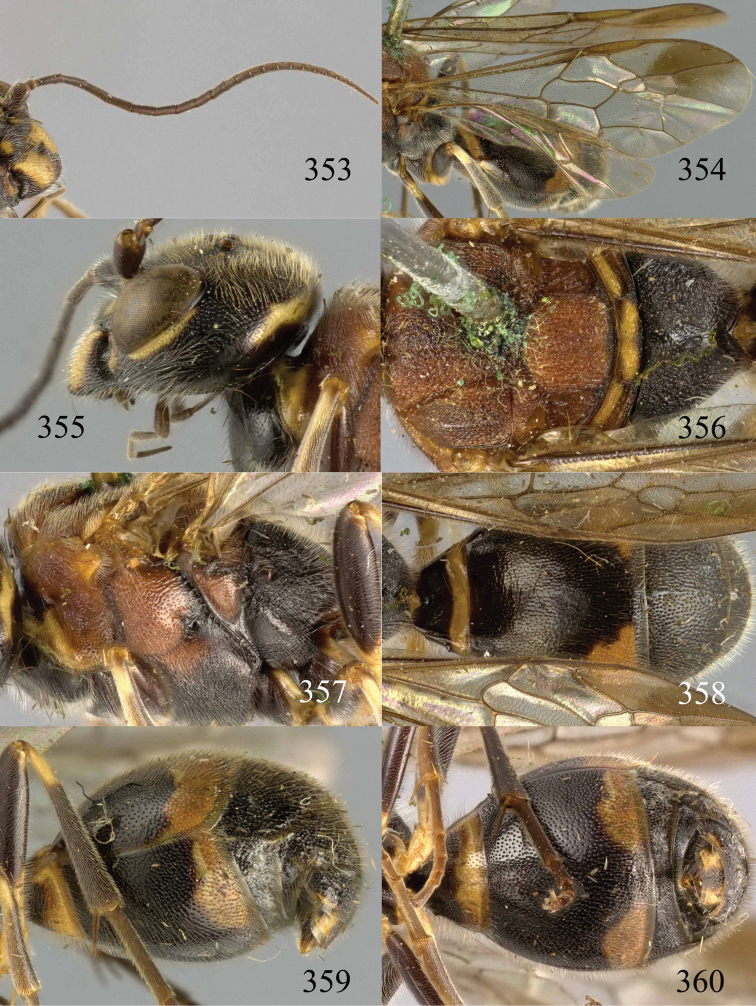
*Taeniogonalos fasciata* f. *interrupta* (Chen, 1949), holotype, female. **353** Antenna **354** fore and hind wings **355** head lateral **356** mesosoma dorsal **357** mesosoma lateral **358** metasoma dorsal **359** metasoma lateral **360** metasoma ventral.

#### Description.

Holotype, female, length of body 6.4 mm (of fore wing 5.9 mm).

*Head*. Antenna with 22 segments; frons densely reticulate-punctate (Fig. 329); vertex reticulate-punctate with distinct smooth interspaces behind stemmaticum and near eyes, becoming smooth posteriorly (Fig. 330); temple rather sparsely punctate near eye and remainder smooth; head slightly narrowed behind eyes, eye in dorsal view as long as temple (Fig. 330); occipital carina narrow medio-dorsally and smooth (Fig. 330); supra-antennal elevations medium-sized (about 0.5 times as long as scapus), outer side oblique, lamelliform and largely smooth, remainder punctate; clypeus distinctly concave and thick medio-ventrally.

*Mesosoma*. Length of mesosoma 1.3 times its height (Fig. 334); mesopleuron below transverse mesopleural groove sublongitudinally rugulose anteriorly and smooth posteriorly, above groove anteriorly rugose, striate dorsally and sparsely punctate posteriorly (Fig. 334); transverse mesopleural groove narrow, shallow and sparsely crenulate; notauli narrow and smooth; middle lobe of mesoscutum coarsely densely rugose, lateral lobe rather finely and densely rugose; scutellar sulcus narrow and moderately crenulate; scutellum reticulate-rugose laterally and anteriorly, medially rather flat and anteriorly near level of mesoscutum; metanotum medially moderately convex, medially depressed and rugulose (Fig. 333); propodeum obliquely striate laterally, mainly finely rugose medially, and smooth posteriorly (Fig. 333); posterior propodeal carina rather wide lamelliform and strongly arched, foramen medially 0.8 times higher than wide basally.

*Wings*. Fore wing: length of vein 1-M 1.5 times as long as vein 1-SR (Fig. 338); second submarginal cell slightly longer than third cell.

*Metasoma*. First tergite 0.5 times as long as apically wide, largely smooth and with distinct elliptical depression medially (Fig. 335); second-sixth tergites densely and rather finely punctate; second tergite depressed medio-basally and with superficial transverse rugose elements; first and second sternites rather sparsely and mainly finely punctate, third-fifth sternites anteriorly coarsely punctate, laterally sparser punctate; second sternite distinctly convex, without medio-apical protuberance but rather steeply depressed medio-posteriorly, and similarly setose as surroundings (Fig. 337); third sternite about 0.2 times as long as second sternite (Fig. 337); hypopygium triangular in ventral view and densely setose (Fig. 337).

*Colour*. Blackish brown; mandible, triangular patch along inner orbita, supra-antennal elevations, nearly entire clypeus, medium-sized stripe along outer orbita and interrupted transverse stripe on posterior vertex, anterior margin of pronotum broadly and dorsally with a narrow stripe, metanotum partly, fore coxa ventrally, middle coxa dorsally largely, trochanters largely, trochantelli, fore tibia anteriorly, base and apex of middle femur, base of hind femur, base of middle and hind tibia, pale yellow or ivory; small medial patch of vertex brown; mesosoma largely orange-brown dorsally and orange-brown laterally, but darkened ventrally; mesosternum dark brown, metasoma blackish brown but broad band on posterior margin of first tergite and of second tergite and of first sternite, broadly interrupted band on posterior margin of second sternite and sixth tergite yellowish brown; palpi yellowish brown, but labial palp partly dark brown; antenna dark brown, but scapus with yellowish ventro-apically (Fig. 331); remainder of legs dark brown; pterostigma brown, but veins, marginal cell and area below it dark brown, remainder of wing membrane subhyaline.

*Variation*. Length of body 5.2–13.4 mm, of fore wing 2.2–12.2 mm; antenna of ♀ with 22 (5), 23 (1), 24 (2), 25 (4) or 26 (2) segments; vertex largely smooth with sparse fine punctures behind stemmaticum to densely punctate; lateral lobe of mesoscutum longitudinally rugulose to coarsely rugose; propodeum mainly rugulose to coarsely rugose; yellow patches of inner orbita wide triangular or linear, supra-antennal elevations largely black only apically brownish to entirely yellow; transverse stripe on posterior vertex orange-yellow or reduced; pair of yellow spots on clypeus large, small or absent or clypeus entirely yellow; mesoscutum and its scutellum more or less with extensive dark brown pattern, propodeum largely orange-brown, largely dark brown except for long pale yellow stripes laterally (Fig. 356) or entirely black; mesosoma largely black laterally or dorsal half orange-brown; yellow stripe on posterior margin of second tergite narrow or interrupted medially, yellow band on posterior margin of first sternite narrow to wide, yellow posterior band of second sternite reduced to wide; legs sometimes largely dark brown; length of vein 1-M of fore wing 1.4–1.8 times as long as vein 1-SR; second metasomal tergite more or less with a shallow depression medio-basally; second sternite usually densely punctate, but sometimes sparsely so.

*Male*. Length of body 5.8–11.1 mm, of fore wing 5.1–10.8 mm; antenna with 22 (3), 23 (1), 24 (3) segments, tyloids linear, in specimen with 22 or 23 segments: 0.8 times as long as segment on 11^th^–14^th^ segments and 0.2 times as long as segment on 15^th^ segment; in specimens with 24 segments: 0.8 times as long as segment on 12^th^–14^th^ segments and 0.2 times as long as segment on 15^th^ segment; second sternite of ♂ partly flattened medio-posteriorly; genitalia internal; head and apex of metasoma of male darker than of female; otherwise variation as in female.

#### Biology.

Unknown. Collected in April–November at 900–1550 m.

#### Distribution.

China (Jilin, Liaoning, Shaanxi, Henan, Anhui, Zhejiang, Taiwan, Fujian, Hunan, Guangdong, Guizhou, Guangxi, Hainan); Russia (Primorskii krai); Japan; Korea (Lelej 1995; Carmean and Kimsey 1998). Also reported from Iran, Malaysia and Indonesia (Carmean and Kimsey 1998), but this needs reconfirmation.

#### Notes.

This species is variable in size, but less in colour. The form of females without apical ivory segments of the metasoma (as in males) is f. *magnifica* (Teranishi, 1929) and the form with the basal part of the marginal cell subhyaline is f. *interrupta* (Chen, 1949).

### Taeniogonalos flavoscutellata

(Chen, 1949) comb. n., re-instated

http://species-id.net/wiki/Taeniogonalos_flavoscutellata

Figs 361 –371

Poecilogonalos flavoscutellata Chen, 1949: 14; He and Chen 1986: 231; Weinstein and Austin 1991: 423; He and Chen 1992: 1291. Synonymized by Tsuneki 1991 with *Taeniogonalos sauteri* Bischoff, 1913.

#### Type material.

Lectotype here designated, ♀ (IZCAS) “[China: Shandong,] Tsinanfou, Long-tong, 500 à 700 m, Musée Heude”, “*Poecilogonalos flavoscutellata* n. sp.”. Paralectotypes: 3 ♀ (IZCAS), topotypic and with identical locality label.

#### Additional material.

1 ♀ (ZJUH), “[China:] Beijing, Mt. Xiang, 22.VII.1972, Ji-kun Yang, No.178”; 1 ♂ (ZJUH), “[China:] Fujian, Mt. Jin, 5.IV.1999, 20003722”; 1 ♀ + 1 ♂ (ZJUH), “[China:] Hunan, Changsha, VI.1984, Ming-de Han, 864555, primary host: *Phanerotoma flava* (Braconidae), det. He & Chen, 24.XII.1984”; 1 ♂ (ZJUH), “[China:] Hunan, Changsha, VI.1982, Jian-wen Peng, 840754, reared from *Locastra muscosalis* (Pyralidae), det. He & Chen, 24.XII.1984”; 1 ♂ (ZJUH), “[China:] Zhejiang, Songyang, 5.IX.1982, Han-lin Chen, reared from *Locastra muscosalis* (Pyralidae), det. He & Chen, 24.XII.1984”.

#### Diagnosis.

Supra-antennal elevations 0.1–0.4 times as long as scapus and outer side of elevations oblique and at least partly yellow (Fig. 363); head posteriorly with extensive yellowish or orange-brown pattern, including a V-shaped yellow or orange pattern behind stemmaticum (Fig. 363); head dorsally often densely reticulate-punctate (Fig. 363); mesopleuron with extensive yellowish pattern (Fig. 368); scutellum antero-medially yellow; anterior half of fore wing subhyaline except for isolated subapical dark patch (Fig. 365); second metasomal sternite with distinct medio-apical protuberance (Fig. 371), in ♀ resulting in circular or rectangular opening between second and following sternites in lateral view (Fig. 370); third sternite of female flat medio-apically in lateral view (Fig. 370).

**Figures 361–363. F71:**
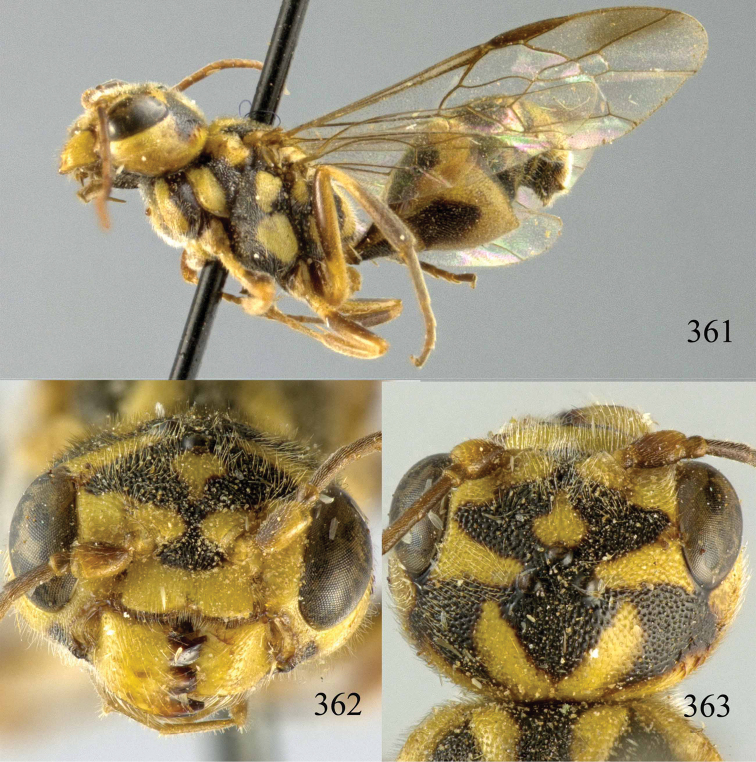
*Taeniogonalos flavoscutellata* (Chen, 1949), lectotype, female. **361** Habitus lateral **362** head anterior **363** head dorsal.

**Figures 364–371. F72:**
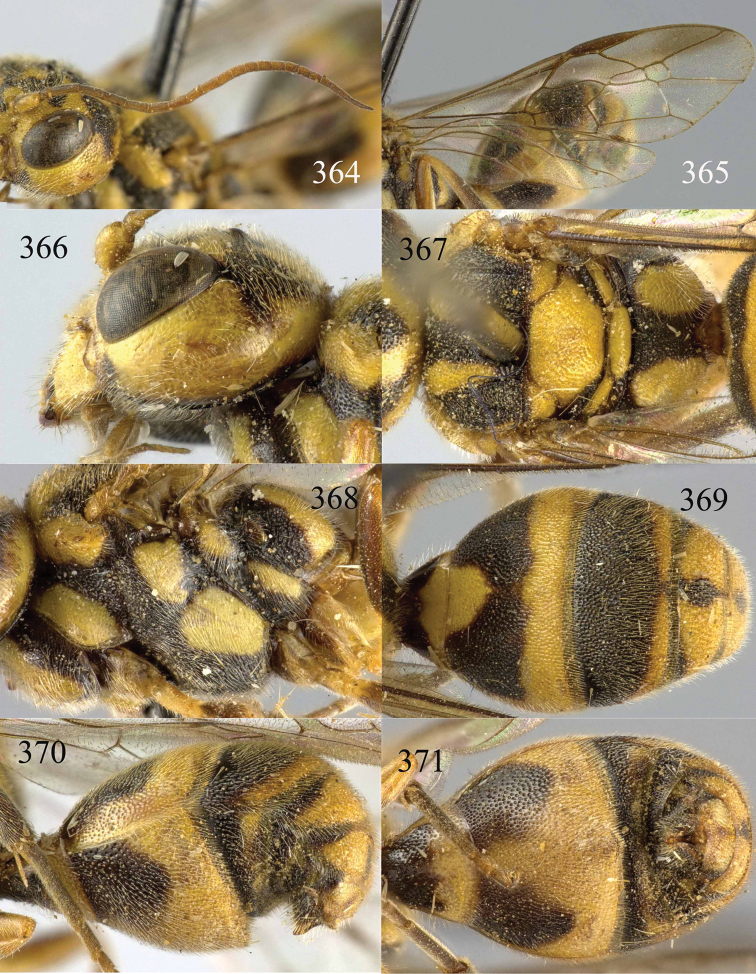
*Taeniogonalos flavoscutellata* (Chen, 1949), lectotype, female. **364** Antenna **365** fore and hind wings **366** head lateral **367** mesosoma dorsal **368** mesosoma lateral **369** metasoma dorsal **370** metasoma lateral **371** metasoma ventral.

#### Description.

Lectotype, female, length of body 8.1 mm (of fore wing 6.8 mm).

*Head*. Antenna with 24 segments; frons reticulate-punctate (Fig. 362); vertex reticulate-punctate behind stemmaticum, becoming spaced punctate (interspaces much wider than width of punctures) posteriorly (Fig. 363); temple largely smooth, with sparse fine punctures (Fig. 363); head gradually narrowed behind eyes, eye in dorsal view 0.9 times as long as temple (Fig. 363); occipital carina narrow, non-lamelliform and smooth medio-dorsally (Fig. 366); supra-antennal elevations medium-sized (about 0.2 times as long as scapus) and their outer side oblique (Fig. 363); clypeus moderately concave and thick medio-ventrally.

*Mesosoma*. Length of mesosoma 1.3 times its height (Fig. 368); mesopleuron densely and coarsely reticulate-rugose anteriorly, densely and finely punctate posteriorly; transverse mesopleural groove narrow, shallow and weakly crenulate; notauli narrow, deep and weakly crenulate; mesoscutum reticulate-rugose (Fig. 367); scutellar sulcus narrow, but complete and weakly crenulate; scutellum densely and finely reticulate-rugose, rather flat and anteriorly near level of mesoscutum; metanotum medially slightly convex, not protruding and finely rugose (Fig. 367); propodeum densely reticulate-rugose (Fig. 367); posterior propodeal carina thick lamelliform and strongly arched, foramen medially 0.7 times higher than wide basally.

*Wings*. Fore wing: vein 1-M 1.5 times as long as vein 1-SR (Fig. 365).

*Metasoma*. First tergite 0.7 times as long as apically wide, smooth and with distinct elliptical depression medially (Fig. 369); second–sixth tergites densely reticulate-punctate; sternites densely punctate; second sternite distinctly convex in lateral view, medio-apical protuberance triangularly protruding behind apical margin of sternite and posteriorly truncate (Fig. 370); third sternite about 0.2 times as long as second sternite (Fig. 371); hypopygium triangular in ventral view.

*Colour*. Yellow with black markings; frons largely yellow, area above and between supra-antennal elevations largely black but with a heart-shaped yellow spot below anterior ocellus, black marks medio-ventrally extending to base of clypeus; clypeus entirely yellow; mandible yellow with teeth dark brown; vertex largely black with a V-shaped yellow patch posteriorly and postero-lateral area above occipital carina yellow; temple entirely yellow; mesosoma laterally black with following yellow markings: broad oblique stripe on lower propleuron, stripe on upper pronotum and lower pronotum, broad oblique but medially interrupted stripe on mesopleuron and metapleuron; mesosoma dorsally yellow with following black markings: broad median stripes on middle lobe of mesoscutum, lateral lobes of mesoscutum, broad median stripe on propodeum; metasoma yellow with following black markings: anterior half of first tergite and sternite, anterior 0.7 of second tergite except for broad median patch, anterior 0.8 of third tergite, small median patch on fourth and fifth tergites, broad antero-lateral patches on second sternite, anterior half of third sternite, entirely fourth and fifth sternites; palpi yellowish brown; antenna dark brown; dorsal coxae, trochanters and trochantelli, base and apex of femora yellow, remainder of legs dark brown; pterostigma dark brown, marginal cell more or less dark brown, remainder of wing membrane subhyaline.

*Variation*. Length of body 7.0–8.4 mm, of fore wing 6.7–7.5 mm; antenna of ♀ with 25 segments; length of vein 1-M of fore wing 1.4–1.6 times as long as vein 1-SR; palpi yellow; antenna yellowish brown; legs paler; dark brown patch of marginal cell of fore wing weakly to distinctly pigmented.

*Male*. Length of body 7.6–8.5 mm, of fore wing 6.0–6.7 mm; antenna with 25–26 segments, tyloids linear, 0.7 times as long as antennal segment on 11^th^–14^th^ segments and about half as long on 10^th^ and 15^th^ segments with a remnant on 16^th^ segment; genitalia internal.

#### Biology.

Hyperparasitoid of *Phanerotoma flava* (Hymenoptera: Braconidae) in *Locastra muscosalis* caterpillars (Lepidoptera: Pyralidae) (He and Chen 1986). Collected in April, June and July; reared in December.

#### Distribution.

China (Beijing, Shandong, Zhejiang, Fujian, Hunan).

### Taeniogonalos formosana

(Bischoff, 1913)

http://species-id.net/wiki/Taeniogonalos_formosana

**Figures 372–374. F73:**
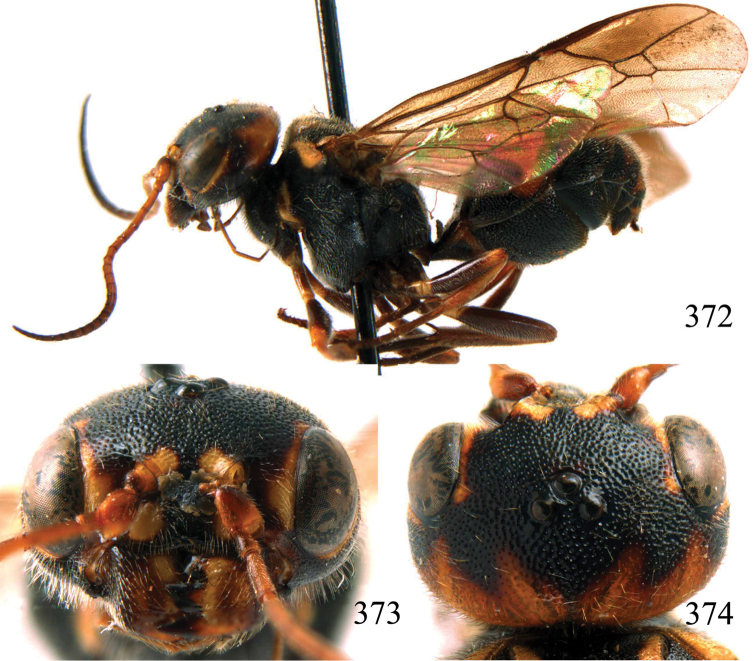
*Taeniogonalos formosana* (Bischoff, 1913), holotype, female. **372** Habitus lateral **373** head anterior **374** head dorsal.

Figs 372 [Fig F74] [Fig F75] [Fig F76] [Fig F77] –405

Poecilogonalos formosana Bischoff, 1913: 151; Tsuneki 1991: 51; Weinstein and Austin 1991: 423.
Taeniogonalos formosana ; Carmean and Kimsey 1998: 67.
Poecilogonalos intermedia Chen, 1949: 12; Marshakov 1981: 106; Weinstein and Austin 1991: 423; He and Lou 2001: 686. Syn. n.
Taeniogonalos intermedia ; Carmean and Kimsey 1998: 68.
Poecilogonalos unifasciata Chen, 1949: 13; He 2004: 75. Synonymized by Marshakov 1981 with *Poecilogonalos maga* (Teranishi, 1929). Syn. n.
Poecilogonalos unifasciatas (!); He and Lou 2001: 687.

#### Type material.

Holotype of *Poecilogonalos formosana*, ♀ (ZMB), “[China:] Formosa, Taihorin, X.[19]10, H. Sauter S.G.”, “*formosana* Bisch.*, det. Bischoff”, “Type”. Holotype of *Poecilogonalos intermedia*, ♀ (IZCAS), “[China: Zhejiang,] T’ienmu Shan (= Mt. Tianmu), Musée Heude”, “25.VI.[19]36, O. Piel”, “*Poecilogonalos intermedia* n. sp.”. Holotype of *Poecilogonalos unifasciata*, ♂ (IZCAS), “[China: Zhejiang,] Tienmushan, 24.VII.1937, Ouchi”, “*Poecilogonalos unifasciata* Chen”.

#### Additional material.

4 ♀ + 3 ♂ (ZJUH, RMNH) “[China:] Henan, Luoshan County, Mt. Ling, 22.V.2000, Ping Cai, 200101850, 200101863, 200101872, 200101865, 200101856, 200101894, 200101851”; 2 ♂ (ZJUH), “[China:] Jilin, Dongliao, 22–31.VII.1988, Xiao-Ming Lou, 888612, 888344”; 1 ♀ (SCAU), “[China:] Jilin, Zuojia Nature Reserve, 15.VIII.2012, Liang Fan, SCAU 358”; 2 ♀ (ZJUH), “[China:] Shanxi, Lishan Nature Reserve, 26–30.VII.2012, Zheng Liu, 201200420, 201200429”; 1 ♀ + 1 ♂ (SCAU) “[China:] Ningxia, Mt. Liupan, Qiuqianjia, 9–11.VII.2009, Guang-yue Wang, SCAU 022; id., but 8.VII.2008, 200801031”; 1 ♂ (SCAU) id., but 6.VII.2008, Gang Yao, 200800478; 2 ♀ + 1 ♂ (ZJUH) “[China:] Henan, Song County, Mt. Baiyun, 1400 m, 26.VII.2003, Yan-bing Shen, 20047322; id., but 22.VII.2003, Tao Yang, 20047316; id., but 24.VII.2003, Jing-bo Li, 20047320”; 1 ♂ (ZJUH) “[China:] Henan, Dengfeng, Mt. Shaoshi, 800 m, 9.VI.2000, Xiao-cheng Shen, 20047327”; 5 ♀ + 1 ♂ (ZJUH) “[China:] Zhejiang, Mt. Tianmu, 18.VI.1983, Yun Ma, 831376, 831380; id., but 27.VII.1988, Ying Qian, 940233; id., 21.VII.1987, Xue-xin Chen, 940234; id., but 3.VII.2010, Qing-ling Miu, SCAU 074, 079”; 5 ♀ (ZJUH, RMNH), “[China:] Zhejiang, Mt. Tianmu, Xianrending, 1509 m, Qiong Wu, 20034562; id., but 20.VII.1998, MT, 992747; id., but 14.IX.1998, 200010706; id., but 5.IX.1998, light trap, 200010702”; 1 ♀ + 3 ♂ (ZJUH, RMNH) “[China:] Zhejiang, Longquan, Mt. Fengyang, 30.V.2007, Sheng-long Liu, 200704937; id., but 11.VII.1984, 8433553,; id., but 31.VII.2008, Jing-xian Liu, 200801104, 200801105”; 1 ♂ (ZJUH) “[China:] Fujian, Chong’an, Mt. Huanggang, 30.VII.1985, Ming-hui Liu, SCAU 091”; 1 ♀ (ZJUH) “[China:] Hunan, Mt. Tianping, 26.VIII.1981, Xin-wang Tong, 896471”; 2 ♂ (RMNH) “[China:] Guangdong, Nanling Nature Reserve, VI.2011, Zai-fu Xu et al.”; 2 ♀ (ZJUH) “[China:] Sichuan, Mt. Emei, 7.VII.2009, Jiang-li Tan, 200906432, 200906432”; 1 ♀ (ZJUH) “[China:] Guizhou, Daozhen, Dashahe Nature Reserve, 1360 m, 21.VIII.2004, Qiong Wu, 20047404”; 1 ♀ (ZJUH) “[China:] Guizhou, Kuankuoshui National Nature Reserve, 1450 m, 6.VIII.2003, Qiong-zhang Song, SCAU 359”; 1 ♂ (ZJUH) “[China:] Tibet, Yarlung Zangbo Grand Canyon, 10.VI.2009, Jiang-li Tan, 200906509”; 1 ♀ (ZJUH) “[China:] Yunnan, Tengchong, Houqiao Town, 1.VI.2009, Jiang-li Tan, 200907082”; 1 ♀ (ZJUH) “[China:] Yunnan, Dali, Yunlong, 3.VI.2009, Jiang-li Tan, 200906611”; 1 ♀ (ZJUH) “[China:] Yunnan, Kunming, 18.V.1981, Jun-hua He, 814751”.

#### Diagnosis.

Supra-antennal elevations 0.1–0.4 times as long as scapus and outer side of elevations moderately oblique (angle about 35°; Figs 374, 385, 396); area between elevations comparatively shallowly depressed (Figs 374, 385, 396); head anteriorly and posteriorly, mesosoma dorsally and pronotum laterally more or less with a yellowish or brownish pattern (Figs 373, 374, 378, 379), including a more or less developed V-shaped yellow or orange pattern behind stemmaticum, area besides stemmaticum variable, often dark brown (Fig. 374); V-shaped pale area of head dorsally with smooth interspaces between punctures (Fig. 374); clypeus shallowly emarginate medio-ventrally (Fig. 373); middle mesoscutal lobe similar to lateral lobes, black or yellow laterally and black medially (Figs 378, 389, 401); notauli smooth posteriorly (Fig. 378); mesopleuron black or at most with medio-dorsal orange-brown patch and metapleuron black (Fig. 379); third submarginal cell of fore wing 0.7–1.0 times as long as second submarginal cell (Fig. 376); basal cell and posterior half of first submarginal cells of fore wing subhyaline (but costal cell more or less dark brown), distinctly paler than marginal cell (Fig. 376); propodeal foramen comparatively wide and less arched (Fig. 389, 401); second tergite bicoloured (Fig. 380), anteriorly black and posteriorly yellow; third–fifth metasomal tergites with yellow patches postero-laterally; second sternite of both sexes without medio-apical protuberance (Fig. 382), distinctly convex and no opening between second and following sternites in lateral view (Fig. 382); third sternite of female without apical ledge (Fig. 382); sixth tergite with black central patch, surrounded by U-shaped yellow patch (Fig. 382).

**Figures 375–382. F74:**
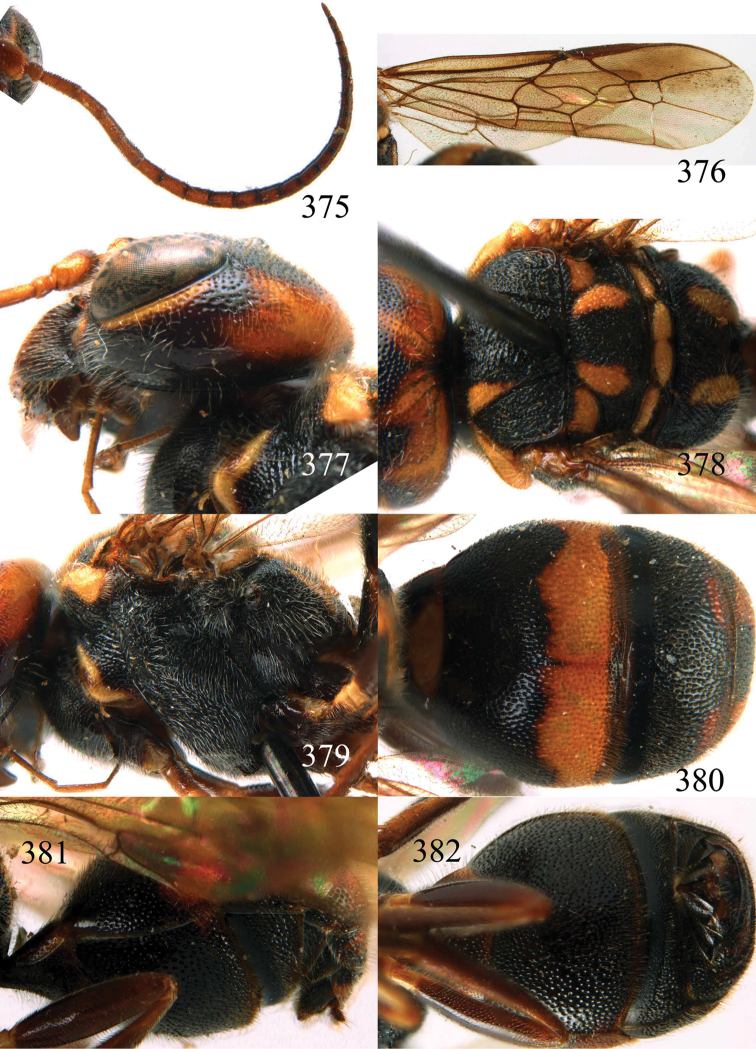
*Taeniogonalos formosana* (Bischoff, 1913), holotype, female. **375** Antenna **376** fore and hind wings **377** head lateral **378** mesosoma dorsal **379** mesosoma lateral **380** metasoma dorsal **381** metasoma lateral **382** metasoma ventral.

**Figures 383–385. F75:**
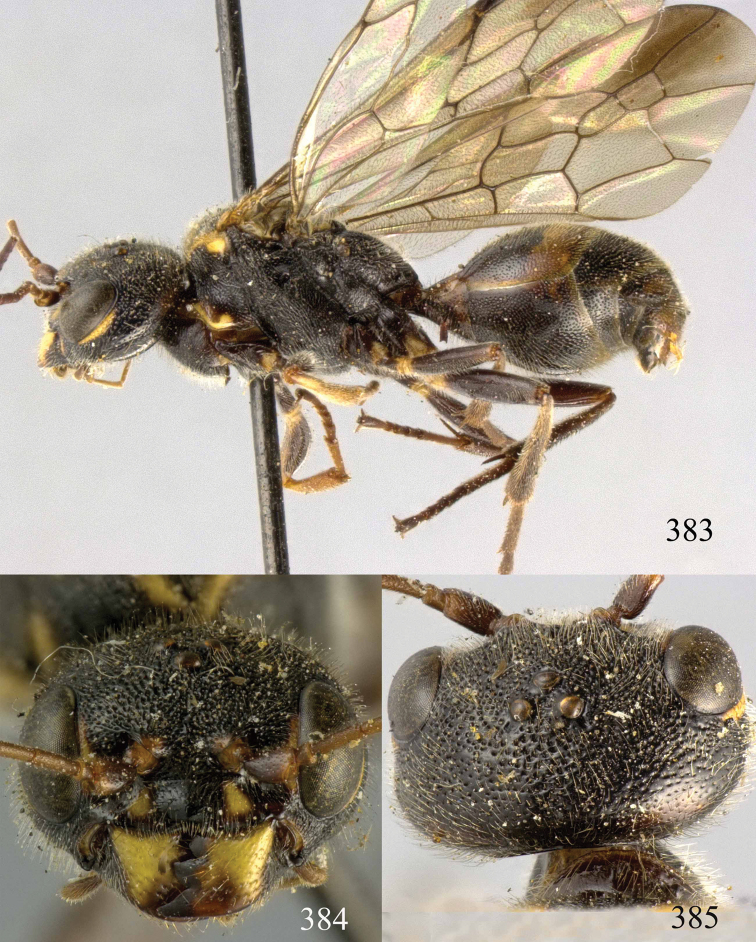
*Taeniogonalos intermedia* (Chen, 1949), lectotype, female. **383** Habitus lateral **384** head anterior **385** head dorsal.

**Figures 386–393. F76:**
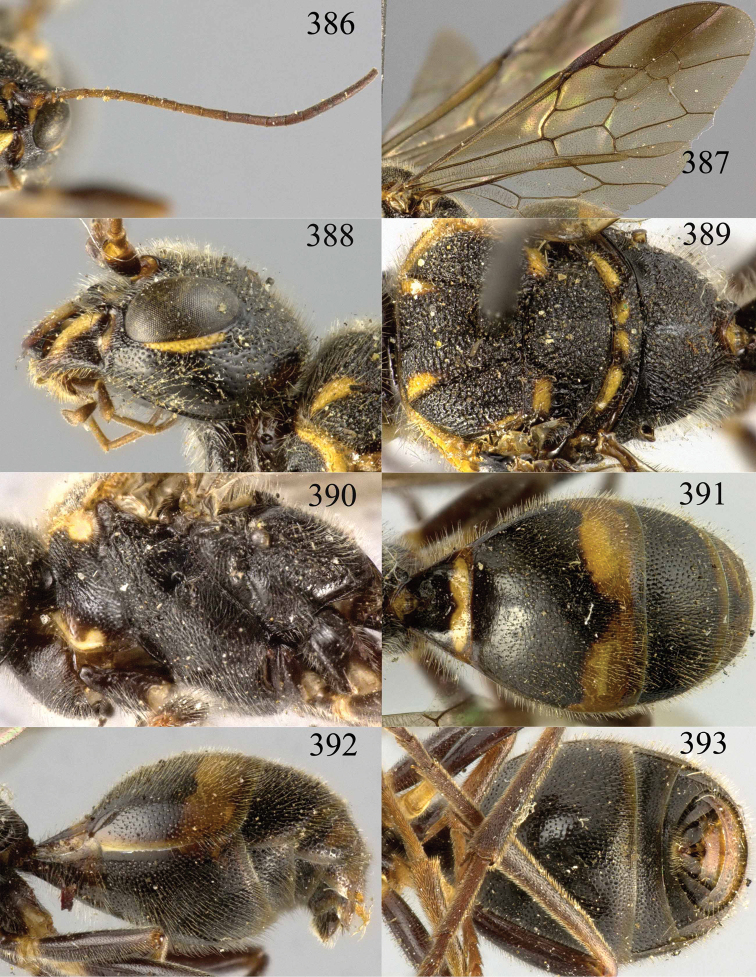
*Taeniogonalos intermedia* (Chen, 1949), lectotype, female. **386** Antenna **387** fore and hind wings **388** head lateral **389** mesosoma dorsal **390** mesosoma lateral **391** metasoma dorsal **392** metasoma lateral **393** metasoma ventral.

**Figures 394–397. F77:**
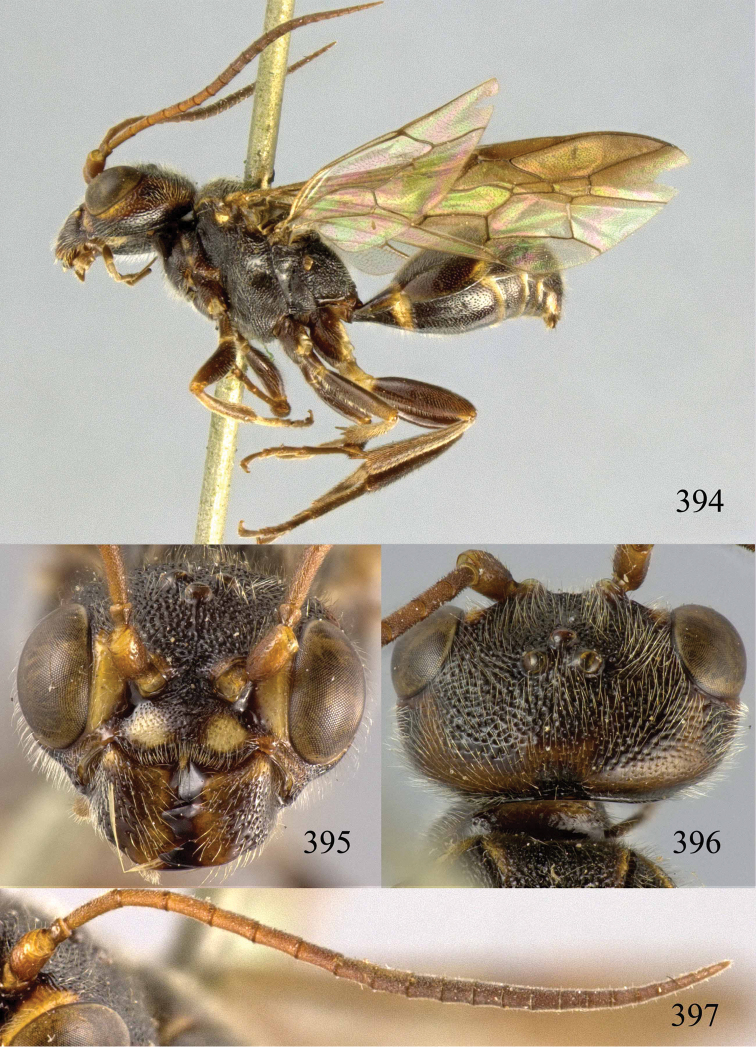
*Taeniogonalos unifasciata* (Chen, 1949), lectotype, male. **394** Habitus lateral **395** head anterior **396** head dorsal **397** antenna.

**Figures 398–405. F78:**
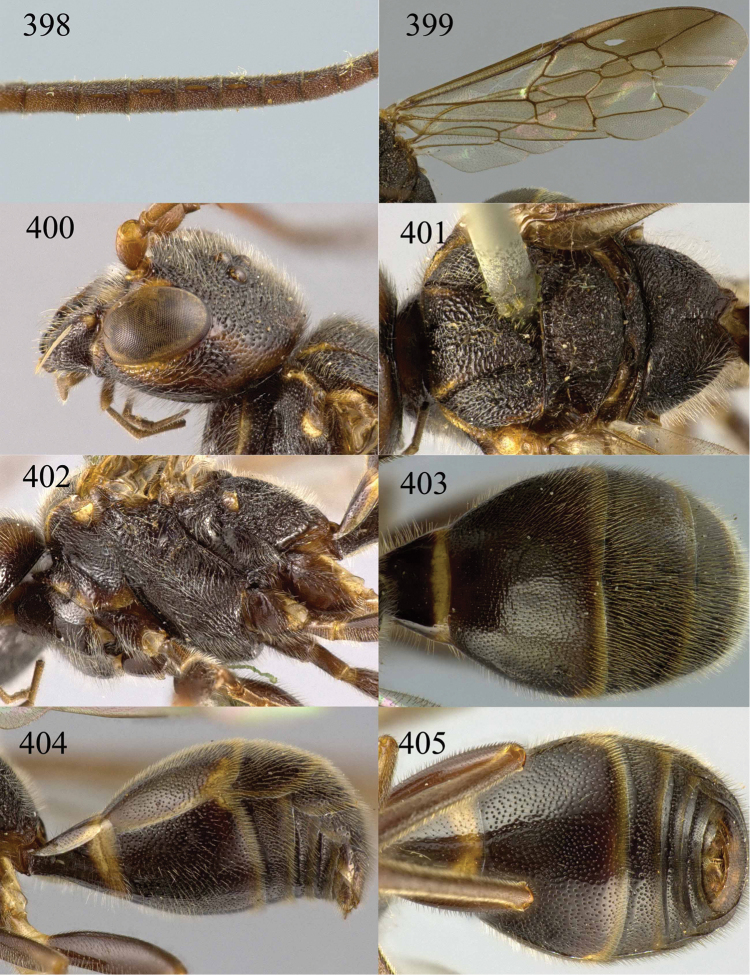
*Taeniogonalos unifasciata* (Chen, 1949), lectotype, male. **398** Tyloids on 11^th^–16^th^ segments of antenna **399** fore and hind wings **400** head lateral **401** mesosoma dorsal **402** mesosoma lateral **403** metasoma dorsal **404** metasoma lateral **405** metasoma ventral.

#### Description.

Holotype, female, length of body 7.9 mm (of fore wing 7.1 mm).

*Head*. Antenna with 24 segments; frons densely punctate and narrow smooth interspaces; vertex punctate with medium-sized smooth interspaces but pale posterior area with wide interspaces and distinctly shiny (Fig. 374), with medium-sized setae; temple largely smooth with sparse fine punctures (Fig. 377), but less sparsely near eye; head subparallel behind eyes, eye in dorsal view as long as temple (Fig. 374); occipital carina narrow, non-lamelliform, smooth (Fig. 374); supra-antennal elevations medium-sized (about 0.5 times as long as scapus), outer side oblique (angle about 35°) lamelliform and largely smooth (Fig. 374); area between elevations shallowly concave; clypeus slightly concave and thick medio-ventrally.

*Mesosoma*. Length of mesosoma 1.2 times its height (Fig. 379); mesopleuron densely and coarsely punctate-rugose, with satin sheen; transverse mesopleural groove medium-sized, shallow and distinctly crenulate; notauli narrow, deep and smooth; middle lobe of mesoscutum transversely rugose, lateral lobes vermiculate-rugose and partly rather shiny (Fig. 378); scutellar sulcus complete, narrow and finely crenulate; scutellum densely rugose but largely smooth posteriorly, convex laterally, near level of mesoscutum, flattened medially and posteriorly; metanotum medially flattened and finely rugose (Fig. 378); propodeum largely obliquely rugose (Fig. 378); posterior propodeal carina thick lamelliform and medium-sized, foramen moderately arched and 1.2 times higher than wide basally.

*Wings*. Fore wing: vein 1-M as long as vein 1-SR (Fig. 376), distinctly curved and vein 1-SR widened anteriorly; second submarginal cell about as long as third cell.

*Metasoma*. First tergite 0.4 times as long as apically wide, largely smooth and with shallow linear depression medially (Fig. 380); other tergites largely densely punctate (Fig. 380); all sternites densely punctate, but third sternite finely coriaceous anteriorly; second sternite moderately convex and medio-posteriorly truncate and densely setose in lateral view (Fig. 381); third sternite distinctly depressed posteriorly.

*Colour*. Black; orbita yellow, narrow but widened near antennal sockets, supra-antennal elevations largely yellow and connected to yellow patch (Fig. 374), clypeus with pair of large yellow patches; head posteriorly brownish-yellow, including two pairs of oblique patches; mandibles largely dark brown with some yellow near base of teeth; mesosoma laterally black except for dorsal and ventral yellow patches of pronotal side; middle lobe of mesoscutum with pair of triangular yellow patches anteriorly; tegulae yellow; scutellum laterally and axilla with pair of large yellow patches; metanotum with a large medial and a large lateral patch yellow; propodeum pair of large yellow patches (Fig. 378); first tergite ivory medio-posteriorly, second tergite widely yellow posteriorly, third tergite with pair of small yellow patches postero-laterally; fourth–sixth tergites with pair of large yellow patches; metasoma ventrally black but apical margin of first sternites yellowish and apex of hypopygium brownish (Fig. 382); palpi and apical half of antenna brown; remainder of antenna mainly light brown; apex of trochanters, trochantelli largely, anterior stripe on fore tibia, base of all femora and apex of fore and middle femora, base of middle and hind tibiae pale yellow; remainder of tibiae and tarsi more or less dark brown; pterostigma and marginal cell of fore wing largely and area below it dark brown; remainder of wing membrane slightly infuscate.

*Male*. Similar to the pale and dark (= “*unifasciata*”) forms of the female, but vertex and frons largely black; second sternite distinctly flattened medially; tyloids linear.

*Variation*. The pale and typical form has the supra-antennal sockets largely yellow (Fig. 374), but these are largely or entirely black or dark brown in the dark form (Fig. 385). The vertex is partly yellowish brown or orange posteriorly (Fig. 374), sometimes including posterior part of frons, but rather obscurely so in the dark form (Fig. 385); lobes of mesoscutum sometimes largely orange laterally; yellow medial patch of metanotum is sometimes divided into two patches; metasoma finely to coarsely punctate dorsally; second–fifth sternites laterally black or partly orange; second submarginal cell of fore wing 1.0–1.5 times as long as third cell.

#### Biology.

Collected in May–August and October at 800–1509 m.

#### Distribution.

China (Jilin, Shanxi, Ningxia, Henan, Fujian, Taiwan, Zhejiang, Guangdong, Sichuan, Yunnan, Guizhou, Tibet); Russia; Japan.

#### Notes.

*Poecilogonalos intermedia* Chen, 1949 (♀), and *Poecilogonalos unifasciata* Chen, 1949 (♂), are synonymized here with *Taeniogonalos formosana*, because they share the less steeply protruding supra-antennal protruberances (angle about 35°; Figs 374, 385, 396), the more or less brownish or yellowish vertex posteriorly, head anteriorly and mesosoma dorsally with a yellowish pattern and differ only in colour from *Taeniogonalos formosana* (Bischoff) and intermediates are present. If the melanistic form should be named, then f. *intermedia* (Chen) is available; *Trigonalys lachrymosa* (Westwood, 1874), comb. n. from Philippines has a similar colour pattern but a different venation according to the original description. *Taeniogonalos taihorina* (Bischoff, 1914) is very similar to the dark form of *Taeniogonalos formosana*, but differs by having the area between the supra-antennal protuberances distinctly concave and the protuberances more steeply protruding laterally (angle about 45°), the pronotum laterally, the head anteriorly and the mesosoma dorsally entirely black.

### Taeniogonalos geminata sp. n.

http://zoobank.org/F2330C5A-0F04-4A8C-8B8A-A03E51520F76

http://species-id.net/wiki/Taeniogonalos_geminata

Figs 406 –416

#### Type material.

Holotype, ♀ (ZJUH) “[China:] Zhejiang, Mt. Tianmu, 18.VI.1983, Yun Ma, 831522”.

#### Diagnosis.

Outer side of supra-antennal elevations oblique and elevations about 0.3 times as long as scapus (Fig. 408); occipital carina strongly widened medio-dorsally and distinctly lamelliform (Fig. 408); head posteriorly entirely black and dorsally punctate with distinct smooth interspaces (Fig. 408); middle mesoscutal lobe similarly coloured to lateral lobes, yellow latero-posteriorly, black or yellow laterally and black medially (Fig. 412); scutellum with pair of medium-sized yellow patches medially (Fig. 412); anterior half of fore wing only below pterostigma or subapically dark brown and remainder subhyaline (Fig. 410); mesosoma with moderately limited yellowish pattern dorsally (Fig. 412); second sternite of female distinctly convex (Fig. 415; probably less of ♂); third sternite of female without protuberance (Fig. 416); metasoma with limited pale pattern (Figs 414, 415, 416).

**Figures 406–408. F79:**
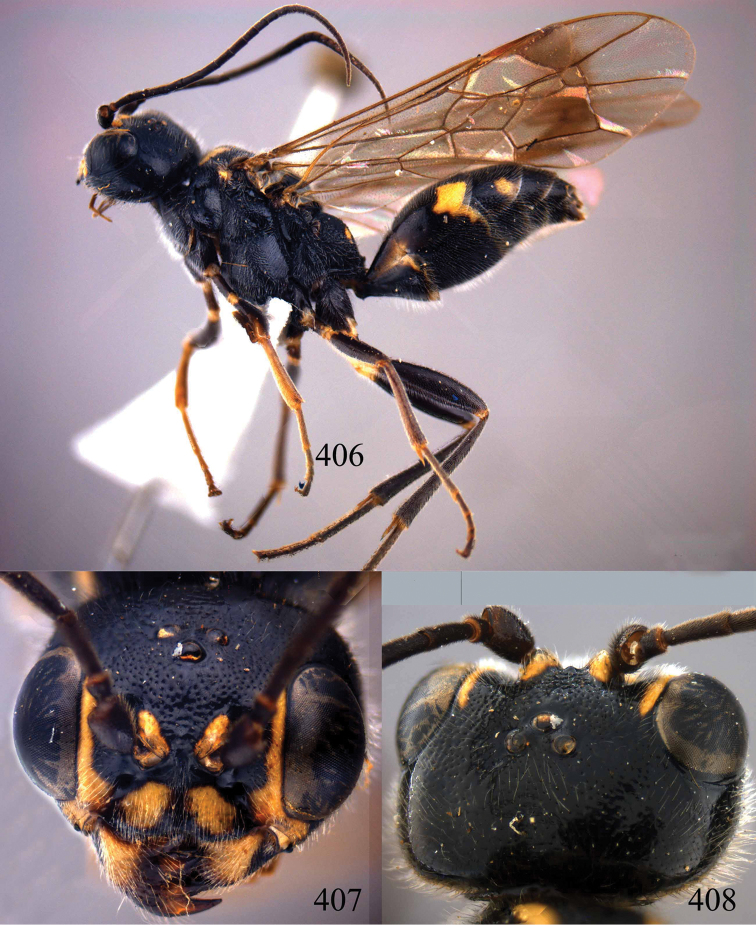
*Taeniogonalos geminata* sp. n., holotype, female. **406** Habitus lateral **407** head anterior **408** head dorsal.

**Figures 409–416. F80:**
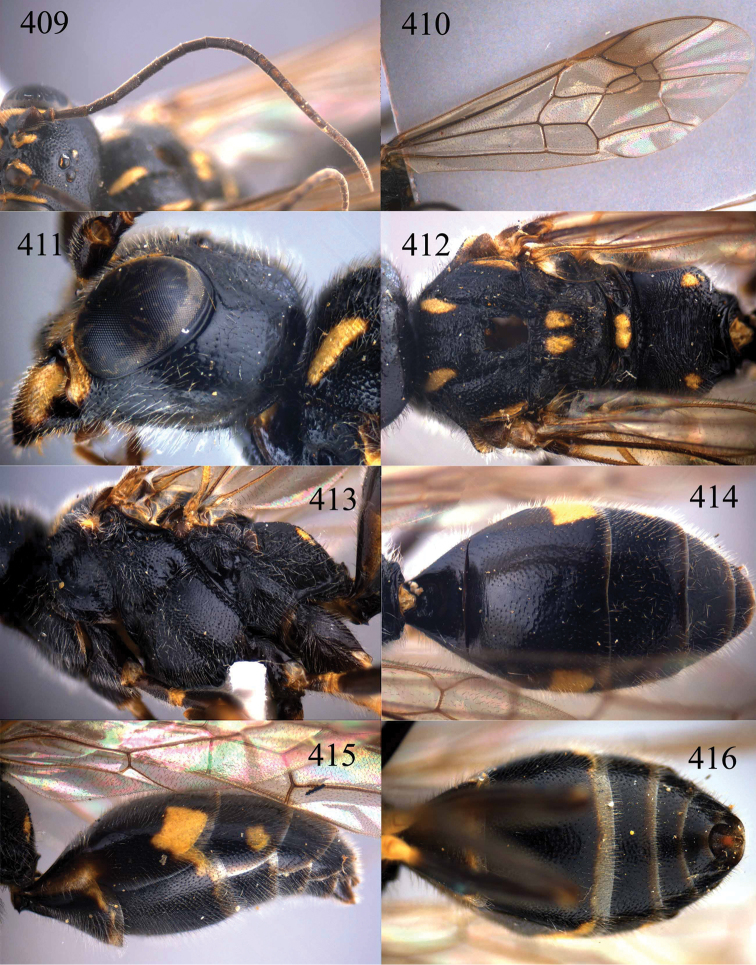
*Taeniogonalos geminata* sp. n., holotype, female. **409** Antenna **410** fore wing **411** head lateral **412** mesosoma dorsal **413** mesosoma lateral **414** metasoma dorsal **415** metasoma lateral **416** metasoma ventral.

#### Description.

Holotype, female, length of body 11.1 mm (of fore wing 7.6 mm).

*Head*. Antenna with 26 segments; frons densely punctate and no smooth interspaces; vertex punctate with large smooth interspaces but patch behind stemmaticum and near eyes with narrow interspaces and distinctly shiny (Fig. 408), with rather long setae; temple largely smooth with sparse fine punctures (Fig. 411); head gradually narrowed behind eyes, eye in dorsal view 1.4 times as long as temple (Fig. 408); occipital carina strongly widened medio-dorsally and distinctly lamelliform, smooth (Fig. 408); supra-antennal elevations medium-sized (about 0.3 times as long as scapus), outer side oblique and largely smooth except for sparse punctures; clypeus slightly concave and thick medio-ventrally.

*Mesosoma*. Length of mesosoma 1.6 times its height (Fig. 413); mesopleuron densely and finely rugulose-punctate, with satin sheen; notauli moderately wide, deep and moderately crenulate; mesoscutum largely finely and densely rugulose-punctate and with satin sheen, rather contrasting with shiny head (Fig. 412); scutellar sulcus complete, strongly wide and crenulate; scutellum densely rugose-punctate, convex anteriorly and flattened posteriorly and near level of mesoscutum; metanotum medially protruding, obtuse and densely rugose-punctate (Fig. 412); propodeum largely transversely strigose, reticulate-rugose, smooth and shiny posteriorly and antero-laterally (Fig. 412); posterior propodeal carina thick lamelliform.

*Wings*. Fore wing: length of vein 1-M 1.4 times as long as vein 1-SR (Fig. 410).

*Metasoma*. First tergite 0.5 times as long as apically wide, largely smooth and with shallow elliptical depression medially (Fig. 412); other tergites largely smooth with sparse punctures (Fig. 412); all sternites densely finely punctate; second sternite slightly convex.

*Colour*. Black; frons with narrow yellow stripes along inner orbita extend to malar space, supra-antennal elevations yellow, clypeus with pair of yellow patches, remainder of head black; mandibles largely yellow with dark brown teeth; mesosoma laterally black except for dorsal yellow patches of pronotal side; middle lobe of mesoscutum with pair of elongate yellow patches anteriorly, lateral lobes of mesoscutum with narrow yellow patch along outer margin; scutellum with pair of wide yellow patches anteriorly; metanotum with one large medial yellow patch; propodeum pair of small yellow patches (Fig. 412); metasoma dorsally black, lateral margin of first tergite ivory and remainder black, second tergite with pair of large yellow patches postero-laterally, third tergite with pair of small yellow patches postero-laterally; metasoma ventrally black (Fig. 416), apical margin of all sternites ivory; palpi and antenna dark brown; patch on middle and hind coxae, fore and middle trochanters largely, anterior stripe on fore femur, base of all femora and apex of fore and middle femora, fore tibia anteriorly, base of middle and hind tibiae pale yellow; remainder of tibiae and tarsi more or less dark brown; pterostigma and apical half of marginal cell of fore wing and area below it dark brown; remainder of wing membrane slightly infuscate.

*Male*. Unknown.

#### Biology.

Unknown. Collected in June.

#### Distribution.

China (Zhejiang).

#### Etymology.

Name derived from “*geminus*” (Latin for “twin”), because of the pair of yellow patches on the scutellum.

### Taeniogonalos gestroi

(Schulz, 1908) comb. n., re-instated

http://species-id.net/wiki/Taeniogonalos_gestroi

Figs 417 –427

Poecilogonalos thwaitesi var. *gestroi* Schulz, 1908: 24; Bischoff 1938: 8.
Poecilogonalos pulchella gestroi ; Weinstein and Austin 1991: 423.
Poecilogonalos thwaitesi thwaitesi ; Weinstein and Austin 1991: 424 (p.p.).
Taeniogonalos thwaitesii ; Tsuneki 1991: 51; Carmean and Kimsey 1998: 68 (p.p.; not Westwood 1874).

#### Additional material.

1 ♀ (ZMB), “[China:] Formosa, Taihorin [= Dalin, Chiayi County], V.[19]09, H. Sauter, S.G.”, “*pulchella* Westw., det. Bischoff”; 1 ♀ (IZCAS) “[China:] Yunnan, Naban River Watershed National Nature Reserve, 22.13059°N, 100.66817°E, 753m, 6.VI.2008, MT, A. Weigel, IOZ(E)1903927”; 1 ♂ (IZCAS) “[China:] Jiangsu, Nanjing, Lingusi, 12.VII.1957, IOZ(E)1495290”; 1 ♂ (IZCAS) “[China:] Guangxi, Pingxiang, 12.IV.1963, Shu-yong Wang, IOZ(E)1495289”; 11 ♀ + 5 ♂ (IZCAS) “[China:] Yunnan, Naban River Watershed National Nature Reserve, 22.13059°N, 100.66817°E, 753m, 6.VI.2008, MT, A. Weigel, IOZ(E)1903925, IOZ(E)1903926; id., but 8.VI.2008, IOZ(E)1910500; id., but 10.VI.2008, IOZ(E)1903929; id, but 16.VI.2009, Ling-zeng Meng, IOZ(E)1903935; id., but, 22.15857°N, 100.66529°E, 709 m, 20.V.2008, IOZ(E)1903928; id., but 26.III.2009, Ling-zeng Meng, IOZ(E)1903928; id., but 18.VIII.2008, IOZ(E) 1903931; id., but 20.X.2008, IOZ(E)1903932; id., but 28.VI.2008, IOZ(E)1903933, IOZ(E)1903934; id., but 6.VI.2008, IOZ(E)1903937; id., but 15.VI.2008, IOZ(E)1903938; id., but 25–26.V.2008, Feng Liu, IOZ(E)1903939; id., but, 22.13091°N, 100.66861°E, 689m, 28.VI.2008, Ling-zeng Meng, IOZ(E)1903935; id., but 14.XII.2007, IOZ(E) 1903940”; 1 ♀ (IZCAS) “[China:] Yunnan, Hekou, Xiaonanxi, 200 m, 9.VI.1956, Ke-ren Huang, IOZ(E)1495286”; 1 ♀ (IZCAS) “[China:] Yunnan, Xishuangbanna, Damenglong, 7.IV.1958, Fu-ji Pu, IOZ(E)1495287”; 3 ♀ + 1 ♂ (IZCAS) “[China:] Yunnan, Jingping, Mengla, 420 m, 19–21. IV.1956, Ke-ren Huang, IOZ(E)1495291, IOZ(E)1495292; id., but 400 m, 28.IV.1956, IOZ(E)1495285; id., but 370 m, 12.IV.1956, IOZ(E)1495288”.

#### Diagnosis.

Supra-antennal elevations 0.1–0.4 times as long as scapus and outer side of elevations oblique (Fig. 419); head posteriorly with extensive yellowish or orange-brown pattern, including a V-shaped yellow or orange pattern behind stemmaticum (Fig. 419); head dorsally often densely reticulate-punctate (Fig. 419); clypeus moderately emarginate medio-ventrally (Fig. 418); middle mesoscutal lobe similar to lateral lobes, black or yellow laterally and black medially (Fig. 423); mesopleuron and metapleuron with extensive yellow pattern; third submarginal cell of fore wing 0.5–0.7 times as long as second submarginal cell (Fig. 421); basal cell often largely and posterior half of first submarginal cells of fore wing more or less darkened, similar to colour of marginal cell (Fig. 421); second sternite of both sexes without medio-apical protuberance (Fig. 427), distinctly convex and no opening between second and following sternites in lateral view (Fig. 426); third sternite of female without apical ledge (Fig. 427). *Taeniogonalos gestroi* is close to *Taeniogonalos formosana* (sharing the flattened and smooth metanotum medially, the oblique striae or rugae of the propodeum and the densely and finely rugose lateral lobes of the mesoscutum), but differs by the partly yellow mesopleuron and metapleuron (black in *Taeniogonalos formosana*; Figs 379, 390, 402), clypeus moderately emarginate medio-ventrally (shallowly emarginate), apex of hypopygium of ♀ yellowish brown (dark brown), second submarginal cell 0.5–0.7 times as long as third submarginal cell (0.7–0.8 times) and the gradually depressed second sternite medio-posteriorly (comparatively steeply depressed medio-posteriorly; Fig. 427).

**Figures 417–419. F81:**
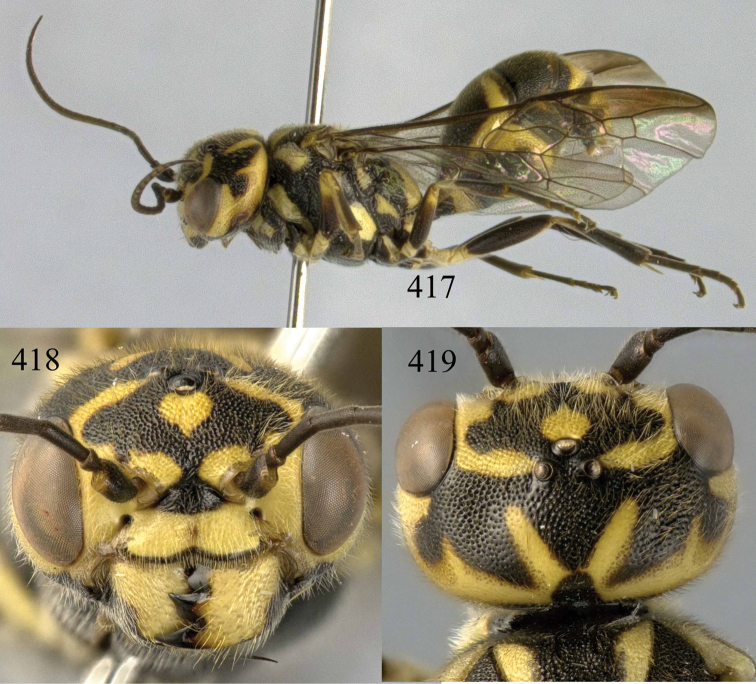
*Taeniogonalos gestroi* (Schulz, 1908), female from Yunnan. **417** Habitus lateral **418** head anterior **419** head dorsal.

**Figures 420–427. F82:**
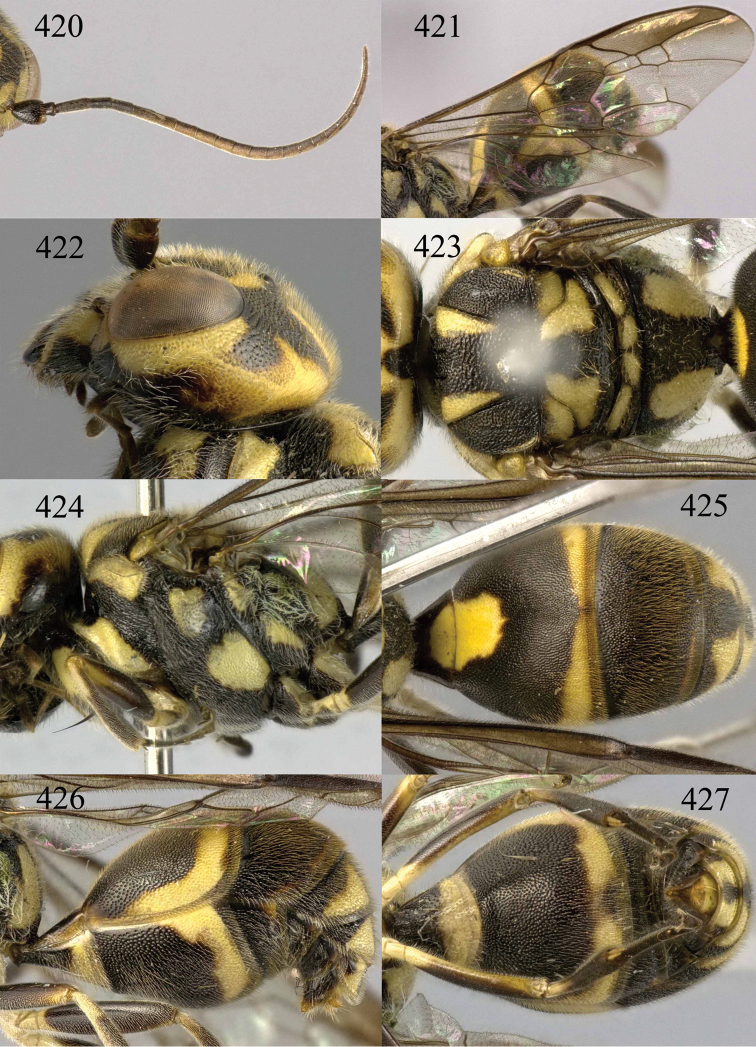
*Taeniogonalos gestroi* (Schulz, 1908), female from Yunnan. **420** Antenna **421** fore and hind wings **422** head lateral **423** mesosoma dorsal **424** mesosoma lateral **425** metasoma dorsal **426** metasoma lateral **427** metasoma ventral.

#### Description.

Redescribed after a female from Naban River Watershed, length of body 8.9 mm (of fore wing 7.2 mm).

*Head*. Antenna with 24 segments; frons reticulate-punctate (Fig. 418); vertex reticulate-punctate behind stemmaticum and near eyes, becoming spaced punctate (interspaces much wider than width of punctures) posteriorly (Fig. 419); temple largely smooth with sparse fine punctures (Fig. 422); head gradually narrowed behind eyes, eye in dorsal view 0.8 times as long as temple (Fig. 419); occipital carina narrow, non-lamelliform and smooth medio-dorsally (Fig. 419); supra-antennal elevations medium-sized (about 0.2 times as long as scapus) and their outer side oblique (Fig. 419); clypeus moderately concave and thick medio-ventrally.

*Mesosoma*. Length of mesosoma 1.2 times its height (Fig. 424); mesopleuron densely and coarsely reticulate-rugose anteriorly, densely and finely punctate posteriorly; transverse mesopleural groove narrow, shallow and weakly crenulate; notauli narrow, deep and weakly crenulate; middle lobe of mesoscutum reticulate-rugose anteriorly, transversely rugose posteriorly, lateral lobes reticulate-rugose (Fig. 423); scutellar sulcus narrow but complete, weakly crenulate; scutellum densely and finely reticulate-rugose, rather flat and anteriorly near level of level of mesoscutum; metanotum medially slightly convex, not protruding and finely rugose (Fig. 423); propodeum densely and coarsely reticulate-rugose (Fig. 423); posterior propodeal carina thick lamelliform and strongly arched, foramen medially 0.5 times higher than wide basally.

*Wings*. Fore wing: length of vein 1-M 1.4 times as long as vein 1-SR (Fig. 421).

*Metasoma*. First tergite 0.6 times as long as apically wide, smooth and with shallow elliptical depression medially (Fig. 425); second–sixth tergites densely reticulate-punctate; sternites densely punctate; second sternite distinctly convex in lateral view, without medio-apical protuberance (Fig. 427); third sternite about 0.2 times as long as second sternite (Fig. 427); hypopygium triangular in ventral view (Fig. 427).

*Colour*. Yellow with black pattern; frons largely yellow, area above and between supra-antennal elevations largely black but with a heart-shaped yellow spot below anterior ocellus, black marks medio-ventrally extending to base of clypeus; clypeus yellow with ventral margin dark brown; mandible yellow with teeth and latero-ventral margin black; vertex largely black with a V-shaped yellow patch posteriorly and postero-lateral area above occipital carina yellow; upper temple yellow and lower margin black; mesosoma laterally black with following yellow markings: broad oblique stripe on propleuron, stripe on upper pronotum and lower pronotum, broad oblique but medially interrupted stripe on mesopleuron and metapleuron; mesosoma dorsally black with following yellow markings: antero-lateral stripes on middle lobe of mesoscutum, axilla, antero-lateral half, metanotum, broad longitudinal lateral stripes on propodeum; metasoma black with following yellow markings: broad patch on posterior margin of first tergite and anterior margin of second tergite, narrow continuous stripe on posterior an lateral margin of second tergite, postero-lateral broad patches on fourth to sixth tergites, posterior half of first sternite, rather broad stripe on posterior margin of second sternite; palpi dark brown; antenna dark brown with first five segments darker; coxae dorsally, trochanters and trochantelli, base and apex of femora and base of tarsi yellow, remainder of legs dark brown to black; pterostigma dark brown, marginal cell more or less dark brown, remainder of wing membrane subhyaline.

*Variation*. Length of body 5.5–9.3 mm, of fore wing 4.2–8.1 mm; length of vein 1-M of fore wing 1.2–1.4 times as long as vein 1-SR.

*Male*. Length of body 7.4–9.1 mm, of fore wing 5.8–6.9 mm; antenna with 24 segments, tyloids linear, 0.8 times as long as segment on 10^th^–16^th^ segments and 0.5 times as long as segment on 17^th^ segment; genitalia internal.

#### Biology.

Hyperparasitoid of Ichneumonidae in Pyralid caterpillars (Carmean and Kimsey 1998; as *Taeniogonalos thwaitesii*).

#### Distribution.

China (Jiangsu, Taiwan, Hainan, Yunnan); India (Sikkim); Sri Lanka; Thailand (Thaleban N.P., 200 m); Laos; Papua New Guinea; Myanmar (syntype); Malaysia; Indonesia (Sumatra, syntype).

#### Notes.

As pointed out by Schulz (1908) specimens from Indonesia and Myanmar differ from both examined female syntypes of *Taeniogonalos thwaitesii* (Westwood, 1874) from Sri Lanka. He created a new name for the specimens from Indonesia and Myanmar: *Poecilogonalos pulchella* var. *gestroi*. After examination of a type of *Taeniogonalos thwaitesii*, Dr D. Smith (in litt.) kindly informed one of us (CvA) that *Taeniogonalos thwaitesii* (auctt.) from North India up to China is indeed a species different from the typical *Taeniogonalos thwaitesii* from Sri Lanka and possibly South India. Therefore, we use the first available name for this species, *Taeniogonalos gestroi* (Schulz, 1908) which is a new combination.

### Taeniogonalos maga

(Teranishi, 1929)

http://species-id.net/wiki/Taeniogonalos_maga

**Figures 428–430. F83:**
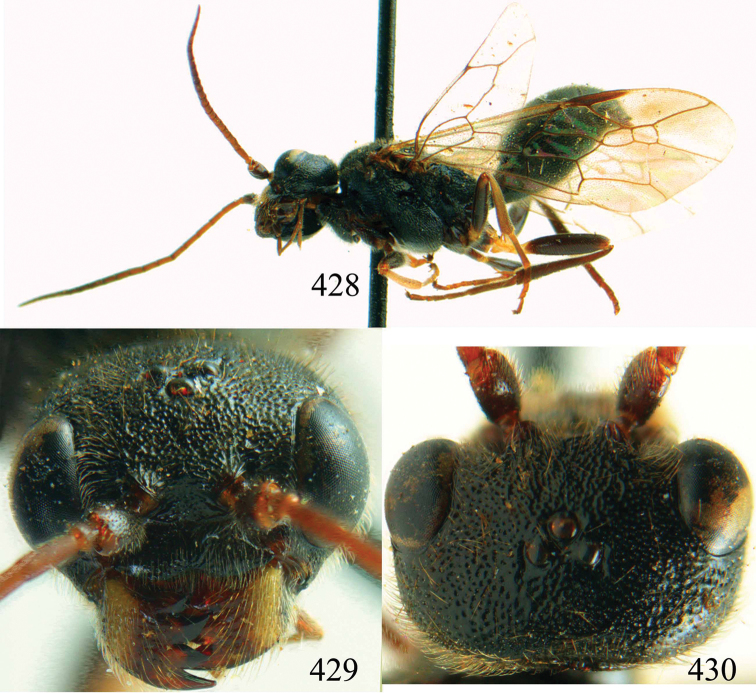
*Taeniogonalos maga* (Teranishi, 1929), holotype, male. **428** Habitus lateral **429** head anterior **430** head dorsal.

Figs 428 [Fig F84] [Fig F85] –448

Poecilogonalos maga Teranishi, 1929: 148; Marshakov 1981: 106; Tsuneki 1991: 51; Weinstein and Austin 1991: 423; Lelej 1995: 14.
Taeniogonalos maga ; Carmean and Kimsey 1998: 68; Bennett and Lelej 2003: 8; Li et al. 2012: 4.
Taiwanogonalos alishana Tsuneki, 1991: 36. Synonymized by Carmean and Kimsey 1998 with *Taeniogonalos maga*.
Taiwanogonalos claripennis Tsuneki, 1991: 38. Synonymized by Carmean and Kimsey 1998 with *Taeniogonalos maga*.

#### Type material.

Holotype of *Poecilogonalos maga*, ♂ (OMNH), “[Japan:], Nagano, Shimajima, VII.1927, K. Sato”, “Holotype *Poecilogonalos maga*”; 1 paratype, ♀ (OMNH), topotypic and same date, “Allotype *Poecilogonalos magae*”. Holotype of *Taeniogonalos alishana*, ♂ (OMNH), “[China:], Taiwan, Arisan, 27.V.1929, K. Sato”, “Holotype *P. maga arisana*” [MS label by C. Teranishi], “*Taiwanogonalos alishana* Tsuneki, ♂, holotype”.

#### Additional material.

1 ♀ (ZJUH) “[China:] Zhejiang, Mt. Tianmu, 18.VI.1983, Yun Ma, 831378”; 1 ♀ + 7 ♂ (ZJUH, RMNH) “[China:] Zhejiang, Mt. Tianmu, Xianrending, 1509 m, 14.VII.1998, MT, Ming-shui Zhao, 200010683, 200010684, 200010687; id., but 20.VII.1998, 992748, 992753; id., but 23.VII.1998, 200010697; id., but 28.VII.2003, Xiao-xia Yu, 20034472; id., but 2–4.VI.1990, Zu-hua Shi, 901559; id., but Yong-gen Lou, 900463”.

#### Diagnosis.

Outer side of supra-antennal elevations oblique and elevations 0.4–0.5 times as long as scapus (Fig. 430); occipital carina moderately widened medio-dorsally (near weak medio-posterior depression of vertex up to 0.5 times as wide as diameter of ocellus; Fig. 430); anteriorly vertex of ♀ often partly coarsely punctate and smooth interspaces partly narrower than diameter of punctures; head posteriorly entirely black (Fig. 430); mesosoma of both sexes black dorsally and coarsely sculptured (Fig. 434); marginal cell of fore wing with brown patch (Fig. 432); second sternite of female distinctly convex, of male with shallow depressed subposterior area (Fig. 438); third sternite of male slightly convex or flat subposteriorly; third sternite 0.2-0.3 times as long as second sternite.

**Figures 431–438. F84:**
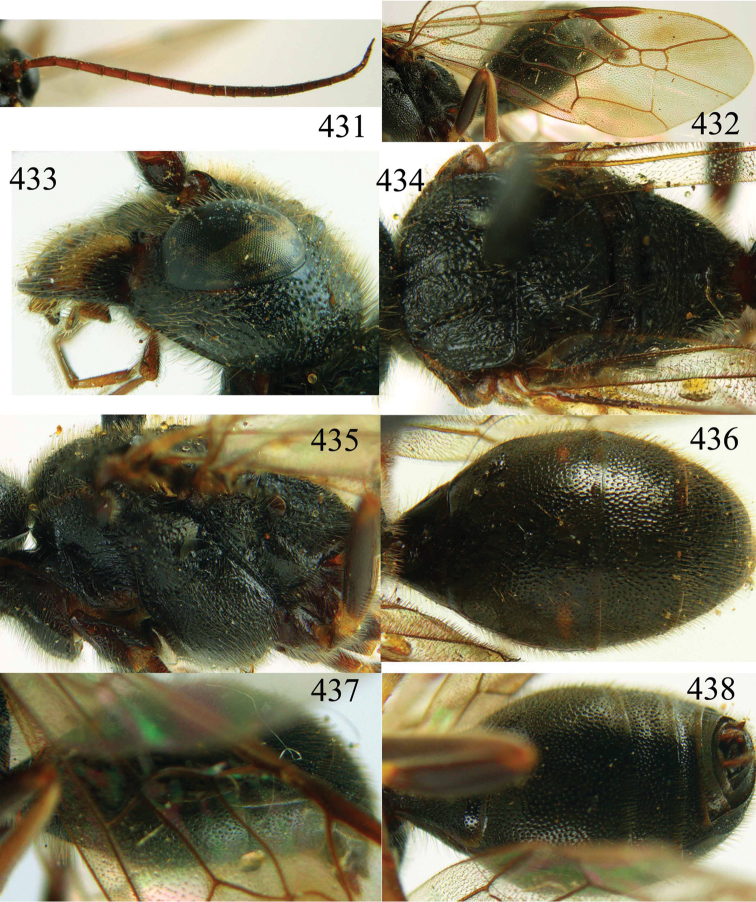
*Taeniogonalos maga* (Teranishi, 1929), holotype, male. **431** Antenna **432** fore wing **433** head lateral **434** mesosoma dorsal **435** mesosoma lateral **436** metasoma dorsal **437** metasoma lateral **438** metasoma ventral.

**Figures 439–442. F85:**
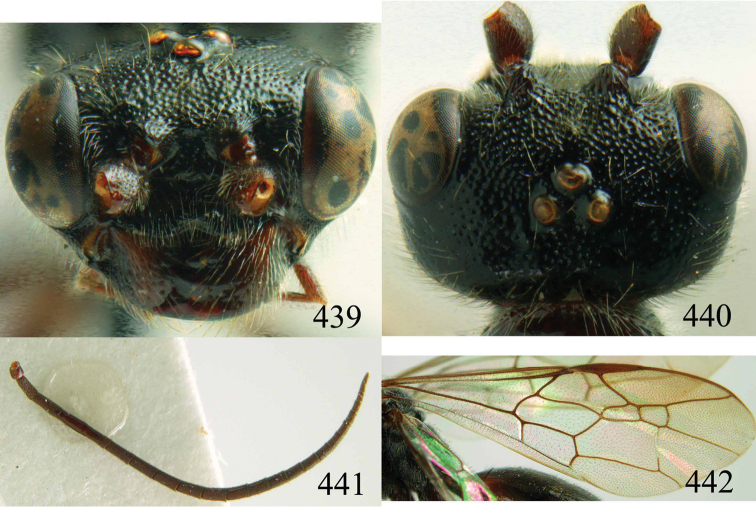
*Taeniogonalos alishana* (Tsuneki, 1991), holotype, male. **439** Head anterior **440** head dorsal **441** antenna **442** fore wing.

**Figures 443–448. F86:**
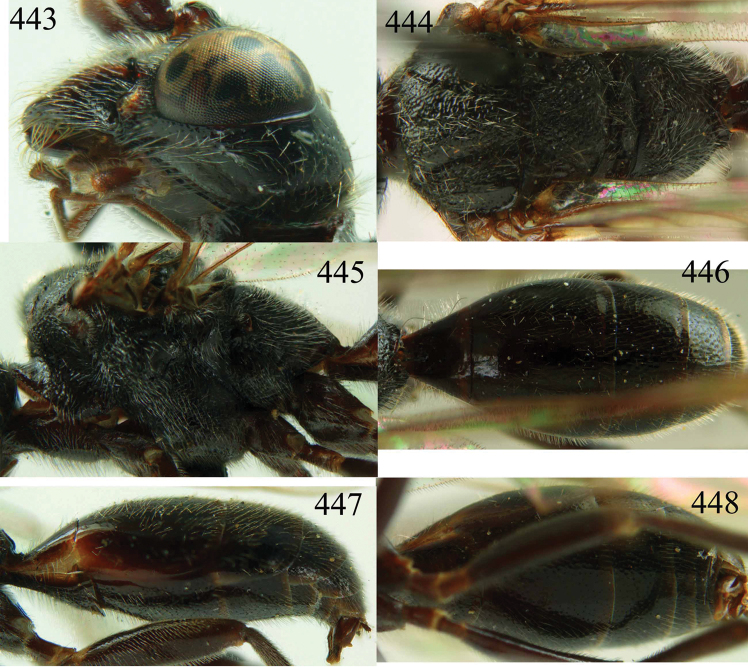
*Taeniogonalos alishana* (Tsuneki, 1991), holotype, male. **443** Head lateral **444** mesosoma dorsal **445** mesosoma lateral **446** metasoma dorsal **447** metasoma lateral **448** metasoma ventral.

#### Description.

Holotype, male, length of body 8.7 mm (of fore wing 7.8 mm).

*Head*. Antenna with 23 segments with longitudinal tyloids on 10^th^-14^th^ segments; frons densely coarsely punctate (interspaces less than width of punctures) and with some rugae anteriorly; vertex coarsely punctate (Fig. 430), with medium-sized setae; temple spaced punctate (Fig. 433); head gradually narrowed behind eyes, eye in dorsal view as long as temple (Fig. 430); occipital carina moderately widened medio-dorsally (near weak medio-posterior depression of vertex up to 0.5 times as wide as diameter of ocellus; Fig. 430); with few weak crenulae dorsally; supra-antennal elevations medium-sized (about 0.5 times as long as scapus) and their outer side rather steeply oblique (Fig. 430); clypeus weakly concave and thick medio-ventrally.

*Mesosoma*. Length of mesosoma 1.4 times its height (Fig. 435); transverse mesopleural groove moderately wide, deep and coarsely crenulate; mesopleuron reticulate-rugose (antero-dorsally more coarsely so), but speculum smooth and shiny; notauli moderately narrow, deep and sparsely crenulate; middle lobe of mesoscutum coarsely vermiculate rugose, lateral lobes more spaced rugose (Fig. 434); scutellar sulcus complete, medium-sized and crenulate medially and wider laterally; scutellum densely rugose, rather flat anteriorly and slightly above level of mesoscutum, but rather longitudinally depressed medially; metanotum medially distinctly convex, moderately protruding and rugose (Fig. 434); propodeum entirely vermiculate-rugose or nearly so (Fig. 434); posterior propodeal carina rather wide lamelliform and distinctly arched, foramen about 1.3 times as wide basally as high medially.

*Wings*. Fore wing: length of vein 1-M 1.6 times as long as vein 1-SR (Fig. 432).

*Metasoma*. First tergite 0.6 times as long as apically wide, largely smooth and with distinct wide depression medially (Fig. 436); second and following tergites densely superficially punctate and shiny (Fig. 436); sternites rather coarsely and densely punctate; second sternite weakly convex in lateral view (Fig. 437); third sternite 0.3 times as long as second sternite; second sternite slightly depressed medio-posteriorly (Fig. 438); third sternite flat medio-posteriorly.

*Colour*. Black; mandible medially, apices of fore and middle trochanters, hind trochanter and trochantellus and bases of femora largely ivory; second tergite with indistinct pair of small brownish patches posteriorly (Fig. 436); antenna (but darkened basally), palpi and tegulae mainly brown; fore tibia and tarsus, middle tibia basally brownish-yellow and remainder of legs dark brown; subapical patch of fore wing infuscate; pterostigma and veins brown, remainder of wing membrane subhyaline.

*Variation*. Length of body 7.0–8.7 mm, of fore wing 6.8–7.8 mm; antenna of ♂ with 22 (1), 23 (1) or 24 (1) segments, with linear tyloids on (9^th^–)10^th^–14^th^(–15^th^) segments; second tergite black, ivory or yellowish latero-posteriorly, propodeum coarsely to superficially sculptured.

*Female*. Female paratype is very similar to holotype, but brown patch of fore wing larger and darker, first metasomal tergite with pair of ivory patches apically, and second tergite with pair of large ivory patches latero-posteriorly; length of body 9.0 mm and of fore wing 8.8 mm.

#### Biology.

Unknown (record for *Taeniogonalos maga* concerns *Taeniogonalos taihorina*). Collected in May–July, up to 1509 m.

#### Distribution.

China (Taiwan, Zhejiang); Japan (Honshu). Other records of this species need reconfirmation.

### Taeniogonalos mongolica

(Popov, 1945)

http://species-id.net/wiki/Taeniogonalos_mongolica

Figs 449–451

Nanogonalos mongolicus Popov, 1945: 76; Marshakov 1981: 107 (lectotype designation); Weinstein and Austin 1991: 421.
Taeniogonalos mongolica ; Lelej 2003: 6.

#### Type material.

Lectotype, ♂ (ZISP) from Mongolia.

#### Additional material.

No material available for this study; reported from China by Lelej (2003).

#### Diagnosis.

**Figures 449–451. F87:**
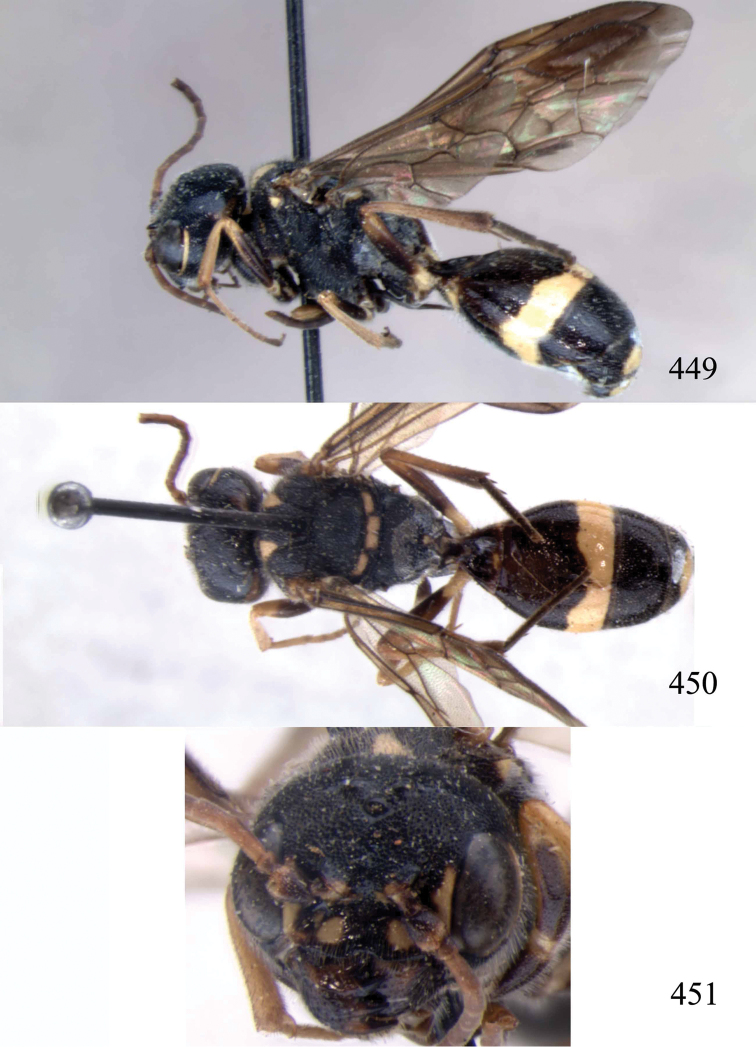
*Taeniogonalos mongolicus* (Popov, 1945), lectotype, male. **449** Habitus lateral **450** habitus dorsal **451** head anterior.

Supra-antennal elevations 0.1–0.4 times as long as scapus and outer side of elevations oblique (Fig. 451); occipital carina lamelliform, wide; head black, but supra-antennal elevation anteriorly, two patches of clypeus, inner and (narrowly) outer orbita yellow; metanotum with lateral yellow patch; entire fore wing dark brown (Fig. 449); second metasomal sternite of female simple, only slightly convex (Fig. 449); second and third sternites of male with flattened and shiny posterior patch; second tergite and sternite with wide yellow band apically, and remainder of metasoma largely black (Fig. 450); length of body 14.5 mm.

#### Description

(translation of short redescription in Russian by Marshakov (1981); only males known). Male. Antenna with 23–25 segments, 10^th^–16^th^ segments with tyloids; two spots on clypeus, a narrow band on inner and outer orbita of eyes, two spots on mesoscutum, [most of] postscutellum, band on first [mainly ventrally] and second [metasomal] segments, anterior and middle tarsi and tibiae yellow; [remainder of] legs light brown, antenna rusty yellow to light brown. Specimens from Amurskoj oblast have the scutellum marked with yellow, the wings are very slightly darkened and the background colour of the first and second [metasomal] segments is black (with brown-red hue in specimens from Mongolia); the head, the mesoscutum and the propodeum dorsally have large spots; the mesopleuron is finely densely punctate, with almost no interspaces between the punctures, matt; the frontal keels are like a “7”. The length of the body is 9-11 mm.

#### Biology.

Unknown.

#### Distribution.

China (Inner Mongolia); Russia; Mongolia; Korea (Lelej 2003).

#### Notes.

*Taeniogonalos flavocincta* (Teranishi, 1929) was synonymized with *Taeniogonalos mongolica* by Marshakov (1981), but the holotype of *Taeniogonalos flavocincta* is not synonym with *Taeniogonalos mongolica*. The male holotype of *Taeniogonalos flavocincta* differs as follows: the frons, vertex and mesoscutum coarsely reticulate-punctate (moderately spaced punctate with distinct smooth interspaces in *Taeniogonalos mongolica*; Fig. 451), the clypeus black (with pair of ivory patches; Fig. 451), the metanotum with only a medial ivory patch (with medial and pair of lateral patches; Fig. 450), the mesoscutum black anteriorly (with pair of ivory patches; Fig. 450), the second and third tergites densely and coarsely punctate, rather matt (largely smooth and strongly shiny; Fig. 450) and the posterior half of the fore wing subhyaline (infuscate; Fig. 449). From the translated redescription of Marshakov above it is clear that his series is mixed and consists of several species; the presence of *Taeniogonalos mongolica* in Inner Mongolia needs reconfirmation.

### Taeniogonalos rufofasciata

(Chen, 1949)

http://species-id.net/wiki/Taeniogonalos_rufofasciata

Figs 452 –462

Poecilogonalos rufofasciata Chen, 1949: 15; Weinstein and Austin 1991: 423.
Taeniogonalos rufofasciata ; Carmean and Kimsey 1998: 68.

#### Type material.

Lectotype of *Poecilogonalos rufofasciata* here designated, ♀ (IZCAS) “[China: Jiangxi,] Ku-ling. Musée Heude”, 3.VIII.[19]35. O. Piel”, “*Poecilogonalos rufofasciata* n. sp.”. Paralectotypes of *Poecilogonalos rufofasciata*: 3 ♀ (IZCAS), topotypic, but 7.VII.1935, 9.VII.1935 and 10.VIII.1935.

#### Additional material.

1 ♀ (ZJUH) “[China:] Fujian, Mt. Xianfengling, 13.VII.1963, Jia-hua Chen, SCAU 311”.

#### Diagnosis.

Supra-antennal elevations 0.1–0.4 times as long as scapus and outer side of elevations oblique and art least partly yellow (Fig. 454); occipital carina comparatively wide medio-dorsally (Fig. 454); head posteriorly with extensive yellowish or orange-brown pattern, including a V-shaped yellow or orange pattern behind stemmaticum (Fig. 454); head dorsally often densely reticulate-punctate (Fig. 454); temple dorsally coarsely reticulate-punctate (Fig. 457); mesopleuron largely black, without pale pattern (Fig. 459), at most with orange patch medio-dorsally; middle lobe of mesoscutum coarsely reticulate-punctate (Fig. 458); scutellum black medio-anteriorly and medially (Fig. 458); anterior half of fore wing more or less brown, with a less isolated patch (Fig. 456); protuberance of second metasomal sternite truncate, not protruding behind apical margin of sternite and posteriorly concave (Figs 461, 462), in female resulting in circular or rectangular opening between second and following sternites in lateral view (Fig. 461); third sternite of female subtruncate apically in lateral view (Fig. 461); third–fifth tergites with continuous yellow band posteriorly; sixth tergite largely yellow (Fig. 462).

**Figures 452–454. F88:**
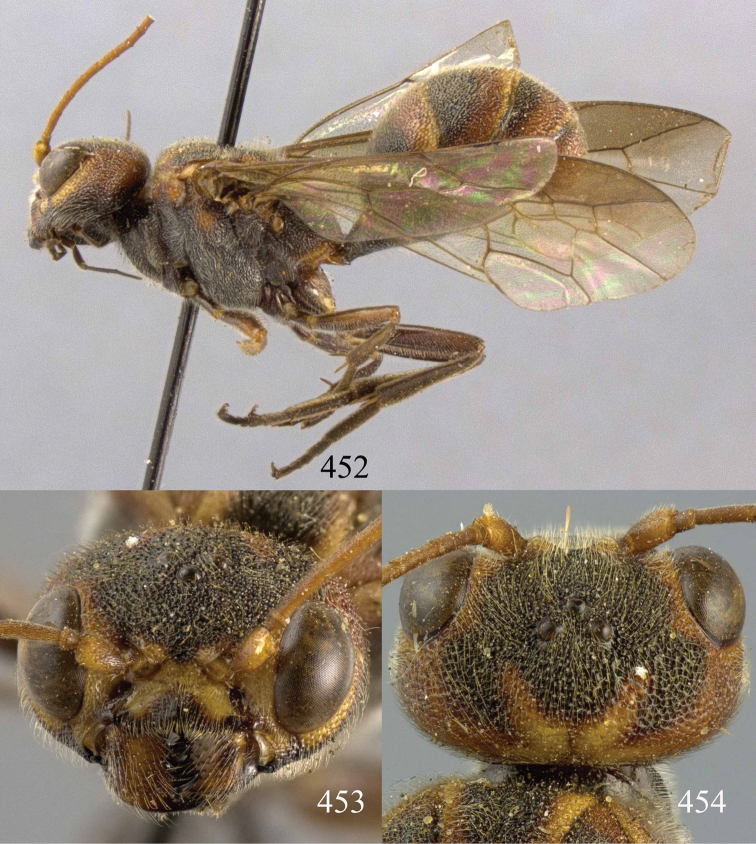
*Taeniogonalos rufofasciata* (Chen, 1949), lectotype, female. **452** Habitus lateral **453** head anterior **454** head dorsal.

**Figures 455–462. F89:**
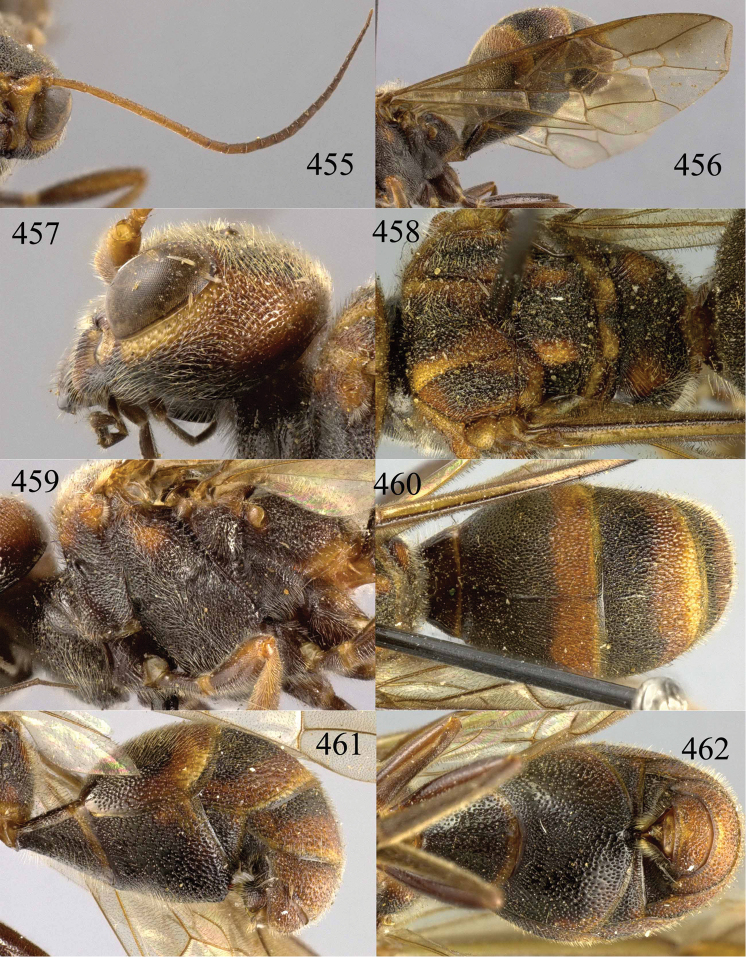
*Taeniogonalos rufofasciata* (Chen, 1949), lectotype, female. **455** Antenna **456** fore and hind wings **457** head lateral **458** mesosoma dorsal **459** mesosoma lateral **460** metasoma dorsal **461** metasoma lateral **462** metasoma ventral.

#### Description.

Redescribed after the female from Fujian, length of body 12.7 mm (of fore wing 10.3 mm).

*Head*. Antenna with 26 segments; frons, vertex and temple coarsely reticulate-punctate (Figs 453, 454, 457); head gradually narrowed behind eyes, eye in dorsal view 0.8 times as long as temple (Fig. 454); occipital carina weakly developed medially, narrowly lamelliform laterally; supra-antennal elevations medium-sized (about 0.1 times as long as scapus), outer side oblique and rugulose; clypeus slightly concave and thick medio-ventrally.

*Mesosoma*. Length of mesosoma 1.2 times its height (Fig. 459); mesopleuron coarsely reticulate-rugose; transverse mesopleural groove narrow, shallow and weakly crenulate; notauli moderately narrow, deep and coarsely crenulate; middle lobe of mesoscutum coarsely reticulate-punctate, lateral lobes somewhat longitudinally rugose (Fig. 458); scutellar sulcus complete, medium-sized and crenulate medially and wider laterally; scutellum densely and coarsely reticulate-punctate, convex anteriorly and near level of mesoscutum; metanotum medially slightly convex, not protruding and rugose (Fig. 458); propodeum obliquely striate antero-laterally, irregularly rugose medially and posteriorly (Fig. 458); posterior propodeal carina thick lamelliform and slightly arched, foramen medially 0.6 times higher than wide basally.

*Wings*. Fore wing: length of vein 1-M 1.2 times as long as vein 1-SR (Fig. 456).

*Metasoma*. First tergite 0.4 times as long as apically wide, smooth and with shallow elliptical depression medially (Fig. 460); second–sixth tergites densely and coarsely reticulate-punctate (Fig. 460); sternites densely and coarsely punctate; second sternite distinctly convex, medio-apical protuberance truncate, not protruding behind apical margin of sternite and posteriorly concave (Fig. 462); third sternite about 0.1 times as long as second sternite and subtruncate apically in lateral view (Fig. 461); hypopygium triangular in ventral view.

*Colour*. Head largely dark brown with following orange-brown markings: stripes along inner and outer orbita (including malar space), lateral area of clypeus and area above clypeus, V-shaped patch behind stemmaticum; mesosoma largely dark brown to black with following yellowish brown makings: antero-lateral stripes on middle lobe of mesoscutum, tegulae, posterior half of axillae, submedial spot on scutellum, submedian and lateral spots on metanotum, antero-dorsal spot on lateral pronotum side, dorsal spot on mesopleuron, rather large lateral spots on propodeum; metasoma largely black with following orange-brown or yellowish brown markings: narrow stripe on posterior margin of first tergite and sternite, broad stripe on posterior margin of second and third tergites, posterior 0.7 of fourth and almost entirely fifth and sixth tergites, postero-lateral spots on second sternite; mandible dark brown; palpi dark brown; basal half of antenna (including scapus) yellowish brown, otherwise dark brown; legs dark brown to nearly black; pterostigma and anterior half of fore wing dark brown, remainder of wing membrane subhyaline.

*Variation*. Length of body 7.8–12.7 mm, of fore wing 6.4–10.3 mm; antenna of ♀ with 24–27 segments; orange-brown markings on body paler or darker; length of vein 1-M of fore wing 1.0–1.2 times as long as vein 1-SR.

*Male*. Unknown.

#### Biology.

Unknown. Collected in July and August.

#### Distribution.

China (Fujian, Jiangxi).

### Taeniogonalos sauteri

Bischoff, 1913

http://species-id.net/wiki/Taeniogonalos_sauteri

**Figures 463–467. F90:**
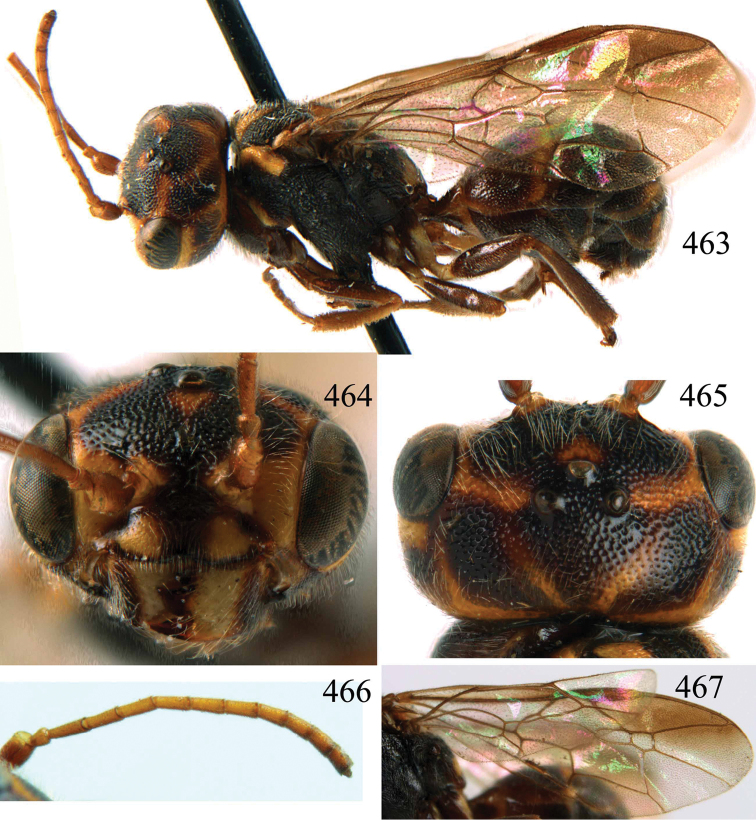
*Taeniogonalos sauteri* Bischoff, 1913, holotype, female. **463** Habitus lateral **464** head anterior **465** head dorsal **466** antenna **467** fore and hind wings.

Figs 463 [Fig F91] [Fig F92] –484

Taeniogonalos sauteri Bischoff, 1913: 153; Weinstein and Austin 1991: 416; Tsuneki 1991: 59; Carmean and Kimsey 1998: 68; He 2004: 76.
Taeniogonalos pictipennis Strand, 1914: 32; Weinstein and Austin 1991: 416; Tsuneki 1991: 59. Synonymized by Tsuneki 1991.

#### Type material.

Holotype of *Taeniogonalos sauteri*, ♀ (ZMB), “[China:] Formosa, Hoozan, I.[19]10, H. Sauter S.G.”, “*Taeniogonalos sauteri* m.*, det. Bischoff”, “Type”. Holotype of *Taeniogonalos pictipennis*, ♀ (SDEI) “[China: Taiwan,] Sokutsu, Banshoryo Distr., H. Sauter, 1912, “7.VII”, “Holotypus”, “Type”. “*Taeniogonalos pictipennis* m., ♀, Strand det.”.

#### Additional material.

2 ♀ (ZJUH) “[China:] Fujian, Jianyang, X.1985, Fu-ming Liu, 854116, 854117”; 1 ♀ (ZJUH) “[China:] Guangxi, Longsheng, Neichu River, 24.VI.1982, Jun-hua He, 823444”; 1 ♀ (ZJUH), “[China:] Zhejiang, Songyang, IX.1982, Han-lin Chen, reared from *Phanerotoma flava* (Braconidae), parasitoid of *Locastra muscosalis*, det. He”.

#### Diagnosis.

Supra-antennal elevations 0.1–0.4 times as long as scapus and outer side of elevations oblique and art least partly yellow (Figs 465, 476); occipital carina comparatively wide medio-dorsally (Fig. 465); head posteriorly with extensive yellowish or orange-brown pattern, including a V-shaped yellow or orange pattern behind stemmaticum (Fig. 465); head dorsally often densely reticulate-punctate (Fig. 465); temple dorsally largely smooth except for some punctures; mesopleuron largely black, without pale pattern (Fig. 470), at most with orange patch medio-dorsally; middle lobe of mesoscutum moderately to finely reticulate-punctate (Fig. 469); scutellum medio-anteriorly yellowish or orange-brown (Fig. 469), black medially; anterior half of fore wing more or less brown, with a less isolated patch (Fig. 467); protuberance of second metasomal sternite triangularly protruding behind apical margin of sternite and posteriorly truncate (Figs 472, 473); in female resulting in circular or rectangular opening between second and following sternites in lateral view (Fig. 472); third sternite of female subtruncate apically in lateral view (Fig. 472); third–fifth tergites with continuous yellow band posteriorly; sixth tergite largely yellow (Fig. 473).

**Figures 468–473. F91:**
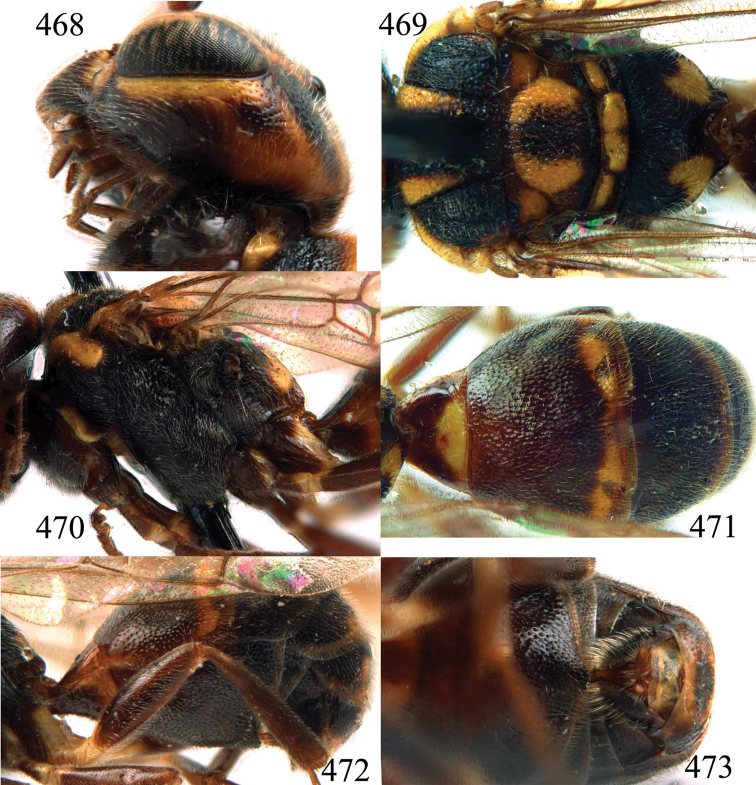
*Taeniogonalos sauteri* Bischoff, 1913, holotype, female. **468** Head lateral **469** mesosoma dorsal **470** mesosoma lateral **471** metasoma dorsal **472** metasoma lateral **473** metasoma ventral.

**Figures 474–477. F92:**
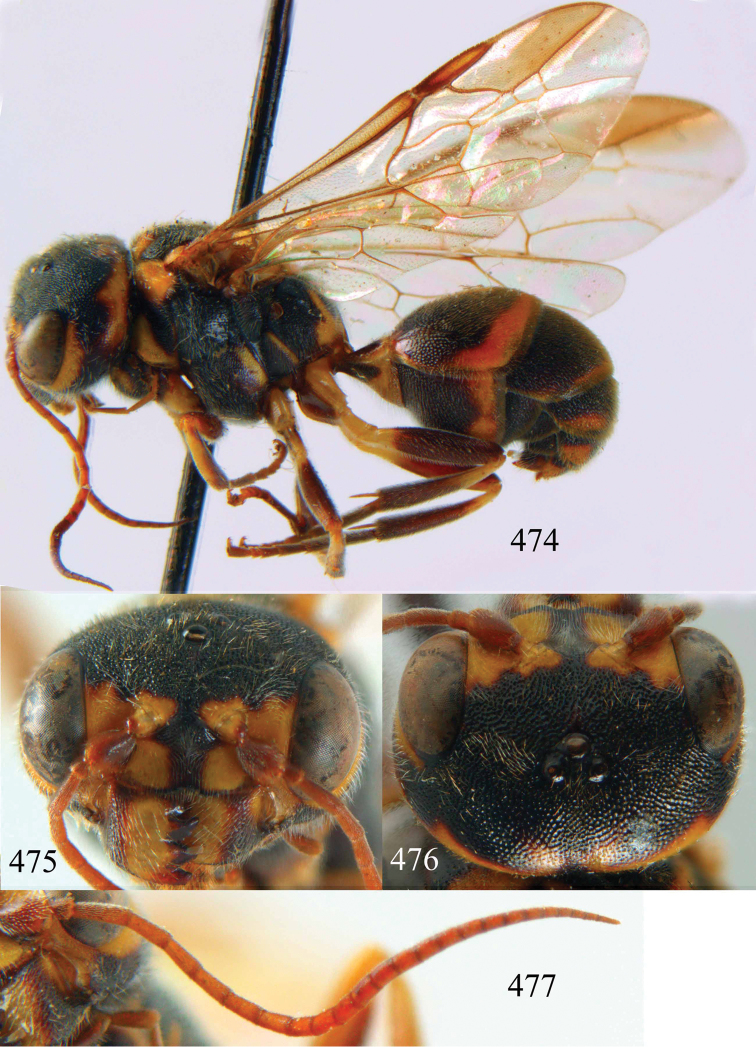
*Taeniogonalos pictipennis* Strand, 1914, holotype, female. **474** Habitus lateral **475** head anterior **476** head dorsal **477** antenna.

**Figures 478–484. F93:**
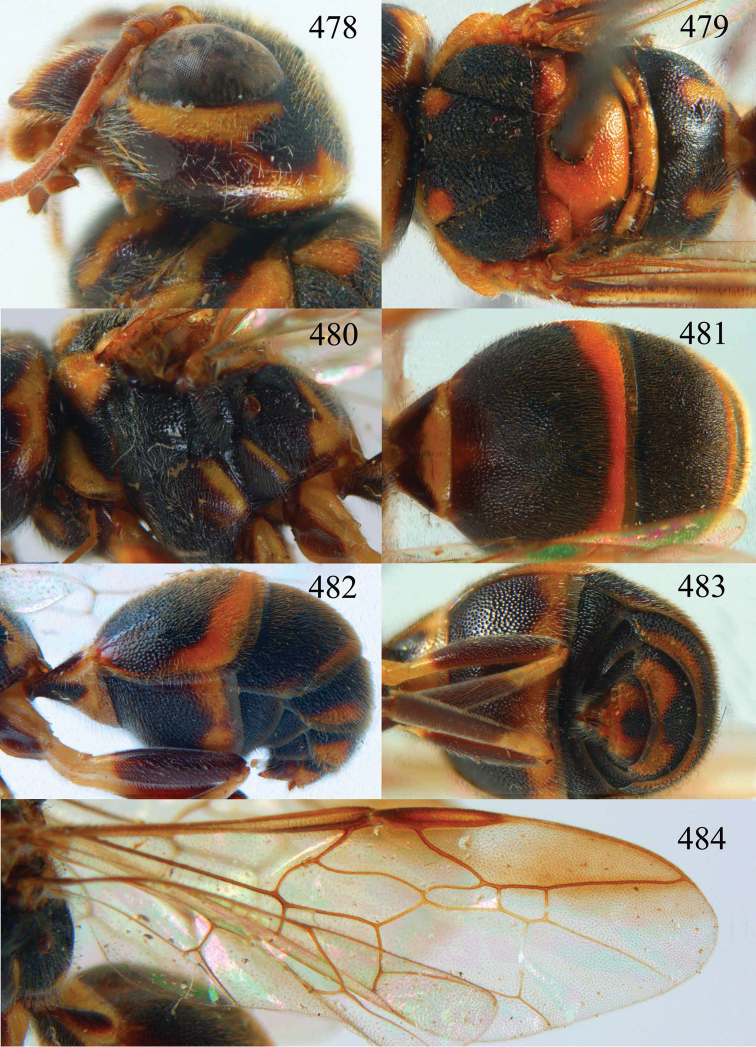
*Taeniogonalos pictipennis* Strand, 1914, holotype, female. **478** Head lateral **479** mesosoma dorsal **480** mesosoma lateral **481** metasoma dorsal **482** metasoma lateral **483** metasoma ventral **484** fore and hind wings.

#### Description.

Holotype of *Taeniogonalos sauteri*, female, length of body 5.5 mm (of fore wing 4.7 mm).

*Head*. Antenna incomplete (of female from Fujian with 25 segments); frons densely and rather coarsely reticulate-punctate; vertex densely and rather coarsely reticulate-punctate, but smooth posteriorly (Fig. 465); temple rather sparsely punctate dorsally and anteriorly, and remainder smooth (Fig. 468); head gradually narrowed behind eyes, eye in dorsal view 0.8 times as long as temple (Fig. 465); occipital carina narrow, hardly lamelliform and smooth medio-dorsally; supra-antennal elevations medium-sized (about 0.5 times as long as scapus), outer side oblique, lamelliform and largely smooth; clypeus weakly concave and thick medio-ventrally.

*Mesosoma*. Length of mesosoma 1.3 times its height (Fig. 470); mesopleuron densely obliquely rugulose, but irregularly rugulose dorsally and mainly smooth posteriorly (Fig. 470); transverse mesopleural groove medium-sized and distinctly crenulate; notauli narrow and smooth; middle lobe of mesoscutum transversely costate, lateral lobes densely punctate-rugose (Fig. 469); scutellar sulcus obsolescent medially, narrow and narrowly crenulate laterally; scutellum coarsely reticulate-rugose, but posteriorly largely smooth, largely flat and anteriorly near level of mesoscutum; metanotum medially nearly flat, shiny and largely smooth (Fig. 469); propodeum with curved striae, largely smooth medially (Fig. 469); posterior propodeal carina weakly developed, non-lamelliform and strongly arched, foramen medially 0.8 times higher than wide basally.

*Wings*. Fore wing: length of vein 1-M 1.6 times as long as vein 1-SR (Fig. 467); second submarginal cell 1.7 times as long as third cell.

*Metasoma*. First tergite 0.4 times as long as apically wide, smooth and with distinct elliptical depression medially (Fig. 471); second–sixth tergites densely and rather finely reticulate-punctate but exposed anterior rim of tergites superficially coriaceous; second sternite rather densely and coarsely reticulate-punctate with distinct smooth interspaces, other sternites largely smooth; second sternite strongly convex, medio-apical protuberance triangularly protruding behind apical margin of sternite in lateral view, apically triangular in posterior view and truncate in ventral view (Figs 472, 473); third sternite about 0.1 times as long as second sternite (Fig. 473); hypopygium triangular in ventral view (Fig. 473).

*Colour*. Head tricoloured, frons below supra-antennal elevations largely yellow except for interantennal area and middle of clypeus dark brown, above elevations blackish brown; with orange-brown patch in front of anterior ocellus and transverse stripes beside lateral ocelli and pair of oblique stripes branching off mainly orange-brown posterior part of head (Fig. 465); orbita largely yellow, but inner orbita with wide triangularly yellow patch ventrally; clypeus yellow but medially and ventral margin dark brown; mandible yellow with teeth and largely basally dark brown; mesosoma largely black but pronotal side dorsally and ventrally, middle lobe of mesoscutum laterally, axilla largely, large lateral spots on metanotum and propodeum yellow, antero-lateral 0.6 of scutellum and metanotum largely, pair of large patches on propodeum and tegulae yellow; metasoma blackish brown but first tergite medio-posteriorly, posterior band of first sternite and of second and third tergites, pair of postero-lateral patches on fourth–seventh tergites yellow; posterior margin of second sternite pale yellowish; palpi and antenna brown; coxae dorsally partly, trochanters dorsally and apically, trochantelli, base of femora narrowly and tibiae subbasally ivory, remainder of legs dark brown; pterostigma and marginal cell of fore wing and area below cell dark brown, remainder of wing membrane largely subhyaline.

*Variation*. Length of body 5.5–7.6 mm, of fore wing 4.7–6.5 mm; length of vein 1-M of fore wing 1.2–1.6 times as long as vein 1-SR; upper frons largely black with small triangular yellow patch below anterior ocellus; clypeus weakly concave medio-apically, often nearly straight; area besides stemmaticum yellow or orange or dark brown; propodeum with reversed comma-shaped lateral patch or oval patch; scutellum entirely orange-brown or with brown or black patch or all black; metasoma very variable in colour (see figures in Tsuneki 1991), may be darker than holotype, bands and patches on tergites of variable size, second–sixth sternites entirely black, with small yellowish brown postero-lateral patch on third sternite or fourth–sixth tergites of ♀ yellow, with medial dark brown patch or only anteriorly dark brown; basal half of second tergite sometimes medially yellow or blackish; mesopleuron black or with two fine yellow stripes. The holotype of *Taeniogonalos pictipennis* has the scutellum entirely yellow, the mesopleuron and the metapleuron with an elongate yellow patch postero-ventrally, the scutellar sulcus smooth and narrow, the vertex behind the posterior ocelli densely sculptured (without smooth interspaces between punctures) and the second sternite densely punctate. The differences seem to be intraspecific and, therefore, we accept the synonymy of this species with *Taeniogonalos sauteri*.

*Male*. Unknown.

#### Biology.

Reared from *Phanerotoma flava* Ashmead, 1906 (Braconidae), parasitoid of *Locastra muscosalis* (He, 2004). Collected in January, June and October.

#### Distribution.

China (Shandong, Zhejiang, Taiwan, Fujian, Hunan, Guangxi); Japan (Honshu, Kyushu, Ryukyu); Philippines.

### Taeniogonalos sculpturata sp. n.

http://zoobank.org/60FAD08B-FB15-4964-92BB-D3F0970F3D35

http://species-id.net/wiki/Taeniogonalos_sculpturata

Figs 485 –495

#### Type material.

Holotype, ♀ (SCAU) “[China:] Guizhou, Mt. Fanjing, Huixiangping, 11.VII.1993, Zai-fu Xu, 936625”.

#### Diagnosis.

Supra-antennal elevation 0.4 times as long as scapus and outer side of elevations oblique (Fig. 487) occipital carina widened medio-dorsally and crenulate (Fig. 487); head dorso-posteriorly densely punctate and entirely black (female) (Fig. 487); basal half of antenna (except scapus) yellowish brown (Fig. 488); mesosoma mainly or entirely orange-brown dorsally (Fig. 491) or black with extensive yellowish and orange pattern with middle mesoscutal lobe similar to lateral lobes (Fig. 491); notauli posteriorly and scutellar sulcus wide and both coarsely crenulate (Fig. 491); fore wing without subapical infuscation (Fig. 489); second sternite of female distinctly convex (Fig. 494).

**Figures 485–487. F94:**
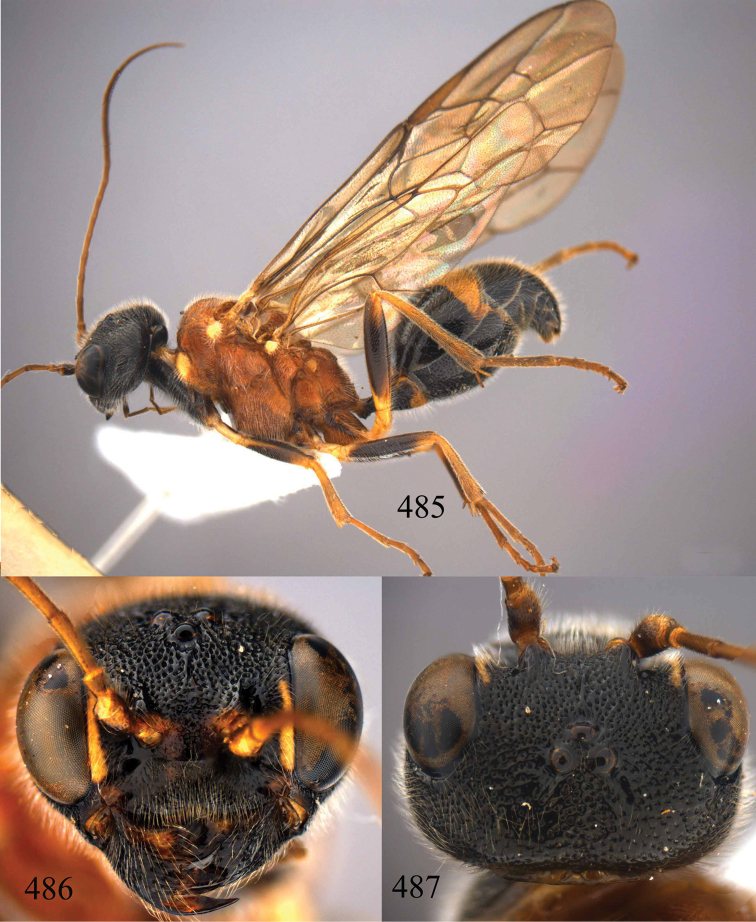
*Taeniogonalos sculpturata* sp. n., holotype, female. **485** Habitus lateral **486** head anterior **487** head dorsal.

**Figures 488–495. F95:**
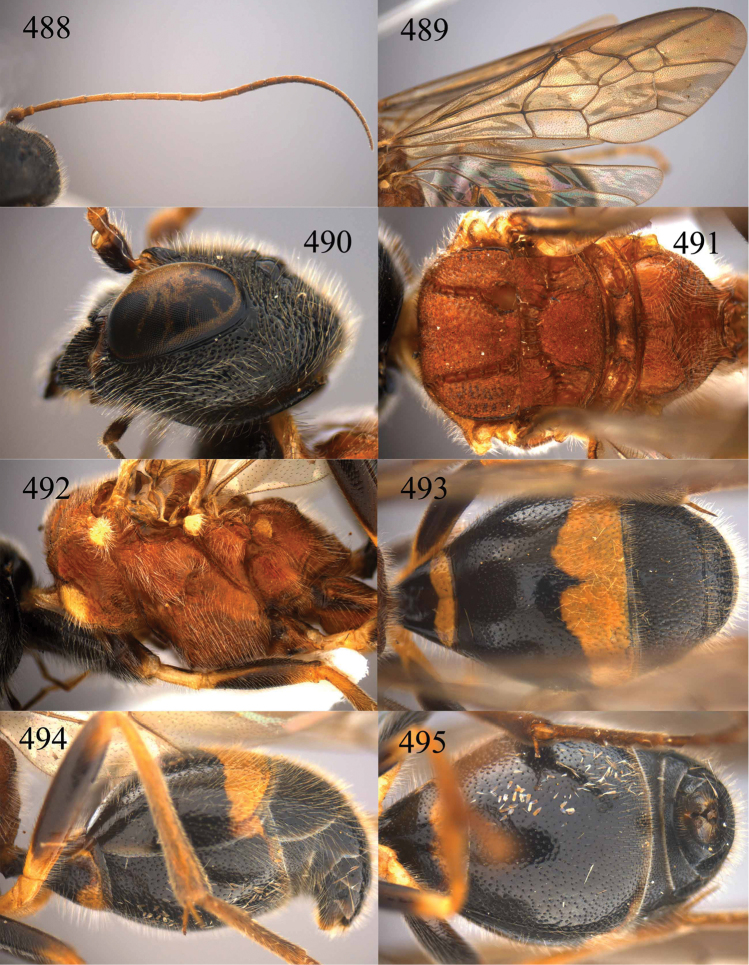
*Taeniogonalos sculpturata* sp. n., holotype, female. **488** Antenna **489** fore and hind wings **490** head lateral **491** mesosoma dorsal **492** mesosoma lateral **493** metasoma dorsal **494** metasoma lateral **495** metasoma ventral.

#### Description.

Holotype, female, length of body 12.3 mm (of fore wing 11.1 mm).

*Head*. Antenna with 26 segments; frons coarsely reticulate-punctate (Fig. 486); vertex coarsely reticulate-punctate behind stemmaticum and near eyes, becoming spaced punctate (interspaces much wider than width of punctures) posteriorly (Fig. 487); temple reticulate-punctate dorsally, becoming spaced punctate ventrally; head gradually narrowed behind eyes, eye in dorsal view 1.2 times as long as temple (Fig. 487); occipital carina widened medio-dorsally and crenulate (Fig. 487); supra-antennal elevations medium-sized (about 0.4 times as long as scapus), outer side oblique and largely smooth except for sparse punctures; clypeus slightly concave and thick medio-ventrally.

*Mesosoma*. Length of mesosoma 1.4 times its height (Fig. 492); mesopleuron below transverse mesopleural groove transversely rugose anteriorly and smooth posteriorly, above groove entirely transversely rugose (Fig. 492); transverse mesopleural groove rather wide, deep and coarsely crenulate; notauli wide, deep and coarsely crenulate; middle lobe of mesoscutum coarsely reticulate-rugose, lateral lobe mainly rugose; scutellar sulcus wide, both medially and laterally and coarsely crenulate; scutellum coarsely reticulate-rugose, convex medially and anteriorly near level of mesoscutum; metanotum medially slightly convex, not protruding and rugose (Fig. 491); propodeum rugulose to smooth antero-laterally, transversely striate medially, irregularly rugulose posteriorly (Fig. 491); posterior propodeal carina thick lamelliform and slightly arched, foramen medially 0.4 times higher than wide basally.

*Wings*. Fore wing: length of vein 1-M twice as long as vein 1-SR (Fig. 489).

*Metasoma*. First tergite 0.6 times as long as apically wide, largely smooth and with distinct elliptical depression medially (Fig. 493); second tergite spaced and rather finely punctate, third–sixth tergites densely and coarsely punctate; first and second sternites spaced and rather finely punctate, third–fifth sternites densely and finely punctate; second sternite distinctly convex, without medio-apical protuberance (Fig. 494); third sternite about 0.1 times as long as second sternite (Fig. 495); hypopygium triangular in ventral view (Fig. 494).

*Colour*. Head black with pale yellow stripes along inner orbita and apex of supra-antennal elevations and dorsal-medial occipital carina brown; mesosoma orange-brown dorsally (Fig. 491); mesosoma largely orange-brown laterally except for anterior and dorsal yellow patches of pronotal side and dark brown propleuron; metasoma with moderately wide orange-yellow apical band at apex of first tergite and sternite, second tergite with broad orange-yellow band at posterior margin, remainder of metasoma black; palpi dark brown; mandible black with teeth dark brown; basal half of antenna (except scapus) yellowish brown, scapus and apical half of antenna dark brown (Fig. 488); trochanters and trochantelli and base of femora of all legs pale yellow, tibiae and tarsi of fore and mid leg yellowish brown, mid and hind coxae orange-brown, remainder of legs dark brown; pterostigma dark brown, remainder of wing membrane subhyaline.

*Male*. Unknown.

#### Biology.

Unknown. Collected in July.

#### Distribution.

China (Guizhou).

#### Etymology.

Named after the coarsely sculptured notauli and scutellar sulcus: from “*sculpturatus*” (Latin for “sculptured”).

### Taeniogonalos subtruncata nom. n.

**Figures 496–499. F96:**
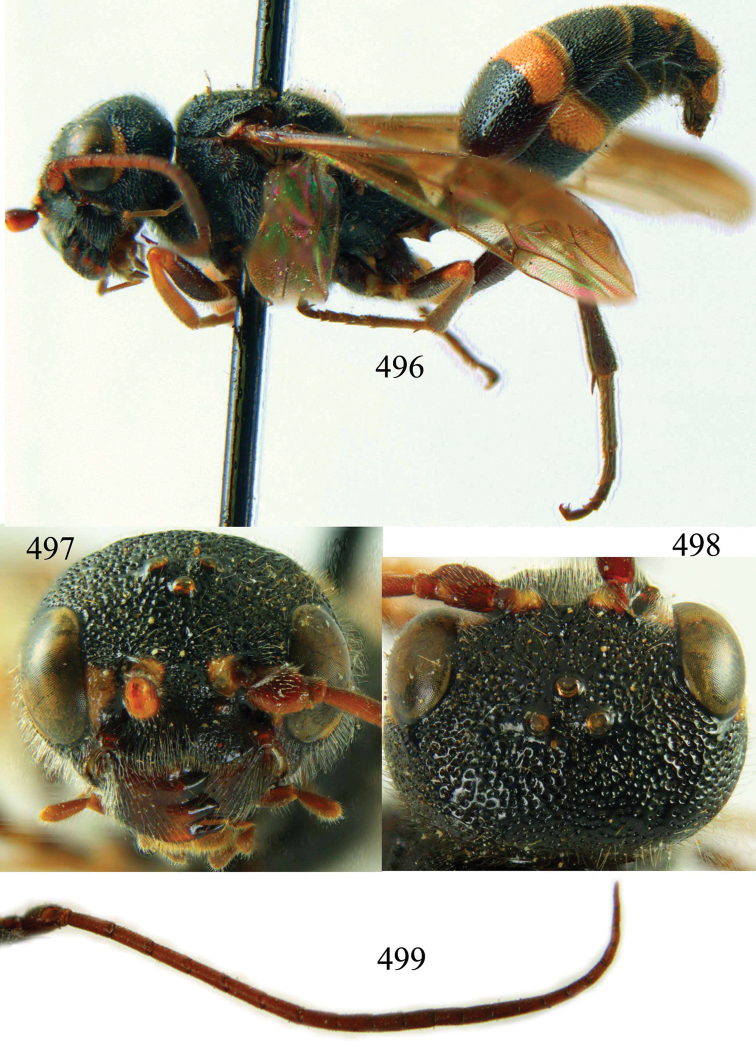
*Taeniogonalos flavocincta* (Teranishi, 1929), holotype, male. **496** Habitus lateral **497** head anterior **498** head dorsal **499** antenna.

Figs 496 [Fig F97] [Fig F98] –517

Nanogonalos flavocincta Teranishi, 1929: 140; Weinstein and Austin 1991: 421.
Poecilogonalos flavocincta ; Marshakov 1981: 107; Lelej 1995: 14. Synonymized with *Nanogonalos mongolicus* by Marshakov 1981.
Taeniogonalos flavocincta ; Carmean and Kimsey 1998: 67 [not *Taeniogonalos flavicincta* (Bischoff, 1913)].

#### Type material.

Holotype, ♂ (OMNH), “Corea, Suigen, V.1928, C.P. Clausen”, “Holotype *Nanogonalos flavicincta* [*sic*!]”.

#### Additional material.

1 ♀ (IZCAS) “[China:] Shaanxi, Zhenping, 21.VII.1983, IOZ(E)1495446”.

#### Diagnosis.

Supra-antennal elevations 0.4–0.5 times as long as scapus and outer side of elevations oblique, largely smooth (except for sparse punctures) and apically brown (Figs 498, 509); tyloids of male antenna linear; occipital carina distinctly lamelliform, moderately widened (about 0.5 times as wide as diameter of ocellus) and smooth medio-dorsally (Fig. 498); head posteriorly with two small ivory spots (Fig. 509; female) or entirely black (male); head dorsally coarsely reticulate-punctate and largely without distinct smooth interspaces (Figs 498, 509); mesosoma black dorsally except for ivory patch(es) on metanotum medially (Fig. 501); mesoscutum and scutellum coarsely punctate-reticulate (Fig. 501); scutellum entirely black (Fig. 501); anterior half of fore wing dark brown (Fig. 506); third submarginal cell of fore wing nearly as long as second submarginal cell (Fig. 506); first discal cell of fore wing comparatively elongate (Fig. 506); metasoma coarsely punctate (Fig. 503), with wide band at apex of second tergite and sternite, and fourth-sixth tergites largely orange or yellowish and remainder of metasoma black (Fig. 503); first tergite about half as long as its apical width; protuberance of second sternite of female subtruncate medio-apically and in lateral view curved and rounded apically (Figs 516, 517); second sternite of male distinctly shallowly depressed medio-posteriorly (Fig. 505); third sternite of male flat medially; third sternite 0.3–0.4 times as long as second sternite.

**Figures 500–506. F97:**
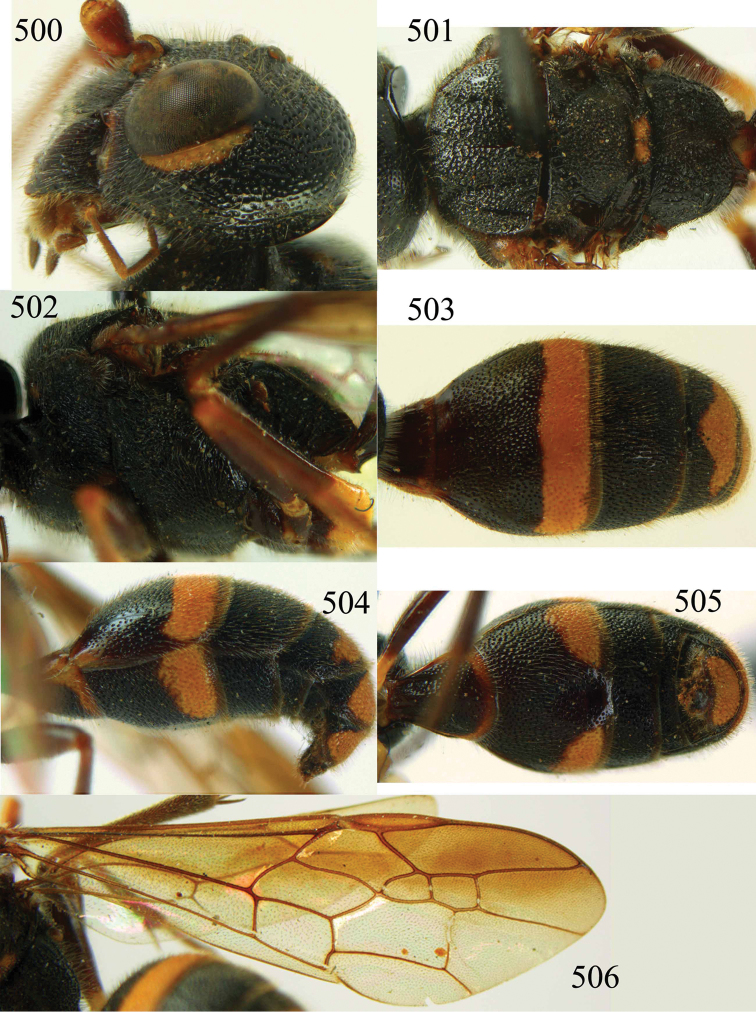
*Taeniogonalos flavocincta* (Teranishi, 1929), holotype, male. **500** Head lateral **501** mesosoma dorsal **502** mesosoma lateral **503** metasoma dorsal **504** metasoma lateral **505** metasoma ventral **506** fore wing.

**Figures 507–509. F98:**
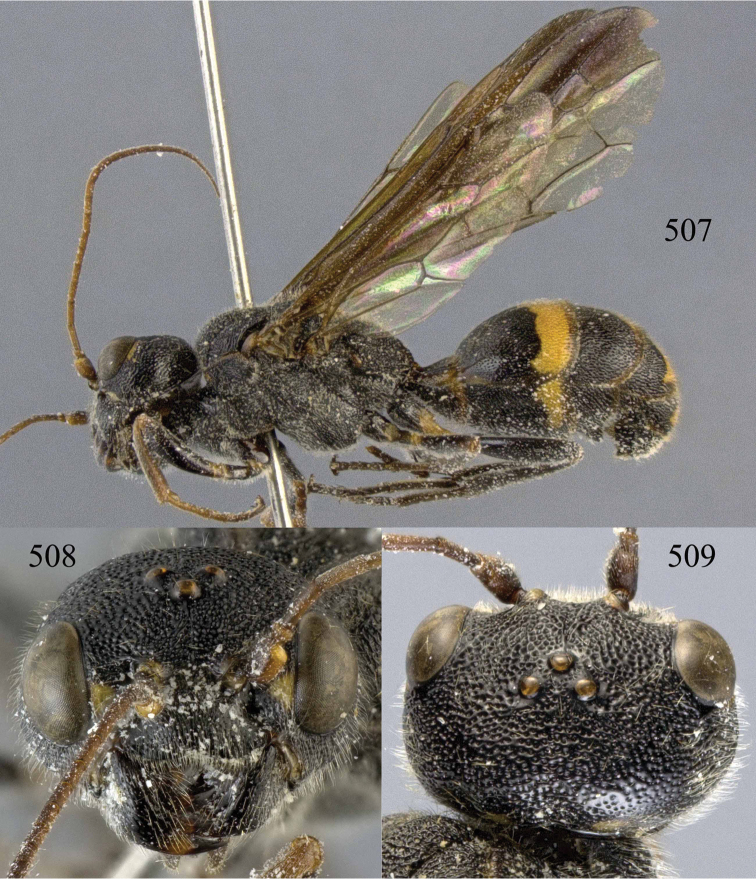
*Taeniogonalos subtruncata* nom. n., female from Shaanxi. **507** Habitus lateral **508** head anterior **509** head dorsal.

**Figures 510–517. F99:**
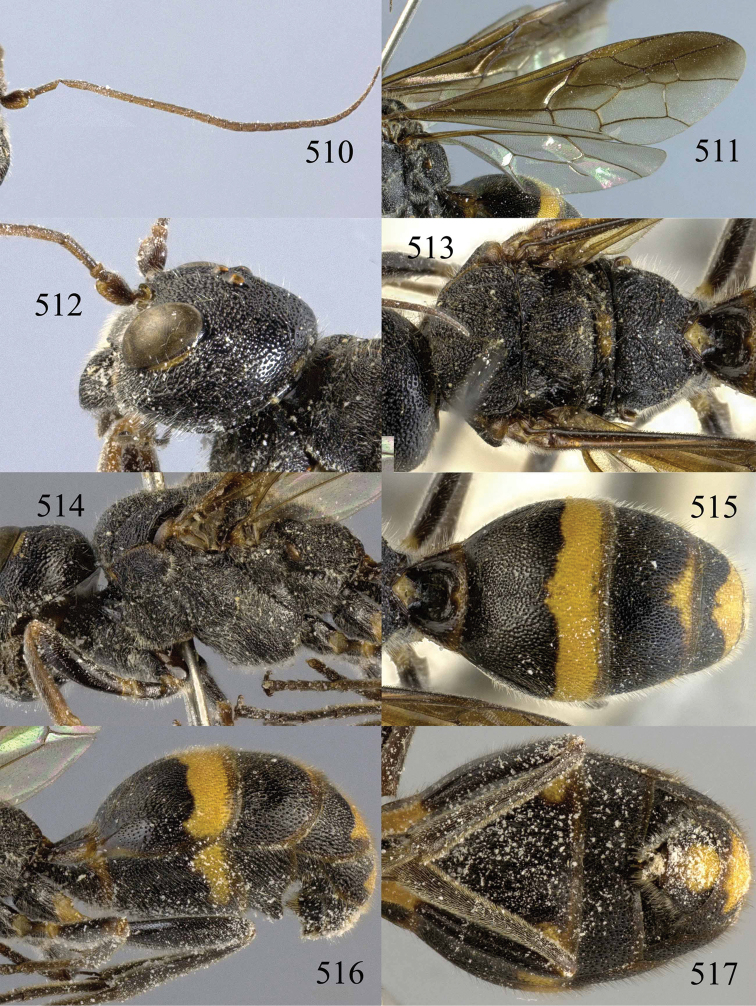
*Taeniogonalos subtruncata* nom. n., female from Shaanxi. **510** Antenna **511** fore and hind wings **512** head lateral **513** mesosoma dorsal **514** mesosoma lateral **515** metasoma dorsal **516** metasoma lateral **517** metasoma ventral.

#### Description.

Female from Shaanxi, length of body 12.4 mm (of fore wing 9.8 mm).

*Head*. Antenna with 25 segments; frons, vertex and temple coarsely reticulate-punctate (Figs 508, 509, 512); head gradually narrowed behind eyes, eye in dorsal view 0.7 times as long as temple (Fig. 509); occipital carina narrowly lamelliform and smooth medio-dorsally; supra-antennal elevations medium-sized (about 0.4 times as long as scapus), outer side oblique and largely smooth except for sparse punctures; clypeus slightly concave and thick medio-ventrally.

*Mesosoma*. Length of mesosoma 1.5 times its height (Fig. 514); mesopleuron coarsely reticulate-punctate anteriorly, transversely rugulose posteriorly; transverse mesopleural groove narrow, deep and moderately crenulate; notauli narrow, deep and finely crenulate; mesoscutum coarsely reticulate-punctate (Fig. 513); scutellar sulcus complete, medium-sized and crenulate medially and wider laterally; scutellum coarsely reticulate-punctate, convex medially and anteriorly slightly above level of mesoscutum; metanotum medially slightly convex, not protruding and rugose (Fig. 513); propodeum coarsely rugose (Fig. 513); posterior propodeal carina thick lamelliform and strongly arched, foramen medially 0.4 times higher than wide basally.

*Wings*. Fore wing: length of vein 1-M 1.8 times as long as vein 1-SR (Fig. 511).

*Metasoma*. First tergite 0.6 times as long as apically wide, largely smooth and with shallow elliptical depression medially (Fig. 515); second–sixth tergites densely and coarsely punctate; sternites densely and coarsely punctate; second sternite distinctly convex in lateral view, its medio-apical protuberance medium-sized and blunt medio-apically (Fig. 517); third sternite about 0.4 times as long as second sternite, with two close large triangular protuberances medially (Fig. 517); hypopygium triangular in ventral view (Fig. 517).

*Colour*. Black; palpi dark brown; mandible black with teeth dark brown; antenna dark brown; frons with small yellowish brown spots along inter orbita, remainder of head black except for posterior vertex with small yellowish brown spots and narrow yellowish brown stripes along outer orbita; apex of supra-antennal elevations yellowish brown; tegulae dark brown; mesosoma laterally black except for antero-dorsal yellowish brown patch of pronotal side; metanotum with one medium-sized medial yellowish brown patch; hind trochanter, trochantellus and base of hind femur ivory, remainder of legs dark brown to black with tarsi paler; anterior half of fore wing dark brown, posterior half subhyaline; metasoma with moderate wide orange-brown apical band at apex of second tergite and sternite, third tergite with narrow orange-brown band at posterior margin, fourth to sixth tergites with large orange-brown patches posteriorly.

*Male*. Holotype male is very similar to the redescribed female except shape of second sternite. It has length of body 10.7 mm (of fore wing 8.1 mm); brown antenna with 23 segments with longitudinal tyloids on 10^th^-16^th^ segments; outer orbita largely and pronotum dorso-posterioly ivory, head posteriorly and third tergite apically black; second sternite of male distinctly shallowly depressed medio-posteriorly; third sternite of male flat medially;

#### Biology.

Unknown. Collected in May and July.

#### Distribution.

China (Shaanxi), Korea.

#### Etymology.

Renamed after the blunt (subtruncate) protuberance of the second sternite of the female: from “sub” (Latin for “under, less”) and “truncus” (Latin for “cut off”).

#### Notes.

The replacement name of *Taeniogonalos subtruncata* is necessary after transferring *flavicincta* Bischoff, 1913 from the genus *Lycogonalos* Bischoff, 1913 and *flavocincta* Teranishi, 1929 from the genus *Nanogonalos* Schulz, 1906 to the genus *Taeniogonalos* Schulz, 1906 (Carmean and Kimsey 1998). Both became secondary homonyms in spite that the names differ in one letter (articles 57.3.1, 58.12 of ICZN 1999; Lelej 2003).

### Taeniogonalos taihorina

(Bischoff, 1914)

http://species-id.net/wiki/Taeniogonalos_taihorina

**Figures 518–520. F100:**
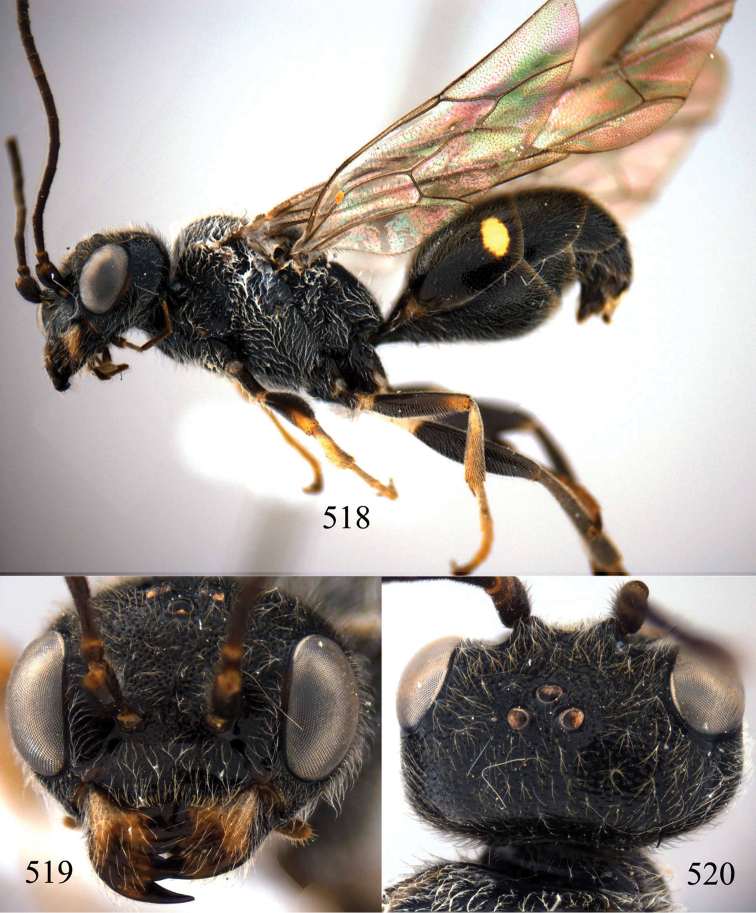
*Taeniogonalos taihorina* (Bischoff, 1914), female from Zhejiang. **518** Habitus lateral **519** head anterior **520** head dorsal.

Figs 518 [Fig F101] [Fig F102] [Fig F103] [Fig F104] –548

Nanogonalos taihorina Bischoff, 1914: 93; Tsuneki 1991: 58; Weinstein and Austin 1991: 421.
Taeniogonalos taihorina ; Carmean and Kimsey 1998: 68.
Poecilogonalos yuasai Teranishi, 1938: 178; Weinstein and Austin 1991: 423. Synonymized by Tsuneki 1991 with *Taeniogonalos maga*. Syn. n.
Poecilogonalos maga taiwana Tsuneki, 1991: 55–56. Syn. n.

#### Type material.

Holotype of *Poecilogonalos taihorina*, ♀ (ZMB), “[China:] Formosa, Taihorin, X.[19]10, H. Sauter S.G.”, “*taihorina* Bisch.*, det. Bischoff”, “Type”. Holotype of *Poecilogonalos yuasai*, ♀ (OPU), “[Japan: Honshu,] Tôkyô-hu, Hikawa, 4.X.1936, H. Yuasa”, “*Poecilogonalos yuasai* Teranishi, (Ty[p]es)”, “Type specimen”, “*Taeniogonalos maga* (Teranishi, 1929), ♀, det. Carmean ‘96”. Holotype of *Poecilogonalos maga taiwana*, ♀ (OMNH), “[China: Taiwan,] Arisan, 10.X.1912, I. Nitobe”, “3241”, “*Poecilogonalos maga taiwana* Tsuneki, ♀, holotype”.

#### Additional material.

1 ♀ (ZJUH), “[China:] Heilongjiang, Mt. Dailing, 24.VII.1977, Jun-hua He, No. 771709”; 1 ♀ (SCAU), “[China:] Heilongjiang, Yichun, Fenglin Nature Reserve, 22.VII.2008, Yi-ping Wang, SCAU 127”; 1 ♂ (ZJUH), “[China:] Gansu, Dandchang, Daheba, 2530 m, 31.VII.2004, Qiong Wu, 20047038”; 1 ♂ (ZJUH), “[China:] Gansu, Langzhou, 19.VII.1980, You-zheng Wang, 853598”; 1 ♀ + 3 ♂ (SCAU) “[China:] Ningxia, Mt. Liupan, Qiuqianjia, 9–11.VII.2009, Guang-yue Wang, SCAU 019; id., but 2.VII.2008, Jing-xian Liu, 200800778; id., but 8.VII.2008, 200801032, 200801040”; 1 ♂ (SCAU), “[China:] Ningxia, Mt. Liupan, Hongxia Forestry Farm, 1–7.VII.2008, Jie-Min Yao, 200808928”; 1 ♂ (SCAU) id., but 1.VII.2008, Jing-xian Liu, 200800701; 1 ♂ (SCAU) “[China:] Ningxia, Pengyang, Guamagou, 9.VII.2008, Jing-xian Liu, 200801050”; 1 ♂ (SCAU) “[China:] Hubei, Shennonjia, 2.VI.2010, Mao-ling Sheng, reared from *Vibrissina turrita* (Diptera: Tachinidae) parasitizing *Arge pullata* (Hymenoptera: Argidae), SCAU 360”; 2 ♀ + 1 ♂ (ZJUH) “[China:] Zhejiang, Mt. Tianmu, 18.VI.1983, Yun Ma, 831379; id., but 12.VI.1993, 934326; id., VIII.1981, Xue-xin Chen, 816314; 3 ♀ + 16 ♂ (ZJUH, RMNH), “[China:] Zhejiang, Mt. Tianmu, Xianrending, 1509 m, 1.VII.2001, Mei-hua Piao, 200106397–200106403”; id., but 28–29.VII.2003, Xue-xin Chen, 20034488, 20034516; id., but 2–4.VI.1990, Xin-geng Wang, 902855, 902857; id., but 5.X.1998, Ming-shui Zhao, 20057338, 200010700; id., but 20.VII.1998, MT, 992749; id., but 14.IX.1998, 200010704, 200010705, 200010682, 200010685; 1 ♀ + 2 ♂ (ZJUH), “[China:] Zhejiang, Longquan, Mt. Fengyang, 27.VI.2007, Sheng-long Liu, 200704940, 200704941; id., but 30.VI.2007, 200704934”; 1 ♀ + 2 ♂ (ZJUH, RMNH), id., but 30.V.2007, 200704935, 200704938; id., but 11.VII.1984, 8433556; 1 ♂ (ZJUH, RMNH) “[China:] Zhejiang, Mt. Tianmu, Xianrending, 5.IX.1998, MT, Ming-shui Zhao, 200010701; 1 ♂ (ZJUH) “[China:] Zhejiang, West Mt. Tianmu, Laodian–Xianrending, 1250–1506 m, 6.VI.1989, Xin-geng Wang, 892773”; 1 ♀ + 1 ♂ (ZJUH) “[China:] Zhejiang, Songyang, 15–17.VII.1989, Jun-hua He, 894126, 894073”; 1 ♀ (ZJUH) “[China:] Fujian, Fuzhou, 25.IV.1991, Chang-ming Liu, 966572”; 1 ♂ (ZJUH) “[China:] Taiwan, Nantou, Mt. Lu, 19.VI.2011, Pu Tang, 201101478”; 1 ♂ (ZJUH) “[China:] Sichuan, Wolong National Nature Reserve, 21.VII.2006, Hong-ying Zhang, 200610801”; 2 ♂ (ZJUH) “[China:] Sichuan, Pingwu, Baimazhai, 24.VII.2006, Zhi-lei Gao, 200610893; id., but 25.VII.2006, Hong-ying Zhang, 200611106”; 1 ♂ (ZJUH) “[China:] Tibet, Lage–Hanmi, 12.VI.2009, Jiang-li Tan, 200908326”; 1 ♀ (ZJUH) “[China:] Yunnan, Dali, Yunlong, 3.VI.2009, Jiang-li Tan, 200906647”; 1 ♀ (IZCAS) “[China:] Guangxi, Jinxiu, Jinzhong Road, 1100 m, 12.V.1999, Wen-zhu Li, SCAU 343”; 1 ♂ (IZCAS) “[China:] Guangxi, Jinxiu, Yonghe, 500 m, 12.V.1999, De-cheng Yan, SCAU 098”.

#### Diagnosis.

Outer side of supra-antennal elevations comparatively steep (angle about 45°) and elevations about 0.5 times as long as scapus and area between elevations distinctly concave (Figs 520, 531, 542); occipital carina narrow, non-lamelliform and smooth medio-dorsally or nearly so (Figs 520, 531, 542); head anteriorly and posteriorly, mesosoma dorsally and pronotum laterally entirely black (Figs 519, 520, 524); notauli crenulate posteriorly (Fig. 524), rarely indistinctly so; middle mesoscutal lobe similarly coloured to lateral lobes, yellow latero-posteriorly, black or yellow laterally and black medially (Figs 524, 535, 545); scutellum entirely black (Fig. 524); metanotum of male black medially or with pair of yellow (often small) patches; anterior half of fore wing only below pterostigma or subapically dark brown and remainder subhyaline (Figs 522, 533, 544); propodeal foramen comparatively narrow and more arched than in *Taeniogonalos formosana* (Fig. 524); second sternite of male flattened medio-posteriorly and bicoloured or entirely black; third sternite of female simple (Fig. 528); metasoma dorsally black with yellowish pattern (Figs 538, 546).

**Figures 521–528. F101:**
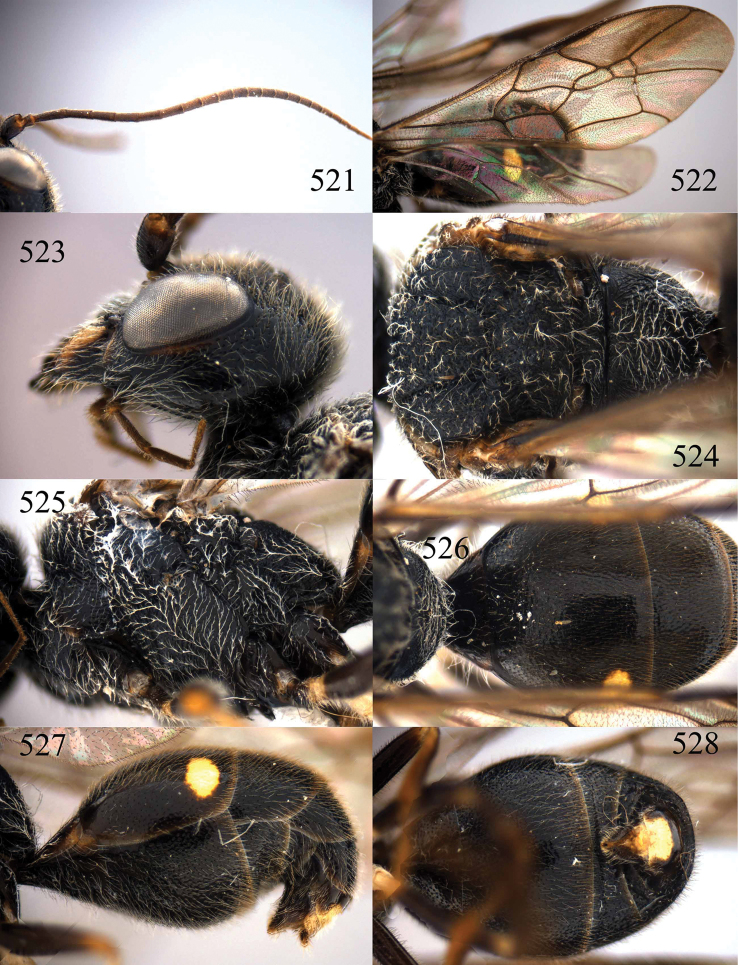
*Taeniogonalos taihorina* (Bischoff, 1914), female from Zhejiang. **521** Antenna **522** fore and hind wings **523** head lateral **524** mesosoma dorsal **525** mesosoma lateral **526** metasoma dorsal **527** metasoma lateral **528** metasoma ventral.

**Figures 529–533. F102:**
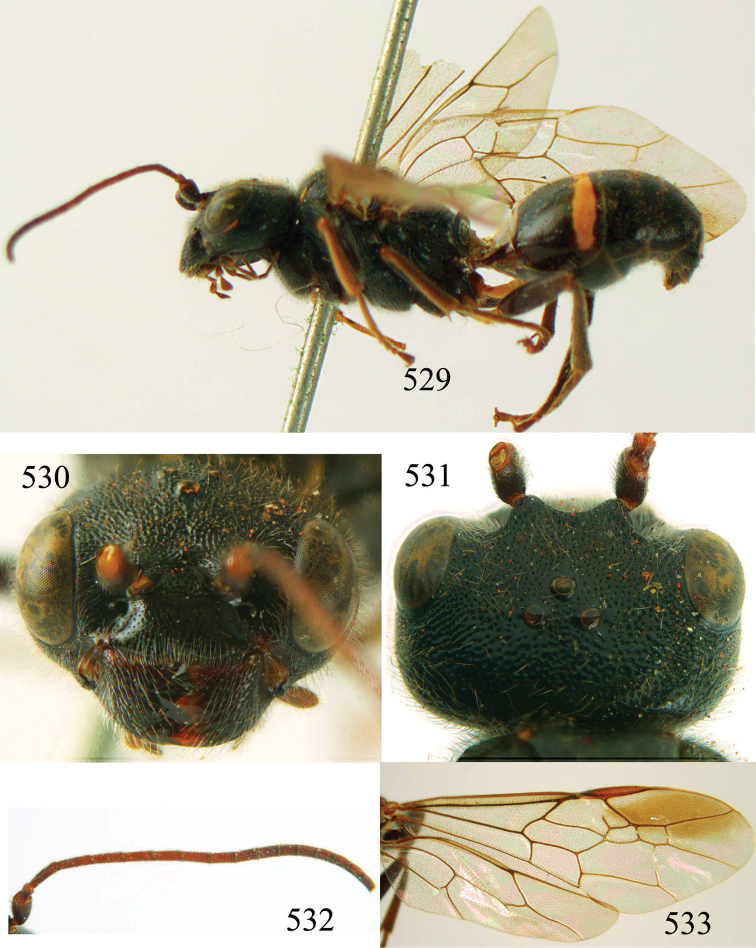
*Taeniogonalos yuasai* (Teranishi, 1938), holotype, female. **529** Habitus lateral **530** head anterior **531** head dorsal **532** antenna **533** fore and hind wings.

**Figures 534–539. F103:**
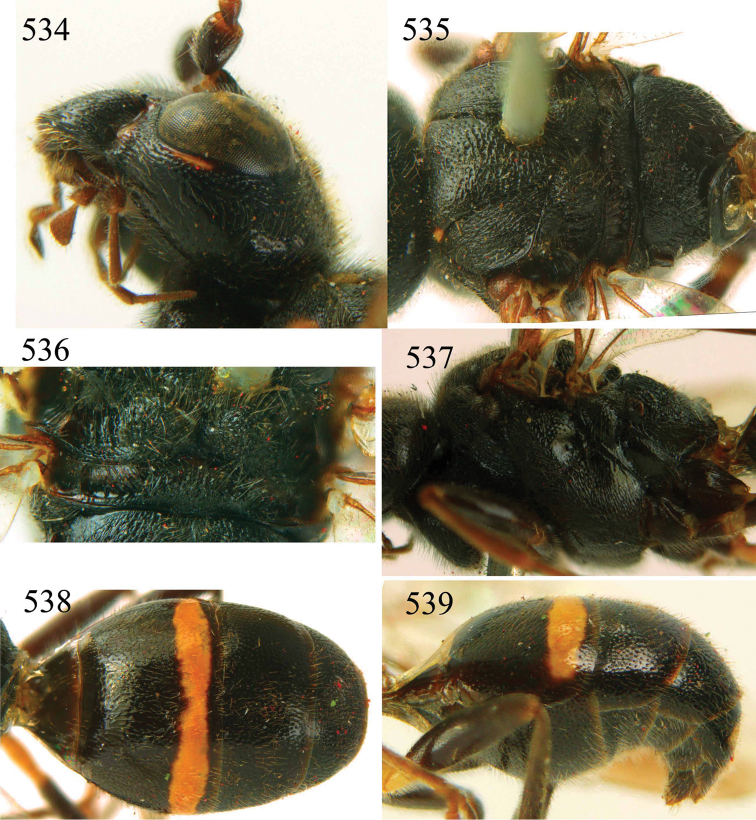
*Taeniogonalos yuasai* (Teranishi, 1938), holotype, female. **534** Head lateral **535** mesosoma dorsal **536** metanotum dorsal **537** mesosoma lateral **538** metasoma dorsal **539** metasoma lateral.

**Figures 540–542. F104:**
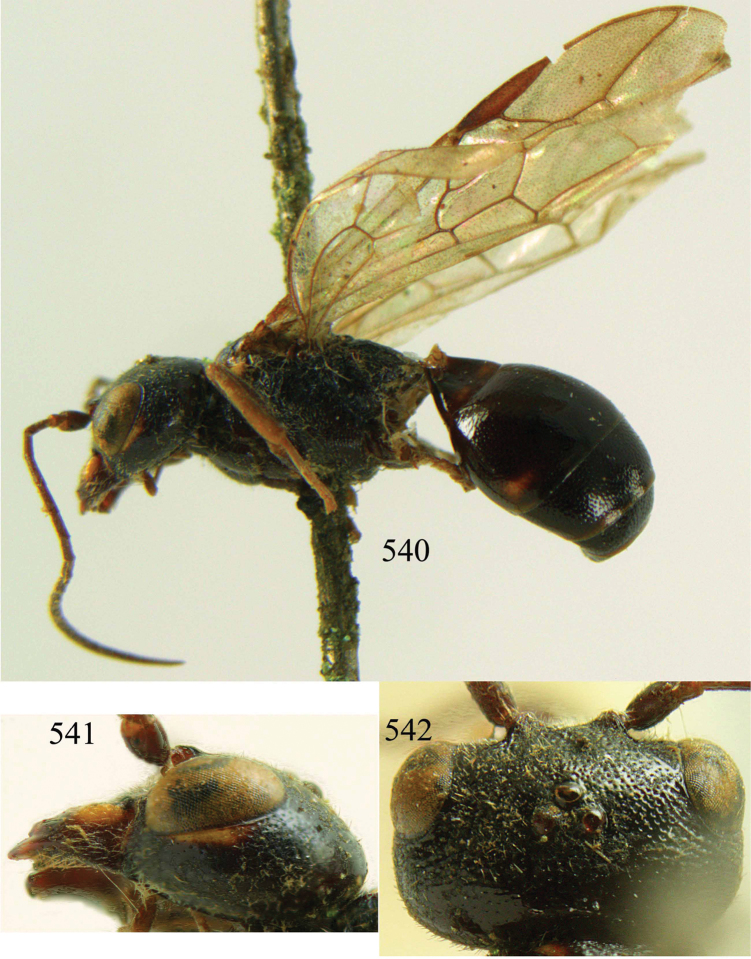
*Taeniogonalos maga taiwana* (Tsuneki, 1991), holotype, female. **540** Habitus lateral **541** head lateral **542** head dorsal.

**Figures 543–548. F105:**
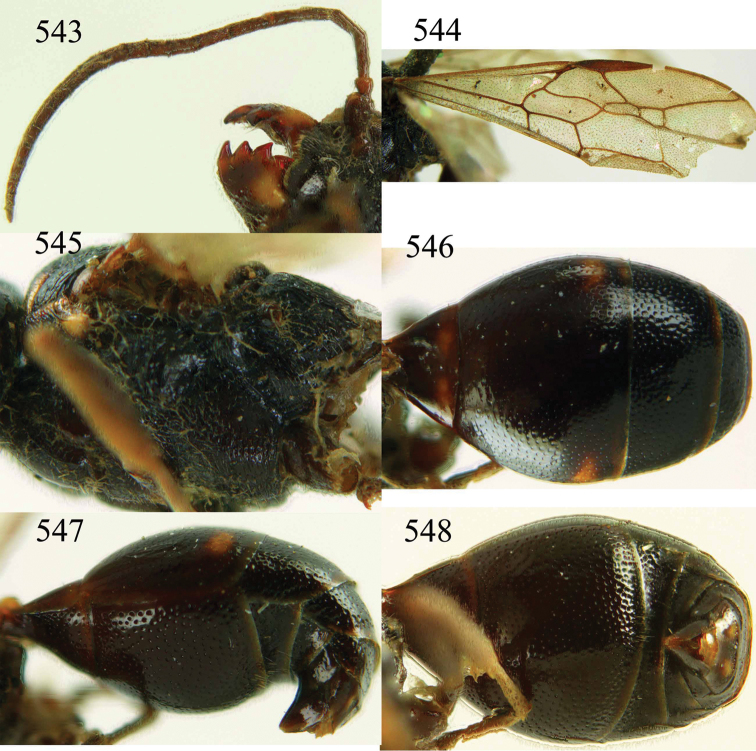
*Taeniogonalos maga taiwana* (Tsuneki, 1991), holotype, female. **543** Antenna and mandibles **544** fore wing **545** mesosoma lateral **546** metasoma dorsal **547** metasoma lateral **548** metasoma ventral.

#### Description.

Holotype of *Taeniogonalos taihorina*, female, length of body 5.7 mm (of fore wing 5.0 mm).

*Head*. Antenna with 22 segments; frons densely punctate and medium-sized smooth interspaces; vertex punctate with mostly wide smooth interspaces and shiny (Fig. 520), with medium-sized setae; temple largely smooth with sparse fine punctures (Fig. 523), but less sparsely near eye; head subparallel behind eyes, eye in dorsal view as long as temple (Fig. 520); occipital carina narrow, non-lamelliform, smooth (Fig. 520); supra-antennal elevations medium-sized (about 0.5 times as long as scapus), outer side oblique (angle about 45°) lamelliform and with some punctures (Fig. 520); area between elevations distinctly concave (Fig. 520); clypeus shallowly concave and thick medio-ventrally.

*Mesosoma*. Length of mesosoma 1.4 times its height (Fig. 525); mesopleuron densely and finely punctate-rugose but smooth posteriorly, with satin sheen; transverse mesopleural groove medium-sized, shallow and distinctly crenulate; notauli narrow, deep and finely crenulate posteriorly; middle lobe of mesoscutum transversely rugose, lateral lobes spaced rugose and with satin sheen (Fig. 524); scutellar sulcus complete, narrow and narrowly crenulate; scutellum densely rugose but superficially coriaceous-rugulose posteriorly, convex laterally and near level of mesoscutum, flattened medially and posteriorly; metanotum medially weakly convex and coriaceous-rugulose (Fig. 524); propodeum laterally obliquely rugose and medially longitudinally rugulose bot posteriorly smooth (Fig. 524); posterior propodeal carina thick lamelliform and medium-sized, foramen narrower and more arched than in *Taeniogonalos formosana* (Fig. 524) and 1.4 times higher than wide basally.

*Wings*. Fore wing: vein 1-M 1.3 times as long as vein 1-SR (Fig. 522), distinctly curved and vein 1-SR widened anteriorly; second submarginal cell about twice as long as third cell.

*Metasoma*. First tergite 0.5 times as long as apically wide, smooth and with shallow wide oval depression medially (Fig. 524); second tergite superficially punctate and with vague transverse elements anteriorly, other tergites largely densely punctate (Fig. 524); second sternite densely punctate; second sternite strongly convex and medio-posteriorly gradually lowered and only apical rim densely setose in lateral view (Fig. 527); third sternite distinctly depressed posteriorly and sparsely punctate.

*Colour*. Black; ventrally outer orbita narrowly yellow (inner orbita black or dark brown); supra-antennal elevations dark brown (Fig. 520), clypeus and head posteriorly black; mandibles largely dark brown, but dorsally yellow; mesosoma laterally black or dark brown except for dorsal yellow patch of pronotal side; middle lobe of mesoscutum with pair of narrow brownish stripes anteriorly; tegulae brown; scutellum laterally and axilla black; metanotum black or dark brown; propodeum black (Fig. 524); first tergite dark brown medio-posteriorly, remainder of metasoma black or nearly so (Fig. 526); palpi brown; antenna; mainly dark brown; apex of trochanters, trochantelli largely, anterior stripe on fore tibia, base of all femora and apex of fore femur pale yellow; remainder of tibiae and tarsi more or less dark brown; pterostigma and apical half of marginal cell of fore wing largely and area below it infuscate; remainder of wing membrane subhyaline (Fig. 522).

*Male*. Length of fore wing 5.2–6.6 mm; body black, but sixth and seventh tergites ivory and genitalia dark brown; apex of first tergite black or ivory, second tergite apically ivory or only laterally with pale patch; second sternite distinctly flattened medially; tyloids linear.

*Variation*. Holotype of *Taeniogonalos yuasai* has head densely punctate dorsally, mandible entirely dark brown, hind trochanter entirely ivory, scutellum teratological (it has a deep median depression), vein 2-SR with extra vein, fifth and sixth tergites with pair of ivory patches. Second submarginal cell of fore wing 1.4–2.0 times as long as third cell.

#### Biology.

Reared from *Vibrissina turrita* (Diptera: Tachinidae) parasitizing *Arge pullata* (Hymenoptera: Argidae). Hyperparasitoid of Tachinidae in Argidae (Li et al. 2012). Collected in April–October at 500–2530 m.

#### Distribution.

China (Heilongjiang, Gansu, Ningxia, Zhejiang, Fujian, Taiwan, Hubei, Guangxi, Sichuan, Yunnan, Tibet); Russia (Far East); Japan (Hokkaido, Honshu).

#### Notes.

The holotype of *Poecilogonalos maga taiwana* differs mainly by having the postero-dorsal corner of the pronotum and a pair of small elongate patches on the mesoscutum anteriorly ivory and the body is comparatively weakly sculptured.

### Taeniogonalos triangulata sp. n.

http://zoobank.org/1817017B-B17F-4054-A9C1-B75D3D102D34

http://species-id.net/wiki/Taeniogonalos_triangulata

Figs 549 –559

#### Type material.

Holotype, ♀ (ZJUH) “[China:] Yunnan, Kunming, reared from Diprionidae (Hymenoptera), 15.VIII.1978, Jing-liang Qi, 803127”. Paratypes: 1 ♀ + 2 ♂ (ZJUH) “[China:] Yunnan, Kunming, reared from Diprionidae (Hymenoptera), 12.IX.1979, Jing-liang Qi, 803128”; 3 ♀ + 1 ♂ (ZJUH, RMNH) “[China:] Yunnan, Xiangyun, reared from Diprionidae (Hymenoptera), 12.VIII.1980, Hai-lin Wang, 888667, 888665, 888666; id., but VIII.1981, 840755”.

#### Diagnosis.

Supra-antennal elevations 0.5–0.7 times as long as scapus and outer side of elevations often subvertical, densely punctate, dull and apically black (Fig. 551); tyloids of male antenna linear; occipital carina smooth medio-dorsally (Fig. 551); third submarginal cell of fore wing 0.6–0.7 times as long as second submarginal cell (Fig. 553); first discal cell of fore wing comparatively elongate (Fig. 553); metasoma coarsely punctate (Fig. 557), with wide orange-brown band at apex of second tergite and sternite and remainder black (Fig. 557); first tergite about half as long as its apical width; second sternite of female triangularly protruding medio-apically and in lateral view acute apically (Fig. 558; but of male flat or slightly convex); third sternite 0.3–0.4 times as long as second sternite.

**Figures 549–551. F106:**
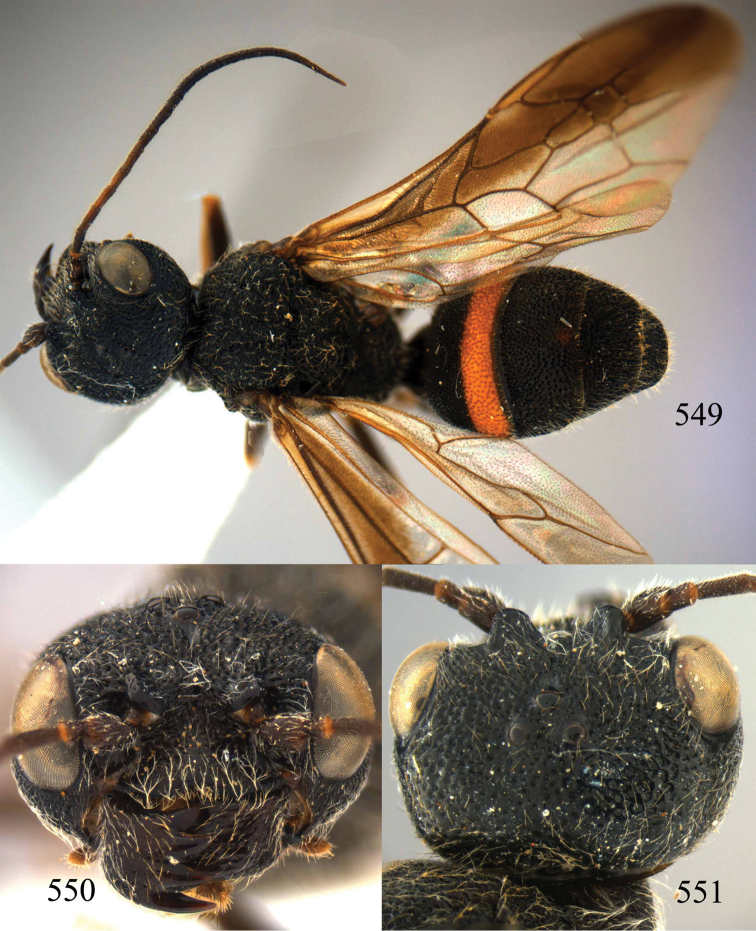
*Taeniogonalos triangulata* sp. n., holotype, female. **549** Habitus lateral **550** head anterior **551** head dorsal.

**Figures 552–559. F107:**
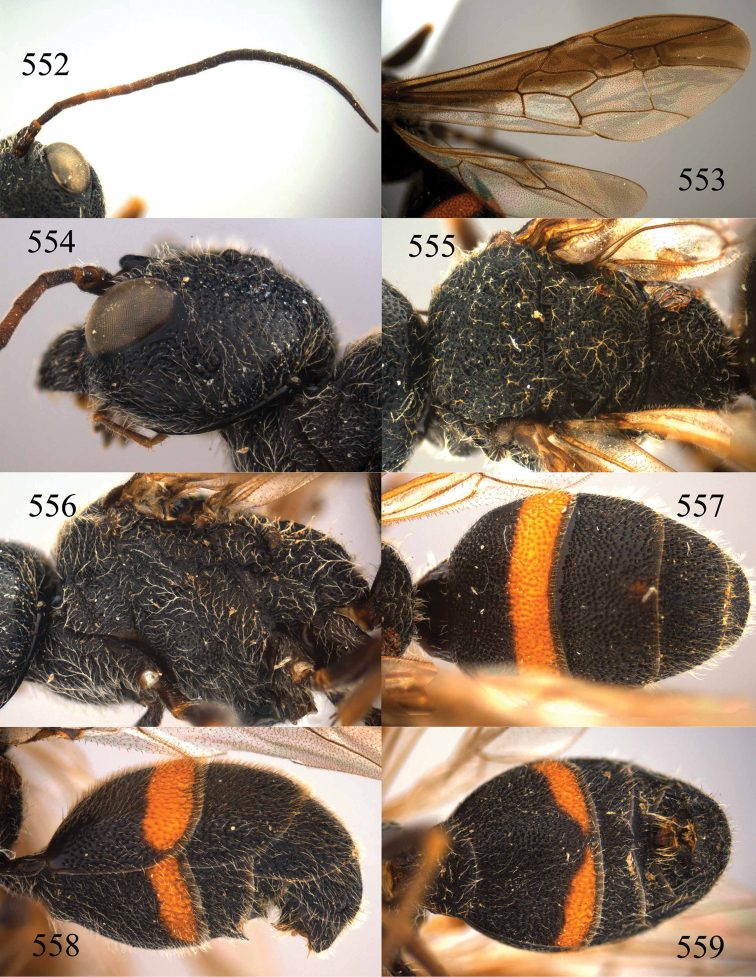
*Taeniogonalos triangulata* sp. n., holotype, female. **552** Antenna **553** fore and hind wings **554** head lateral **555** mesosoma dorsal **556** mesosoma lateral **557** metasoma dorsal **558** metasoma lateral **559** metasoma ventral.

#### Description.

Holotype, female, length of body 8.8 mm (of fore wing 7.1 mm).

*Head*. Antenna with 24 segments; frons, vertex and temple coarsely reticulate-punctate (Figs 550, 551); head gradually narrowed behind eyes, eye in dorsal view 0.8 times as long as temple (Fig. 551); occipital carina moderately widened and lamelliform, weakly crenulate medio-dorsally; supra-antennal elevations strongly enlarged (about 0.7 times as long as scapus), outer side subvertical and distinctly densely punctate; clypeus slightly concave and thick medio-ventrally.

*Mesosoma*. Length of mesosoma 1.6 times its height (Fig. 556); mesopleuron coarsely irregular rugose dorsally, coarsely reticulate-punctate ventrally; transverse mesopleural groove moderately wide, deep and coarsely crenulate; notauli moderately wide, deep and coarsely crenulate; mesoscutum coarsely reticulate-punctate (Fig. 555); scutellar sulcus wide, both medially and laterally and coarsely crenulate; scutellum coarsely reticulate-punctate, slightly convex and anteriorly near level of mesoscutum; metanotum medially slightly convex, not protruding and reticulate-punctate (Fig. 555); propodeum coarsely reticulate-rugose (Fig. 555); posterior propodeal carina thick lamelliform and slightly arched, foramen medially 0.8 times higher than wide basally.

*Wings*. Fore wing: length of vein 1-M 1.4 times as long as vein 1-SR (Fig. 553).

*Metasoma*. First tergite 0.7 times as long as apically wide, smooth and with distinct elliptical depression medially (Fig. 557); second–sixth tergites densely and coarsely reticulate-punctate (Fig. 557); sternites densely and coarsely punctate; second sternite distinctly convex, without medio-apical protuberance (Fig. 559); third sternite about 0.3 times as long as second sternite, with medio-apical protuberance triangularly protruding (Fig. 559); hypopygium triangular in ventral view (Fig. 559).

*Colour*. Black; metasoma largely black with wide orange-brown band at apex of second tergite and sternite; mandible nearly black, somewhat brownish; antenna dark brown to black, becoming darker apically; tibiae and tarsi yellowish brown, remainder of legs dark brown to nearly black; pterostigma and anterior half of fore wing dark brown, remainder of wing membrane subhyaline.

*Variation*. Length of body 8.8–10.1 mm, of fore wing 7.1–8.2 mm; length of vein 1-M of fore wing 1.4–1.6 times as long as vein 1-SR.

*Male*. Length of body 8.2–9.7 mm, of fore wing 6.8–7.9 mm; antenna with 23 segments, tyloids linear, 0.8 times as long as segment on 10^th^–14^th^ segments and 0.5 times as long as segment on 15^th^ segment; genitalia extruded.

#### Biology.

Reared from Diprionidae, probably hyperparasitoid of (Ichneumonoid or Tachinid) endoparasitoid. Collected in August–September.

#### Distribution.

China (Yunnan).

#### Etymology.

Named after the triangular medio-apical protuberance of the third sternite: “*triangulus*” is Latin for “having three angles”.

### Taeniogonalos tricolor

(Chen, 1949)

http://species-id.net/wiki/Taeniogonalos_tricolor

Figs 560 –570

Poecilogonalos tricolor Chen, 1949: 16; Weinstein and Austin 1991: 424; He and Lou 2001: 687.
Paecilogonalos (!) *tricolor*; He 2004: 75.
Taeniogonalos tricolour (!): Carmean and Kimsey 1998: 68.

#### Type material.

Lectotype here designated, ♀ (IZCAS) “[China: Zhejiang,] Mt. Tianmu, 17.VII.1937, Ouchi”, “*Poecilogonalos tricolor* Chen”. Paralectotype, 1 ♂ (IZCAS), topotypic, but 18.VII.1937.

#### Additional material.

1 ♀ (ZJUH) “[China:] Shaanxi, Ningshan, 22.VIII.1996, Hy-11080”; 1 ♀ + 1 ♂ (ZJUH) “[China:] Henan, Luoshan County, Mt. Ling, 22.V.2000, Ping Cai, 200101857, 200101870”; 1 ♀ (ZJUH) “[China:] Henan, Neixiang County, Mt. Baotianman, 13–15.VII.1998, sweeping, Yun Ma, 987289”; 1 ♀ (ZJUH) “[China:] Henan, Dengfeng, Mt. Shaoshi, 800 m, 9.VI.2000, Xiao-cheng Shen, 20047328”; 2♀ + 1 ♂ (ZJUH, RMNH) “[China:] Zhejiang, West Mt. Tianmu, 1.VII.1982, Rui-liang Wang, 825927; id., but 18.VI.1983, Jun-hua He, 831806; id., but 21.VI.2008, MT, SCAU 310”; 1 ♀ (ZJUH) “[China:] Hubei, Lichuan City, Moudao Town, 1410 m, 1.VIII.2003, Yong-xue Deng, Hy-645”; 2 ♂ (ZJUH) “[China:] Guizhou”; 1 ♂ (SCAU) “[China:] Sichuan, Hongya County, Mt. Wawu, 29°39.907’N, 102°56.457’E, 1920 m, 5.VII.2009, Geng-yun Niu, SCAU 319”; 1 ♀ (ZJUH) “[China:] Guangxi, Lintian, Langping, 30.V.1982, Jun-hua He, 822564”; 2 ♀ (IZCAS) “[China:] Guangxi, Jinxiu, Mt. Shengtang, 900–1900 m, 17.V.1999, Hong-xiang Han, 200105069; id., but 900 m, 200105073”; 1 ♂ (IZCAS) “[China:] Guangxi, Jinxiu, Jinzhong Road, 1100 m, 11.V.1999, De-cheng Yan, 200105072”; 1 ♂ (ZJUH) “[China:] Yunnan, Tengchong, 27.IV.1981, Jun-Hua He, 812174”; 1 ♀ (ZJUH) “[China:] Hainan, Mt. Jianfengling, Tianchi, 25.XI.2008, Man-man Wang, 200806072”.

#### Diagnosis.

Supra-antennal elevations 0.1–0.4 times as long as scapus and outer side of elevations oblique (Fig. 562); head posteriorly with extensive yellowish or orange-brown pattern, including a V-shaped yellow or orange pattern behind stemmaticum, area besides stemmaticum variable, often dark brown (Fig. 562); most of V-shaped pale area of head dorsally without distinct interspaces between punctures (Fig. 562); clypeus shallowly emarginate medio-ventrally (Fig. 561); middle mesoscutal lobe similar to lateral lobes, black or yellow laterally and black medially (Fig. 566); mesopleuron black or at most with medio-dorsal orange-brown patch and metapleuron black (Fig. 567); third submarginal cell of fore wing 0.7–1.0 times as long as second submarginal cell (Fig. 564); basal cell largely and posterior half of first submarginal cells of fore wing more or less darkened, similar to colour of marginal cell (Fig. 564); second tergite tricoloured: dark brown anteriorly, followed by intermediate chestnut brown area and a medially widened posterior yellow band or bicoloured (Fig. 568); fourth–sixth tergites without medial dark brown patch; second sternite of both sexes without medio-apical protuberance (Fig. 570), distinctly convex and no opening between second and following sternites in lateral view (Fig. 569); third sternite of female without apical ledge (Fig. 570); sixth tergite medially orange-brown or yellow (Fig. 570).

**Figures 560–562. F108:**
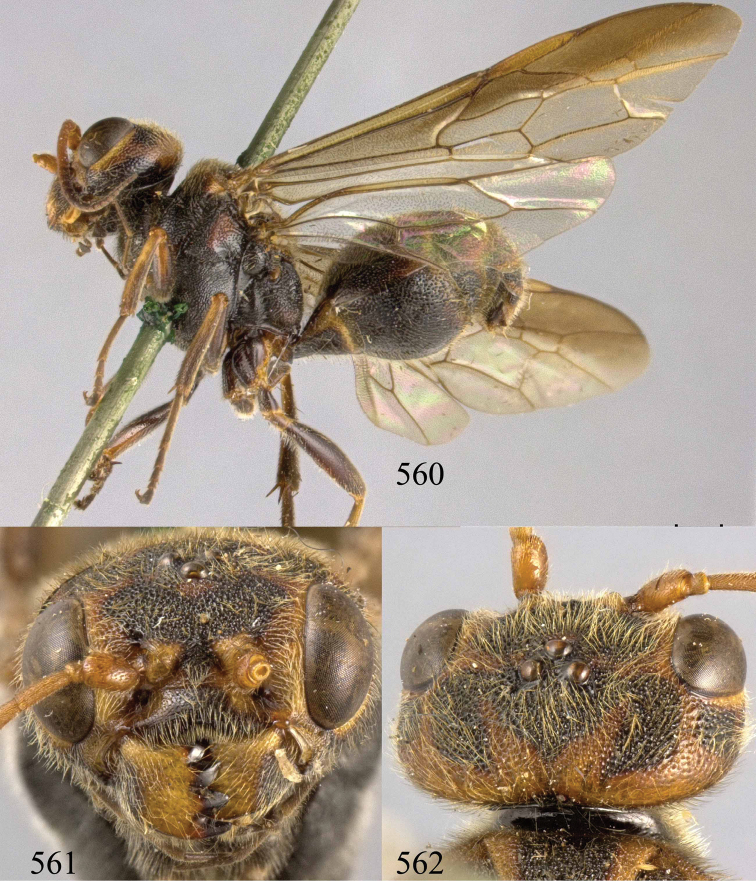
*Taeniogonalos tricolor* (Chen, 1949), lectotype, female. **560** Habitus lateral **561** head anterior **562** head dorsal.

**Figures 563–570. F109:**
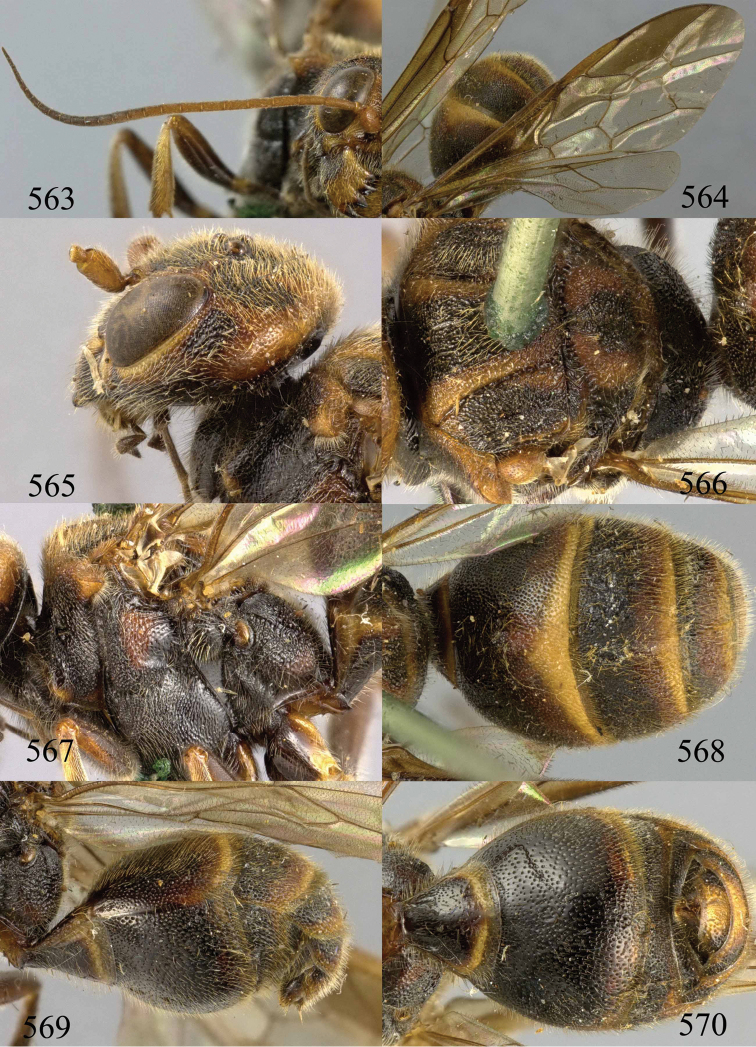
*Taeniogonalos tricolor* (Chen, 1949), lectotype, female. **563** Antenna **564** fore and hind wings **565** head lateral **566** mesosoma dorsal **567** mesosoma lateral **568** metasoma dorsal **569** metasoma lateral **570** metasoma ventral.

#### Description.

Lectotype, female, length of body 11.7 mm (of fore wing 10.8 mm).

*Head*. Antenna with 26 segments; frons, vertex and temple densely and coarsely reticulate-punctate (Figs 561, 562, 565); head gradually narrowed behind eyes, eye in dorsal view 0.7 times as long as temple (Fig. 562); occipital carina narrowly lamelliform and smooth medio-dorsally; supra-antennal elevations medium-sized (about 0.1 times as long as scapus), outer side oblique and largely smooth except for sparse fine punctures; clypeus moderately concave and thick medio-ventrally.

*Mesosoma*. Length of mesosoma about equal to its height (Fig. 567); mesopleuron below transverse mesopleural groove rugose anteriorly, rugulose posteriorly, above groove rugose anteriorly, sparsely punctate posteriorly; transverse mesopleural groove narrow, shallow and weakly crenulate; notauli moderately narrow, deep and coarsely crenulate; middle lobe of mesoscutum coarsely reticulate-punctate, lateral lobes somewhat longitudinally rugose (Fig. 566); scutellar sulcus complete, medium-sized and crenulate medially and wider laterally; scutellum densely and coarsely reticulate-punctate, convex anteriorly and near level of mesoscutum; metanotum medially flat and rugose (Fig. 566); propodeum mainly rugose, smooth at posterior end (Fig. 566); posterior propodeal carina thick lamelliform and strongly arched, foramen medially 0.8 times higher than wide basally.

*Wings*. Fore wing: length of vein 1-M 1.3 times as long as vein 1-SR (Fig. 564).

*Metasoma*. First tergite 0.8 times as long as apically wide, smooth and with distinct elliptical depression medially (Fig. 568); second–sixth tergites densely and coarsely reticulate-punctate (Fig. 568); first and second sternites sparsely punctate, third-fifth sternites coarsely reticulate-punctate; second sternite distinctly convex, without medio-apical protuberance (Fig. 570); third sternite about 0.2 times as long as second sternite (Fig. 570); hypopygium triangular in ventral view.

*Colour*. Head largely dark brown with following yellow or orange-brown markings: stripes along inner and outer orbita (including malar space), lateral corner of clypeus, V-shaped patch behind stemmaticum and oblique patches on postero-lateral vertex; mesosoma largely black with following yellow or yellowish brown makings: lateral stripes on middle lobe of mesoscutum, tegulae, lateral stripes on scutellum, metanotum, small spots on upper and lower pronotum, dorsal spot on mesopleuron, small lateral spots on propodeum; metasoma largely black with following dark brown or yellow markings: narrow stripe on posterior margin of first–fourth tergites and first sternite, large median patch on fifth and sixth tergites yellow, narrow stripe in front of yellow stripe on first–fourth tergites dark brown; mandible largely yellow with antero-ventral corner and teeth dark brown; palpi dark brown; basal half of antenna yellowish brown, apical half dark brown; legs dark brown to black; pterostigma and anterior half of fore wing dark brown, remainder of wing membrane subhyaline.

*Variation*. Length of body 8.6–12.1 mm, of fore wing 8.4–11.7 mm; antenna of ♀ with 25–28 segments; head largely orange-brown or reddish brown with similar but reduced black markings as in lectotype; clypeus largely yellow with middle dark brown; lower pronotum with or without yellow spot; mesopleuron with or without orange-brown spot; lateral spot on propodeum larger or absent; metasoma extensively varies, yellow stripe on posterior margin of second tergites broader or absent, third–sixth tergites with reddish brown or dark brown stripe on posterior margin, second sternite with broad reddish brown stripes on posterior margin; antenna entirely dark brown; outer side of hind coxa, trochanter, trochantellus and base of femur ivory; length of vein 1-M of fore wing 1.1–1.4 times as long as vein 1-SR; sixth tergite with whitish or yellowish pubescence; head besides stemmaticum with orange transverse patch; frons also posteriorly and partly anteriorly orange.

*Male*. Length of body 7.8–12.4 mm, of fore wing 7.2–11.1 mm; antenna with 25–28 segments, tyloids linear, 0.8 times as long as segment on 10^th^–12^th^ segments and as long as segment on 13^th^–15^th^ segments with a remnant on 16^th^ segment; genitalia internal, sometimes extruded; second sternite with shallow concave and shiny medio-posterior area; males more or less coloured as *Taeniogonalos sauteri*, but sometimes with pale head as females, colour varies as in females.

#### Biology.

Unknown. Collected in April–August and November at 900–1920 m.

#### Distribution.

China (Shaanxi, Henan, Zhejiang, Fujian, Jiangxi, Hubei, Hainan, Guangxi, Sichuan, Guizhou, Yunnan); Korea; Thailand.

### Taeniogonalos tricolorisoma sp. n.

http://zoobank.org/B1D7479E-815D-4A4C-9750-67FA12FEE09A

http://species-id.net/wiki/Taeniogonalos_tricolorisoma

Figs 571 –581

#### Type material.

Holotype, ♂ (ZJUH) “[China:] 2.VI.2005, Guizhou, Leigong county, 1000 m, Hong-ying Zhang, 20059320”. Paratypes: 1 ♂ (ZJUH) “[China:] Guizhou, Mt. Fanjing, 1300 m, 1.VIII.2001, Yun Ma, 200108311”; 1 ♂ (IZCAS) “[China:] Yunnan, Yongsheng, Delu, 2250 m, 9.VII.1984, Jian-guo Fan, IOZ(E) 1903975”.

#### Diagnosis.

Occipital carina narrow, non-lamelliform and smooth medio-dorsally or nearly so (Fig. 573); outer side of supra-antennal elevations oblique and elevations weakly protruding, 0.3 times as long as scapus (Fig. 573); head dorsally punctate with distinct interspaces (Fig. 573), posteriorly entirely black (Fig. 573) or with small yellowish patches and black behind stemmaticum; mesosoma with limited yellowish pattern, scutellum black or largely so (Fig. 578); metasoma dorsally largely orange-brown except first tergite (Fig. 580); second sternite of ♂ distinctly impressed medio-posteriorly and tri-coloured (Fig. 581); second tergite and sternite densely coarsely punctate (Figs 580, 581); fore wing with subapical dark brown patch and remainder subhyaline (Fig. 576).

**Figures 571–573. F110:**
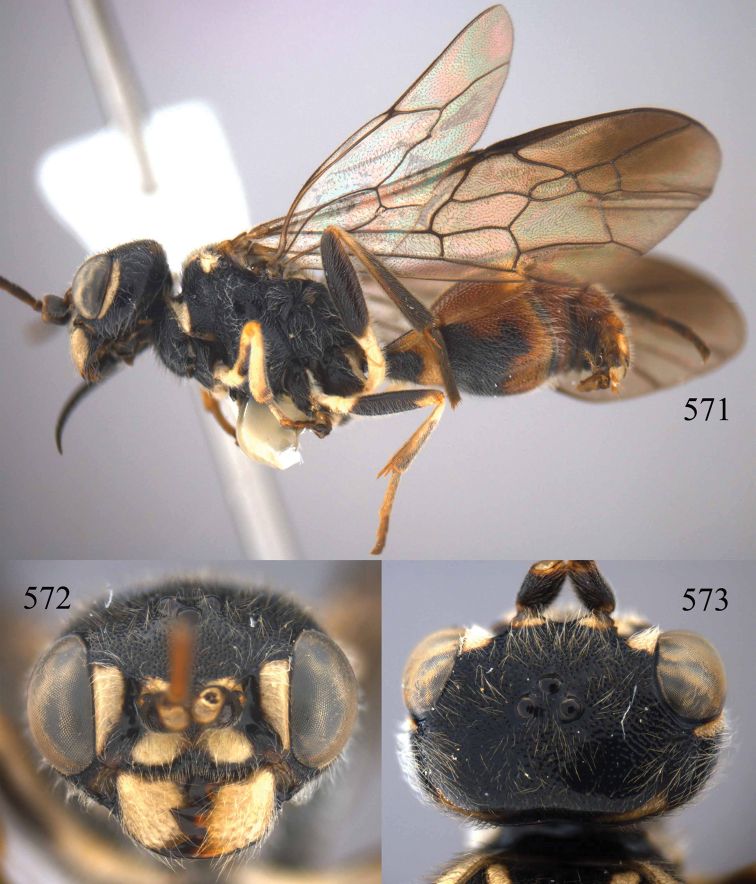
*Taeniogonalos tricolorisoma* sp. n., holotype, male. **571** Habitus lateral **572** head anterior **573** head dorsal.

**Figures 574–581. F111:**
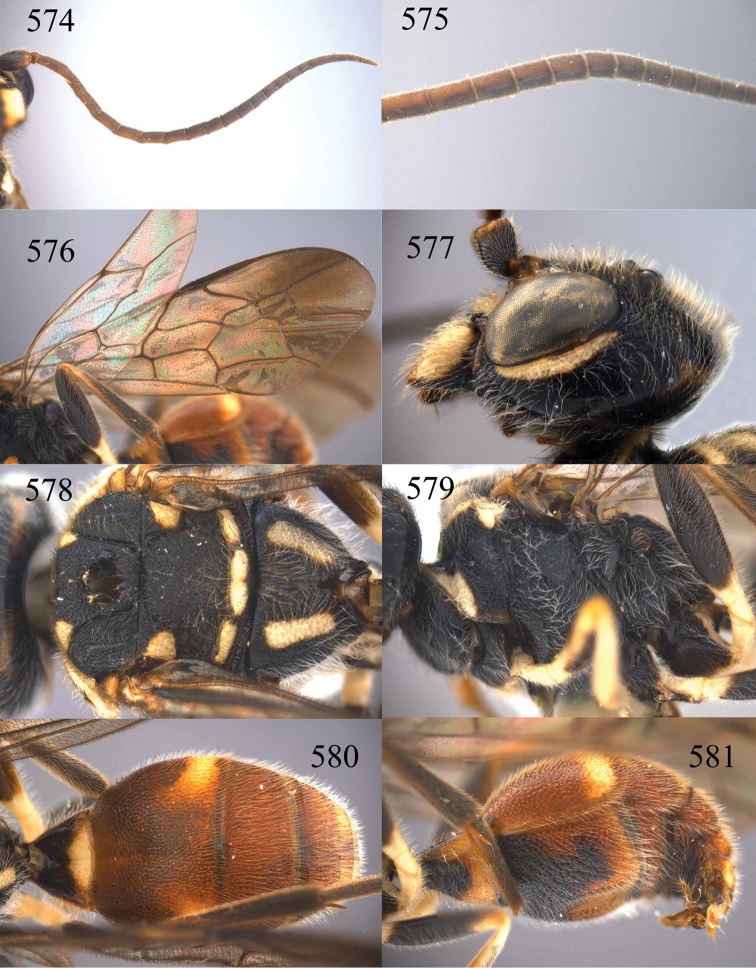
*Taeniogonalos tricolorisoma* sp. n., holotype, male. **574** Antenna **575** tyloids on 10^th^–15^th^ segments of antenna **576** fore and hind wings **577** head lateral **578** mesosoma dorsal **579** mesosoma lateral **580** metasoma dorsal **581** metasoma lateral.

#### Description.

Holotype, male, length of body 10.2 mm (of fore wing 8.9 mm).

*Head*. Antenna with 25 segments, tyloids nearly as long as segment present on 10^th^–13^th^ segments, on 14^th^ and 15^th^ segments shorter tyloids; frons densely punctate and no smooth interspaces; vertex punctate with large smooth interspaces but patch behind stemmaticum and near eyes with narrow interspaces and distinctly shiny (Fig. 573), with rather long setae; temple largely smooth with sparse fine punctures (Fig. 577); head gradually narrowed behind eyes, eye in dorsal view 1.9 times as long as temple (Fig. 573); occipital carina narrow medio-dorsally; supra-antennal elevations medium-sized (about 0.3 times as long as scapus), outer side oblique and largely smooth except for sparse punctures; clypeus slightly concave and thick medio-ventrally.

*Mesosoma*. Length of mesosoma 1.3 times its height (Fig. 579); mesopleuron densely and finely rugulose-punctate, with satin sheen; notauli narrow, deep and largely smooth; mesoscutum largely finely and densely reticulate-rugose and with satin sheen, rather contrasting with shiny head (Fig. 578); scutellar sulcus complete, moderately wide and crenulate; scutellum densely reticulate-rugose, convex anteriorly and flattened posteriorly and near level of mesoscutum; metanotum medially protruding, obtuse and densely reticulate-rugose (Fig. 578); propodeum largely reticulate-rugose, smooth and shiny posteriorly and medially densely rugulose (Fig. 578); arched posterior propodeal carina thick lamelliform.

*Metasoma*. First tergite 0.6 times as long as apically wide and with shallow elliptical depression medially (Fig. 580); second tergite and sternite (as other tergites except first one and sternites) densely and coarsely punctate (Figs 580, 581); second sternite with wide and rather shallow depression medio-posteriorly; genitalia extruded (Fig. 581).

*Colour*. Black; head anteriorly with extensive pale yellow pattern (Fig. 572); remainder of head black except pale yellow outer orbita and pair of posterior patches on the vertex pale yellow (Fig. 573); mesosoma laterally black except for anterior and dorsal yellow patches of pronotal side; middle lobe of mesoscutum with pair of wide pale yellow patches anteriorly; axilla with large pale yellow patch; metanotum with one large medial and pair of large lateral pale yellow patches; propodeum with pair of large elongate pale yellow patches (Fig. 578); metasoma dorsally orange-brown, first tergite posteriorly largely pale yellow and remainder black, second tergite postero-laterally, fifth tergite laterally and most of sixth tergite (except medial brown patch) pale yellow; metasoma ventrally tricoloured (Fig. 581): first sternite black basally, narrowly orange-brown and yellow apically, second sternite black, medio-posteriorly and laterally orange-brown except for narrow yellow apical margin, remainder of metasoma ventrally mainly orange-brown; palpi and antenna (but ventrally brown) dark brown; patch on middle and hind coxae, fore and middle trochanters largely, anterior stripe on fore femur, base of all femora and apex of fore and middle femora, fore tibia anteriorly, base of middle and hind tibiae pale yellow; remainder of tibiae and tarsi more or less dark brown; pterostigma and apical half of marginal cell of fore wing and area below it dark brown; remainder of wing membrane slightly infuscate.

*Female*. Unknown.

*Variation*. Paratype from Guizhou: length of body 8.2 mm and of fore wing 7.0 mm, antenna with 25 segments and tyloids similar to holotype; pale patches of vertex and of mesoscutum slightly smaller than of holotype and orange-brown part of second sternite is smaller. The paratype from Yunnan has the pale patches of vertex and of mesoscutum absent and median patch of metanotum reduced to 2 small pale patches.

#### Biology.

Unknown. Collected in June–August at 1000–2250 m.

#### Distribution.

China (Guizhou, Yunnan).

#### Etymology.

Named after the tricoloured ventral aspect of the metasoma.

### Taeniogonalos uncifera sp. n.

http://zoobank.org/3168CD05-7FA7-4C09-BC11-05AEE58D8397

http://species-id.net/wiki/Taeniogonalos_uncifera

Figs 582 –592

#### Type material.

Holotype, ♀ (IZCAS) “[China:] Sichuan, Mt. Emei, Baoguosi, 550–750 m, 5.VII.1957, Ke-ren Huang, IOZ(E)1495445”. Paratypes: 2 ♀ (IZCAS), same data as holotype, IOZ(E)1495246, IOZ(E)14952467; 1 ♀ (IZCAS) “[China:] Sichuan, Mt. Emei, Qingyingge, 800–1000 m, 11.VII.1957, You-cai Lu, IOZ(E)1495245”.

#### Diagnosis.

Outer side of supra-antennal elevations oblique and elevations about 0.4 times as long as scapus (Fig. 584); occipital carina strongly widened medio-dorsally and distinctly lamelliform (Fig. 584); head posteriorly entirely black and dorsally punctate with distinct smooth interspaces (Fig. 584); middle mesoscutal lobe similarly coloured to lateral lobes, black or yellow laterally and black medially (Fig. 588); anterior half of fore wing membrane dark brown (Fig. 586); mesosoma entirely black (Fig. 590); second sternite of female distinctly convex (Fig. 591; probably less of ♂); third sternite of female with large hook-shaped protuberance (Fig. 591); metasoma with limited pale pattern (Fig. 590).

**Figures 582–584. F112:**
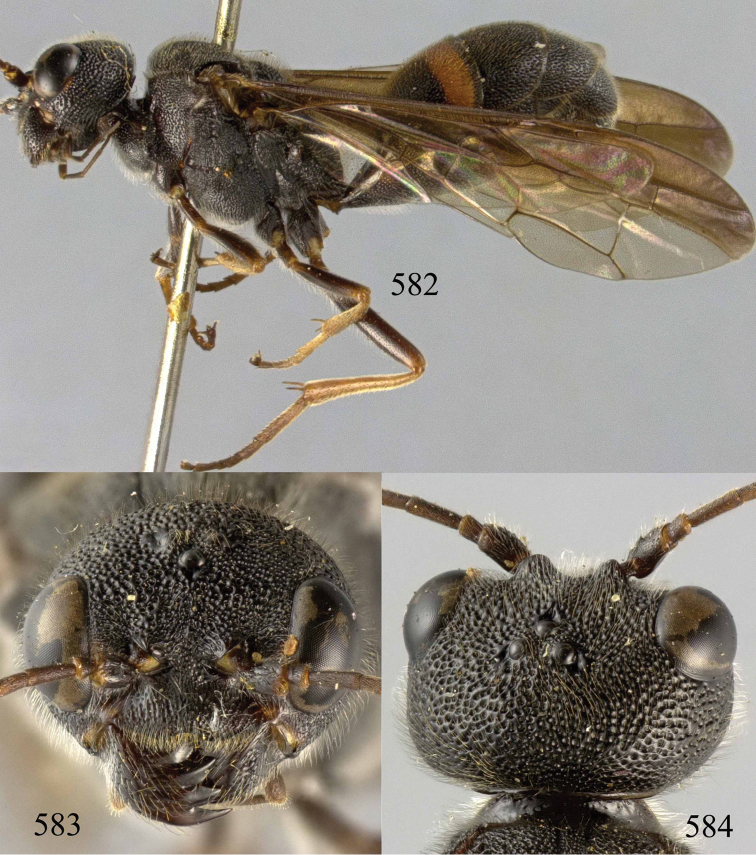
*Taeniogonalos uncifera* sp. n., holotype, female. **582** Habitus lateral **583** head anterior **584** head dorsal.

**Figures 585–592.**
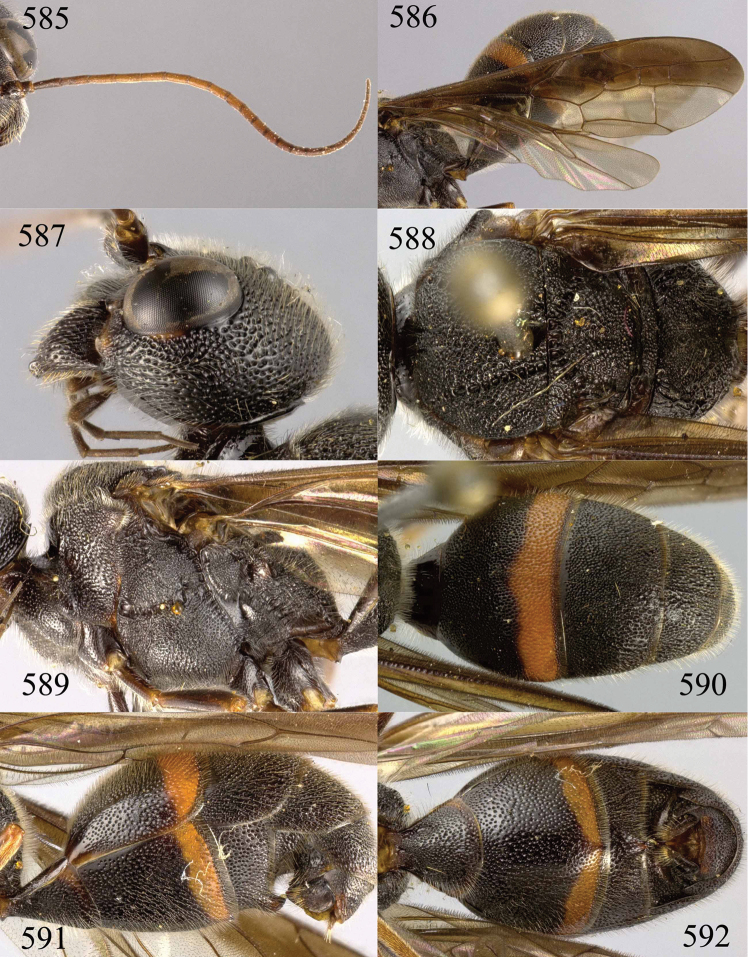
*Taeniogonalos uncifera* sp. n., holotype, female. **585** Antenna **586** fore and hind wings **587** head lateral **588** mesosoma dorsal **589** mesosoma lateral **590** metasoma dorsal **591** metasoma lateral **592** metasoma ventral.

#### Description.

Holotype, female, length of body 14.2 mm (of fore wing 12.0 mm).

*Head*. Antenna with 25 segments; frons, vertex and temple coarsely reticulate-punctate (Figs 583, 584, 587); head gradually narrowed behind eyes, eye in dorsal view 0.7 times as long as temple (Fig. 584); occipital carina narrowly lamelliform and smooth medio-dorsally; supra-antennal elevations medium-sized (about 0.4 times as long as scapus), outer side oblique and rugulose; clypeus slightly concave and thick medio-ventrally.

*Mesosoma*. Length of mesosoma 1.4 times its height (Fig. 589); mesopleuron coarsely reticulate-punctate, above transverse mesopleural groove with large foveolae medio-dorsally; notauli moderately wide, deep and coarsely crenulate; mesoscutum coarsely reticulate-punctate (Fig. 588); scutellar sulcus complete, moderately wide and crenulate; scutellum coarsely reticulate-punctate, convex anteriorly and near level of mesoscutum, with a shallow furrow medially; metanotum medially slightly convex, not protruding and densely reticulate-punctate (Fig. 588); propodeum coarsely reticulate-rugose, rugae less coarse medially (Fig. 588); posterior propodeal carina thick lamelliform and slightly arched (about twice as wide as high medially).

*Wings*. Fore wing: length of vein 1-M 1.4 times as long as vein 1-SR (Fig. 586).

*Metasoma*. First tergite 0.7 times as long as apically wide, smooth and with distinct elliptical depression medially (Fig. 590); second–sixth tergites coarsely reticulate-rugose; sternites densely and coarsely punctate; second sternite distinctly convex in lateral view, its medio-apical protuberance small and acute medio-apically (Fig. 591); third sternite about 0.3 times as long as second sternite, with a large hook-shaped triangular protuberance medially (Figs 591, 592); hypopygium triangular in ventral view (Fig. 592).

*Colour*. Black; palpi dark brown; mandible dark brown; antenna dark brown; frons with narrow yellowish brown stripes along inter orbita, remainder of head black; tegulae dark brown; legs dark brown to black with tibia and tarsi paler; metasoma with moderate wide orange-brown apical band at apex of second tergite and sternite; pterostigma and anterior half of fore wing dark brown, ventral half subhyaline.

*Variation*. Length of body13.1–14.4 mm, of fore wing 10.0–12.0 mm; mandible black; length of vein 1-M of fore wing 1.3–1.5 times as long as vein 1-SR.

*Male*. Unknown.

#### Biology.

Unknown. Collected in July at 550–1000 m.

#### Distribution.

China (Sichuan).

#### Etymology.

Named after the hook-shaped protuberance of the third sternite: from “*uncus*” (Latin for “hook”) and “*fera*” (Latin suffix for “carry, have”).

### Teranishia

Tsuneki, 1991

http://species-id.net/wiki/Teranishia

**Figures 593–595.**
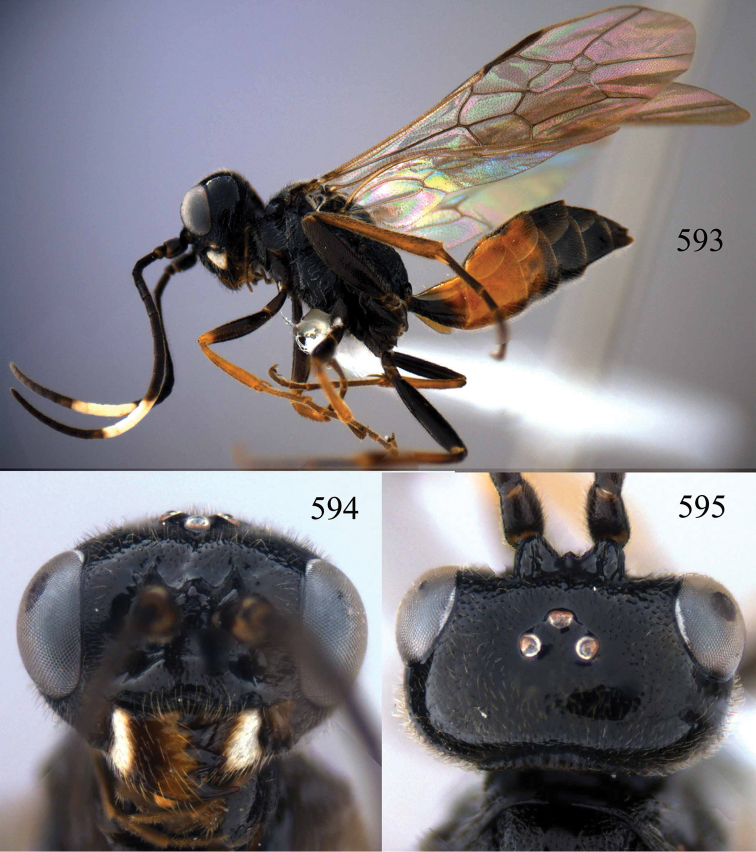
*Teranishia crenulata* sp. n., holotype, female. **593** Habitus lateral **594** head anterior **595** head dorsal.

Figs 593 [Fig F115] [Fig F116] [Fig F117] –616

Teranishia Tsuneki, 1991: 15; Lelej 1995: 12; 2003: 3; Carmean and Kimsey 1998: 73. Type species (by original designation): *Teranishia nipponica* Tsuneki, 1991.

#### Diagnosis.

Length of body 6.0–12.0 mm; antenna black and with 24–27 segments; area above supra-antennal elevations flat, more or less punctate, with protuberance between elevations and inner side of supra-antennal elevations flat, smooth and black (Figs 595, 606); tyloids on 11^th^–14^th^ antennal segments of male absent; occipital carina widened medio-dorsally; apical segment of labial palp widened and obtuse, more or less triangular; vertex normal, at most with slight median depression dorsally (Fig. 595); mandibles wide in anterior view and sublaterally attached to head (Fig. 594); anterior propodeal sulcus distinctly crenulate (Fig. 599); metanotum strongly convex and finely sculptured medially (Fig. 599); anterior propodeal sulcus crenulate and medially widened (Figs 599, 610); posterior propodeal carina curved and distinctly protruding and more or less separated from foramen medio-dorsally (Figs 599, 610); fore wing with large dark patch below pterostigma; vein 1-SR of fore wing long (Figs 597, 608); hind trochanter black, dark brown or ivory; hind tarsus slightly or not modified; second and third sternites of ♀ flat and moderately sclerotized and no protuberances (Fig. 603); body without pale pattern, at most malar space and margins of basal metasomal sternites and tergites narrowly ivory, remainder black.

**Figures 596–603. F115:**
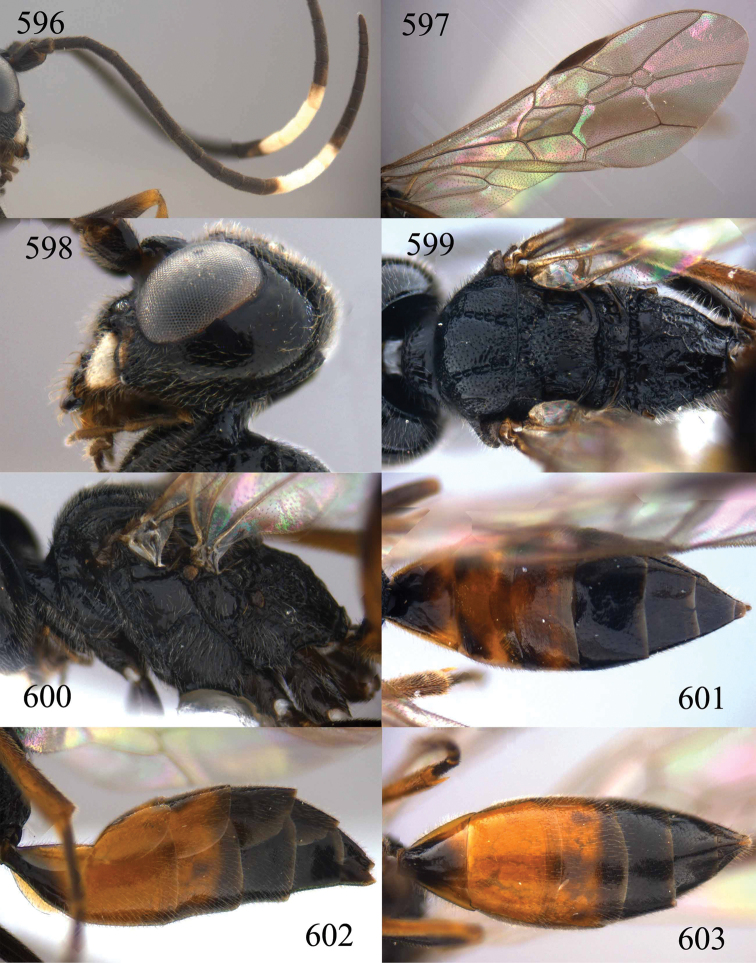
*Teranishia crenulata* sp. n., holotype, female. **596** Antennae **597** fore wing **598** head lateral **599** mesosoma dorsal **600** mesosoma lateral **601** metasoma dorsal **602** metasoma lateral **603** metasoma ventral.

**Figures 604–606. F116:**
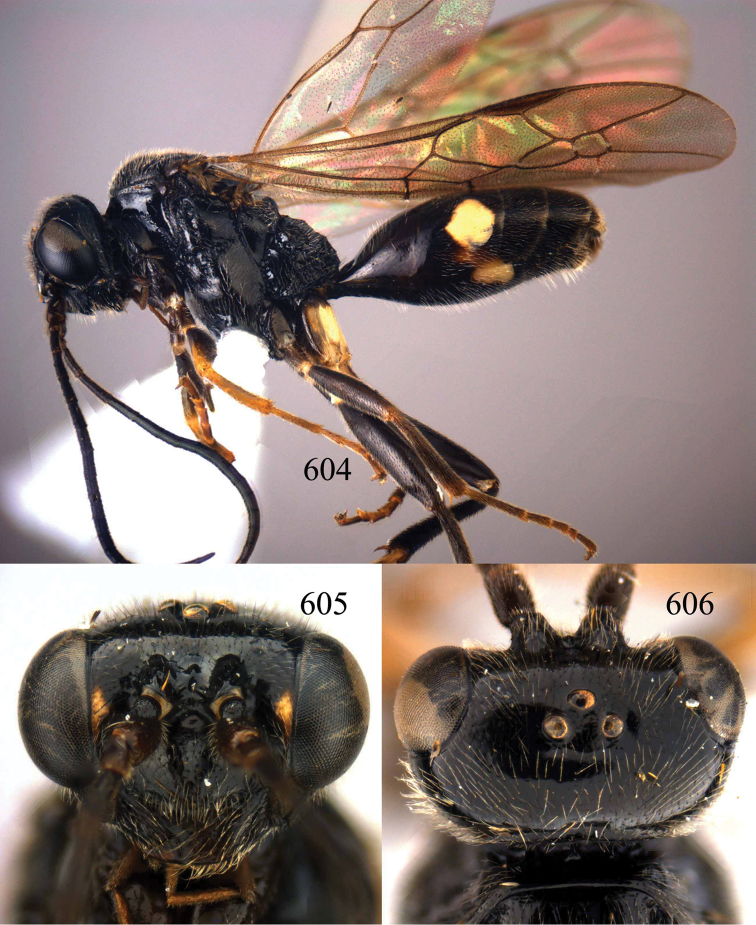
*Teranishia glabrata* sp. n., holotype, female. **604** Habitus lateral **605** head anterior **606** head dorsal.

**Figures 607–614. F117:**
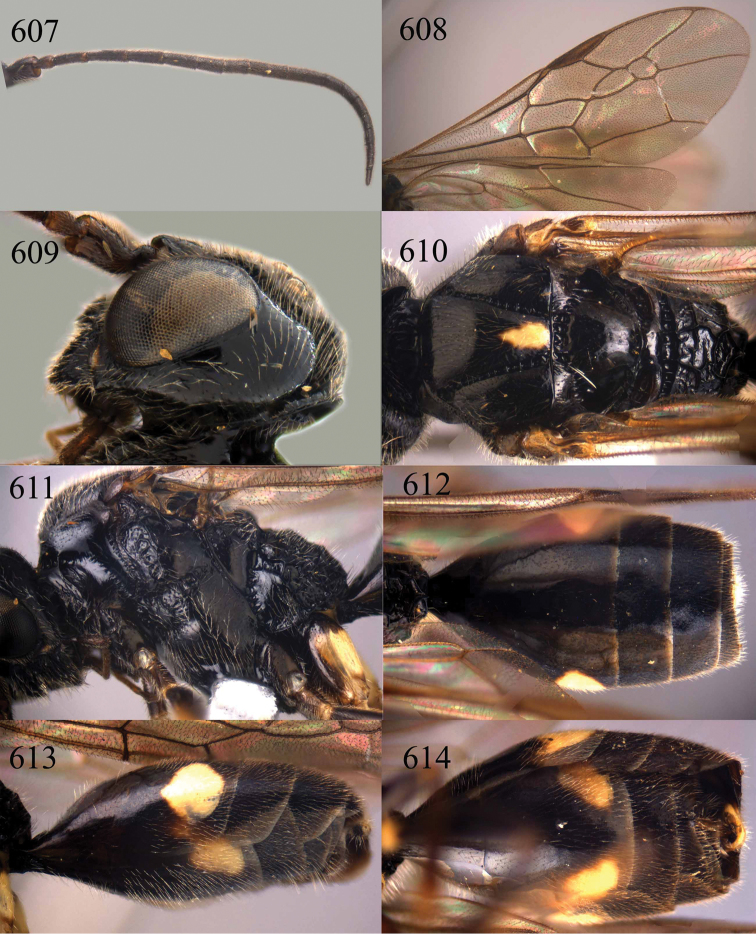
*Teranishia glabrata* sp. n., holotype, female. **607** Antenna **608** fore and hind wings **609** head lateral **610** mesosoma dorsal **611** mesosoma lateral **612** metasoma dorsal **613** metasoma lateral **614** metasoma ventral.

**Figures 615–616.**
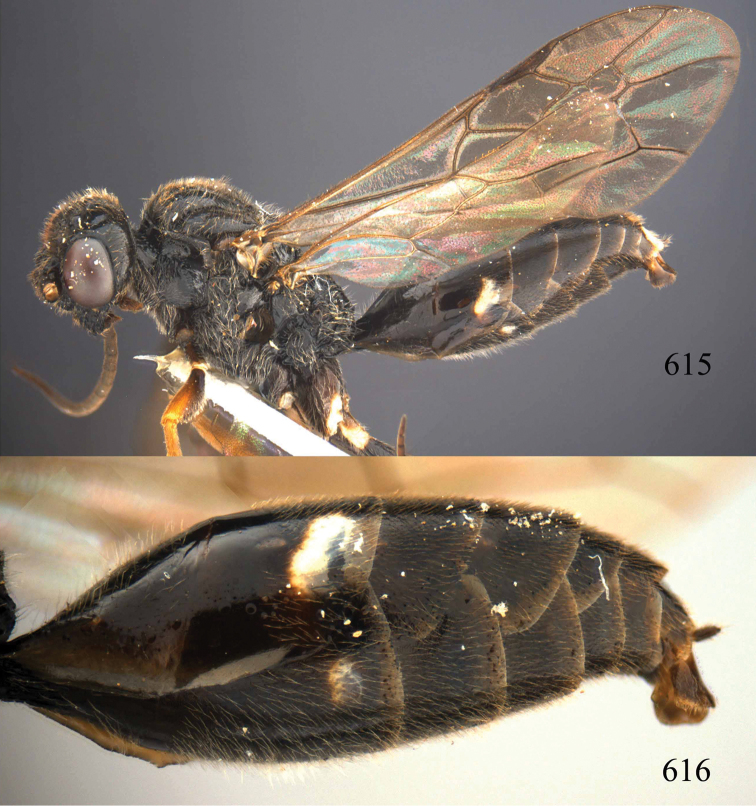
*Teranishia glabrata* sp. n., paratype, male from Zhejiang. **615** Habitus lateral **616** metasoma lateral.

#### Notes.

According to Lelej (2003) the female paratypes (not male holotype) of *Teranishia nipponica* belong to *Teranishia marujamae* Tsuneki, 1991. However, in the original description of *Teranishia nipponica* is indicated that the supra-antennal elevations have a medial yellow spot, which is absent in the holotype of *Teranishia marujamae*; therefore, we refrain from accepting this synonymy.

#### Key to species of *Teranishia* Tsuneki, 1991

**Table:** 

1	Supra-antennal elevations black apically and yellow medially; frons convex medio-anteriorly and more or less lobe-shaped protruding between supra-antennal elevations (Fig. 48 in Tsuneki 1991); Japan and not yet found in China	*Teranishia nipponica* Tsuneki, 1991
–	Supra-antennal elevations apically ivory, yellow or black (Figs 595, 606), if black apically then also black medially; frons flat medio-anteriorly or nearly so and not protruding between supra-antennal elevations (Figs 594, 605)	2
2	Antenna with a small ivory band subapically (Fig. 596); occipital carina strongly developed and finely crenulate dorsally (Fig. 595); scutellum densely rugose or rugulose (Fig. 599); frons densely punctate (Fig. 594), but sometimes reduced	*Teranishia crenulata* sp. n.
–	Antenna without pale band subapically (Fig. 607); occipital carina comparatively weakly developed and smooth dorsally (Fig. 606); scutellum smooth (Fig. 610); frons strongly shiny and smooth (Fig. 605); [mesoscutum sometimes with central ivory patch]	*Teranishia glabrata* sp. n.

### Teranishia crenulata sp. n.

http://zoobank.org/83BD156C-53D5-4A37-86A7-BD7960546826

http://species-id.net/wiki/Teranishia_crenulata

Figs 593 –603

#### Type material.

Holotype, ♀ (SCAU) “[China:] Ningxia, Mt. Liupan, 21.VI–14.VII.2008, Jie-min Yao, 200808062”. Paratypes: 1 ♂ (SCAU), same data, but 200808083; 1 ♂ (SCAU), “[China:] Ningxia, Mt. Liupan, Fengtai, 28.VI.2008, Jing-xian Liu, 200800605”; 1 ♂ (RMNH) “[China:] Ningxia, Mt. Liupan, Sutai, 26.VI.2008, Jing-xian Liu, 200800529”; 1 ♂ (ZJUH) “[China:] Gansu, Dangchang, Daheba, 2539 m, 31.VII.2004, Qiong Wu, 20047026”; 1 ♂ (ZJUH) “[China:] Sichuan, Pingwu, Baimazhai, 25.VII.2006, Hong-ying Zhang, 200611109”; 1 ♂ (SCAU) “[China:] Sichuan, Wanglang National Nature Reserve, 2500 m, 25.VII.2006, sweeping, Zhuang Lu, SCAU 356”.

#### Diagnosis.

Antenna with a small ivory band subapically (Fig. 596); occipital carina strongly developed and finely crenulate dorsally (Fig. 595); frons densely punctate (Fig. 594), but sometimes reduced; scutellum densely rugose or rugulose (Fig. 599); mesosoma without pale pattern dorsally, mesoscutum black medially (Fig. 599); third submarginal cell 0.4–0.5 times as long as second submarginal cell; first tergite black, largely brownish-white or with a reddish-yellow lateral patch apically (Fig. 601).

#### Description.

Holotype, female, length of body 6.9 mm (of fore wing 5.2 mm).

*Head*. Antenna with 23 segments; frons densely and finely punctate; vertex sparsely and finely punctate and becoming smooth posteriorly (Fig. 595); temple largely smooth with sparse fine punctures; head subparallel-sided behind eyes, eye in dorsal view 0.9 times as long as temple (Fig. 595); occipital carina strongly developed and finely crenulate dorsally (Fig. 595); supra-antennal elevations strongly enlarged (about 0.7 times as long as scapus), outer side subvertical and largely smooth except for sparse punctures (Fig. 595); clypeus slightly concave and thick medio-ventrally.

*Mesosoma*. Length of mesosoma 1.6 times its height (Fig. 600); mesopleuron below transverse mesopleural groove coarsely rugose antero-dorsally, smooth ventrally and transversely striate postero-dorsally, above groove coarsely rugose anteriorly and smooth posteriorly; transverse mesopleural groove wide, deep and coarsely crenulate; notauli wide, deep and coarsely crenulate; mesoscutum densely punctate rugose, lateral lobe with a narrow smooth mid-longitudinal line (Fig. 599); scutellar sulcus wide, both medially and laterally and coarsely crenulate; scutellum densely rugose and anteriorly near level of scutellum (Fig. 599); metanotum medially protruding, obtuse and rugose (Fig. 599); propodeum irregularly areolate (Fig. 599); posterior propodeal carina thick lamelliform and slightly arched, foramen medially 0.5 times higher than wide basally.

*Wings*. Fore wing: length of vein 1-M 2.4 times as long as vein 1-SR (Fig. 597).

*Metasoma*. First tergite 0.7 times as long as apically wide, smooth and without depression antero-medially (Fig. 601); other tergites and sternites smooth to superficially coriaceous and shiny (Figs 601, 603); third sternite about 0.7 times as long as second sternite (Fig. 603); hypopygium triangular in ventral view (Fig. 603).

*Colour*. Black; malar space brownish; postero-lateral margin of first tergite and sternite, second and third tergites and sternites largely reddish-yellow; palpi dark brown; mandible with large basal ivory spot, otherwise orange-brown to dark brown; antenna black with a small ivory band subapically; apex of fore femur, tibia and tarsus, ventral side of mid and hind tibiae yellowish brown, remainder of legs dark brown to black; pterostigma dark brown; wing membrane subhyaline.

*Male*. Length of body 6.2–10.2 mm, of fore wing 5.1–8.1 mm; antenna with 23 (2), 24 (2) or 25 (2) segments; frons densely and coarsely punctate; vertex densely and finely punctate; frons with ivory stripes along inner orbita; dorsal half of clypeus ivory; colour of metasoma similar to female or darker; genitalia extruded.

#### Biology.

Unknown. Collected in June–July.

#### Distribution.

China (Ningxia, Gansu, Sichuan).

#### Etymology.

Named after the finely crenulate occipital carina: from “*crenulatus*” (Latin for “minutely crenate or notched”).

### Teranishia glabrata sp. n.

http://zoobank.org/704E0EF8-8C8B-4BBE-9232-8826C97AE6AD

http://species-id.net/wiki/Teranishia_glabrata

Figs 604 – 616

#### Type material.

Holotype, ♀ (ZJUH) “[China:] Henan, Neixiang, Mt. Baotianman, 15.VII.1998, Yun Ma, 986723”. Paratypes: 1 ♂ (ZJUH), “[China:] Ningxia, Mt. Liupan, Hongxia Forestry Farm, 2.VII.2008, Jing-xian Liu, 200800812”; 1 ♀ (ZJUH), “[China:] Henan, Song County, Mt. Baiyun, 1400 m, 26.VII.2003, Yan-bing Shen, 20047321”; 1 ♀ + 2 ♂ (ZJUH, RMNH), “[China:] Zhejiang, Mt. Tianmu, Xianrending, 1509 m, 1.VII.2001, Mei-hua Piao, 200106405, 200106406; id., but 2.VII.2000, Yun Ma”; 1 ♀ (ZJUH), “[China:] Sichuan, Wolong National Nature Reserve, 21.VII.2006, Hong-ying Zhang, 200610791”.

#### Diagnosis.

Antenna without ivory band subapically (Fig. 607); occipital carina weakly developed and smooth dorsally (Fig. 606); frons strongly shiny and smooth (Fig. 605); scutellum largely smooth, with longitudinal depression and strongly shiny (Fig. 610); mesosoma without pale pattern dorsally, mesoscutum black medially (Fig. 610); third submarginal cell 0.4–0.5 times as long as second submarginal cell; first tergite metasomal black, largely brownish-white or with a reddish-yellow lateral patch apically (Fig. 612); second tergite usually with ivory patch laterally (Fig. 612).

#### Description.

Holotype, female, length of body 7.5 mm (of fore wing 6.8 mm).

*Head*. Antenna with 20 segments; frons, vertex and temple smooth and shiny (Figs 605, 606, 609); head gradually narrowed behind eyes, eye in dorsal view 1.2 times as long as temple (Fig. 606); occipital carina weakly developed and smooth dorsally (Fig. 606); supra-antennal elevations medium-sized (about 0.4 times as long as scapus), outer side subvertical and obliquely and weakly striate (Fig. 606); clypeus hardly concave and very thick medio-ventrally.

*Mesosoma*. Length of mesosoma 1.4 times its height (Fig. 611); mesopleuron below transverse mesopleural groove coarsely rugose antero-dorsally and smooth ventrally and posteriorly, above groove coarsely rugose anteriorly and smooth posteriorly; transverse mesopleural groove wide, deep and coarsely crenulate; notauli moderately wide, deep and coarsely crenulate; mesoscutum smooth and strongly shiny, lateral lobe with a narrow and shallow mid-longitudinal furrow (Fig. 610); scutellar sulcus wide, both medially and laterally and coarsely crenulate; scutellum largely smooth, with longitudinal depression and strongly shiny, anteriorly near level of scutellum (Fig. 610); metanotum medially slightly convex, not protruding and smooth (Fig. 610); propodeum irregularly areolate (Fig. 610); posterior propodeal carina thick lamelliform and strongly arched, foramen medially 0.7 times higher than wide basally.

*Wings*. Fore wing: length of vein 1-M 2.8 times as long as vein 1-SR (Fig. 608).

*Metasoma*. First tergite 0.4 times as long as apically wide, smooth and with shallow depression antero-medially (Fig. 612); other tergites and sternites smooth to superficially coriaceous and shiny (Figs 612, 614); third sternite about 0.3 times as long as second sternite (Fig. 614); hypopygium triangular in ventral view.

*Colour*. Head black with short ivory stripes along inner orbita; mesosoma black except for middle lobe with central ivory patch posteriorly; metasoma largely black with large ivory spots on postero-lateral margin of second tergite and sternite; palpi dark brown; mandible black; antenna black; apex of fore femur, tibia and tarsus yellowish brown, outer side of hind coxa, hind trochanter and trochantellus ivory, remainder of legs dark brown to black; pterostigma of fore wing dark brown, remainder of wing membrane subhyaline.

*Variation*. Length of body 7.0–8.2 mm, of fore wing 5.7–7.0 mm; frons entirely black or with very faint ivory stripes; mesosoma entirely black or with faint and small central ivory patch; ivory spots on second tergite very small and absent on second sternite; outer surface of hind coxa black and all legs darker; length of vein 1-M of fore wing 2.8–3.2 times as long as vein 1-SR.

*Male*. Length of body 8.0–10.2 mm, of fore wing 6.7–8.1 mm; antenna with 20 segments; genitalia extruded (Fig. 616); otherwise similar to female.

#### Biology.

Unknown. Collected in July at 1400–1509 m.

#### Distribution.

China (Ningxia, Henan, Zhejiang, Sichuan).

#### Etymology.

Named after the largely smooth and shiny frons and scutellum: from “*glaber*” (Latin for “hairless, bald, smooth”).

**Table 3.** List of species of Trigonalyidae known from China (valid species in bold).

*alishana* Tsuneki, 1991 => *maga*
***alticola*** (Tsuneki, 1991), **re-instated**
***angusta* sp. n.**
*anglicana* Shuckard, 1841 => *hahnii*
***angustula* sp. n.**
*aterrima* Eversmann, 1849 => *hahnii*
***bucarinata* sp. n.**
***cheni* sp. n.**
*claripennis* Tsuneki, 1991 => *alticola* **syn. n.**
***clypeata* sp. n.**
***cordata* sp. n.**
***crenulata* sp. n.**
***elliptifera* sp. n.**
***elongata*** Teranishi, 1929
*enslini* Torka, 1936 => *hahnii*
*europaea* Westwood, 1861 => *hahnii*
***fasciata*** (Strand, 1913)
*flavocincta* Teranishi, 1929 => *subtruncata*
***flavonigrata* sp. n.**
***flavoscutellata*** (Chen, 1949), **comb. n., re-instated**
***formosana*** (Bischoff, 1913) (*Taeniogonalos*)
***formosana*** Teranishi, 1931 (*Orthogonalys*)
***geminata* sp. n.**
***gestroi*** (Schulz, 1918), **comb. n., re-instated**
***glabrata* sp. n.**
***hahnii*** (Spinola, 1840)
***huisuni*** Yamane & Yamane, 1975
*intermedia* Chen, 1949 => *formosana* (*Taeniogonalos*) **syn. n.**
*interrupta* Chen, 1949 => *fasciata*
***jiangliae* sp. n.**
***laeviceps*** (Tsuneki, 1991), **comb. n., re-instated**
***luteata* sp. n.**
*macquartii* Guérin-Méneville, 1844 => *hahnii*
***maga*** (Teranishi, 1929)
*magnifica* Teranishi, 1929 => *fasciata*
*minima* Tsuneki, 1991 => *alticola* **syn. n.**
***mongolica*** (Popov, 1945)
*nigra* Westwood, 1843 => *hahnii*
***nigralva* sp. n.**
***nigrata* sp. n.**
*phaeognatha* Enderlein, 1905 => *hahnii*
*pictipennis* Strand, 1914 => *sauteri*
*prudnicensis* Torka, 1936 => *hahnii*
***robusta* sp. n.**
*rubrothoracica* Bischoff, 1913 => *fasciata*
***rufofasciata*** (Chen, 1949)
***satoi*** (Tsuneki, 1991), **comb. n., re-instated**
***sauteri*** Bischoff, 1913
***seidakka*** Yamane & Terayama, 1983, **re-instated**
***sculpturata* sp. n.**
*similis* Tsuneki, 1991 => *alticola* **syn. n.**
*solitaria* Jacobs, 1878 => *hahnii*
***subtruncata* nom. n.**
***taihorina*** (Bischoff, 1914)
*taiwana* Tsuneki, 1991 => *taihorina* **syn. n.**
*thwaitesii* auctt. (not Westwood, 1874) => *gestroi*
***triangulata* sp. n.**
***tricolor*** (Chen, 1949)
***tricolorisoma* sp. n.**
***uncifera* sp. n.**
*unifasciata* Chen, 1949 => *formosana* (*Taeniogonalos*) **syn. n.**
***violaceipennis*** Chen, 1949

## Supplementary Material

XML Treatment for Trigonalyidae

XML Treatment for Bakeronymus

XML Treatment for Bakeronymus seidakka

XML Treatment for Bareogonalos

XML Treatment for Bareogonalos huisuni

XML Treatment for Jezonogonalos

XML Treatment for Jezonogonalos elliptifera

XML Treatment for Jezonogonalos jiangliae

XML Treatment for Jezonogonalos laeviceps

XML Treatment for Jezonogonalos luteata

XML Treatment for Jezonogonalos nigrata

XML Treatment for Jezonogonalos satoi

XML Treatment for Lycogaster

XML Treatment for Lycogaster angustula

XML Treatment for Lycogaster flavonigrata

XML Treatment for Lycogaster nigralva

XML Treatment for Lycogaster violaceipennis

XML Treatment for Orthogonalys

XML Treatment for Orthogonalys cheni

XML Treatment for Orthogonalys clypeata

XML Treatment for Orthogonalys elongata

XML Treatment for Orthogonalys formosana

XML Treatment for Orthogonalys robusta

XML Treatment for Pseudogonalos

XML Treatment for Pseudogonalos angusta

XML Treatment for Pseudogonalos hahnii

XML Treatment for Taeniogonalos

XML Treatment for Taeniogonalos alticola

XML Treatment for Taeniogonalos bucarinata

XML Treatment for Taeniogonalos cordata

XML Treatment for Taeniogonalos fasciata

XML Treatment for Taeniogonalos flavoscutellata

XML Treatment for Taeniogonalos formosana

XML Treatment for Taeniogonalos geminata

XML Treatment for Taeniogonalos gestroi

XML Treatment for Taeniogonalos maga

XML Treatment for Taeniogonalos mongolica

XML Treatment for Taeniogonalos rufofasciata

XML Treatment for Taeniogonalos sauteri

XML Treatment for Taeniogonalos sculpturata

XML Treatment for Taeniogonalos subtruncata

XML Treatment for Taeniogonalos taihorina

XML Treatment for Taeniogonalos triangulata

XML Treatment for Taeniogonalos tricolor

XML Treatment for Taeniogonalos tricolorisoma

XML Treatment for Taeniogonalos uncifera

XML Treatment for Teranishia

XML Treatment for Teranishia crenulata

XML Treatment for Teranishia glabrata
